# Selective Scission of Orthogonal Bonds in Four‐Membered Ring Mechanophores upon Activation by a Rotaxane Actuator

**DOI:** 10.1002/anie.202511039

**Published:** 2025-06-18

**Authors:** Lei Chen, Guillaume De Bo

**Affiliations:** ^1^ Department of Chemistry University of Manchester Oxford Road Manchester M13 9PL UK

**Keywords:** Controlled release, Force, Mechanophore, Retrocycloaddition, Rotaxane

## Abstract

Mechanophores (mechanosensitive molecules) are usually activated by pulling force transduced by polymer covalently attached to their structures. A variety of mechanophores have been described to date but many rely on a limited number of core structures. The diversity of products formed mechanochemically could be improved by promoting the scission of varied bonds within a single mechanophore. 4‐membered rings are particularly suited for this as two sets of products can be obtained by formal retro‐[2 + 2] cycloaddition depending on the pulling direction. This concept has been applied to a couple of mechanophores but the repositioning of the anchoring points often requires a redesign of the structures, and the pulling activation is not amenable to the release of the products. Here we show that the unique pushing activation of a rotaxane actuator enables the selective activation of covalent bonds parallel or perpendicular to the main chain in diazetidinone and β‐lactam mechanophores. The selectivity depends on the orientation of the bulky substituents pointing toward or away from the incoming macrocycle. We were able to release ketene and triaryl imine/ethene molecules, and to generate imine, azobenzene, and isocyanate units. This alternative activation method demonstrates how to elicit new reactivity from known mechanophores and should inspire the design of new ones. The variety of structures released/generated here illustrate how this approach can be used for the release of reactive species and other functional molecules.

## Graphical abstract

A rotaxane actuator controls bond scission—perpendicular or parallel to the axle—based on bulky group orientation in a 4‐membered ring. This allows diverse functional molecules to be released from a single core structure, expanding the molecular variety obtainable from new or existing mechanophores.

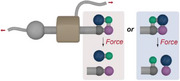

Since the emergence of the mechanophore concept,^[^ ^1^ ^]^ a variety of ingenious force‐sensitive molecules (i.e., mechanophores) have been devised for applications such as: damage reporting,^[^ ^2^ ^]^ catalysis,^[^ ^3^ ^]^ and controlled release.^[^ ^4^ ^]^ However, most are built on a limited selection of structural cores,^[^ ^5^ ^]^ the most popular being the strained 3‐ and 4‐membered rings.^[^ ^6^, ^7^ ^]^ An interesting way to increase the diversity of products that can be obtained from a defined mechanophoric core, is to vary the position of the pulling points to load different (orthogonal) bonds. Examples of such orthogonal bond activation have been demonstrated in the benzocyclobutene (BCB) mechanophore, which can generate an *ortho*‐quinodimethide intermediate upon electrocyclic ring opening^[^ ^8^ ^]^ or an aryne upon formal retro‐[2 + 2] cycloaddition^[^ ^9^ ^]^ of orthogonal bonds. Similarly, the mechanical activation of a bicyclo[2.2.0]‐hexene (BCH) core has been shown to undergo a r[2 + 2], an electrocyclic ring opening, or a 1,3‐allylic migration depending on the bond activated.^[^ ^10^ ^]^ Ring expansion and ring opening products have been obtained from a ladderane mechanophore as a function of the rung activated.^[^ ^11^ ^]^ Though elegant, this approach often requires a substantial redesign of the mechanophore to allow the installation of new anchoring points to the core structure. Moreover, a pulling mode of activation, where actuating polymers are covalently attached to the mechanophore, is not easily amenable to force‐controlled release applications.^[^ ^4^ ^]^ The presence of a mechanical bond in a polymer chain can have profound effects on its mechanical strength,^[^ ^12^, ^13^, ^14^, ^15^ ^]^ and we have shown that the pushing activation of a rotaxane actuator, in which the macrocycle pushes against the mechanophore until activation occurs, is unique in its ability to trigger mechanochemical reactions.^[^ ^16^ ^]^ Based on this principle, we have recently described a molecular device able to efficiently release a variety of cargo molecules appended to its axle.^[^ ^17^, ^18^ ^]^

Here we take advantage of the unique ability of rotaxane actuators to activate bonds by pushing, combined with the propensity of 4‐membered rings to break orthogonally along their vertical or horizontal axis in function of the position of the pulling points, to elicit the release of different molecules from the same 4‐membered ring mechanophore depending on its orientation on the rotaxane axle. As a proof‐of‐concept, we selected diazetidinone^[^ ^19^ ^]^ and β‐lactam^[^ ^20^ ^]^ mechanophores, which have only been reported to cleave along a single axis using covalently attached polymers due to the difficulty of obtaining the corresponding adducts with orthogonal pulling points.^[^ ^19^ ^]^ We show that the pushing activation of these mechanophores leads to the release of a ketene and triaryl imine/ethylene, and the generation of imine, azobenzene, and isocyanate units at the end of the axle. This new activation method enables new reactivities from existing mechanophores and should help the creation of new structures for various applications, in particular for the release of reactive species^[^ ^21^ ^]^ and functional molecules.^[^ ^22^ ^]^

We designed two pairs of 4‐membered rings mechanophores (See Figure 1b) built around diazetidinone (X = N) or β‐lactam (X = CH) cores appended with a bulky *gem*‐diphenyl group (gDP). These mechanophores were incorporated into a rotaxane actuator in a *cis* or *trans* orientation depending on the gDP pointing toward or away from the macrocycle. Considering the ability of a rotaxane to activate a mechanophores by pushing, whereby the forceful contact between the macrocycle and the mechanophore leads to the scission of a covalent bond in the latter,^[^ ^16^ ^]^ we anticipated the possibility of activating orthogonal bonds in the 4‐membered ring depending on the orientation of the gDP (i.e., bonds parallel or perpendicular to the main chain). Due to its large size, the gDP is likely to act as the main pushing point for the macrocycle. In the *cis* isomers, this would result in the gDP being pushed away from the macrocycle via the elongation of the C─X bond in an unzipping geometry. In contrast, the *trans* isomers are expected to dissociate upon elongation of the bonds parallel to the main axis in a shearing geometry, due to both the gDP and methoxyphenyl groups being pushed away (Figure 1b).

**Figure 1:**
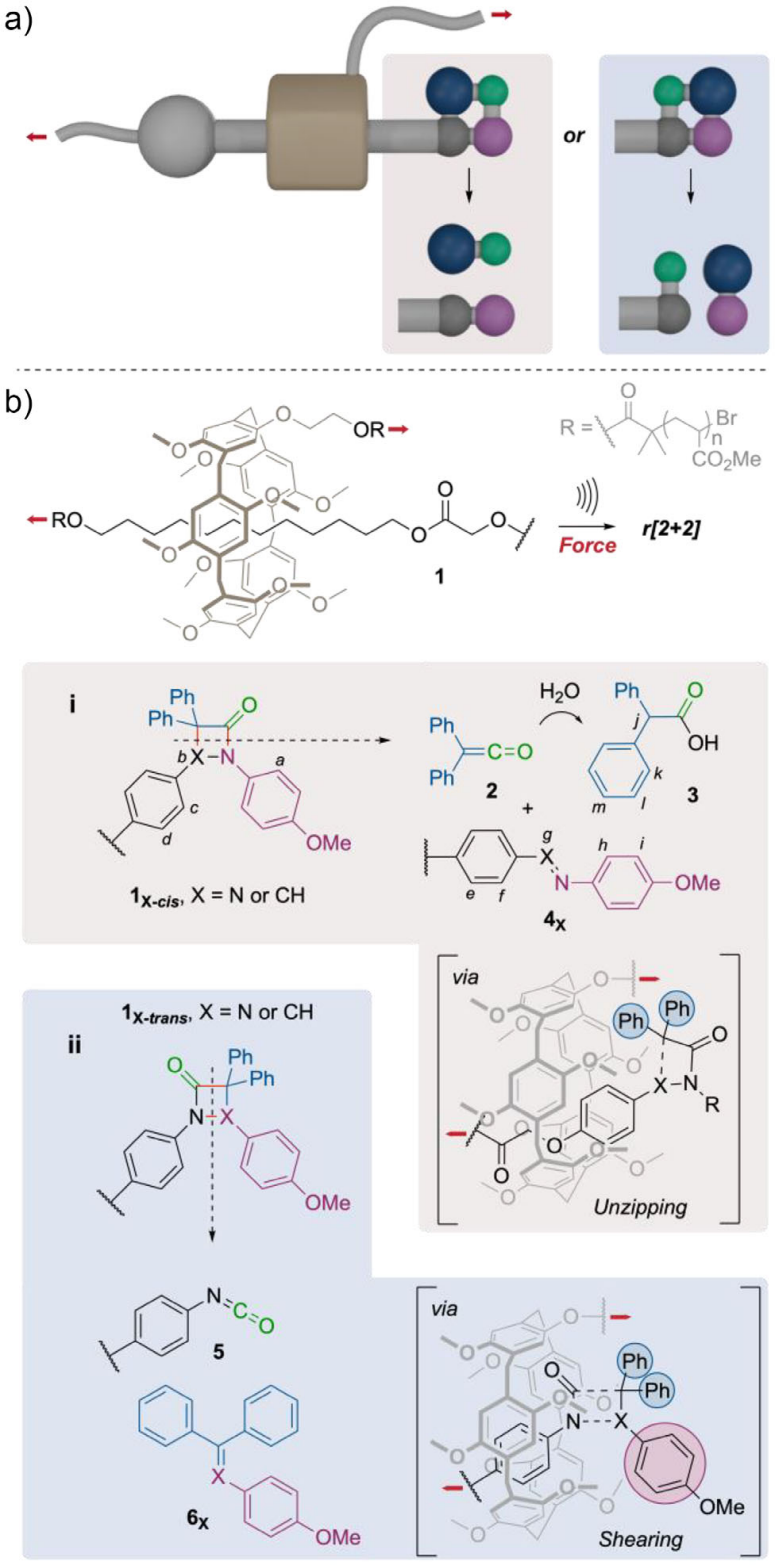
a) Models of mechanical orthogonal bond scission of a 4‐membered ring upon *pushing* force from a rotaxane actuator. Red arrows indicate the direction of the force. b) *Pushing* actuation leads to retro‐[2 + 2] cycloaddition of diazetidinone or β‐lactam cores, in which scissile bonds are shown in red. The pathway of bond cleavage via unzipping (i) or shearing (ii) was affected by a bulky *gem*‐diphenyl group pointing toward (i) or away from (ii) the macrocycle, releasing ketene or triaryl imine/ethene respectively and the remaining polymer. Conditions: US (20 kHz, 13.0 W cm^−2^, 1s ON/1s OFF), THF/H_2_O: 9/1, 5–10 °C, 90 min.

Chain‐centered macromolecular rotaxanes are built around a pillar[5]arene (P5) macrocycle threaded onto a C12 alkyl chain stoppered on one side with the 4‐membered ring mechanophore and on the other side with a poly(methyl acrylate) chain (PMA). These devices can be easily assembled by reacting rotaxane **10** with acid **8** or **9**,^[^ ^23^ ^]^ obtained in two steps from **7**, to afford rotaxanes **11_N‐_ *_cis_*** and **11_N‐_ *_trans_***, which are further converted to the corresponding chain‐centered macromolecular rotaxane **1_N_** by single electron transfer living radical polymerization (SET‐LRP)^[^ ^24^ ^]^ of methyl acrylate (Scheme 1). Rotaxanes **1_C‐*cis/trans*_** were synthesized following a similar strategy (see Supporting Information).

**Scheme 1:**
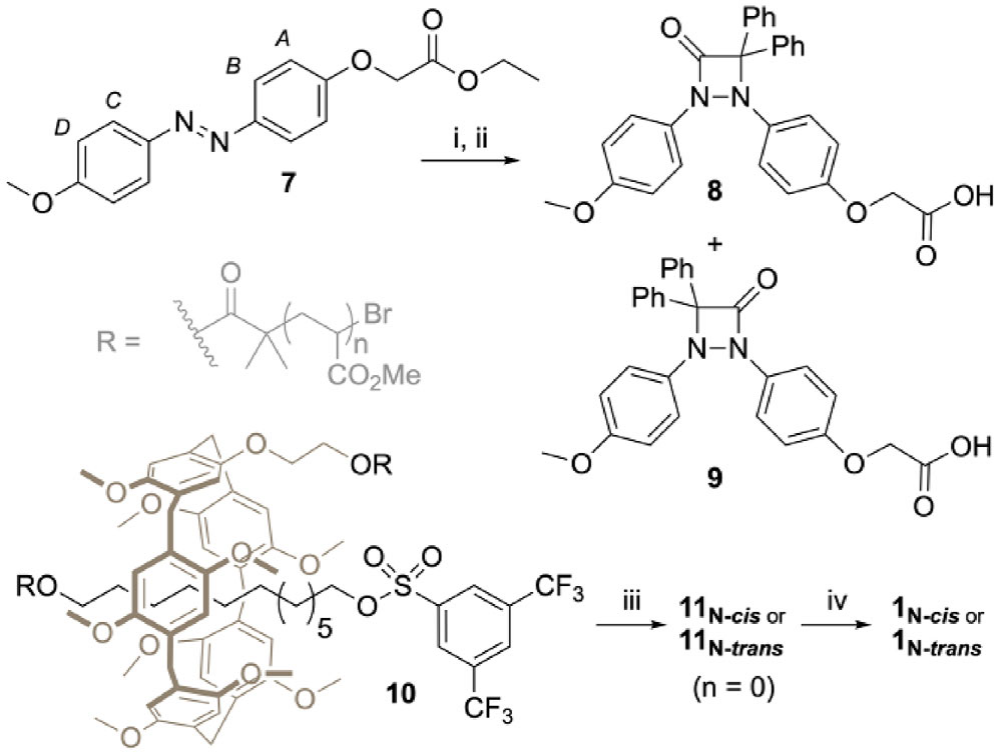
Synthesis of macromolecular rotaxane **1_N_**.^a)^a) Conditions: (i) Diphenylacetyl chloride, Et_3_N, DCM, UV 365 nm, r.t., 16 h, yield: 72% for isomers mixture; (ii) LiOH, THF/MeOH/H_2_O (2/2/1), r.t., 1 h, yield: 42% for **8** and 51% for **9**; (iii) **8** or **9**, K_2_CO_3_, 18‐crown‐6, Acetone, r.t., 16 h, yield: 20%. (iv) Methyl acrylate, CuBr_2_/Cu(0), Me_6_TREN, DMSO, r.t.

The mechanical activation of two sets of two isomers of rotaxane **1** was performed in THF/H_2_O: 9/1 at 5–10 °C, using high‐intensity ultrasound (20 kHz, 13.0 W/cm^2^, 1 s ON/1s OFF, 90 min). ^1^H NMR analysis of the sonication of mechanophore polymers **1_X‐_ *_cis_*** confirms that pushing activation of the *cis* diazetidinone and β‐lactam mechanophores leads to the release of diphenyl ketene **2**, extracted as the corresponding carboxylic acid **3** (*j‐m*, Figure 2b(iii)) formed upon in situ hydrolysis, while leaving *trans*‐azobenzene (*e‐f, h‐i*, Figure 2a(ii)) and imine (*e′‐i′*, Figure 2a(v‐vi)) groups attached to the axle respectively (Figures 1b and 2a). These products confirm that the activation of these *cis* isomers proceeds via the scission of the bonds perpendicular to the main chain. In contrast, the activation of the *trans* isomers **1_X‐_ *_trans_*** results in the release of triphenylethylene **6_C_** or triphenylmethanimine derivative **6_N_** and leaving a terminal isocyanate on the axle (Figure 1b and Supporting Information Section 5). These products arise from the scission of the bonds parallel to the main chain in the *trans* isomers of the 4‐membered ring mechanophores. SEC analysis of the *M*_n_ before and after sonication confirms the complete scission of the initial polymers and, to some extent, of the daughter chains from the first scission (Table 1). The activation efficiency of each mechanophore was determined by comparing diagnostic signals of the mechanophore and its products by ^1^H NMR (Table 1 and Supporting Information Section 6). The activation efficiency also reflects the selectivity of activation as the unreacted mechanophores are located in chains that have been cleaved elsewhere (most likely in the PMA). The *cis* isomers are on average more reactive than their *trans* counterparts (Table 1), which is in accordance with their respective hypothesized unzipping and shearing geometries of activation (Figure 1b). This trend is reflected in the *F_max_* values (Table 1) obtained from CoGEF (Constrained Geometries Simulate External Force) calculations (see below).^[^ ^25^ ^]^

**Figure 2:**
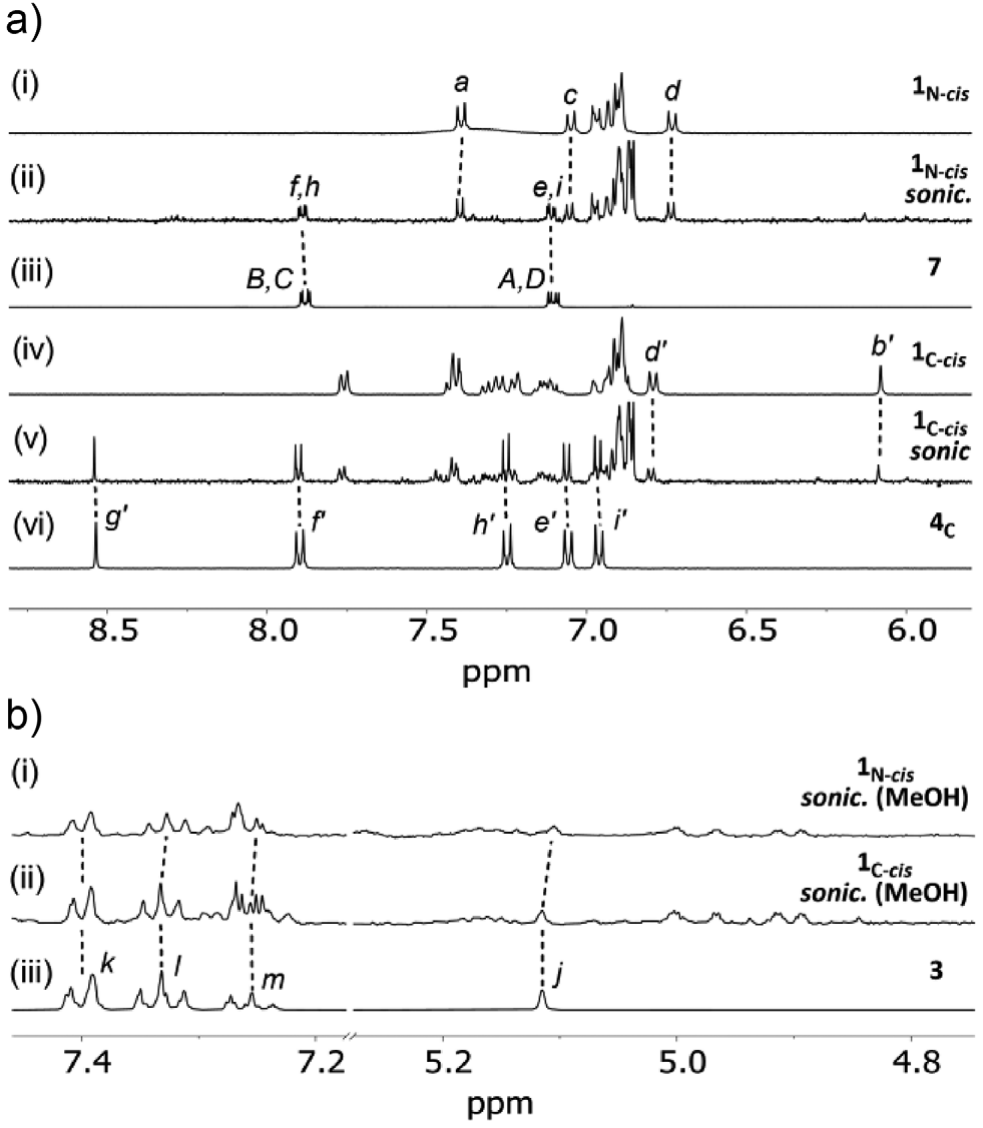
Partial ^1^H NMR (400 MHz, acetone‐*d*_6_, 298 K) spectra of: a) mechanophore **1_N‐*cis*_** before (i) and after (ii) sonication and MeOH extraction, along with reference compound **7** (iii), and mechanophore **1_C‐cis_** before (iv) and after (v) sonication and MeOH extraction, along with reference polymer **4_C_** (vi), b) post‐sonication MeOH extract of mechanophore **1_N‐*cis*_** (i), and **1_C‐*cis*_** (ii), along with reference compound **3** (iii). Assignments correspond to the lettering shown in Figure 1 and Scheme 1 (letters in **X_C_** marked with prime).

**Table 1:** Structural and activation parameters.

1_X_	*Pre‐sonic*. *M_n_ (kDa)/Đ*	*Post‐sonic. M_n_* ^a)^ *(kDa)/Đ*	*Mechanophore* *activation (%)* ^a^ ^),^ ^b)^	*F_max_ (nN)* ^c)^
**N‐*cis***	149/1.42	37/1.31	35	3.6
**C‐*cis***	138/1.18	41/1.25	69	3.4
**N‐*trans***	131/1.39	40/1.32	43	4.9
**C‐*trans***	147/1.17	39/1.31	23	5.4

^a)^ Average value from two parallel experiments. See Table S2 for details.

^b)^ Mechanophore activation is determined from the integration ratio of diagnostic peaks of the mechanophore and its products in the post‐sonication ^1^H NMR spectra. See Supporting Information Section 6 for calculation details.

^c)^ From CoGEF calculations. See Supporting Information Section 7 for details.

The simulated elongation (CoGEF, DFT B3LYP/6–31G, vac) of models of the diazetidinone (**1_N_** *′*) and β‐lactam (**1_C_** *′*) rotaxanes offers some insight into the difference in bond scission observed in the *cis* and *trans* isomers of these 4‐membered ring mechanophores (Figure 3). In the *cis* isomers (**1_X‐_ *_cis_*** *′*) the rotaxane actuation provokes the elongation of bond *b* connecting X to the gDP unit (Figure 3b). This is accompanied by a moderate elongation of bond *b′* on the other side of the ring, along with a contraction of bond *a′* connecting gDP to the carbonyl (which eventually converts into a double bond in the ketene product). Taken together, these deformations suggest that the macrocycle is pushing the gDP unit up and away upon stretching of the rotaxane (Figure 1b(i)), a phenomenon that can be visualized from the structures at *E_max_* (i.e., just before breaking, Figure 3c). As the gDP group is placed on the opposite side of the mechanophore in the *trans* isomers (**1_X‐_ *_trans_*** *′*), the 4‐membered ring can penetrate deeper into the cavity of the macrocycle (see for example the position of the carbonyl in Figure 3d). As a result, the macrocycle is pushing against both the gDP and the methoxyphenyl unit, which results in the preferential elongation of N─N bond *a* in **1_N‐_ *_trans_*** *′*, and of both *a* and *a′* in **1_C‐_ *_trans_*** *′* (Figure 3b). The greater compliance of the N─N bond over the N─C bond presumably originates in the lower bond dissociation energy of the N─N bond.^[^ ^26^ ^]^ Despite a substantial elongation of bond *a*, **1_C‐_ *_trans_*** *′* is predicted to cleave at bond *c* which connects the mechanophore to the axle of the rotaxane, an unstoppering process we have described in other systems.^[^ ^13^ ^]^ This contrasts with the experimental results (Table 1), and highlights the limitations of a static method, such as CoGEF,^[^ ^27^ ^]^ in predicting the scission of similarly loaded bonds^[^ ^28^ ^]^ as it doesn't account for thermal and dynamic effects.^[^ ^29^, ^30^, ^31^, ^32^, ^33^ ^]^

**Figure 3:**
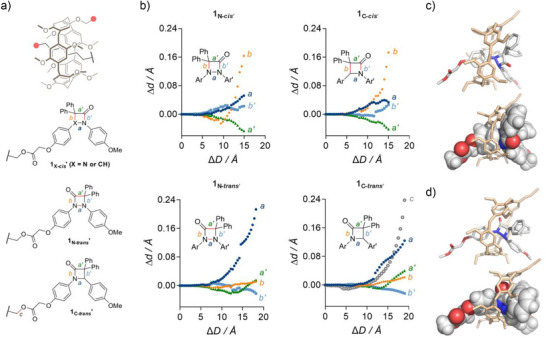
Computational investigation of orthogonal bond scission of diazetidinone or β‐lactam cores by *pushing* actuations (CoGEF, DFT B3LYP/6–31G, vac). a) Models used in the computation indicating key structural parameters. Predicted scissile bonds are shown in red. Anchor atoms are indicated by the pink disks. Evolution of bonds *a*, *a′*, *b*, *b′*, and *c* b) in mechanophores **1_N‐_ *_cis_*** *′*, **1_C‐_ *_cis_*** *′*, **1_N‐_ *_trans_*** *′*, and **1_C‐_ *_trans_*** *′* upon simulated elongation of the rotaxanes. Side views of the model (stick or space‐filling for axle part and stick for macrocycle) of mechanophores **1_N‐_ *_cis_*** *′* c) and **1_N‐_ *_trans_*** *′* d) at maximal deformation (*E_max_* from CoGEF, see Supporting Information) reveals the deformation of the 4‐membered rings and extent of the contact surface between the mechanophore and the macrocycle. Hydrogen atoms in the stick representation and partial CH_2_‐units chain in the axle omitted for clarity.

In conclusion, we elicited the scission of orthogonal bonds in diazetidinone and β‐lactam mechanophores using a rotaxane actuator. The pushing activation of the rotaxane enables the actuation of bonds perpendicular or parallel to the main chain depending on the orientation of bulky groups of the 4‐membered rings pointing toward or away from the incoming macrocycle respectively. In effect, this strategy provides two different mechanophores from the same core structure. Remarkably, even simple structures, such as the diazetidinone and β‐lactam investigated here, can lead to the formation of a variety of useful molecules and imbue the polymer with new properties. Depending on the scission axis we can release either a ketene, which is quickly converted to a carboxylic acid in the presence of water (an example of mechanoacid),^[^ ^34^, ^35^, ^36^ ^]^ or triaryl imine/ethylene derivatives. Triphenylethylene derivatives are potent nonsteroidal oestrogen receptor ligands, some of which are used to treat breast cancer (e.g., tamoxifen),^[^ ^37^ ^]^ and are also used as AIE compounds,^[^ ^38^ ^]^ while triarylimines are useful synthetic precursors of amino acids^[^ ^39^ ^]^ and acenes.^[^ ^40^ ^]^ It is worth noting that these molecules can only be released with the help of a rotaxane actuator. The vertical activation of the β‐lactam leaves an isocyanate on the axle, which can be used for further functionalisation of the chain or to reveal an amine upon decarboxylation in the presence of water. The horizontal activation of the diazetidinone and β‐lactam cores, reveals azobenzene and imine units respectively. The former is a dye and a photoswitch,^[^ ^41^ ^]^ while the latter can be hydrolysed to release the connected amine. Hence, providing the polymer with photochromic properties or further release abilities upon mechanical activation. Overall, this form of orthogonal activation provides a way to rapidly expand the diversity of molecules generated from known or upcoming mechanophores and offers new opportunities for the release of reactive species and other functional molecules.

## Conflict of Interests

The authors declare no conflict of interest.

## Supporting information



Supporting Information

## Data Availability

The data that support the findings of this study are available from the corresponding author upon reasonable request.
