# Supplementary material for: Selective Scission of Orthogonal Bonds in Four‐Membered Ring Mechanophores upon Activation by a Rotaxane Actuator
Source: Angew Chem Int Ed Engl. 2025 Jun 18;64(37):e202511039. doi: 10.1002/anie.202511039 (PMC12416471; doi:10.1002/anie.202511039)
Supplement: Supplementary file 1 — Supporting Information [file ANIE-64-e202511039-s001.pdf]

Supporting Information

Selective Scission of Orthogonal Bonds in Four-Membered Ring Mechanophores upon Activation by a Rotaxane Actuator

*Lei Chen and Guillaume De Bo**

Department of Chemistry, University of Manchester, Oxford Road, Manchester, M13 9PL, UK

*E-mail: guillaume.debo@manchester.ac.uk

1 Table of Contents

1 Table of Contents ... 2
2 General Experimental Details ... 5
3 Synthesis of Mechanophore, Rotaxane, Control, and Reference Compounds ... 6
3.1 Synthesis of Mechanophore Compounds ... 6
3.1.1 Synthetic Routes to 8 and 9 ... 6
3.1.2 Synthesis of S1 ... 6
3.1.3 Synthesis of 8 and 9 ... 7
3.1.4 Synthetic Routes to S5 ... 7
3.1.5 Synthesis of S3 ... 8
3.1.6 Synthesis of S4 ... 8
3.1.7 Synthesis of S5 ... 9
3.1.8 Synthetic Routes to S9 ... 9
3.1.9 Synthesis of S7 ... 9
3.1.10 Synthesis of S8 ... 10
3.1.11 Synthesis of S9 ... 10
3.2 Synthesis of Rotaxane and Control Compounds ... 11
3.2.1 Synthesis of $11_{N\text{-}cis}$ ... 11
3.2.2 Synthesis of $11_{N\text{-}trans}$ ... 12
3.2.3 Synthesis of $11_{C\text{-}cis}$ ... 12
3.2.4 Synthesis of $11_{C\text{-}trans}$ ... 13
3.2.5 Synthesis of S11 ... 14
3.2.6 Synthesis of S12 ... 14
3.2.7 Synthesis of S13 ... 15
3.2.8 Synthesis of S14 ... 15
3.3 Synthesis of Reference Compounds ... 16
3.3.1 Synthetic Routes to S17 ... 16
3.3.2 Synthesis of S16 ... 16
3.3.3 Synthesis of S17 ... 17
4 Synthesis of Polymers ... 18
4.1 Representative Procedure for SET-LRP of Methyl Acrylate Using Mechanophore Initiators ... 18
4.2 Synthesis of Mechanophore and Control Polymers ... 19
4.2.1 Synthesis of Polymer $1_{N\text{-}cis}$ ... 19
4.2.2 Synthesis of Polymer $1_{N\text{-}trans}$ ... 19
4.2.3 Synthesis of Polymer $1_{C\text{-}cis}$ ... 20
4.2.4 Synthesis of Polymer $1_{C\text{-}trans}$ ... 20
4.2.5 Synthesis of Polymer S18 ... 21
4.2.6 Synthesis of Polymer S19 ... 21
4.2.7 Synthesis of Polymer S20 ... 22
4.2.8 Synthesis of Polymer S21 ... 22
4.3 Synthesis of Reference Polymers ... 23
4.3.1 Synthesis of Polymer S22 ... 23
4.3.2 Synthesis of Polymer 4_C ... 23
4.4 SEC Data for Synthesised Polymers ... 24
4.5 SEC Traces for Mechanophore and Control Polymers ... 25
4.6 SEC Traces for Reference Polymers ... 26
5 Mechanophore Activation via Ultrasound ... 27
5.1 General Procedure for Sonication Experiments ... 27
5.2 Sonication of Mechanophore Polymer $1_{N\text{-}cis}$ ... 27
5.3 Sonication of Mechanophore Polymer $1_{N\text{-}trans}$ ... 30
5.4 Sonication of Mechanophore Polymer $1_{C\text{-}cis}$ ... 33
5.5 Sonication of Mechanophore Polymer $1_{C\text{-}trans}$ ... 36

5.6 Sonication of Control Polymers S18, S19, S20 and S21 39
5.7 Determination of the Degradation Product of S26 in the Sonication Experiment 44
5.8 Analysis of Contaminants in Post-Sonication MeOH Extracts 47
6 Calculation of Extent of Mechanophore Activation 48
6.1 Calculations for Polymer $1_{N\text{-}cis}$ 48
6.2 Calculations for Polymer $1_{N\text{-}trans}$ 49
6.3 Calculations for Polymer $1_{C\text{-}cis}$ 50
6.4 Calculations for Polymer $1_{C\text{-}trans}$ 51
6.5 Summary of Mechanophores Activated by Sonication 52
7 CoGEF Calculations 53
7.1 General method 53
7.2 CoGEF of models of $1_{N\text{-}cis}$’ 53
7.3 CoGEF of models of $1_{N\text{-}trans}$’ 54
7.4 CoGEF of models of $1_{C\text{-}cis}$’ 55
7.5 CoGEF of models of $1_{C\text{-}trans}$’ 56
8 NMR Spectra 57
8.1 Small Molecule NMR Spectra 57
8.1.1 Spectra of S1 57
8.1.2 Spectra of S3 58
8.1.3 Spectra of S4 59
8.1.4 Spectra of S5 60
8.1.5 Spectra of S7 61
8.1.6 Spectra of S8 62
8.1.7 Spectra of S9 63
8.1.8 Spectra of $11_{N\text{-}cis}$ 64
8.1.9 Spectra of $11_{N\text{-}trans}$ 65
8.1.10 Spectra of $11_{C\text{-}cis}$ 66
8.1.11 Spectra of $11_{C\text{-}trans}$ 67
8.1.12 Spectra of S11 68
8.1.13 Spectra of S12 69
8.1.14 Spectra of S13 70
8.1.15 Spectra of S14 71
8.1.16 Spectra of S16 72
8.1.17 Spectra of S17 73
8.2 Polymer NMR Spectra 74
8.2.1 Spectra of polymer $1_{N\text{-}cis}$ 74
8.2.2 Spectra of polymer $1_{N\text{-}trans}$ 74
8.2.3 Spectra of polymer $1_{C\text{-}cis}$ 75
8.2.4 Spectra of polymer $1_{C\text{-}trans}$ 75
8.2.5 Spectra of polymer S18 76
8.2.6 Spectra of polymer S19 76
8.2.7 Spectra of polymer S20 77
8.2.8 Spectra of polymer S21 77
8.2.9 Spectra of polymer S22 78
8.2.10 Spectra of polymer 4_C 78
8.3 Post-Sonication NMR Spectra 79
8.3.1 Post-Sonication ^{1}H NMR Spectra of Polymer $1_{N\text{-}cis}$ (Run 1) 79
8.3.2 Post-Sonication ^{1}H NMR Spectra of Polymer $1_{N\text{-}cis}$ (Run 2) 81
8.3.3 Post-Sonication ^{1}H NMR Spectra of Polymer $1_{N\text{-}trans}$ (Run 1) 82
8.3.4 Post-Sonication ^{1}H NMR Spectra of Polymer $1_{N\text{-}trans}$ (Run 2) 84
8.3.5 Post-Sonication ^{1}H NMR Spectra of Polymer $1_{C\text{-}cis}$ (Run 1) 85
8.3.6 Post-Sonication ^{1}H NMR Spectra of Polymer $1_{C\text{-}cis}$ (Run 2) 87
8.3.7 Post-Sonication ^{1}H NMR Spectra of Polymer $1_{C\text{-}trans}$ (Run 1) 88
8.3.8 Post-Sonication ^{1}H NMR Spectra of Polymer $1_{C\text{-}trans}$ (Run 2) 90

8.3.9 Post-Sonication ^{1}H NMR Spectra of Polymer S18 91
8.3.10 Post-Sonication ^{1}H NMR Spectra of Polymer S19 93
8.3.11 Post-Sonication ^{1}H NMR Spectra of Polymer S20 94
8.3.12 Post-Sonication ^{1}H NMR Spectra of Polymer S21 96
9 Mass Spectrometry Isotopic Patterns 98
9.1 Isotopic distribution of $11_{N\text{-}cis}$ 98
9.2 Isotopic distribution of $11_{N\text{-}trans}$ 98
9.3 Isotopic distribution of $11_{C\text{-}cis}$ 99
9.4 Isotopic distribution of $11_{C\text{-}trans}$ 99
9.5 Isotopic distribution of S11 100
9.6 Isotopic distribution of S12 100
9.7 Isotopic distribution of S13 101
9.8 Isotopic distribution of S14 101
10 References 102

2 General Experimental Details

Unless otherwise stated, all reagents and solvents were purchased from commercial suppliers and used without further purification. Dry solvents were obtained by passing through an activated alumina column on a Phoenix SDS solvent drying system (JC Meyer Solvent Systems, CA, USA). Compound **7**,[1] **S6**, **S26**,[2] **S2**,[3] **10**, **S10**,[4] **S15**,[5] $\mathbf{6_N}$[6] and $\mathbf{6_C}$[7] were prepared according to literature procedures.

Size exclusion chromatography (SEC) analyses were performed in THF solution (1.0 mg mL^{-1}) at 40 °C using a GPC/SEC Agilent 1260 Infinity II with 2 × PL gel 10 µm mixed-C and a PL gel 500 Å column, and equipped with a differential refractive index (DRI) detector employing narrow polydispersity polystyrene standards (Agilent Technologies) as a calibration reference. Samples were filtered through a Whatman Puradisc 4 mm syringe filter with 0.45 µm PTFE membrane before injection to equipment, and experiments were carried out with injection volume of 50 µL, flow rate of 1 mL min^{-1}. Results were analyzed using *n*-dodecane as internal marker using Agilent GPC/SEC Software Version 2.2.

Ultrasound experiments were performed using a Sonics VCX 500 ultrasonic processor equipped with a 13 mm diameter solid or replaceable-tip probe. The distance between the titanium tip and the bottom of the Suslick cell was 2 cm. The ultrasonic intensity was calibrated using the method outlined by Hickenboth *et al*.[8] The Suslick cells were fabricated by the Department of Chemistry glass workshop at the University of Manchester.

Analytical TLC was performed on precoated silica gel plates (0.25 mm thick, 60 F254, Merck, Germany) and observed under UV light or stained with a potassium permanganate base solution. Preparative TLC was performed on precoated silica gel plates: 500 µm or 2000 µm, UNIPLATE GF, Analtech Inc., DE, USA. Flash column chromatography was performed with silica gel 60 (230-400 mesh) from Sigma-Aldrich. ^{1}H and ^{13}C NMR spectra were recorded on a Bruker Avance III 600 MHz Prodigy instrument, a Bruker Avance III 500 MHz Prodigy instrument or a Bruker Avance III 400 MHz Prodigy instrument. Chemical shifts are reported in parts per million (ppm) from high to low frequency and referenced to the residual solvent resonance. Coupling constants (*J*) are reported in Hertz (Hz) and splitting patterns are designated as follows: b = broad, s = singlet, d = doublet, t = triplet, q = quartet, p = pentet and m = multiplet. ^{1}H and ^{13}C assignments were made using 1D or 2D NMR methods (HSQC, HMBC, COSY). Mass spectra were obtained through the Mass Spectrometry services in the Department of Chemistry at the University of Manchester.

Abbreviations: APCI: atmospheric-pressure chemical ionization; CoGEF: constrained geometries simulate external force; DCM: dichloromethane; DMSO: dimethylsulfoxide; ESI: electrospray ionization; HRMS: high resolution mass spectrometry; MA: methyl acrylate; MS: mass spectrometry; Me_6TREN: tris[2-(dimethylamino)ethyl]amine; PE: petroleum ether; PMDETA: N,N,N′,N′′,N′′-Pentamethyldiethylenetriamine; THF: tetrahydrofuran; TLC: thin layer chromatography.

3 Synthesis of Mechanophore, Rotaxane, Control, and Reference Compounds

3.1 Synthesis of Mechanophore Compounds

3.1.1 Synthetic Routes to 8 and 9

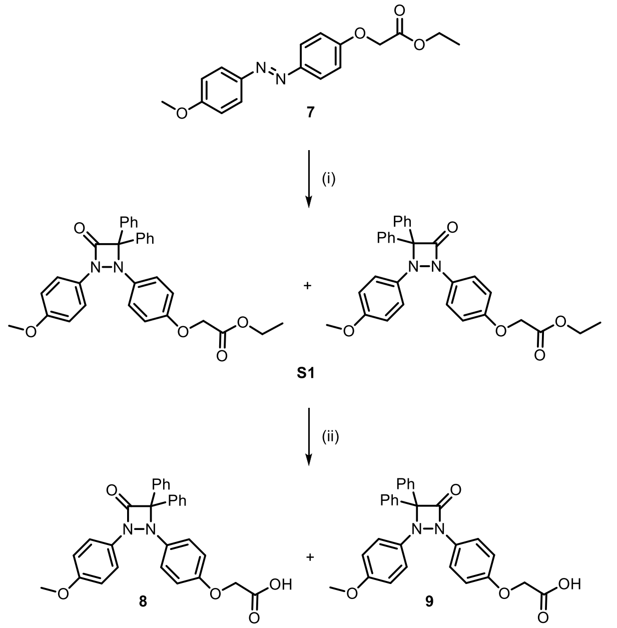

Scheme S1. Synthetic routes to **8** and **9**. Conditions: (i) Diphenylacetyl chloride, Et_3N, DCM, UV 365 nm, r.t., 16 h, 72% yield; (ii) LiOH, THF/MeOH/H_2O (2/2/1), r.t., 1 h, 94% overall yield.

3.1.2 Synthesis of S1

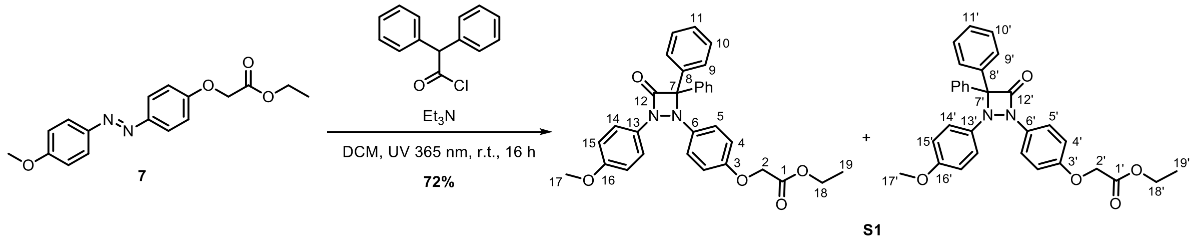

To a solution of **7** (300 mg, 955 µmol, 1.0 eq.) and diphenylacetyl chloride (286 mg, 1242 µmol, 1.3 eq.) in DCM (7 mL) was added Et_3N (145 mg, 1433 µmol, 1.5 eq.). The mixture was then stirred for 16 h under the UV light (365 nm) at room temperature. After the solvent was evaporated, the residue was purified via column chromatography (SiO_2, PE/EtOAc, 10/1) to yield isomer mixture of **S1** as a pale-yellow powder (350 mg, 689 µmol, 72% yield). The mixture was used in the next step (see section 3.1.3) without further purification.

S1 was isolated as a mixture of two isomers as shown in the scheme above. When distinguishable, they are denoted as Xx and Xx′ respectively.

^{1}**H NMR** (400 MHz, Acetone-d_6, 298 K) δ = 7.97 – 7.01 (m, 12H, $H_{9,10.11,14}$), 7.00 – 6.91 (m, 4H, $H_{5,15}$), 6.69 – 6.62 (m, 2H, H_4), 4.69 (s, 1.10H, $H_{2'}$), 4.52 (s, 0.90H, H_2), 4.22 – 4.11 (m, 2H, H_{18}), 3.76 (s, 1.36H,

H_{17}), 3.62 (s, 1.65H, $H_{17'}$), 1.25 – 1.17 (m, 3H, H_{19}).
^{13}C NMR (101 MHz, Acetone-d_6, 298 K) δ = 169.24, 169.18 (C_1), 166.20, 166.18 (C_{12}), 157.85, 157.73 (C_{16}), 156.16, 156.03 (C_3), 139.81, 138.90 (C_6), 139.32, 135.94 (C_8), 132.95, 132.27 (C_{13}), 130.98 – 128.37 ($C_{9,10,11}$), 123.47, 123.29 (C_5), 118.75, 118.57 (C_{14}), 116.06, 115.36, 115.17, 114.50 ($C_{4,15}$), 94.64, 94.63 (C_7), 66.03 ($C_{2'}$), 65.98 (C_2), 61.45, 61.36 (C_{18}), 55.76 (C_{17}), 55.51 ($C_{17'}$), 14.44 (C_{19}).
HRMS-ESI(+): 531.1881 $[M+Na]^+$, calculated for $C_{31}H_{28}N_2O_5Na^+$: 531.1890.

3.1.3 Synthesis of 8 and 9

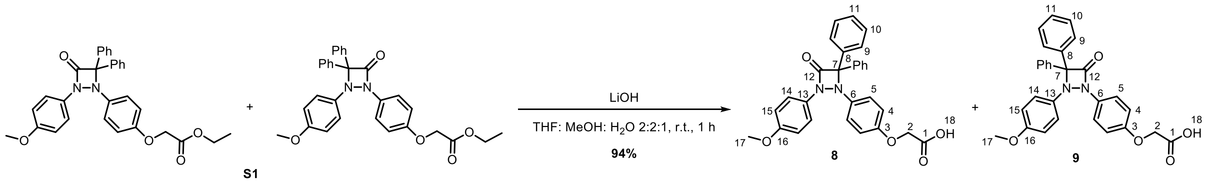

To a solution of **S1** (100 mg, 197 µmol, 1.0 eq.) in a mixture of THF (2 mL), MeOH (2 mL) and water (1 mL) was added LiOH·H_2O (165 mg, 3935 µmol, 20.0 eq.). The mixture was stirred at room temperature for 1 h. After evaporating the organic solvent in the mixture by rotary evaporator, pH of this solution was adjusted to ~2 by addition of aqueous 1 M HCl. Then the mixture was filtered to give yellow solid crude product. After drying under vacuum, this crude was purified via column chromatography (SiO_2, DCM/AcOH, 150/1) to yield **8** (40 mg, 83 µmol, 42% yield) and **9** (48 mg, 100 µmol, 51% yield) as pale-yellow solid. These products were used immediately after column to avoid degredation (see section 3.2.1, 0, 3.2.5 and 3.2.6).

3.1.4 Synthetic Routes to S5

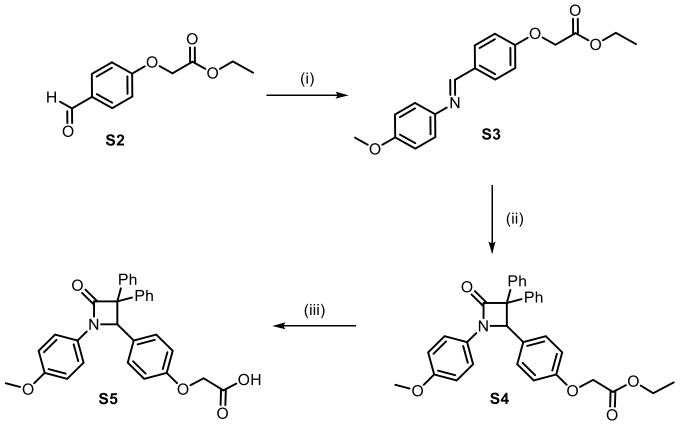

Scheme S2. Synthetic routes to **S5**. Conditions: (i) p-Anisidine, AcOH, EtOH, 80 °C, 2 h, 66% yield; (ii) Diphenylacetyl chloride, Et_3N, DCM, 45 °C, 16 h, 54% yield; (iii) LiOH, THF/MeOH/H_2O (2/2/1), r.t., 1 h, 97% yield.

3.1.5 Synthesis of S3

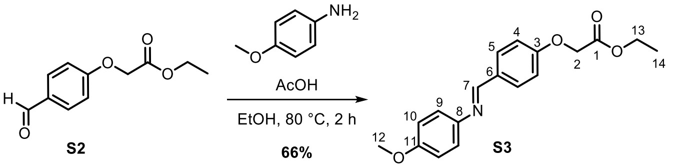

To a solution of **S2** (400 mg, 1922 µmol, 1.0 eq.) and p-anisidine (260 mg, 2115 µmol, 1.1 eq.) in EtOH (7 mL) was added 2 drops of AcOH. The mixture was heated to 80 °C for 2 h. The solution was cooled to room temperature and the mixture was filtered to give solid product. The solid was washed with EtOH and dried under vacuum to yield **S3** as a white powder (395 mg, 1261 µmol, 66% yield).

^{1}H NMR (400 MHz, Acetone-*d*$_6$, 298 K) δ = 8.53 (s, 1H, H_7), 7.92 – 7.85 (m, 2H, H_5), 7.28 – 7.20 (m, 2H, H_9), 7.09 – 7.02 (m, 2H, H_4), 6.99 – 6.92 (m, 2H, H_{10}), 4.82 (s, 2H, H_2), 4.23 (q, *J* = 7.1 Hz, 2H, H_{13}), 3.81 (s, 3H, H_{12}), 1.26 (t, *J* = 7.1 Hz, 3H, H_{14}).
^{13}C NMR (101 MHz, Acetone-*d*$_6$, 298 K) δ = 169.10 (C_1), 161.34 (C_3), 159.13 (C_{11}), 157.99 (C_7), 145.98 (C_8), 131.43 (C_6), 130.93 (C_5), 122.98 (C_9), 115.61 (C_4), 115.15 (C_{10}), 65.77 (C_2), 61.58 (C_{13}), 55.73 (C_{12}), 14.46 (C_{14}).
HRMS-APCI(+): 314.1377 $[M+H]^+$, calculated for $C_{18}H_{19}NO_4H^+$: 314.1387.

3.1.6 Synthesis of S4

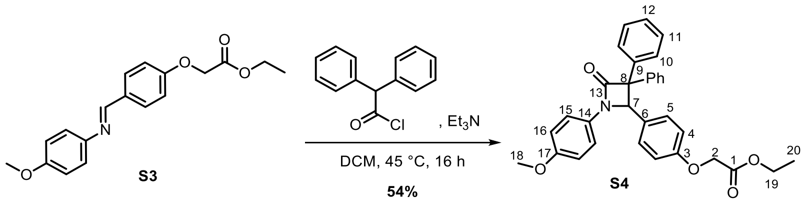

To a solution of **S3** (100 mg, 319 µmol, 1.0 eq.) and diphenylacetyl chloride (88 mg, 383 µmol, 1.2 eq.) in DCM (2 mL) was added Et_3N (116 mg, 1150 µmol, 3.6 eq.). The mixture was then stirred at 45 °C for 16 h. The solution was cooled down to room temperature. After the solvent was evaporated, the residue was purified via by preparative TLC (2000 µm, PE/DCM/EtOAc, 6/3/0.3, eluted three times) to yield **S4** as a white powder (87 mg, 172 µmol, 54% yield).

^{1}H NMR (400 MHz, Acetone-*d*$_6$, 298 K) δ = 7.78 – 7.70 (m, 2H, H_{10}), 7.44 – 7.34 (m, 4H, $H_{11,15}$), 7.33 – 7.26 (m, 1H, H_{12}), 7.22 – 7.14 (m, 4H, $H_{5,10'}$), 7.11 – 7.00 (m, 3H, $H_{11',12'}$), 6.88 – 6.82 (m, 2H, H_{16}), 6.77 – 6.69 (m, 2H, H_4), 6.02 (s, 1H, H_7), 4.60 (s, 2H, H_2), 4.17 (q, *J* = 7.1 Hz, 2H, H_{19}), 3.72 (s, 3H, H_{18}), 1.21 (t, *J* = 7.1 Hz, 3H, H_{20}).
^{13}C NMR (101 MHz, Acetone-*d*$_6$, 298 K) δ = 169.12 (C_1), 166.94 (C_{13}), 158.83 (C_3), 157.16 (C_{17}), 142.35 (C_9), 139.10 ($C_{9'}$), 131.81 (C_{14}), 130.02 (C_5), 129.45 (C_{11}), 129.17 ($C_{10'}$), 128.94 (C_6), 128.70 ($C_{11'}$), 128.30 (C_{10}), 128.10 (C_{12}), 127.46 ($C_{12'}$), 119.67 (C_{15}), 115.19 (C_4), 115.05 (C_{16}), 72.88 (C_8), 66.85 (C_7), 65.73 (C_2), 61.42 (C_{19}), 55.66 (C_{18}), 14.45 (C_{20}).
HRMS-APCI(+): 508.2110 $[M+H]^+$, calculated for $C_{32}H_{29}NO_5H^+$: 508.2118.

3.1.7 Synthesis of S5

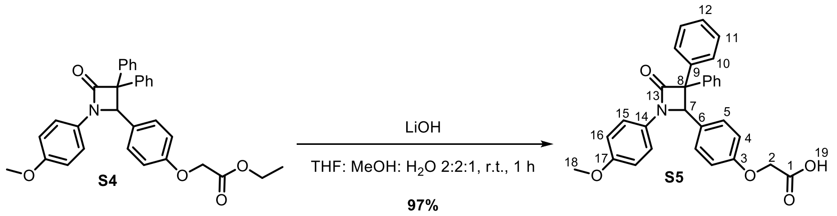

To a solution of **S4** (60 mg, 118 µmol, 1.0 eq.) in a mixture of THF (1 mL), MeOH (1 mL) and water (0.5 mL) was added LiOH·H_2O (99 mg, 2366 µmol, 20.0 eq.). The mixture was stirred at room temperature for 1 h. After evaporating the organic solvent in the mixture by rotary evaporator, pH of this solution was adjusted to ~2 by addition of aqueous 1 M HCl. Then the mixture was filtered to yield **S5** as a white powder (55 mg, 115 µmol, 97% yield).

^{1}H NMR (400 MHz, Acetone-*d*$_6$, 298 K) δ = 7.78 – 7.70 (m, 2H, H_{10}), 7.44 – 7.35 (m, 4H, $H_{11,15}$), 7.33 – 7.25 (m, 1H, H_{12}), 7.22 – 7.13 (m, 4H, $H_{5,10'}$), 7.11 – 6.98 (m, 3H, $H_{11',12'}$), 6.88 – 6.82 (m, 2H, H_{16}), 6.79 – 6.70 (m, 2H, H_4), 6.02 (s, 1H, H_7), 4.60 (s, 2H, H_2), 3.72 (s, 3H, H_{18}).
^{13}C NMR (101 MHz, Acetone-*d*$_6$, 298 K) δ = 169.92 (C_1), 166.96 (C_{13}), 158.85 (C_3), 157.17 (C_{17}), 142.37 (C_9), 139.10 ($C_{9'}$), 131.81 (C_{14}), 130.02 (C_5), 129.45 (C_{11}), 129.17 ($C_{10'}$), 128.88 (C_6), 128.70 ($C_{11'}$), 128.29 (C_{10}), 128.10 (C_{12}), 127.47 ($C_{12'}$), 119.69 (C_{15}), 115.18 (C_4), 115.06 (C_{16}), 72.87 (C_8), 66.88 (C_7), 65.34 (C_2), 55.65 (C_{18}).
HRMS-APCI(-): 478.1659 $[M-H]^-$, calculated for $C_{30}H_{24}NO_5^-$: 478.1660.

3.1.8 Synthetic Routes to S9

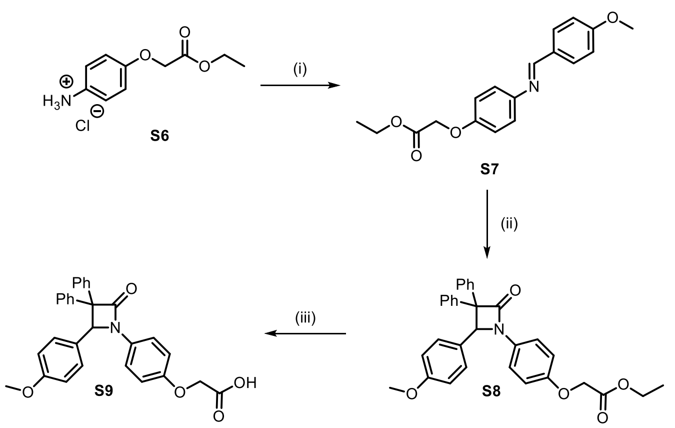

Scheme S3. Synthetic routes to **S9**. Conditions: (i) p-Anisaldehyde, $AlCl_3$, Et_3N, DCM, r.t., 5 h, 15% yield; (ii) Diphenylacetyl chloride, Et_3N, DCM, 45 °C, 16 h, 93% yield; (iii) LiOH, THF/MeOH/H_2O (2/2/1), r.t., 1 h, 71% yield.

3.1.9 Synthesis of S7

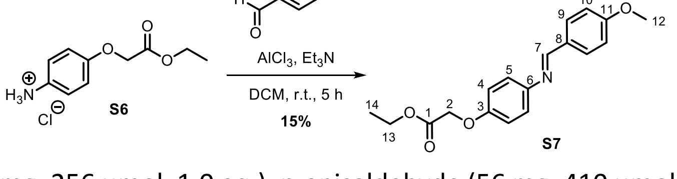

To a solution of **S6** (50 mg, 256 µmol, 1.0 eq.), p-anisaldehyde (56 mg, 410 µmol, 1.6 eq.) and $AlCl_3$ (68 mg, 513 µmol, 2.0 eq.) in DCM (2 mL) was added Et_3N (130 mg, 1281 µmol, 5.0 eq.). The mixture was

then stirred at room temperature for 5 h. After the solvent was evaporated, the residue was purified via column chromatography (SiO_2, PE/EtOAc, 10/1) to yield **S7** as a white powder (12 mg, 38 µmol, 15% yield).

^{1}H NMR (400 MHz, Acetone-d_6, 298 K) δ = 8.52 (s, 1H, H_7), 7.92 – 7.87 (m, 2H, H_9), 7.27 – 7.20 (m, 2H, H_5), 7.08 – 7.01 (m, 2H, H_{10}), 7.00 – 6.94 (m, 2H, H_4), 4.73 (s, 2H, H_2), 4.22 (q, J = 7.1 Hz, 2H, H_{13}), 3.88 (s, 3H, H_{12}), 1.26 (t, J = 7.1 Hz, 3H, H_{14}).
^{13}C NMR (101 MHz, Acetone-d_6, 298 K) δ = 169.43 (C_1), 163.11 (C_{11}), 158.68 (C_7), 157.40 (C_3), 146.86 (C_6), 131.07 (C_9), 130.65 (C_8), 122.90 (C_5), 116.00 (C_4), 114.96 (C_{10}), 66.08 (C_2), 61.45 (C_{13}), 55.80 (C_{12}), 14.47 (C_{14}).
HRMS-APCI(+): 314.1376 $[M+H]^+$, calculated for $C_{18}H_{19}NO_4H^+$: 314.1387.

3.1.10 Synthesis of S8

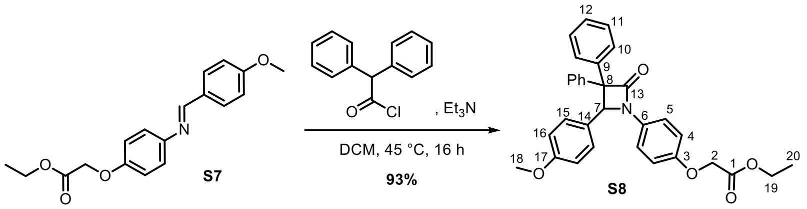

To a solution of **S7** (10 mg, 32 µmol, 1.0 eq.) and diphenylacetyl chloride (9 mg, 38 µmol, 1.2 eq.) in DCM (2 mL) was added Et_3N (12 mg, 115 µmol, 3.6 eq.). The mixture was then stirred at 45 °C for 16 h. The solution was cooled to room temperature. The solvent was evaporated and the residue purified via by preparative TLC (500 µm, PE/EtOAc, 5/1, eluted three times) to yield **S8** as a white powder (15 mg, 30 µmol, 93% yield).

^{1}H NMR (400 MHz, Acetone-d_6, 298 K) δ = 7.77 – 7.70 (m, 2H, H_{10}), 7.44 – 7.34 (m, 4H, $H_{11,5}$), 7.32 – 7.26 (m, 1H, H_{12}), 7.22 – 7.13 (m, 4H, $H_{15,10'}$), 7.11 – 7.00 (m, 3H, $H_{11',12'}$), 6.92 – 6.84 (m, 2H, H_4), 6.75 – 6.69 (m, 2H, H_{16}), 6.01 (s, 1H, H_7), 4.65 (s, 2H, H_2), 4.17 (q, J = 7.1 Hz, 2H, H_{19}), 3.69 (s, 3H, H_{18}), 1.21 (t, J = 7.1 Hz, 3H, H_{20}).
^{13}C NMR (101 MHz, Acetone-d_6, 298 K) δ = 169.30 (C_1), 167.10 (C_{13}), 160.45 (C_{17}), 155.53 (C_3), 142.40 (C_9), 139.16 ($C_{9'}$), 132.56 (C_6), 130.03 (C_{15}), 129.46 (C_{11}), 129.20 ($C_{10'}$), 128.70 ($C_{11'}$), 128.30 (C_{10}), 128.11 (C_{12}), 127.95 (C_{14}), 127.46 ($C_{12'}$), 119.60 (C_5), 115.95 (C_4), 114.49 (C_{16}), 72.91 (C_8), 67.01 (C_7), 65.98 (C_2), 61.42 (C_{19}), 55.43 (C_{18}), 14.43 (C_{20}).
HRMS-APCI(+): 508.2106 $[M+H]^+$, calculated for $C_{32}H_{29}NO_5H^+$: 508.2118.

3.1.11 Synthesis of S9

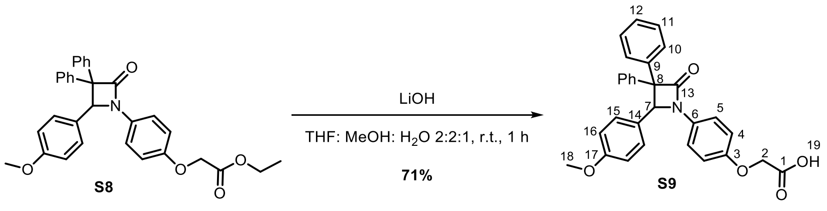

To a solution of **S8** (15 mg, 30 µmol, 1.0 eq.) in a mixture of THF (1 mL), MeOH (1 mL) and water (0.5 mL) was added $LiOH{\cdot}H_2O$ (25 mg, 591 µmol, 20.0 eq.). The mixture was stirred at room temperature for 1 h. After evaporating the organic solvent in the mixture by rotary evaporator, the pH of this solution was adjusted to ~2 by addition of aqueous 1 M HCl. Then the mixture was filtered to yield **S9**

as a white powder (10 mg, 21 µmol, 71% yield).

^{1}H NMR (400 MHz, Acetone-d_6, 298 K) δ = 7.76 – 7.71 (m, 2H, H_{10}), 7.43 – 7.35 (m, 4H, $H_{11,5}$), 7.32 – 7.26 (m, 1H, H_{12}), 7.20 – 7.14 (m, 4H, $H_{15,10'}$), 7.10 – 7.00 (m, 3H, $H_{11',12'}$), 6.92 – 6.86 (m, 2H, H_4), 6.75 – 6.68 (m, 2H, H_{16}), 6.01 (s, 1H, H_7), 4.65 (s, 2H, H_2), 3.69 (s, 3H, H_{18}).
^{13}C NMR (101 MHz, Acetone-d_6, 298 K) δ = 170.12 (C_1), 167.08 (C_{13}), 160.45 (C_{17}), 155.55 (C_3), 142.41 (C_9), 139.17 ($C_{9'}$), 132.49 (C_6), 130.03 (C_{15}), 129.46 (C_{11}), 129.21 ($C_{10'}$), 128.70 ($C_{11'}$), 128.30 (C_{10}), 128.10 (C_{12}), 127.96 (C_{14}), 127.45 ($C_{12'}$), 119.61 (C_5), 115.90 (C_4), 114.48 (C_{16}), 72.90 (C_8), 67.01 (C_7), 65.58 (C_2), 55.43 (C_{18}).
HRMS-APCI(-): 478.1659 $[M-H]^-$, calculated for $C_{30}H_{24}NO_5^-$: 478.1660.

3.2 Synthesis of Rotaxane and Control Compounds

3.2.1 Synthesis of $\mathbf{11_{N\text{-}\mathit{cis}}}$

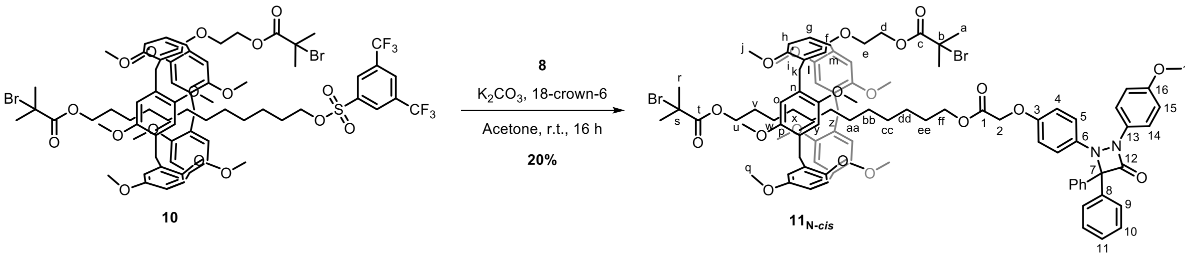

To a solution of **8** (2.8 mg, 6 µmol, 1.0 eq.) in acetone (1 mL) was added K_2CO_3 (0.8 mg, 6 µmol, 1.0 eq.) and 18-crown-6 (1.5 mg, 6 µmol, 1.0 eq.). The mixture was stirred for 2 h at room temperature. **10** (9 mg, 6 µmol, 1.0 eq.) was added and the mixture stirred for a further 16 h at room temperature. The solution was filtered, and the filtrate concentrated under vacuum. The residue was purified by preparative TLC (500 µm, PE/DCM/acetone, 4/4/0.1, eluted twice) to yield $\mathbf{11_{N\text{-}\mathit{cis}}}$ as a white powder (2 mg, 1 µmol, 20% yield).

^{1}H NMR (600 MHz, Acetone-d_6, 298 K) δ = 7.78 – 7.08 (m, 12H, $H_{9,10,11,14}$), 7.05 – 7.01 (m, 2H, H_5), 6.99 – 6.87 (m, 13H, $H_{h,m,o,15}$), 6.75 – 6.70 (m, 2H, H_4), 4.82 – 4.75 (m, 1H, H_d), 4.57 (s, 2H, H_2), 4.42 – 4.36 (m, 1H, H_d), 4.31 – 4.26 (m, 1H, H_e), 4.10 – 4.04 (m, 1H, H_e), 3.91 (t, J = 7.3 Hz, 2H, H_u), 3.82 – 3.68 (m, 40H, $H_{j,q,k,17}$), 3.42 – 3.37 (m, 2H, H_{ff}), 1.98 – 1.96 (m, 12H, $H_{a,r}$), 1.18 – 1.15 (m, 2H, H_v), 0.78 – 0.72 (m, 2H, H_z), 0.67 – 0.56 (m, 4H, $H_{aa,y}$), 0.32 – 0.15 (m, 8H, $H_{w,ee,x,bb}$), -0.55 – -0.63 (m, 2H, H_{cc}), -0.85 – -0.94 (m, 2H, H_{dd}).
^{13}C NMR (151 MHz, Acetone-d_6, 298 K) δ = 172.13 (C_c), 171.90 (C_t), 169.30 (C_1), 166.22 (C_{12}), 157.81 (C_{16}), 156.24 (C_3), 151.57, 151.16, 151.15, 151.13, 151.05, 151.04, 151.01 ($C_{i,p}$), 149.97 (C_f), 139.99 (C_6), 132.26 (C_{13}), 129.45, 129.19, 128.85, 128.69, 128.67, 128.61, 128.57, 128.55 ($C_{g,l,n,9,10,11}$), 123.42 (C_5), 118.86 (C_{14}), 115.43 (C_4), 115.32, 115.23, 114.08, 113.74, 113.70, 113.66, 113.59, 113.56, 113.52, 113.50 ($C_{15,h,m,o}$), 94.68 (C_7), 67.13, 67.10 ($C_{e,u}$), 66.41 (C_{ff}), 66.25 (C_2), 65.46 (C_d), 57.54 (C_s), 57.15 (C_b), 55.78, 55.75, 55.69, 55.59, 55.58, 55.49 ($C_{j,q,17}$), 31.43 (C_{aa}), 31.29 (C_z), 31.08, 31.07 ($C_{a,r}$), 31.01, 30.93 ($C_{bb,y}$), 30.29 – 29.40 ($C_{k,cc,x}$, overlapped with solvent peak), 29.12 (C_v), 28.73 (C_{ee}), 25.60 (C_w), 24.35 (C_{dd}). (Peak C_8 is too broad and weak to show up.)
HRMS-ESI(+): 1763.5939 $[M+Na]^+$, calculated for $C_{95}H_{110}Br_2N_2O_{19}Na^+$: 1763.5962.

3.2.2 Synthesis of $\mathbf{11}_{\mathbf{N}\text{-}\boldsymbol{trans}}$

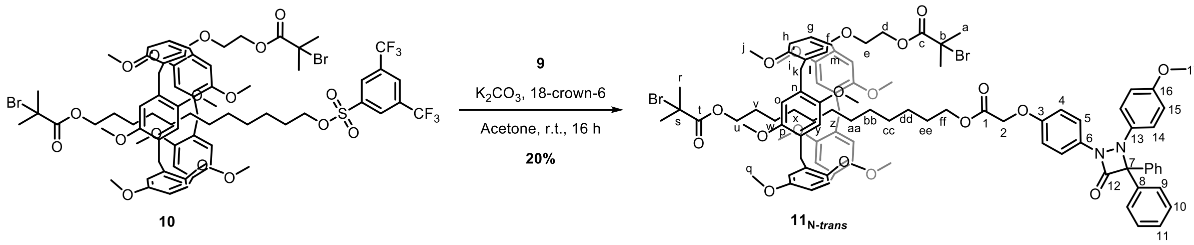

To a solution of **9** (2.8 mg, 6 µmol, 1.0 eq.) in acetone (1 mL) was added K_2CO_3 (0.8 mg, 6 µmol, 1.0 eq.) and 18-crown-6 (1.5 mg, 6 µmol, 1.0 eq.). The mixture was stirred for 2 h at room temperature. **10** (9 mg, 6 µmol, 1.0 eq.) was added and the mixture stirred for a further 16 h at room temperature. The solution was filtered, and the filtrate concentrated under vacuum. The residue was purified by preparative TLC (500 µm, PE/DCM/acetone, 4/4/0.1, eluted twice) to yield $\mathbf{11}_{\mathbf{N}\text{-}\boldsymbol{trans}}$ as a white powder (2 mg, 1 µmol, 20% yield).

^{1}H NMR (600 MHz, Acetone-d_6, 298 K) δ 7.92 – 7.05 (m, 12H, $H_{9,10,11,14}$), 7.03 – 6.99 (m, 4H, $H_{5,15}$), 6.99 – 6.88 (m, 10H, $H_{h,m,o}$), 6.68 – 6.63 (m, 2H, H_4), 4.82 – 4.76 (m, 1H, H_d), 4.73 (s, 2H, H_2), 4.45 – 4.38 (m, 1H, H_d), 4.31 – 4.26 (m, 1H, H_e), 4.10 – 4.04 (m, 1H, H_e), 3.92 – 3.87 (m, 2H, H_u), 3.82 – 3.68 (m, 37H, $H_{j,q,k}$), 3.61 (s, 3H, H_{17}), 3.50 – 3.44 (m, 3H, H_{ff}), 1.98 – 1.96 (m, 12H, $H_{a,r}$), 1.13 – 1.11 (m, 2H, H_v), 0.77 – 0.69 (m, 2H, H_z), 0.65 – 0.59 (m, 2H, H_{aa}), 0.59 – 0.52 (m, 2H, H_y), 0.39 – 0.31 (m, 2H, H_{ee}), 0.25 – 0.10 (m, 6H, $H_{w,bb,x}$), -0.51 – -0.60 (m, 2H, H_{cc}), -0.75 – -0.83 (m, 2H, H_{dd}).
^{13}C NMR (151 MHz, Acetone-d_6, 298 K) δ = 172.10 (C_c), 171.91 (C_t), 169.40 (C_1), 166.28 (C_{12}), 157.92 (C_{16}), 156.09 (C_3), 151.57, 151.16, 151.13, 151.05, 151.01 ($C_{i,p}$), 149.99 (C_f), 138.91 (C_6), 133.09 (C_{13}), 129.47, 129.26, 128.86, 128.70, 128.68, 128.62, 128.57, 128.56 ($C_{g,l,n,9,10,11}$), 123.56 (C_5), 118.69 (C_{14}), 116.15 (C_{15}), 115.37, 114.55, 114.10, 113.74, 113.69, 113.65, 113.60, 113.55, 113.52, 113.50 ($C_{4,h,m,o}$), 94.69 (C_7), 67.17, 67.14 ($C_{e,u}$), 66.48 (C_{ff}), 66.30 (C_2), 65.45 (C_d), 57.54 (C_s), 57.16 (C_b), 55.80, 55.78, 55.75, 55.69, 55.58, 55.53, 55.49 ($C_{j,q,17}$), 31.41 (C_{aa}), 31.29 (C_z), 31.09, 31.07, 31.05 ($C_{a,r}$), 31.02, 30.93 ($C_{bb,y}$), 30.21 – 29.47 ($C_{k,cc,x}$, overlapped with solvent peak), 29.11 (C_v), 28.82 (C_{ee}), 25.53 (C_w), 24.48 (C_{dd}). (Peak C_8 is too broad and weak to show up.)
HRMS-ESI(+): 1763.5929 $[M+Na]^+$, calculated for $C_{95}H_{110}Br_2N_2O_{19}Na^+$: 1763.5962.

3.2.3 Synthesis of $\mathbf{11}_{\mathbf{C}\text{-}\boldsymbol{cis}}$

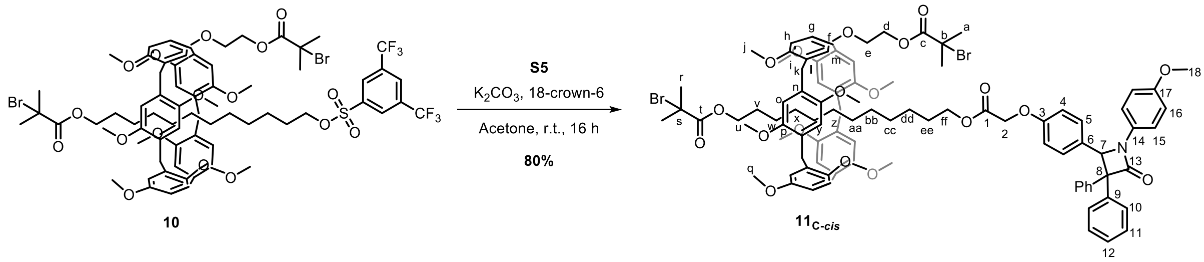

To a solution of **S5** (2.8 mg, 6 µmol, 1.0 eq.) in acetone (1 mL) was added K_2CO_3 (0.8 mg, 6 µmol, 1.0 eq.) and 18-crown-6 (1.5 mg, 6 µmol, 1.0 eq.). The mixture was stirred for 2 h at room temperature. **10** (9 mg, 6 µmol, 1.0 eq.) was added and the mixture stirred for a further 16 h at room temperature. The solution was filtered, and the filtrate concentrated under vacuum. The residue was purified by preparative TLC (500 µm, PE/DCM/acetone, 4/4/0.1, eluted twice) to yield $\mathbf{11}_{\mathbf{C}\text{-}\boldsymbol{cis}}$ as a white powder (8 mg, 5 µmol, 80% yield).

^{1}H NMR (400 MHz, Acetone-d_6, 298 K) δ = 7.79 – 7.71 (m, 2H, H_{10}), 7.46 – 7.36 (m, 4H, $H_{11,15}$), 7.33 – 7.17 (m, 5H, $H_{12,5,10'}$), 7.16 – 7.04 (m, 3H, $H_{11',12'}$), 7.01 – 6.84 (m, 12H, $H_{h,m,o,16}$), 6.82 – 6.76 (m, 2H, H_4), 6.07 (s, 1H, H_7), 4.87 – 4.74 (m, 1H, H_d), 4.64 (s, 2H, H_2), 4.46 – 4.36 (m, 1H, H_d), 4.34 – 4.25 (m, 1H, H_e),

4.15 – 4.02 (m, 1H, H_e), 3.92 (t, J = 7.3 Hz, 2H, H_u), 3.85 – 3.66 (m, 40H, $H_{j,q,k,18}$), 3.45 – 3.35 (m, 2H, H_{ff}), 1.99 – 1.95 (m, 12H, $H_{a,r}$), 1.19 – 1.15 (m, 2H, H_v), 0.82 – 0.71 (m, 2H, H_z), 0.69 – 0.54 (m, 4H, $H_{aa,y}$), 0.36 – 0.15 (m, 8H, $H_{w,ee,x,bb}$), -0.54 – -0.66 (m, 2H, H_{cc}), -0.84 – -0.98 (m, 2H, H_{dd}).

^{13}C NMR (151 MHz, Acetone-d_6, 298 K) δ = 172.13 (C_c), 171.90 (C_t), 169.23 (C_1), 166.96 (C_{13}), 158.90 (C_3), 157.24, 157.23 (C_{17}), 151.57, 151.15, 151.13, 151.05, 151.04, 151.01 ($C_{i,p}$), 149.98 (C_f), 142.40, 142.38 (C_9), 139.17 ($C_{9'}$), 131.81, 131.78 (C_{14}), 130.19, 129.48, 129.26, 129.16, 128.85, 128.78, 128.70, 128.61, 128.57, 128.56, 128.34, 128.33 ($C_{5,11,10',11',10,6,g,l,n}$), 128.14 ($C_{12}$), 127.55 ($C_{12'}$), 119.76, 119.74 ($C_{15}$), 115.34, 115.22, 115.11, 114.10, 114.08, 113.74, 113.72, 113.70, 113.66, 113.60, 113.56, 113.52 ($C_{4,16,h,m,o}$), 72.93, 72.92 (C_8), 67.13, 67.09 ($C_{e,u}$), 66.85, 66.83 (C_7), 66.47 (C_{ff}), 65.98, 65.97 (C_2), 65.48 (C_d), 57.54 (C_s), 57.15 (C_b), 55.82, 55.78, 55.74, 55.69, 55.67, 55.61, 55.59 ($C_{j,q,18}$), 31.43 (C_{aa}), 31.30 (C_z), 31.08, 31.07 ($C_{a,r}$), 31.01, 30.94 ($C_{bb,y}$), 30.24 – 29.51 ($C_{k,cc,x}$, overlapped with solvent peak), 29.12 (C_v), 28.74, 28.73 (C_{ee}), 25.61 (C_w), 24.33 (C_{dd}).

HRMS-APCI(+): 1762.5980 $[M+Na]^+$, calculated for $C_{96}H_{111}Br_2NO_{19}Na^+$: 1762.6009.

3.2.4 Synthesis of **11$_{C\text{-}trans}$**

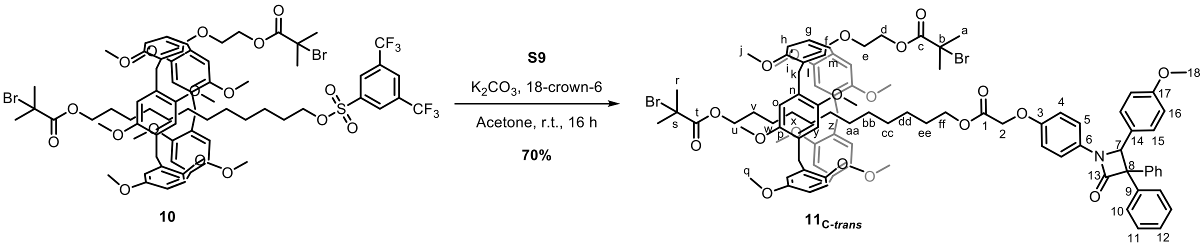

To a solution of **S9** (2.8 mg, 6 µmol, 1.0 eq.) in acetone (1 mL) was added K_2CO_3 (0.8 mg, 6 µmol, 1.0 eq.) and 18-crown-6 (1.5 mg, 6 µmol, 1.0 eq.). The mixture was stirred for 2 h at room temperature. **10** (9 mg, 6 µmol, 1.0 eq.) was added and the mixture stirred for a further 16 h at room temperature. The solution was filtered, and the filtrate concentrated under vacuum. The residue was purified by preparative TLC (500 µm, PE/DCM/acetone, 4/4/0.1, eluted twice) to yield **11$_{C\text{-}trans}$** as a white powder (7 mg, 4 µmol, 70% yield).

^{1}H NMR (400 MHz, Acetone-d_6, 298 K) δ = 7.77 – 7.71 (m, 2H, H_{10}), 7.47 – 7.37 (m, 4H, $H_{5,11}$), 7.33 – 7.26 (m, 1H, H_{12}), 7.22 – 7.16 (m, 4H, $H_{15,10'}$), 7.11 – 7.01 (m, 3H, $H_{11',12'}$), 6.99 – 6.85 (m, 12H, $H_{h,m,o,4}$), 6.76 – 6.70 (m, 2H, H_{16}), 6.03 (s, 1H, H_7), 4.84 – 4.74 (m, 1H, H_d), 4.69 (s, 2H, H_2), 4.45 – 4.33 (m, 1H, H_d), 4.32 – 4.23 (m, 1H, H_e), 4.12 – 4.01 (m, 1H, H_e), 3.94 – 3.87 (m, 2H, H_u), 3.85 – 3.63 (m, 40H, $H_{j,q,k,18}$), 3.48 – 3.39 (m, 2H, H_{ff}), 1.99 – 1.93 (m, 12H, $H_{a,r}$), 1.18 – 1.11 (m, 2H, H_v), 0.80 – 0.68 (m, 2H, H_z), 0.67 – 0.51 (m, 4H, $H_{aa,y}$), 0.37 – 0.14 (m, 8H, $H_{w,ee,x,bb}$), -0.50 – -0.65 (m, 2H, H_{cc}), -0.75 – -0.93 (m, 2H, H_{dd}).

^{13}C NMR (151 MHz, Acetone-d_6, 298 K) δ = 172.09 (C_c), 171.90 (C_t), 169.43, 169.42 (C_1), 167.12 (C_{13}), 160.50, 160.49 (C_{17}), 155.55 (C_3), 151.56, 151.15, 151.04, 151.00 ($C_{i,p}$), 149.98 (C_f), 142.43, 142.42 (C_9), 139.16, 139.15 ($C_{9'}$), 132.66 (C_6), 130.07 (C_{15}), 129.49, 129.45, 129.20, 128.85, 128.72, 128.69, 128.67, 128.63, 128.61, 128.56 ($C_{11,10',11',g,l,n}$), 128.29 (C_{10}), 128.13 (C_{12}), 127.93, 127.92 (C_{14}), 127.48 ($C_{12'}$), 119.72, 119.70 (C_5), 116.00, 115.97 (C_4), 115.33, 114.53, 114.09, 114.08, 113.73, 113.69, 113.65, 113.58, 113.55, 113.51, 113.48 ($C_{h,m,o,16}$), 72.95, 72.93 (C_8), 67.13, 67.12, 67.06 ($C_{e,u,7}$), 66.45 (C_{ff}), 66.24, 66.17 (C_2), 65.43 (C_d), 57.53 (C_s), 57.15 (C_b), 55.77, 55.74, 55.69, 55.56 ($C_{j,q}$), 55.42 (C_{18}), 31.41 (C_{aa}), 31.29 (C_z), 31.07 ($C_{a,r}$), 31.01, 30.93 ($C_{bb,y}$), 30.18 – 29.50 ($C_{k,cc,x}$, overlapped with solvent peak), 29.11 (C_v), 28.79, 28.76 (C_{ee}), 25.58, 25.55 (C_w), 24.42, 24.38 (C_{dd}).

HRMS-APCI(+): 1762.5989 $[M+Na]^+$, calculated for $C_{96}H_{111}Br_2NO_{19}Na^+$: 1762.6009.

3.2.5 Synthesis of S11

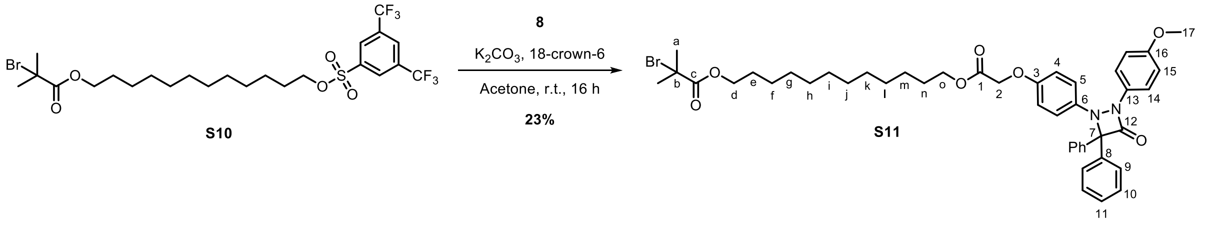

To a solution of **8** (8 mg, 16 µmol, 1.0 eq.) in acetone (1 mL) was added K_2CO_3 (2 mg, 16 µmol, 1.0 eq.) and 18-crown-6 (4 mg, 16 µmol, 1.0 eq.). The mixture was stirred for 2 h at room temperature. **S10** (10 mg, 16 µmol, 1.0 eq.) was added and the mixture stirred for a further 16 h at room temperature. The solution was filtered, and the filtrate concentrated under vacuum. The residue was purified by preparative TLC (500 µm, PE/Et_2O, 3/1, eluted twice) to yield **S11** as a white powder (3 mg, 4 µmol, 23% yield).

^{1}H NMR (600 MHz, Acetone-d_6, 298 K) δ = 8.00 – 7.01 (m, 12H, $H_{9,10,11,14}$), 7.00 – 6.89 (m, 4H, $H_{5,15}$), 6.67 (d, J = 8.7 Hz, 2H, H_4), 4.54 (s, 2H, H_2), 4.16 (t, J = 6.5 Hz, 2H, H_d), 4.10 (t, J = 6.6 Hz, 2H, H_o), 3.76 (s, 3H, H_{17}), 1.92 (s, 6H, H_a), 1.71 – 1.64 (m, 2H, H_e), 1.62 – 1.56 (m, 2H, H_n), 1.45 – 1.38 (m, 2H, H_f), 1.37 – 1.30 (m, 14H, $H_{g\text{-}m}$).
^{13}C NMR (151 MHz, Acetone-d_6, 298 K) δ = 171.83 (C_c), 169.30 (C_1), 166.21 (C_{12}), 157.76 (C_{16}), 156.20 (C_3), 139.83 (C_6), 132.31 (C_{13}), 131.14 – 128.38 ($C_{9,10,11}$), 123.29 (C_5), 118.76 (C_{14}), 115.37 (C_4), 115.19 (C_{15}), 94.66 (C_7), 66.51 (C_d), 65.97 (C_2), 65.43 (C_o), 57.51 (C_b), 55.78 (C_{17}), 30.97 (C_a), 30.19 – 29.50 ($C_{g\text{-}l,n}$, overlapped with solvent peak), 29.09 (C_e), 26.51, 26.49 ($C_{f,m}$). (Peak C_8 is too broad and weak to show up.)
HRMS-APCI(+): 813.3107 $[M+H]^+$, calculated for $C_{45}H_{53}BrN_2O_7H^+$: 813.3109.

3.2.6 Synthesis of S12

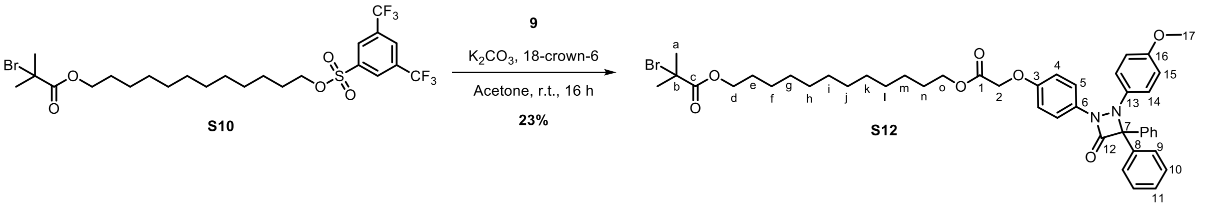

To a solution of **9** (8 mg, 16 µmol, 1.0 eq.) in acetone (1 mL) was added K_2CO_3 (2 mg, 16 µmol, 1.0 eq.) and 18-crown-6 (4 mg, 16 µmol, 1.0 eq.). The mixture was stirred for 2 h at room temperature. **S10** (10 mg, 16 µmol, 1.0 eq.) was added and the mixture stirred for a further 16 h at room temperature. The solution was filtered, and the filtrate concentrated under vacuum. The residue was purified by preparative TLC (500 µm, PE/Et_2O, 3/1, eluted twice) to yield **S12** as a white powder (3 mg, 4 µmol, 23% yield).

^{1}H NMR (600 MHz, Acetone-d_6, 298 K) δ = 7.92 – 7.02 (m, 12H, $H_{9,10,11,14}$), 7.01 – 6.89 (m, 4H, $H_{5,15}$), 6.65 (d, J = 8.5 Hz, 2H, H_4), 4.70 (s, 2H, H_2), 4.19 – 4.10 (m, 4H, $H_{d,o}$), 3.63 (s, 3H, H_{17}), 1.92 (s, 6H, H_a), 1.71 – 1.64 (m, 2H, H_e), 1.64 – 1.57 (m, 2H, H_n), 1.45 – 1.38 (m, 2H, H_f), 1.38 – 1.29 (m, 14H, $H_{g\text{-}m}$).
^{13}C NMR (151 MHz, Acetone-d_6, 298 K) δ = 171.83 (C_c), 169.38 (C_1), 166.20 (C_{12}), 157.88 (C_{16}), 156.05 (C_3), 138.93 (C_6), 132.97 (C_{13}), 131.01 – 128.46 ($C_{9,10,11}$), 123.49 (C_5), 118.57 (C_{14}), 116.04 (C_{15}), 114.51 (C_4), 94.65 (C_7), 66.51 (C_d), 66.01 (C_2), 65.51 (C_o), 57.51 (C_b), 55.53 (C_{17}), 30.97 (C_a), 30.18 – 29.51 ($C_{g\text{-}l,n}$, overlapped with solvent peak), 29.10 (C_e), 26.52, 26.48 ($C_{f,m}$). (Peak C_8 is too broad and weak to show up.)
HRMS-APCI(+): 813.3103 $[M+H]^+$, calculated for $C_{45}H_{53}BrN_2O_7H^+$: 813.3109.

3.2.7 Synthesis of S13

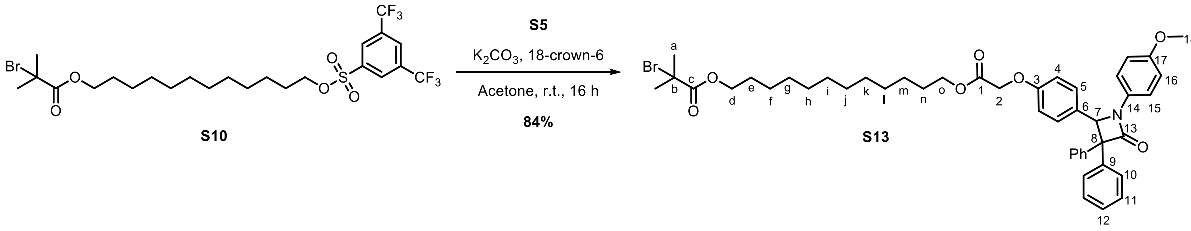

To a solution of **S5** (9 mg, 19 µmol, 1.0 eq.) in acetone (1 mL) was added K_2CO_3 (3 mg, 19 µmol, 1.0 eq.) and 18-crown-6 (5 mg, 19 µmol, 1.0 eq.). The mixture was stirred for 2 h at room temperature. **S10** (12 mg, 19 µmol, 1.0 eq.) was added and the mixture stirred for a further 16 h at room temperature. The solution was filtered, and the filtrate concentrated under vacuum. The residue was purified by preparative TLC (500 µm, PE/DCM/acetone, 4/4/0.1, eluted twice) to yield **S13** as a white powder (13 mg, 16 µmol, 84% yield).

^{1}H NMR (400 MHz, Acetone-*d*$_6$, 298 K) δ = 7.78 – 7.67 (m, 2H, H_{10}), 7.44 – 7.33 (m, 4H, $H_{11,15}$), 7.32 – 7.26 (m, 1H, H_{12}), 7.21 – 7.13 (m, 4H, $H_{5,10'}$), 7.11 – 7.00 (m, 3H, $H_{11',12'}$), 6.89 – 6.82 (m, 2H, H_{16}), 6.77 – 6.69 (m, 2H, H_4), 6.01 (s, 1H, H_7), 4.62 (s, 2H, H_2), 4.19 – 4.09 (m, 4H, $H_{d,o}$), 3.73 (s, 3H, H_{18}), 1.92 (s, 6H, H_a), 1.72 – 1.64 (m, 2H, H_e), 1.64 – 1.56 (m, 2H, H_n), 1.46 – 1.38 (m, 2H, H_f), 1.37 – 1.29 (m, 14H, $H_{g\text{-}m}$).
^{13}C NMR (101 MHz, Acetone-*d*$_6$, 298 K) δ = 171.83 (C_c), 169.23 (C_1), 166.95 (C_{13}), 158.87 (C_3), 157.19 (C_{17}), 142.38 (C_9), 139.12 ($C_{9'}$), 131.84 (C_{14}), 130.03 (C_5), 129.46 (C_{11}), 129.19 ($C_{10'}$), 128.96 (C_6), 128.71 ($C_{11'}$), 128.30 (C_{10}), 128.11 (C_{12}), 127.47 ($C_{12'}$), 119.67 (C_{15}), 115.20 (C_4), 115.07 (C_{16}), 72.89 (C_8), 66.88 (C_7), 66.50 (C_d), 65.73 (C_2), 65.48 (C_o), 57.51 (C_b), 55.67 (C_{18}), 30.98 (C_a), 30.53 – 29.20 ($C_{g\text{-}l,n}$, overlapped with solvent peak), 29.09 (C_e), 26.51, 26.50 ($C_{f,m}$).
HRMS-APCI(+): 812.3149 $[M+H]^+$, calculated for $C_{46}H_{54}BrNO_7H^+$: 812.3156.

3.2.8 Synthesis of S14

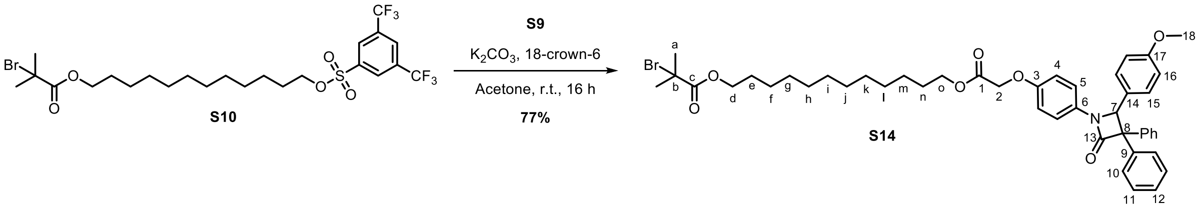

To a solution of **S9** (3 mg, 6 µmol, 1.0 eq.) in acetone (1 mL) was added K_2CO_3 (1 mg, 6 µmol, 1.0 eq.) and 18-crown-6 (2 mg, 6 µmol, 1.0 eq.). The mixture was stirred for 2 h at room temperature. **S10** (4 mg, 6 µmol, 1.0 eq.) was added and the mixture stirred for a further 16 h at room temperature. The solution was filtered, and the filtrate concentrated under vacuum. The residue was purified by preparative TLC (500 µm, PE/DCM/acetone, 4/4/0.1, eluted twice) to yield **S14** as a white powder (4 mg, 5 µmol, 77% yield).

^{1}H NMR (400 MHz, Acetone-*d*$_6$, 298 K) δ = 7.76 – 7.71 (m, 2H, H_{10}), 7.43 – 7.34 (m, 4H, $H_{11,5}$), 7.32 – 7.26 (m, 1H, H_{12}), 7.21 – 7.14 (m, 4H, $H_{15,10'}$), 7.11 – 7.00 (m, 3H, $H_{11',12'}$), 6.90 – 6.83 (m, 2H, H_4), 6.75 – 6.69 (m, 2H, H_{16}), 6.00 (s, 1H, H_7), 4.67 (s, 2H, H_2), 4.16 (t, *J* = 6.5 Hz, 2H, H_d), 4.12 (t, *J* = 6.6 Hz, 2H, H_o), 3.69 (s, 3H, H_{18}), 1.92 (s, 6H, H_a), 1.72 – 1.64 (m, 2H, H_e), 1.63 – 1.57 (m, 2H, H_n), 1.48 – 1.40 (m, 2H, H_f), 1.39 – 1.29 (m, 14H, $H_{g\text{-}m}$).
^{13}C NMR (151 MHz, Acetone-*d*$_6$, 298 K) δ = 171.84 (C_c), 169.41 (C_1), 167.08 (C_{13}), 160.46 (C_{17}), 155.53 (C_3), 142.40 (C_9), 139.17 ($C_{9'}$), 132.57 (C_6), 130.02 (C_{15}), 129.47 (C_{11}), 129.20 ($C_{10'}$), 128.70 ($C_{11'}$), 128.30

(C_{10}), 128.12 (C_{12}), 127.95 (C_{14}), 127.47 ($C_{12'}$), 119.59 (C_5), 115.92 (C_4), 114.49 (C_{16}), 72.92 (C_8), 67.02 (C_7), 66.52 (C_d), 65.95 (C_2), 65.47 (C_o), 57.51 (C_b), 55.44 (C_{18}), 30.98 (C_a), 30.31 – 29.35 ($C_{g\text{-}l,n}$, overlapped with solvent peak), 29.10 (C_e), 26.52, 26.48 ($C_{f,m}$).
HRMS-APCI(+): 812.3153 $[M+H]^+$, calculated for $C_{46}H_{54}BrNO_7H^+$: 812.3156.

3.3 Synthesis of Reference Compounds

3.3.1 Synthetic Routes to S17

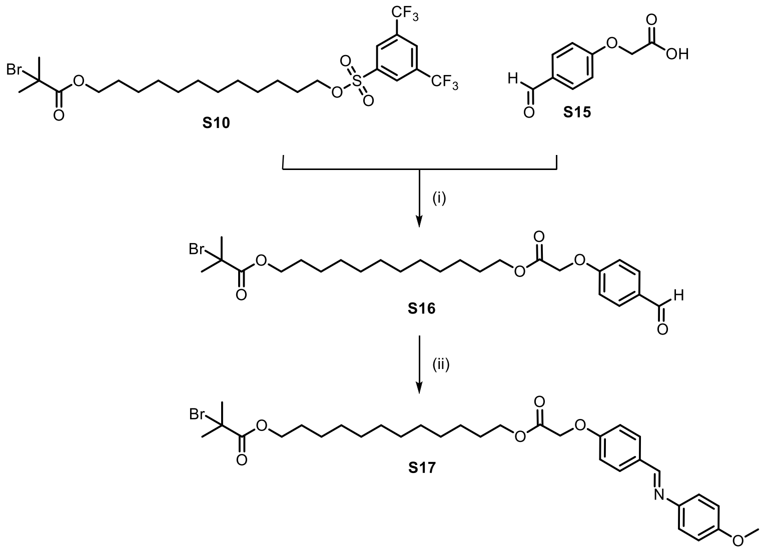

Scheme S4. Synthetic routes to **S17**. Conditions: (i) K_2CO_3, 18-crown-6, Acetone, r.t., 16 h, 61% yield; (ii) p-Anisidine, DCM, 40 °C, rotary evaporator, quantitative yield.

3.3.2 Synthesis of S16

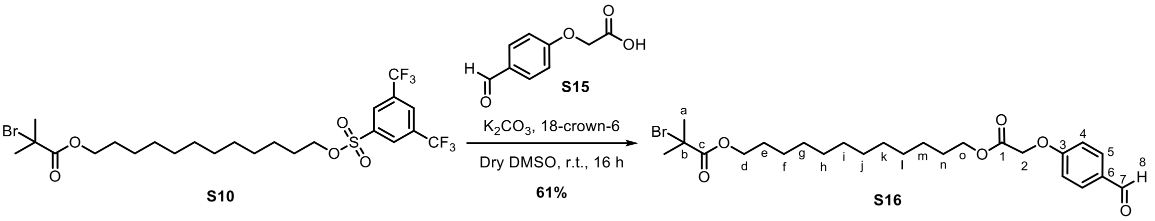

To a solution of **S15** (17 mg, 96 µmol, 1.0 eq.) in dry DMSO (2 mL) was added K_2CO_3 (13 mg, 96 µmol, 1.0 eq.) and 18-crown-6 (25 mg, 96 µmol, 1.0 eq.). The mixture was stirred for 2 h at room temperature. **S10** (60 mg, 96 µmol, 1.0 eq.) was added and the mixture stirred for a further 16 h at room temperature. The reaction mixture was washed by water (2 x 5 mL) and brine (1 x 5 mL) after added DCM (5 mL). The organic layer was collected and dried with magnesium sulfate. The mixture was filtered and concentrated. The residue was purified by preparative TLC (2000 µm, PE/EtOAc, 6/1, eluted twice) to yield **S16** as a white powder (30 mg, 59 µmol, 61% yield).

^{1}H NMR (400 MHz, Acetone-d_6, 298 K) δ = 9.92 (s, 1H, H_8), 7.93 – 7.84 (m, 2H, H_5), 7.17 – 7.09 (m, 2H, H_4), 4.90 (s, 2H, H_2), 4.19 – 4.14 (m, 4H, $H_{o,d}$), 1.92 (s, 6H, H_a), 1.71 – 1.59 (m, 4H, $H_{e,n}$), 1.47 – 1.28 (m, 16H, $H_{f\text{-}m}$).
^{13}C NMR (101 MHz, Acetone-d_6, 298 K) δ = 191.19 (C_7), 171.79 (C_c), 168.90 (C_1), 163.80 (C_3), 132.41 (C_5), 131.69 (C_6), 115.80 (C_4), 66.47 (C_d), 65.69 ($C_{2,o}$), 57.49 (C_b), 30.92 (C_a), 30.47 – 29.21 ($C_{e,g\text{-}l,n}$, overlapped with solvent peak), 26.49 ($C_{f,m}$).
HRMS-ESI(+): 535.1659 $[M+Na]^+$, calculated for $C_{25}H_{37}BrO_6Na^+$: 535.1666.

3.3.3 Synthesis of S17

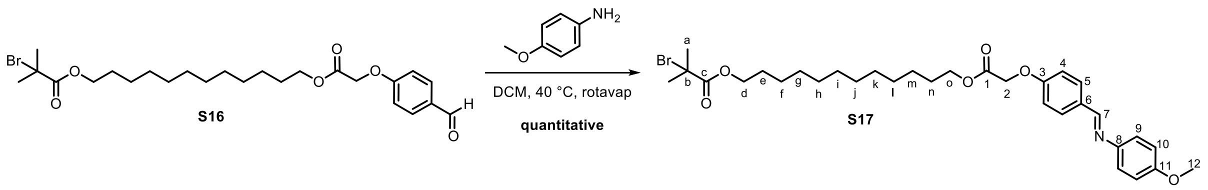

S16 (10 mg, 20 µmol, 1.0 eq.) and p-anisidine (2 mg, 20 µmol, 1.0 eq.) were dissolved in DCM (5 mL) in a round bottle flask. Evaporate solvent by rotary evaporator at 40 °C. The process of dissolution and evaporation was repeated three times to yield **S17** as a pale-yellow solid (12 mg, 20 µmol, quantitative yield).

^{1}H NMR (400 MHz, Acetone-d_6, 298 K) δ = 8.54 (s, 1H, H_7), 7.93 – 7.87 (m, 2H, H_5), 7.28 – 7.22 (m, 2H, H_9), 7.09 – 7.03 (m, 2H, H_4), 6.99 – 6.93 (m, 2H, H_{10}), 4.84 (s, 2H, H_2), 4.19 – 4.12 (m, 4H, $H_{o,d}$), 3.81 (s, 3H, H_{12}), 1.92 (s, 6H, H_a), 1.71 – 1.60 (m, 4H, $H_{e,n}$), 1.45 – 1.29 (m, 16H, $H_{f\text{-}m}$).
^{13}C NMR (101 MHz, Acetone-d_6, 298 K) δ = 171.79 (C_c), 169.20 (C_1), 161.29 (C_3), 159.07 (C_{11}), 157.91 (C_7), 145.89 (C_8), 131.36 (C_6), 130.91 (C_5), 122.97 (C_9), 115.53 (C_4), 115.09 (C_{10}), 66.47 (C_d), 65.68 (C_2), 65.58 (C_o), 57.48 (C_b), 55.69 (C_{12}), 30.93 (C_a), 30.50 – 29.15 ($C_{e,g\text{-}l,n}$, overlapped with solvent peak), 26.51, 26.49 ($C_{f,m}$).
HRMS-ESI(+): 618.2420 [M+H]$^+$, calculated for $C_{32}H_{44}BrO_6H^+$: 618.2425.

4 Synthesis of Polymers

4.1 Representative Procedure for SET-LRP of Methyl Acrylate Using Mechanophore Initiators

Methyl acrylate was filtered through basic alumina to remove the inhibitor prior to use. A stock catalytic solution of Me_6TREN (16 µL, 0.060 mmol) and $CuBr_2$ (5.6 mg, 0.025 mmol) in dry DMSO (1 mL) was prepared. To a 5 mL microwave vial was added the appropriate initiator compound along with catalytic solution, methyl acrylate and dry DMSO. This solution was degassed by bubbling N_2 for 10 min. A Cu(0) wire wrapped around a stirrer bar, having been cleaned in 12 N HCl for 10 min, was added to the reaction mixture. The reaction mixture was degassed for a further 2 min before being allowed to stir for 15 - 40 min (until the extent of polymerization, as determined approximately by the increasing viscosity of the solution, was deemed acceptable). The solution was added dropwise to a solution of vigorously stirred methanol; the precipitated polymer was recovered and dried under vacuum for two days to yield a white material. Molecular weight and dispersity indices were determined using an analytical SEC that had been calibrated with polystyrene standards.

4.2 Synthesis of Mechanophore and Control Polymers

4.2.1 Synthesis of Polymer $\mathbf{1}_{N\text{-}cis}$

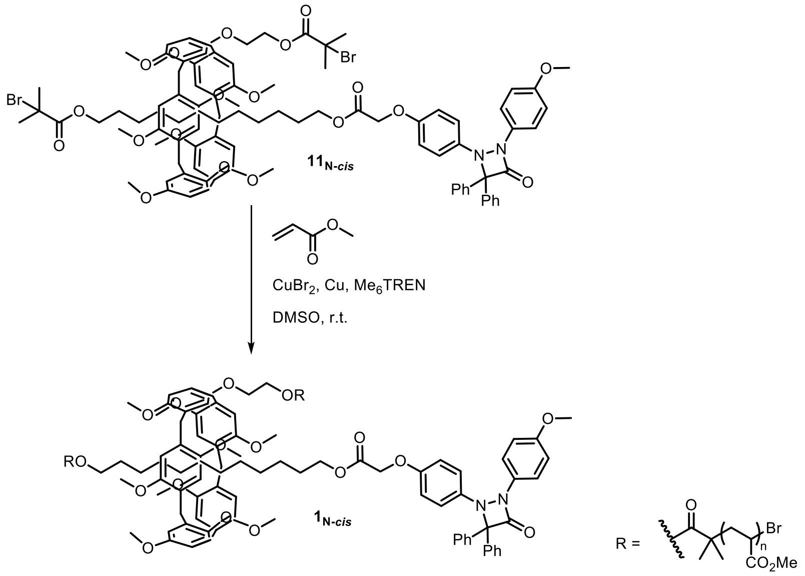

Synthesis followed the representative procedure. $\mathbf{11}_{N\text{-}cis}$ (2.0 mg, 1.1 µmol, 1.0 eq.), 9 µL of catalytic solution ($CuBr_2$: 0.2 µmol, 0.2 eq.; Me_6TREN: 0.6 µmol, 0.5 eq.), methyl acrylate (205 µL, 2.3 mmol, 2000.0 eq.), Cu (0) wire (~3 cm, ~30 mg, 0.5 mmol, ~400.0 eq.) and dry DMSO (205 µL) were used in the reaction to yield polymer $\mathbf{1}_{N\text{-}cis}$ (75 mg, M_n = 149 kDa; $Đ$ = 1.42).

4.2.2 Synthesis of Polymer $\mathbf{1}_{N\text{-}trans}$

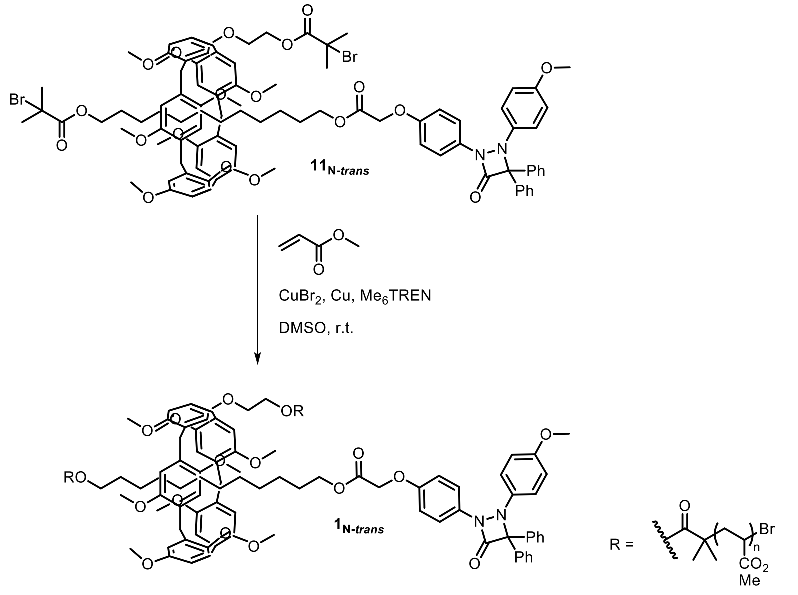

Synthesis followed the representative procedure. $\mathbf{11}_{N\text{-}trans}$ (2.0 mg, 1.1 µmol, 1.0 eq.), 9 µL of catalytic solution ($CuBr_2$: 0.2 µmol, 0.2 eq.; Me_6TREN: 0.6 µmol, 0.5 eq.), methyl acrylate (205 µL, 2.3 mmol, 2000.0 eq.), Cu (0) wire (~3 cm, ~30 mg, 0.5 mmol, ~400.0 eq.) and dry DMSO (205 µL) were used in the reaction to yield polymer $\mathbf{1}_{N\text{-}trans}$ (70 mg, M_n = 131 kDa; $Đ$ = 1.39).

4.2.3 Synthesis of Polymer $1_{C\text{-}cis}$

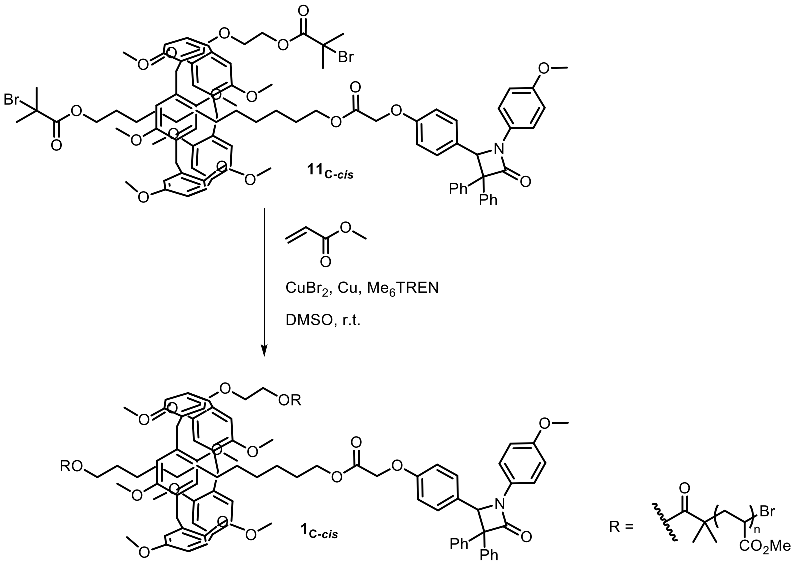

Synthesis followed the representative procedure. $\mathbf{11}_{C\text{-}cis}$ (3.5 mg, 2.0 µmol, 1.0 eq.), 16 µL of catalytic solution ($CuBr_2$: 0.4 µmol, 0.2 eq.; Me_6TREN: 1.0 µmol, 0.5 eq.), methyl acrylate (359 µL, 4.0 mmol, 2000.0 eq.), Cu (0) wire (~3 cm, ~30 mg, 0.5 mmol, ~240.0 eq.) and dry DMSO (359 µL) were used in the reaction to yield polymer $\mathbf{1}_{C\text{-}cis}$ (108 mg, M_n = 138 kDa; $Đ$ = 1.18).

4.2.4 Synthesis of Polymer $1_{C\text{-}trans}$

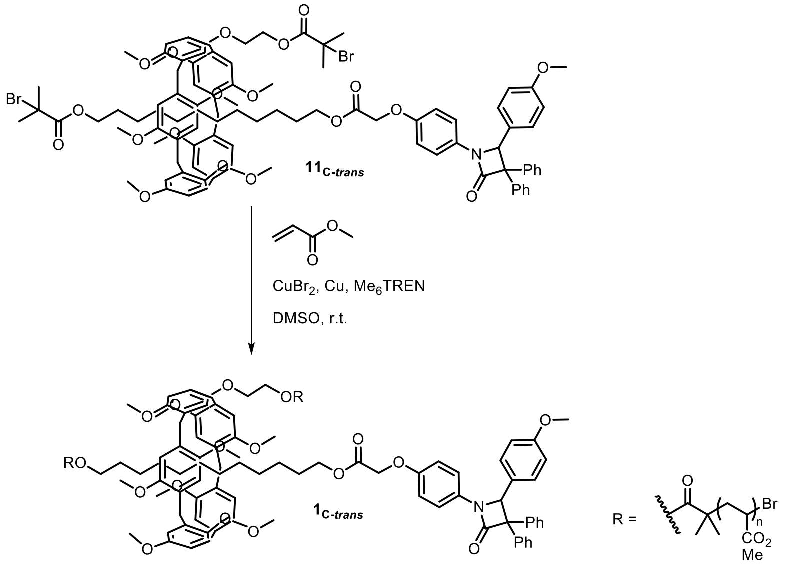

Synthesis followed the representative procedure. $\mathbf{11}_{C\text{-}trans}$ (3.5 mg, 2.0 µmol, 1.0 eq.), 16 µL of catalytic solution ($CuBr_2$: 0.4 µmol, 0.2 eq.; Me_6TREN: 1.0 µmol, 0.5 eq.), methyl acrylate (359 µL, 4.0 mmol, 2000.0 eq.), Cu (0) wire (~3 cm, ~30 mg, 0.5 mmol, ~240.0 eq.) and dry DMSO (359 µL) were used in the reaction to yield polymer $\mathbf{1}_{C\text{-}trans}$ (118 mg, M_n = 147 kDa; $Đ$ = 1.17).

4.2.5 Synthesis of Polymer S18

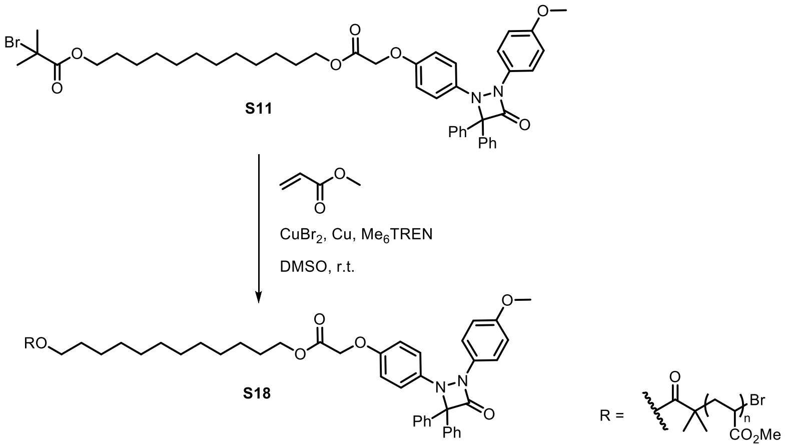

Synthesis followed the representative procedure. **S11** (1.5 mg, 1.8 µmol, 1.0 eq.), 15 µL of catalytic solution ($CuBr_2$: 0.4 µmol, 0.2 eq.; Me_6TREN: 0.9 µmol, 0.5 eq.), methyl acrylate (329 µL, 3.7 mmol, 2000.0 eq.), Cu (0) wire (~3 cm, ~30 mg, 0.5 mmol, ~250.0 eq.) and dry DMSO (329 µL) were used in the reaction to yield polymer **S18** (115 mg, M_n = 147 kDa; $Đ$ = 1.34).

4.2.6 Synthesis of Polymer S19

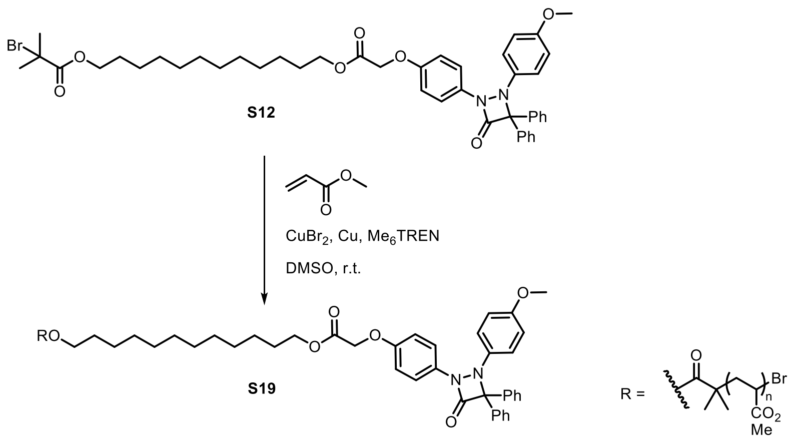

Synthesis followed the representative procedure. **S12** (1.5 mg, 1.8 µmol, 1.0 eq.), 15 µL of catalytic solution ($CuBr_2$: 0.4 µmol, 0.2 eq.; Me_6TREN: 0.9 µmol, 0.5 eq.), methyl acrylate (329 µL, 3.7 mmol, 2000.0 eq.), Cu (0) wire (~3 cm, ~30 mg, 0.5 mmol, ~250.0 eq.) and dry DMSO (329 µL) were used in the reaction to yield polymer **S19** (132 mg, M_n = 162 kDa; $Đ$ = 1.29).

4.2.7 Synthesis of Polymer S20

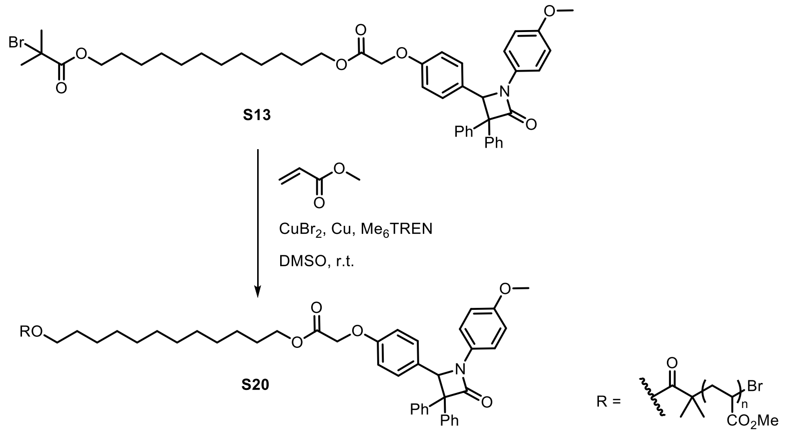

Synthesis followed the representative procedure. **S13** (2.0 mg, 2.5 μmol, 1.0 eq.), 20 μL of catalytic solution ($CuBr_2$: 0.5 μmol, 0.2 eq.; Me_6TREN: 1.2 μmol, 0.5 eq.), methyl acrylate (440 μL, 4.9 mmol, 2000.0 eq.), Cu (0) wire (~3 cm, ~30 mg, 0.5 mmol, ~200.0 eq.) and dry DMSO (440 μL) were used in the reaction to yield polymer **S20** (138 mg, M_n = 128 kDa; *Đ* = 1.37).

4.2.8 Synthesis of Polymer S21

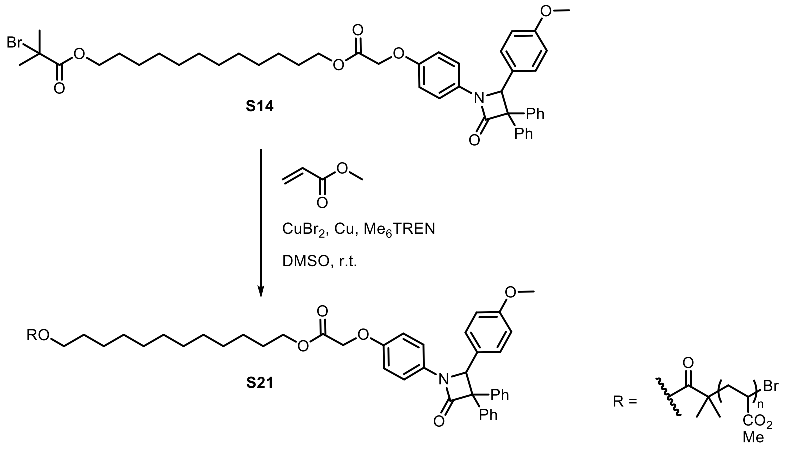

Synthesis followed the representative procedure. **S14** (2.0 mg, 2.5 μmol, 1.0 eq.), 20 μL of catalytic solution ($CuBr_2$: 0.5 μmol, 0.2 eq.; Me_6TREN: 1.2 μmol, 0.5 eq.), methyl acrylate (440 μL, 4.9 mmol, 2000.0 eq.), Cu (0) wire (~3 cm, ~30 mg, 0.5 mmol, ~200.0 eq.) and dry DMSO (440 μL) were used in the reaction to yield polymer **S21** (122 mg, M_n = 124 kDa; *Đ* = 1.37).

4.3 Synthesis of Reference Polymers

4.3.1 Synthesis of Polymer S22

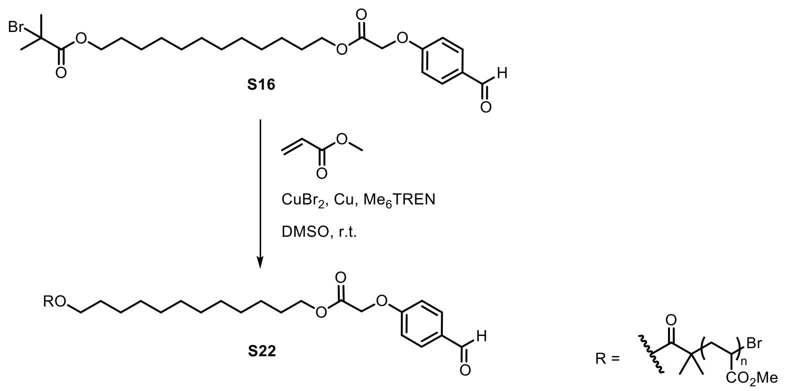

Synthesis followed the representative procedure. **S16** (1.0 mg, 2.0 µmol, 1.0 eq.), 16 µL of catalytic solution ($CuBr_2$: 0.4 µmol, 0.2 eq.; Me_6TREN: 0.9 µmol, 0.5 eq.), methyl acrylate (348 µL, 3.9 mmol, 2000.0 eq.), Cu (0) wire (~3 cm, ~30 mg, 0.5 mmol, ~250.0 eq.) and dry DMSO (348 µL) were used in the reaction to yield polymer **S22** (90 mg, M_n = 98 kDa; $Đ$ = 1.13).

4.3.2 Synthesis of Polymer 4_C

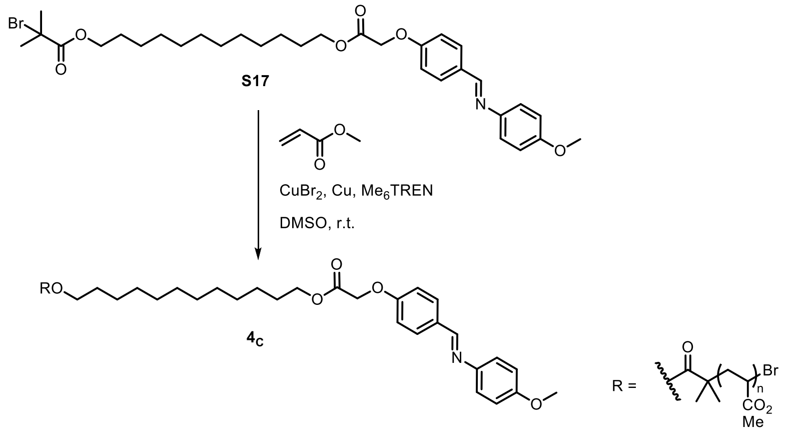

Synthesis followed the representative procedure. **S17** (4.0 mg, 6.5 µmol, 1.0 eq.), 52 µL of catalytic solution ($CuBr_2$: 1.3 µmol, 0.2 eq.; Me_6TREN: 3.1 µmol, 0.5 eq.), methyl acrylate (1156 µL, 13.0 mmol, 2000.0 eq.), Cu (0) wire (~3 cm, ~30 mg, 0.5 mmol, ~80.0 eq.) and dry DMSO (1156 µL) were used in the reaction to yield polymer **4_C** (325 mg, M_n = 114 kDa; $Đ$ = 1.42).

4.4 SEC Data for Synthesised Polymers

Table S1. M_n and $Đ$ values for all synthesised polymers.

Polymer	M_n / kDa	$Đ$
$\mathbf{1}_{N\text{-}cis}$	149	1.42
$\mathbf{1}_{N\text{-}trans}$	131	1.39
$\mathbf{1}_{C\text{-}cis}$	138	1.18
$\mathbf{1}_{C\text{-}trans}$	147	1.17
**S18**	147	1.34
**S19**	162	1.29
**S20**	128	1.37
**S21**	124	1.37
**S22**	98	1.13
$\mathbf{4}_{C}$	114	1.42

4.5 SEC Traces for Mechanophore and Control Polymers

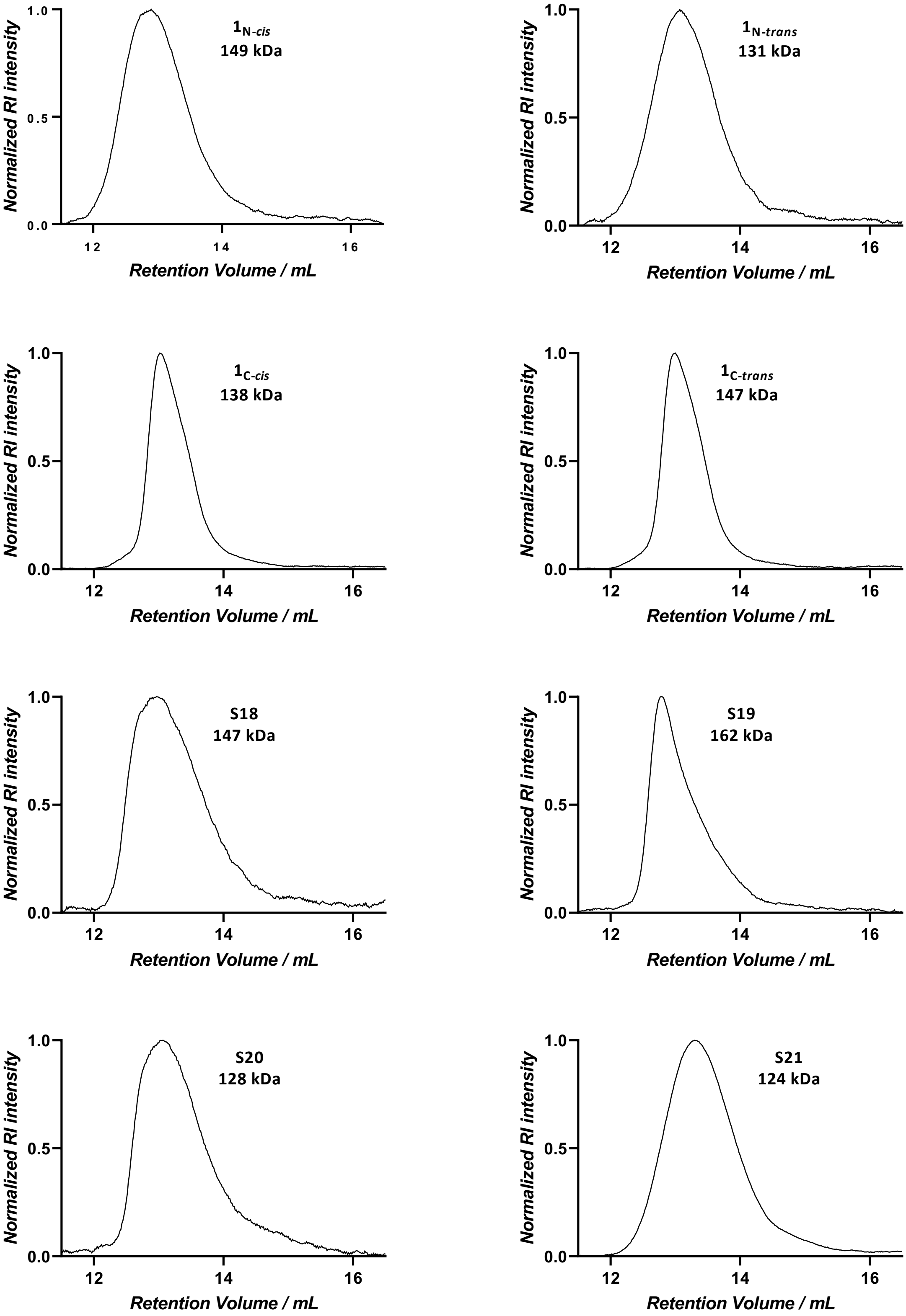

Figure S1. SEC traces for polymers $\mathbf{1}_{N\text{-}cis}$, $\mathbf{1}_{N\text{-}trans}$, $\mathbf{1}_{C\text{-}cis}$, $\mathbf{1}_{C\text{-}trans}$, **S18**, **S19**, **S20** and **S21**.

4.6 SEC Traces for Reference Polymers

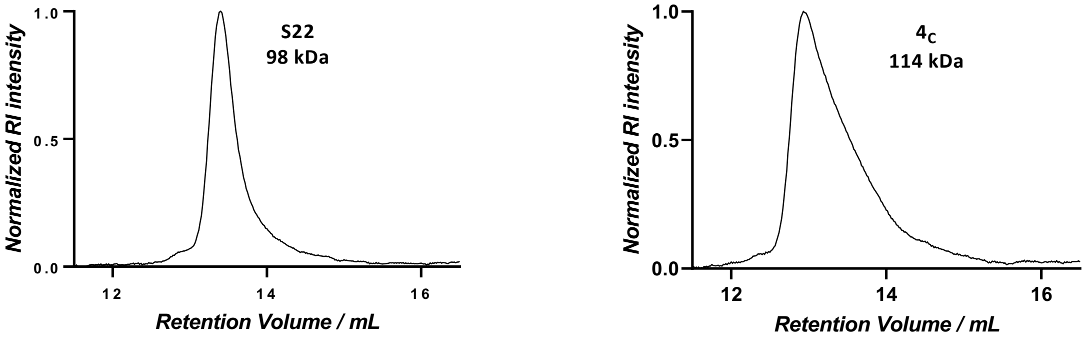

Figure S2. SEC trace of reference polymers **S22** and **4_C**.

5 Mechanophore Activation via Ultrasound

5.1 General Procedure for Sonication Experiments

The appropriate polymer (10 mg) was added to a Suslick cell and dissolved in the appropriate solvent 20 mL). The solution was degassed by bubbling N_2 through it for a minimum of 10 min prior to the start of sonication; bubbling of N_2 was also maintained throughout the experiment. The Suslick cell was cooled with an ice bath throughout the duration of the sonication to maintain a temperature of ~ 5-10 °C inside the cell. Pulsed ultrasound was applied to the system (1 s ON / 1 s OFF, 25% amplitude (13.0 W cm^{-2}), 20 kHz) for the desired period. After sonication, the solvent was evaporated and the polymer was analysed by SEC and NMR spectroscopy. The post-sonication polymer was recovered and washed with MeOH to extract any small molecules not attached to polymer chains. The remaining MeOH-washed polymer and the concentrated MeOH washings were then analysed by NMR spectroscopy.

5.2 Sonication of Mechanophore Polymer $\mathbf{1}_{\mathbf{N}\text{-}\boldsymbol{cis}}$

Sonication of mechanophore polymer $\mathbf{1}_{\mathbf{N}\text{-}\boldsymbol{cis}}$, using the methodology described in the general procedure (*Section 5.1*) and with the solvent used being THF/H_2O (9/1), was carried out twice to determine the extent of activation for retro-[2+2] cycloaddition of diazetidinone structure. SEC analysis of the sonicated polymers showed complete cleavage (M_n of the post-sonication material was less than half of that of the pre-sonication polymer).

Comparison of the ^1H NMR spectra of pre- and post-sonication polymer $\mathbf{1}_{\mathbf{N}\text{-}\boldsymbol{cis}}$ showed that retro-[2+2] cycloaddition of diazetidinone structure happened upon the pushing force with rotaxane actuator to form azo and ketene species. It is evidenced by clear formation of diazobenzene polymer along with diphenylacetic acid generated from diphenylketene trapping water while the intact diazetidinone structure decreased in relative intensity. Aromatic peaks (*d*-*g*) confirmed to be diazobenzene polymer after comparing with peaks (*k*-*n*) in reference **7**. Also, we can clearly find peaks (*h*-*j*) of diphenylacetic acid in the concentrated methanol washings.

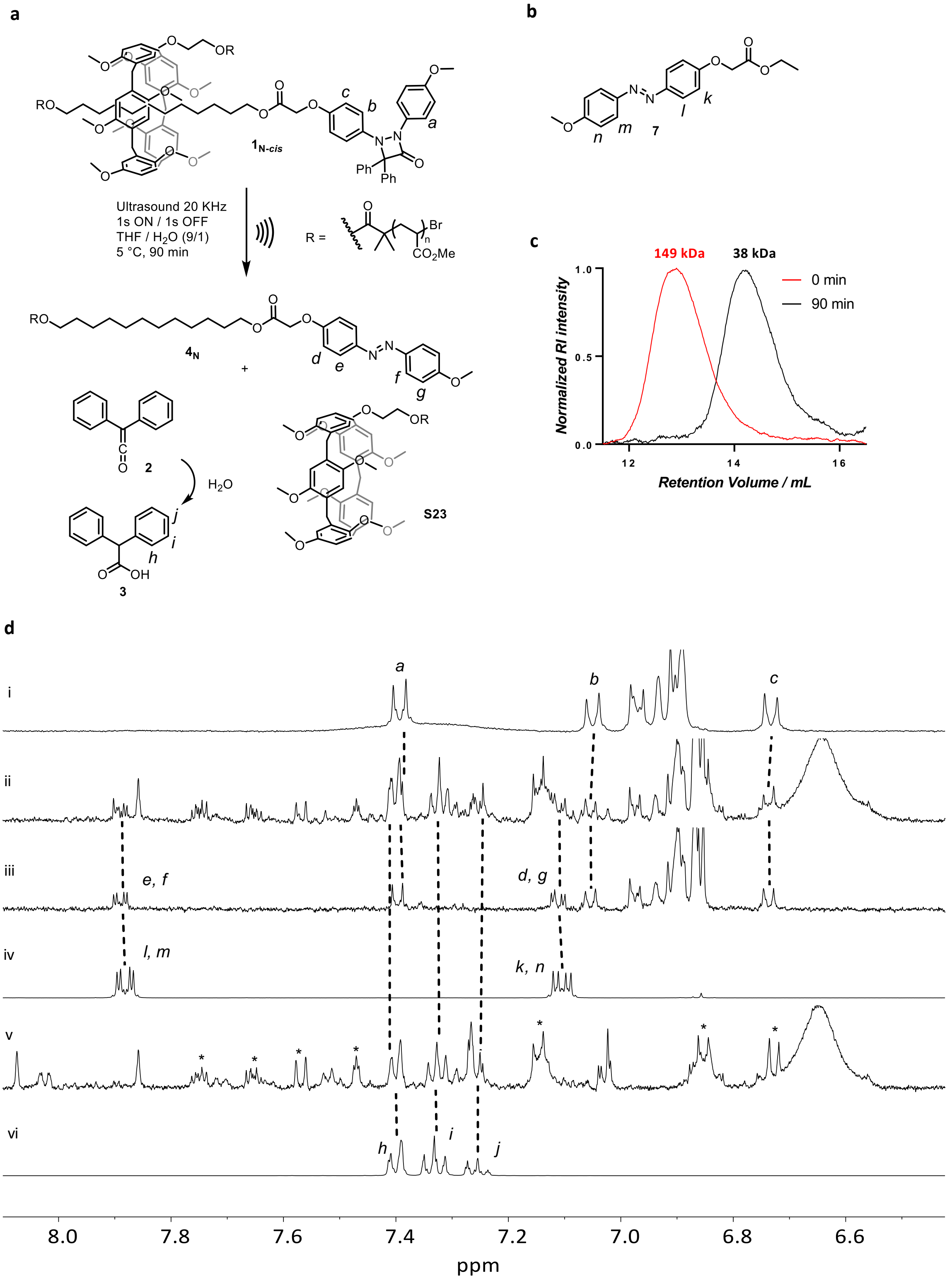

Figure S3. Sonication (run 1) of polymer **1**$_{\mathbf{N}\text{-}cis}$ in THF/H_2O (9/1). Sonication of polymer **1**$_{\mathbf{N}\text{-}cis}$ affords polymer fragments **4**$_{\mathbf{N}}$ and **3** (a). Reference compound **7** (b). SEC traces of polymer **1**$_{\mathbf{N}\text{-}cis}$ (c) with M_n values before (red) and after (black) sonication. Partial ^{1}H NMR (500 MHz, Acetone-d_6, 298 K) spectra comparison (d) of the pre-sonication polymer **1**$_{\mathbf{N}\text{-}cis}$ (i), post-sonication polymer before being washed with methanol (ii), post-sonication polymer after being washed with methanol (iii), reference compound **7** (iv), concentrated methanol washings (v) and reference compound **3** (vi). * peaks from contaminants, see section 5.8 for details.

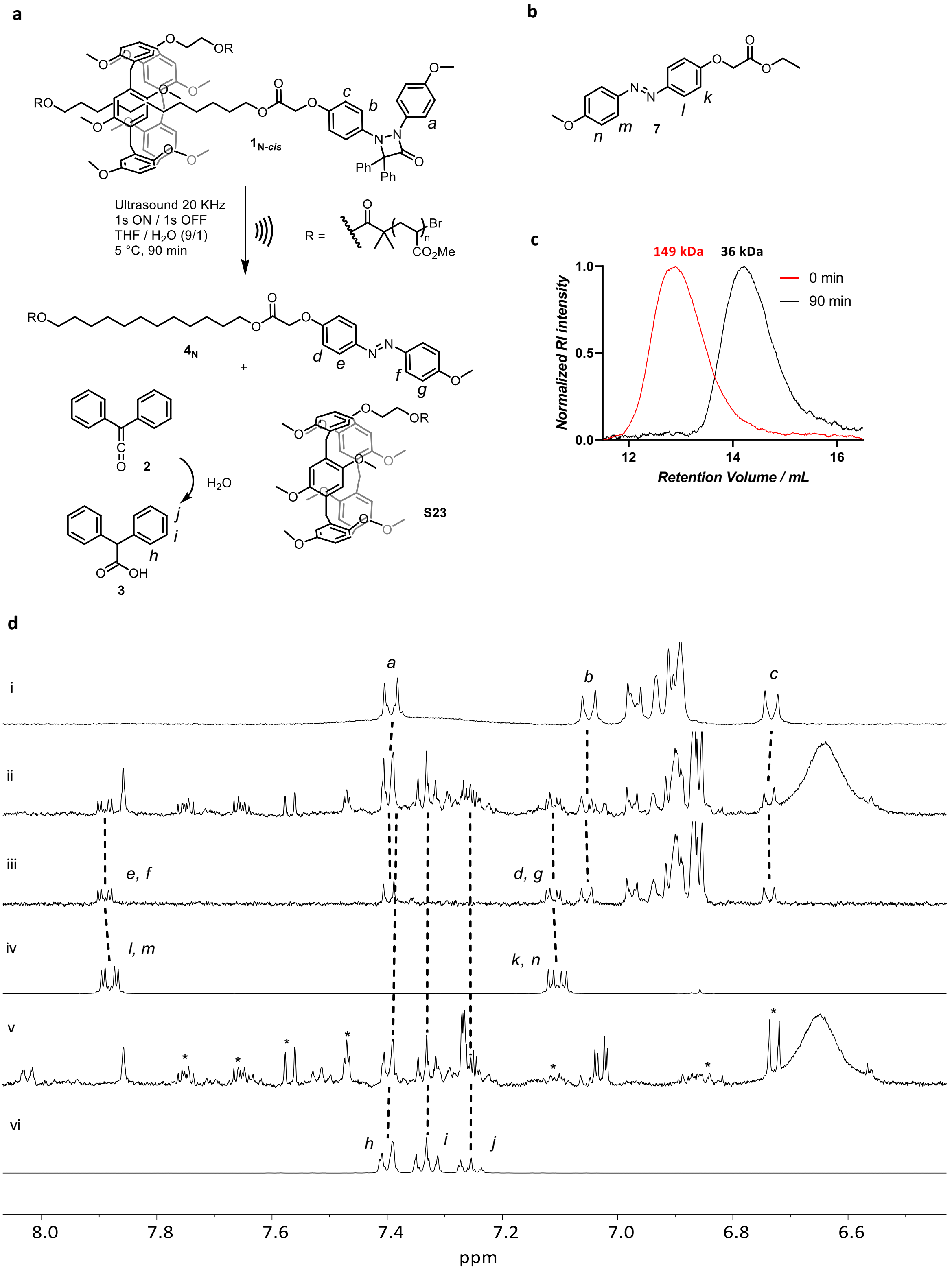

Figure S4. Sonication (run 2) of polymer $\mathbf{1}_{\mathbf{N}\text{-}\boldsymbol{cis}}$ in THF/H_2O (9/1). Sonication of polymer $\mathbf{1}_{\mathbf{N}\text{-}\boldsymbol{cis}}$ affords polymer fragments $\mathbf{4}_{\mathbf{N}}$ and **3** (a). Reference compound **7** (b). SEC traces of polymer $\mathbf{1}_{\mathbf{N}\text{-}\boldsymbol{cis}}$ (c) with M_n values before (red) and after (black) sonication. Partial ^{1}H NMR (500 MHz, Acetone-d_6, 298 K) spectra comparison (d) of the pre-sonication polymer $\mathbf{1}_{\mathbf{N}\text{-}\boldsymbol{cis}}$ (i), post-sonication polymer before being washed with methanol (ii), post-sonication polymer after being washed with methanol (iii), reference compound **7** (iv), concentrated methanol washings (v) and reference compound **3** (vi). * marks peaks from contaminants, see section 5.8 for details.

5.3 Sonication of Mechanophore Polymer $\mathbf{1}_{\mathbf{N}\text{-}\textbf{\textit{trans}}}$

Sonication of mechanophore polymer $\mathbf{1}_{\mathbf{N}\text{-}\textbf{\textit{trans}}}$, using the methodology described in the general procedure (*Section 5.1*) and with the solvent used being THF/H_2O (9/1), was carried out twice to determine the extent of activation for retro-[2+2] cycloaddition of diazetidinone structure. SEC analysis of the sonicated polymers showed complete cleavage (M_n of the post-sonication material was less than half of that of the pre-sonication polymer).

Comparison of the ^{1}H NMR spectra of pre- and post-sonication polymer $\mathbf{1}_{\mathbf{N}\text{-}\textbf{\textit{trans}}}$ showed that retro-[2+2] cycloaddition of diazetidinone structure happened upon the pushing force with rotaxane actuator to form isocyanate and imine species. It is evidenced by clear formation of hemiaminal species (see section 5.7), which was generated from hydrolysis of isocyanate followed by reaction with formaldehyde which came from decomposion of THF during the sonication, along with small molecule imine while the intact diazetidinone structure decreased in relative intensity. Aromatic peaks (*g, h*) confirmed to be hemiaminal polymer **S25** after comparing with peaks (*s, t*) in reference **S27** while amine polymer **S24** can be barely observed according to reference **S26**. Also, we can clearly find peaks (*i-p*) of imine $\mathbf{6}_{\mathbf{N}}$ in the concentrated methanol washings.

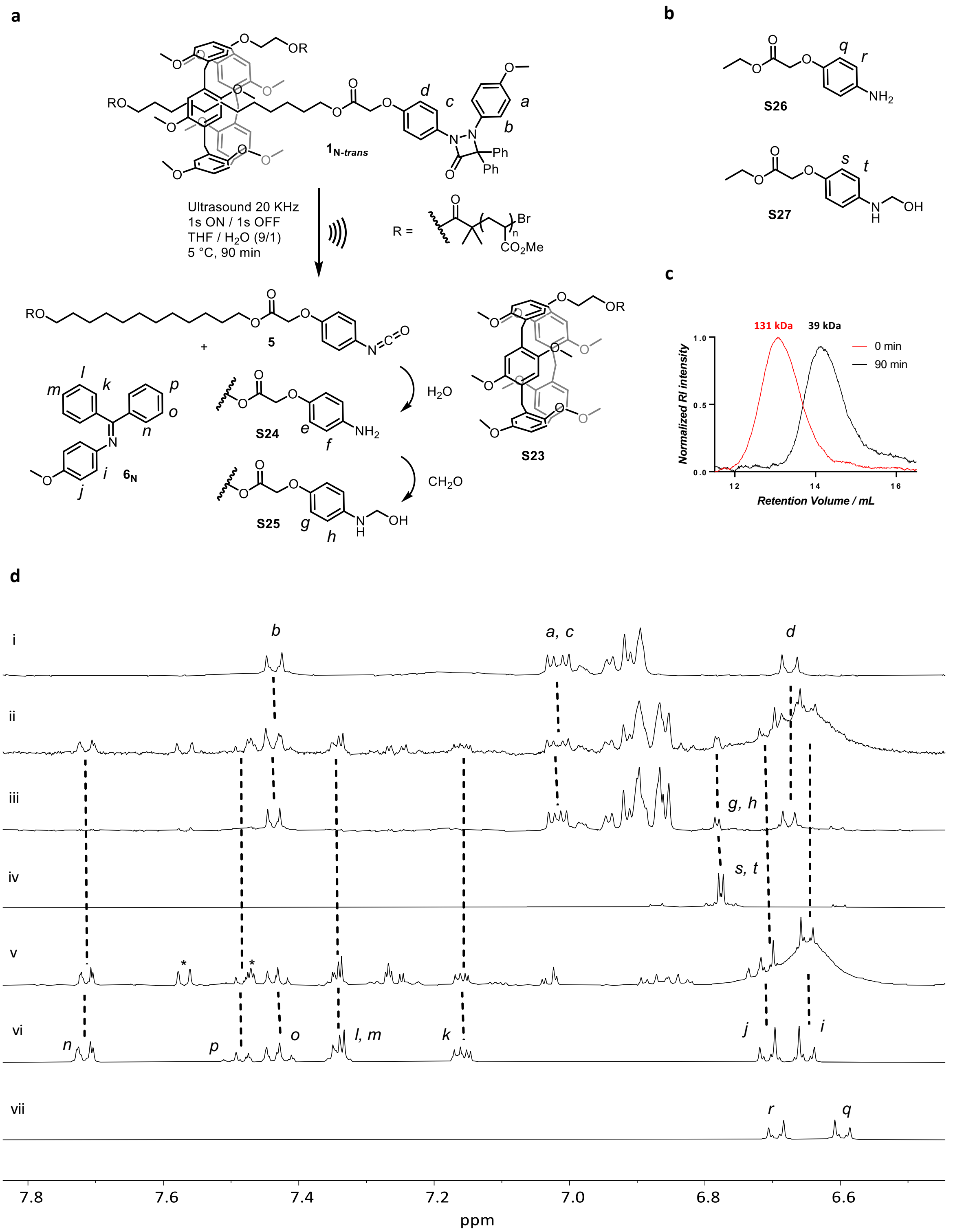

Figure S5. Sonication (run 1) of polymer $\mathbf{1}_{\mathbf{N}\text{-}\boldsymbol{trans}}$ in THF/H_2O (9/1). Sonication of polymer $\mathbf{1}_{\mathbf{N}\text{-}\boldsymbol{trans}}$ affords polymer fragments **S25** and $\mathbf{6}_{\mathbf{N}}$ (a). Reference compound **S26** and **S27** (b). SEC traces of polymer $\mathbf{1}_{\mathbf{N}\text{-}\boldsymbol{trans}}$ (c) with M_n values before (red) and after (black) sonication. Partial ^{1}H NMR (500 MHz, Acetone-d_6, 298 K) spectra comparison (d) of the pre-sonication polymer $\mathbf{1}_{\mathbf{N}\text{-}\boldsymbol{trans}}$ (i), post-sonication polymer before being washed with methanol (ii), post-sonication polymer after being washed with methanol (iii), reference compound **S27** (iv), concentrated methanol washings (v), reference compound $\mathbf{6}_{\mathbf{N}}$ (vi) and reference compound **S26** (vii). * peaks from contaminants, see section 5.8 for details.

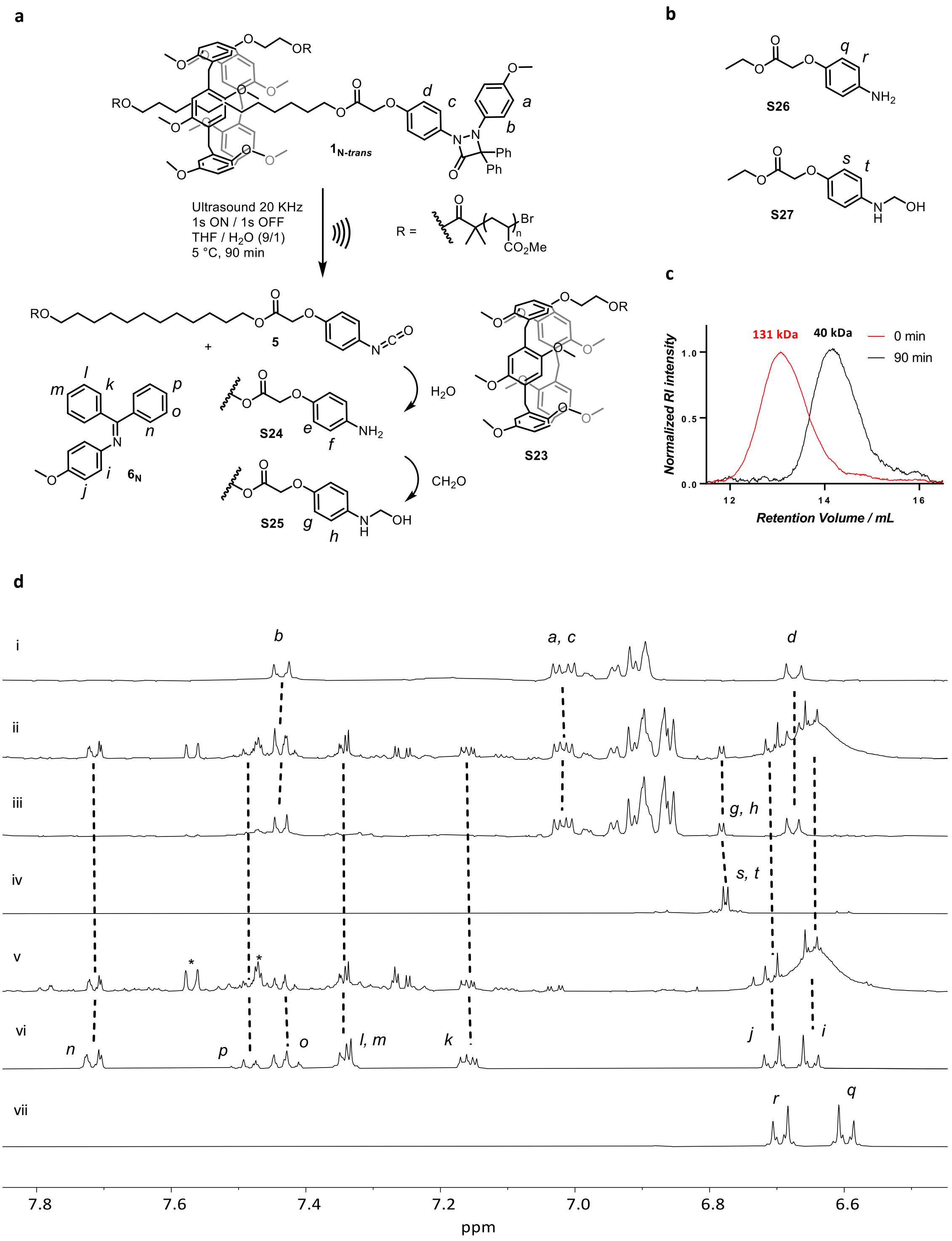

Figure S6. Sonication (run 2) of polymer $\mathbf{1}_{\mathbf{N}\text{-}\boldsymbol{trans}}$ in THF/H_2O (9/1). Sonication of polymer $\mathbf{1}_{\mathbf{N}\text{-}\boldsymbol{trans}}$ affords polymer fragments **S25** and $\mathbf{6}_\mathbf{N}$ (a). Reference compound **S26** and **S27** (b). SEC traces of polymer $\mathbf{1}_{\mathbf{N}\text{-}\boldsymbol{trans}}$ (c) with M_n values before (red) and after (black) sonication. Partial ^{1}H NMR (500 MHz, Acetone-d_6, 298 K) spectra comparison (d) of the pre-sonication polymer $\mathbf{1}_{\mathbf{N}\text{-}\boldsymbol{trans}}$ (i), post-sonication polymer before being washed with methanol (ii), post-sonication polymer after being washed with methanol (iii), reference compound **S27** (iv), concentrated methanol washings (v), reference compound $\mathbf{6}_\mathbf{N}$ (vi) and reference compound **S26** (vii). * marks peaks from contaminants, see section 5.8 for details.

5.4 Sonication of Mechanophore Polymer $\mathbf{1}_{\mathbf{C}\text{-}\textit{cis}}$

Sonication of mechanophore polymer $\mathbf{1}_{\mathbf{C}\text{-}\textit{cis}}$, using the methodology described in the general procedure (*Section 5.1*) and with the solvent used being THF/H_2O (9/1), was carried out twice to determine the extent of activation for retro-[2+2] cycloaddition of β-lactam structure. SEC analysis of the sonicated polymers showed complete cleavage (M_n of the post-sonication material was less than half of that of the pre-sonication polymer).

Comparison of the ^{1}H NMR spectra of pre- and post-sonication polymer $\mathbf{1}_{\mathbf{C}\text{-}\textit{cis}}$ showed that retro-[2+2] cycloaddition of β-lactam structure happened upon the pushing force with rotaxane actuator to form imine and ketene species. It is evidenced by clear formation of imine polymer, which was slightly hydrolyzed to aldehyde polymer due to the presence of water, along with diphenylacetic acid generated from diphenylketene trapping water while the intact β-lactam structure decreased in relative intensity. Aromatic peaks (*d-i*) confirmed to be imine polymer after comparing with reference $\mathbf{4}_{\mathbf{C}}$. And, we can clearly find peaks (*h-j*) of diphenylacetic acid in the concentrated methanol washings.

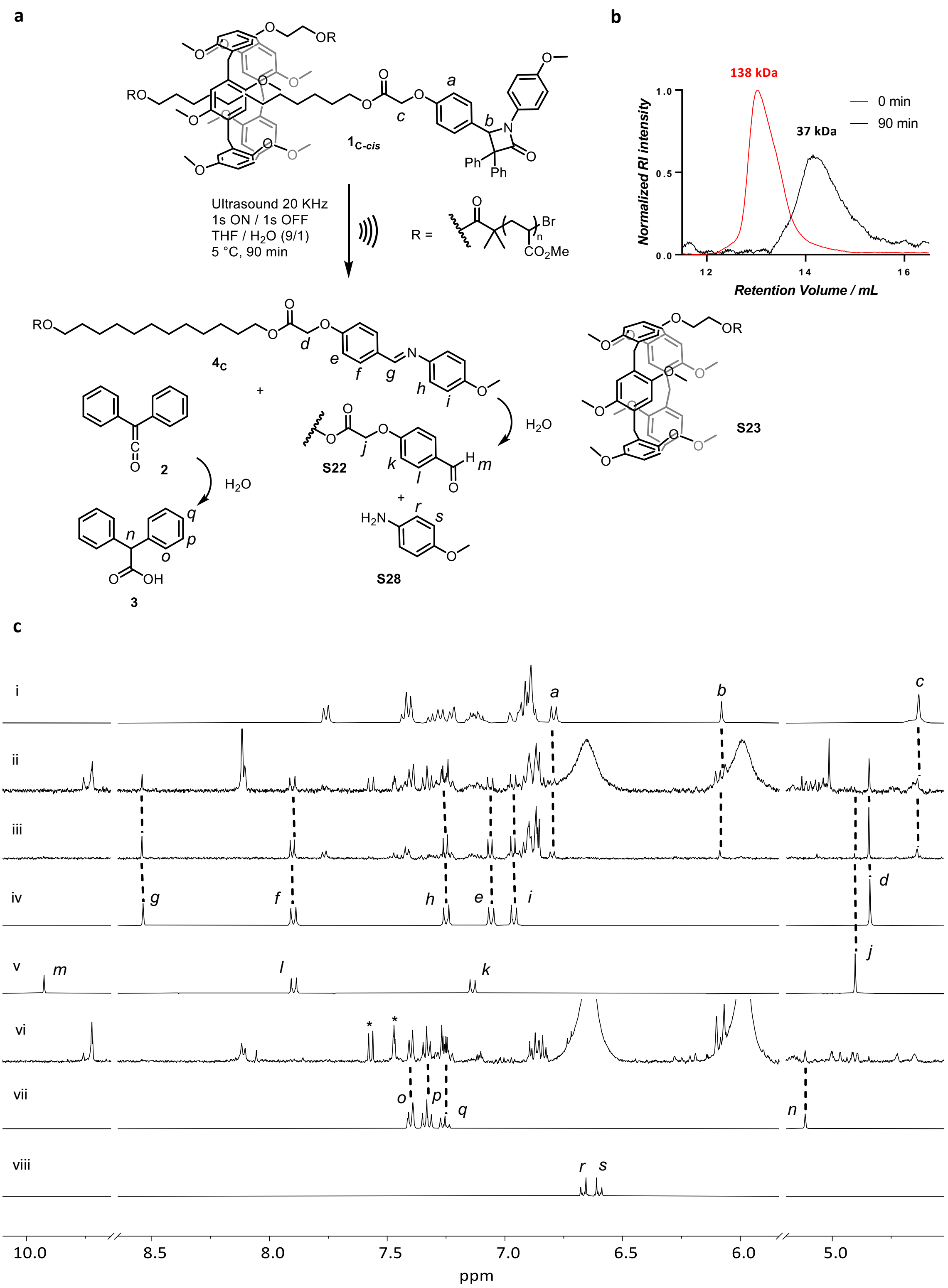

Figure S7. Sonication (run 1) of polymer **1**$_{C\text{-}cis}$ in THF/H_2O (9/1). Sonication of polymer **1**$_{C\text{-}cis}$ affords polymer fragments **4**$_C$, **S22**, **S28** and **3** (a). SEC traces of polymer **1**$_{C\text{-}cis}$ (b) with M_n values before (red) and after (black) sonication. Partial ^{1}H NMR (500 MHz, Acetone-d_6, 298 K) spectra comparison (c) of the pre-sonication polymer **1**$_{C\text{-}cis}$ (i), post-sonication polymer before being washed with methanol (ii), post-sonication polymer after being washed with methanol (iii), reference compound **4**$_C$ (iv), reference compound **S22** (v), concentrated methanol washings (vi), reference compound **3** (vii), and reference compound **S28** (viii). *marks peaks from contaminants, see section 5.8 for details.

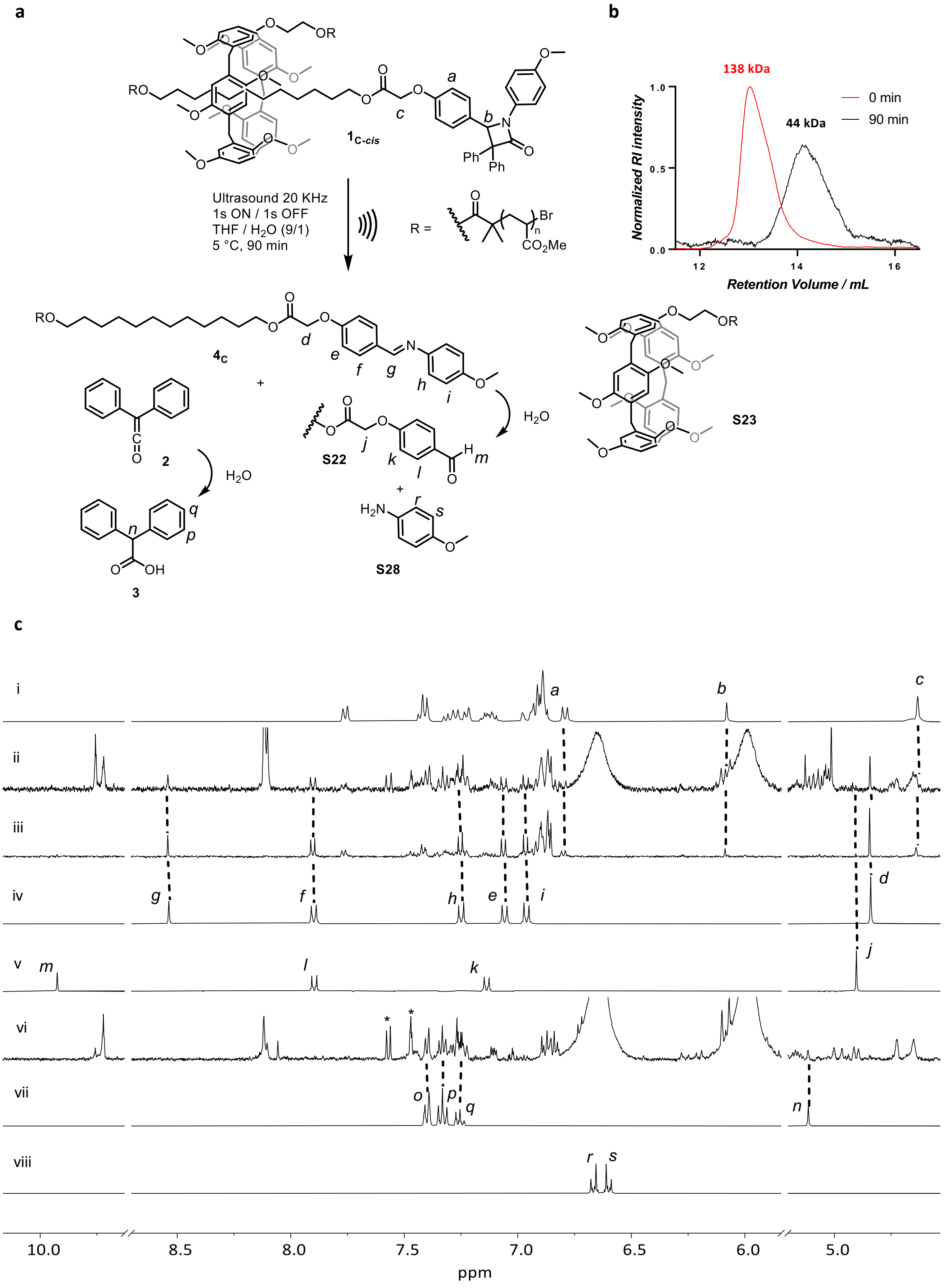

Figure S8. Sonication (run 2) of polymer $\mathbf{1}_{\mathbf{C}\text{-}\textit{cis}}$ in THF/H_2O (9/1). Sonication of polymer $\mathbf{1}_{\mathbf{C}\text{-}\textit{cis}}$ affords polymer fragments $\mathbf{4}_{\mathbf{C}}$, **S22**, **S28** and **3** (a). SEC traces of polymer $\mathbf{1}_{\mathbf{C}\text{-}\textit{cis}}$ (b) with M_n values before (red) and after (black) sonication. Partial ^1H NMR (500 MHz, Acetone-d_6, 298 K) spectra comparison (c) of the pre-sonication polymer $\mathbf{1}_{\mathbf{C}\text{-}\textit{cis}}$ (i), post-sonication polymer before being washed with methanol (ii), post-sonication polymer after being washed with methanol (iii), reference compound $\mathbf{4}_{\mathbf{C}}$ (iv), reference compound **S22** (v), concentrated methanol washings (vi), reference compound **3** (vii), and reference compound **S28** (viii). * marks peaks from contaminants, see section 5.8 for details.

5.5 Sonication of Mechanophore Polymer $\mathbf{1}_{C\text{-}trans}$

Sonication of mechanophore polymer $\mathbf{1}_{C\text{-}trans}$, using the methodology described in the general procedure (*Section 5.1*) and with the solvent used being THF/H_2O (9/1), was carried out twice to determine the extent of activation for retro-[2+2] cycloaddition of β-lactam structure. SEC analysis of the sonicated polymers showed complete cleavage (M_n of the post-sonication material was less than half of that of the pre-sonication polymer).

Comparison of the ^{1}H NMR spectra of pre- and post-sonication polymer $\mathbf{1}_{C\text{-}trans}$ showed that retro-[2+2] cycloaddition of β-lactam structure happened upon the pushing force with rotaxane actuator to form isocyanate and ethylene species. It is evidenced by clear formation of hemiaminal species (see section 5.7), which was generated from hydrolysis of isocyanate followed by reaction with formaldehyde which came from decomposion of THF during the sonication, along with small molecule triphenylethylene with fluorescent and AIE properties (see below) while the intact β-lactam structure decreased in relative intensity. Aromatic peaks (*g, h*) confirmed to be hemiaminal polymer **S25** after comparing with peaks (*m, n*) in reference **S27** while amine polymer **S24** can be barely observed according to reference **S26**. Also, we can clearly find peaks (*h-j*) of triphenylethylene $\mathbf{6}_{C}$ in the concentrated methanol washings.

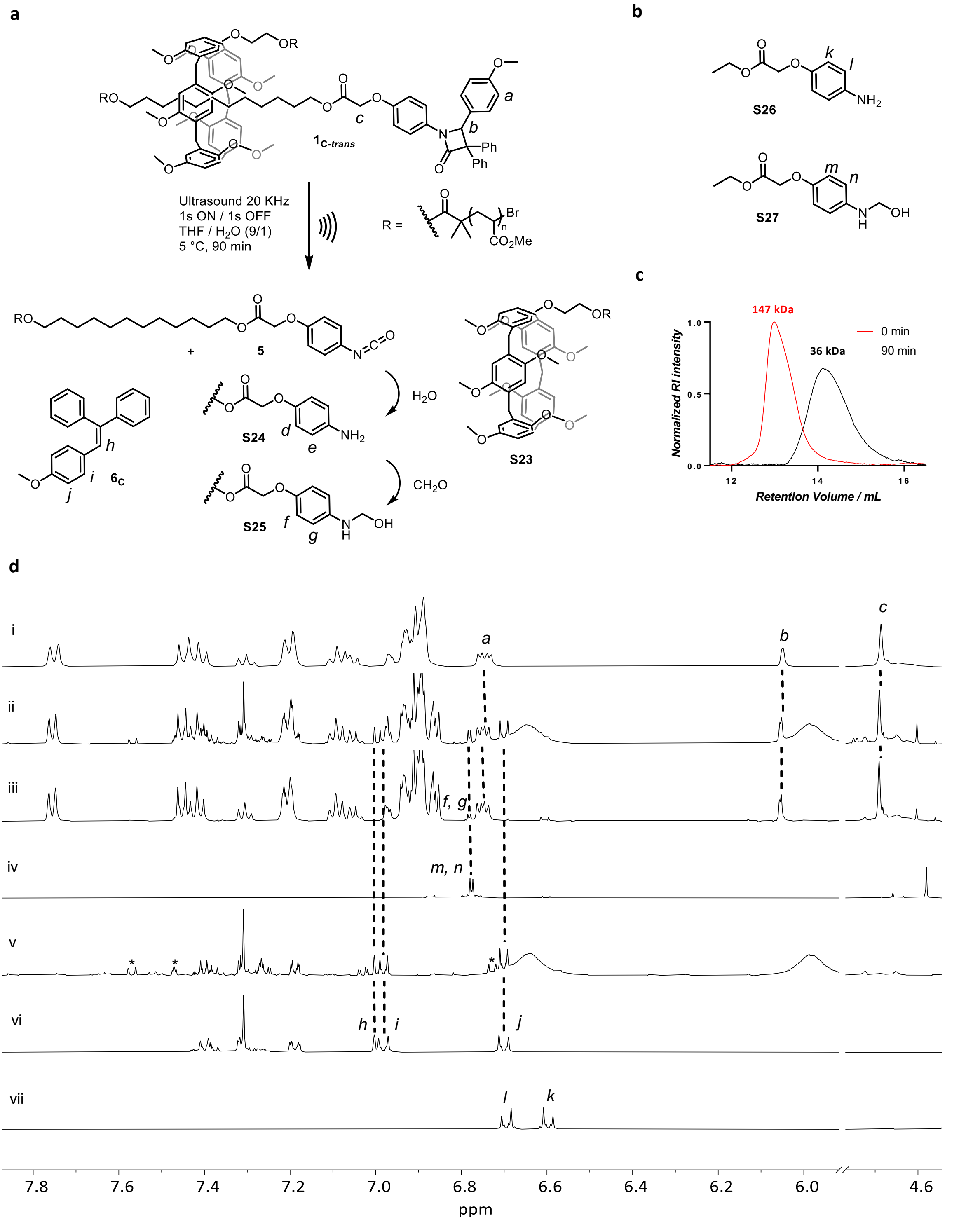

Figure S9. Sonication (run 1) of polymer $\mathbf{1}_{\mathbf{C}\text{-}\boldsymbol{trans}}$ in THF/H_2O (9/1). Sonication of polymer $\mathbf{1}_{\mathbf{C}\text{-}\boldsymbol{trans}}$ affords polymer fragments **S25** and $\mathbf{6}_{\mathbf{C}}$ (a). Reference compound **S26** and **S27** (b). SEC traces of polymer $\mathbf{1}_{\mathbf{C}\text{-}\boldsymbol{trans}}$ (c) with M_n values before (red) and after (black) sonication. Partial ^{1}H NMR (500 MHz, Acetone-d_6, 298 K) spectra comparison (d) of the pre-sonication polymer $\mathbf{1}_{\mathbf{C}\text{-}\boldsymbol{trans}}$ (i), post-sonication polymer before being washed with methanol (ii), post-sonication polymer after being washed with methanol (iii), reference compound **S27** (iv), concentrated methanol washings (v), reference compound $\mathbf{6}_{\mathbf{C}}$ (vi) and reference compound **S26** (vii). * marks peaks from contaminants, see section 5.8 for details.

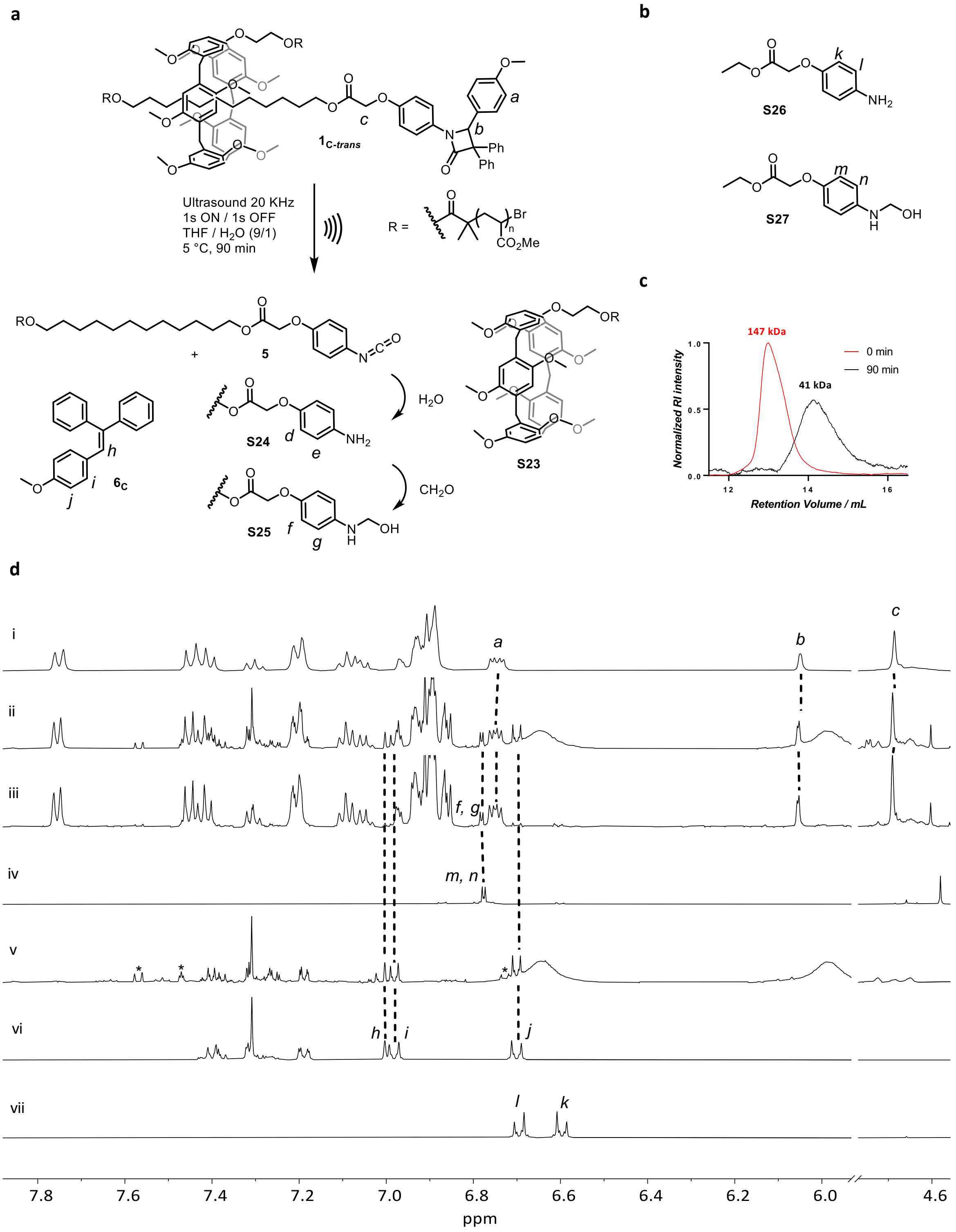

Figure S10. Sonication (run 2) of polymer $\mathbf{1}_{\text{C-}trans}$ in THF/H_2O (9/1). Sonication of polymer $\mathbf{1}_{\text{C-}trans}$ affords polymer fragments **S25** and $\mathbf{6}_{\text{C}}$ (a). Reference compound **S26** and **S27** (b). SEC traces of polymer $\mathbf{1}_{\text{C-}trans}$ (c) with M_n values before (red) and after (black) sonication. Partial ^{1}H NMR (500 MHz, Acetone-d_6, 298 K) spectra comparison (d) of the pre-sonication polymer $\mathbf{1}_{\text{C-}trans}$ (i), post-sonication polymer before being washed with methanol (ii), post-sonication polymer after being washed with methanol (iii), reference compound **S27** (iv), concentrated methanol washings (v), reference compound $\mathbf{6}_{\text{C}}$ (vi) and reference compound **S26** (vii). * marks peaks from contaminants, see section 5.8 for details.

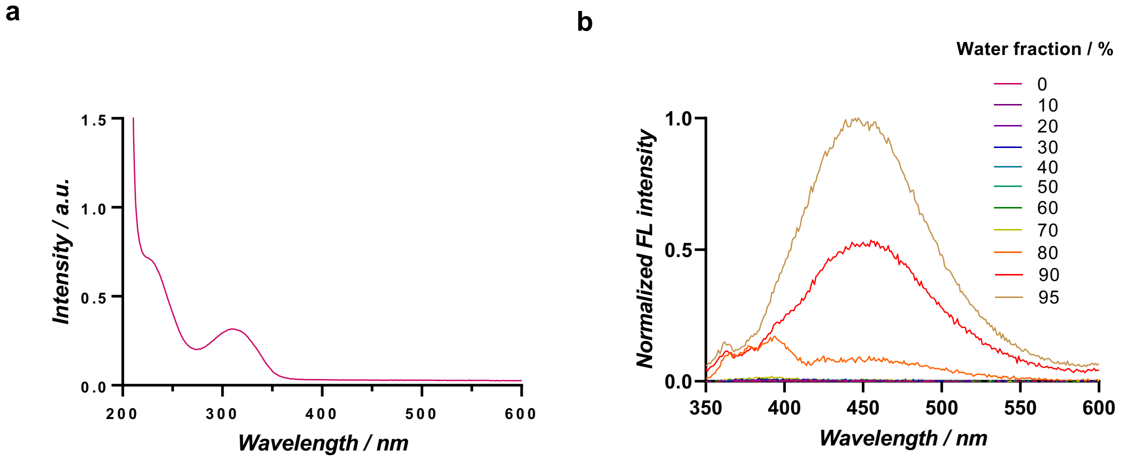

Figure S11. UV spectrum (a) of **6c** in THF (10 µM). And fluorescence spectra (b) of **6c** in water/THF mixtures (400 µM) with different volume fractions of water on excitation at 310 nm.

5.6 Sonication of Control Polymers S18, S19, S20 and S21

Sonication of control polymers **S18**, **S19**, **S20** or **S21**, using the methodology described in the general procedure (*Section 5.1*) and with the solvent used being THF/H_2O (9/1), was carried out to determine the extent of activation for retro-[2+2] cycloaddition of four-membered ring structures. SEC analysis of the sonicated polymers showed complete cleavage (M_n of the post-sonication material was less than half of that of the pre-sonication polymer).

Comparison of the ^{1}H NMR spectra of pre-, post-sonication polymer and reference compounds showed, in all control polymers, retro-[2+2] cycloaddition reaction wasn't observed, which indicates these four-membered ring structures in the control polymers without rotaxane actuator are stable during the sonication.

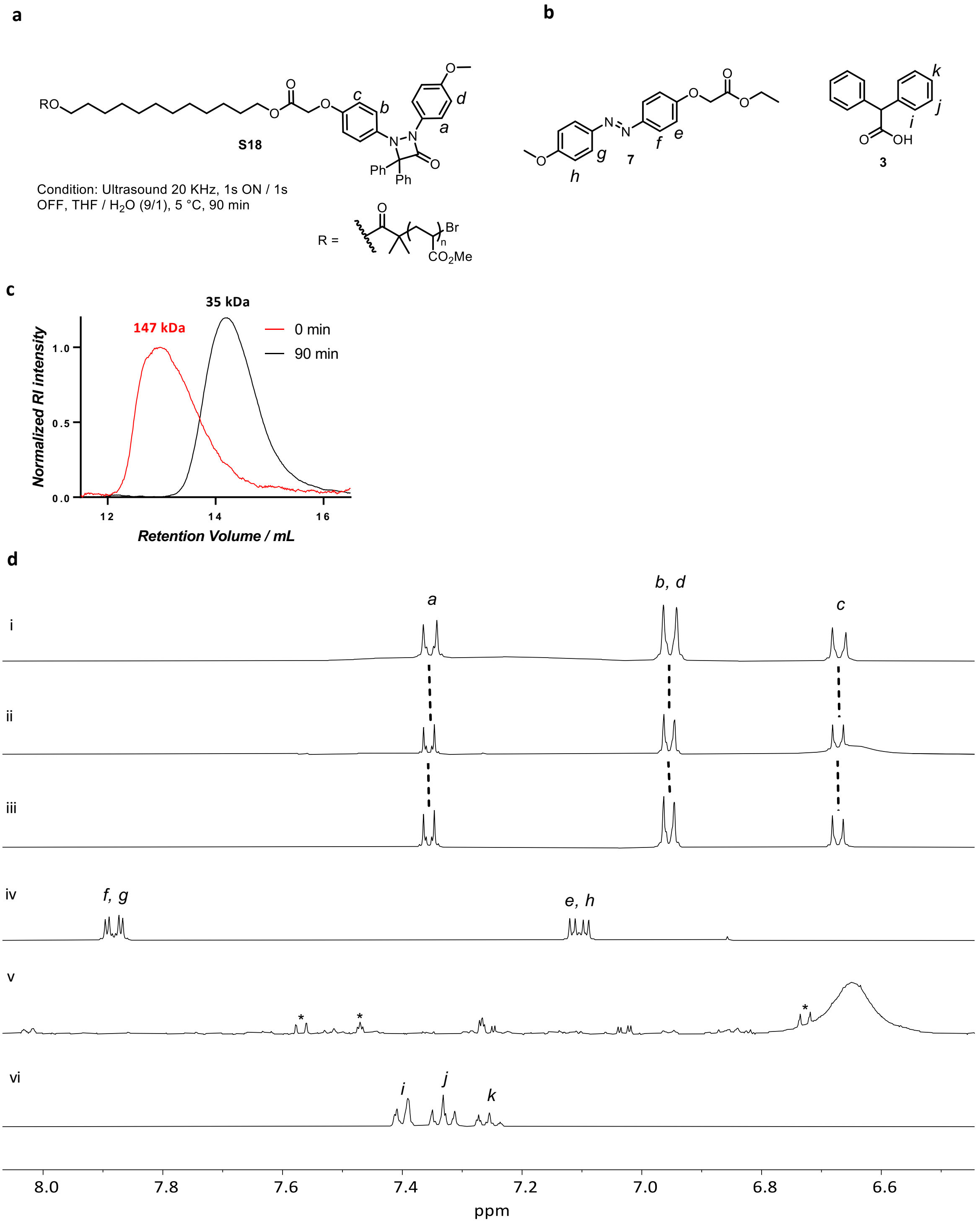

Figure S12. Sonication of polymer **S18** in THF/H_2O (9/1). Structure of polymer **S18** and condition of sonication (a). Reference species **7** and **3** (b). SEC traces of polymer **S18** (c) with M_n values before (red) and after (black) sonication. Partial ^1H NMR (500 MHz, Acetone-d_6, 298 K) spectra comparison (d) of the pre-sonication polymer **S18** (i), post-sonication polymer before being washed with methanol (ii), post-sonication polymer after being washed with methanol (iii), reference compound **7** (iv), concentrated methanol washings (v), and reference compound **3** (vi). * marks peaks from contaminants, see section 5.8 for details.

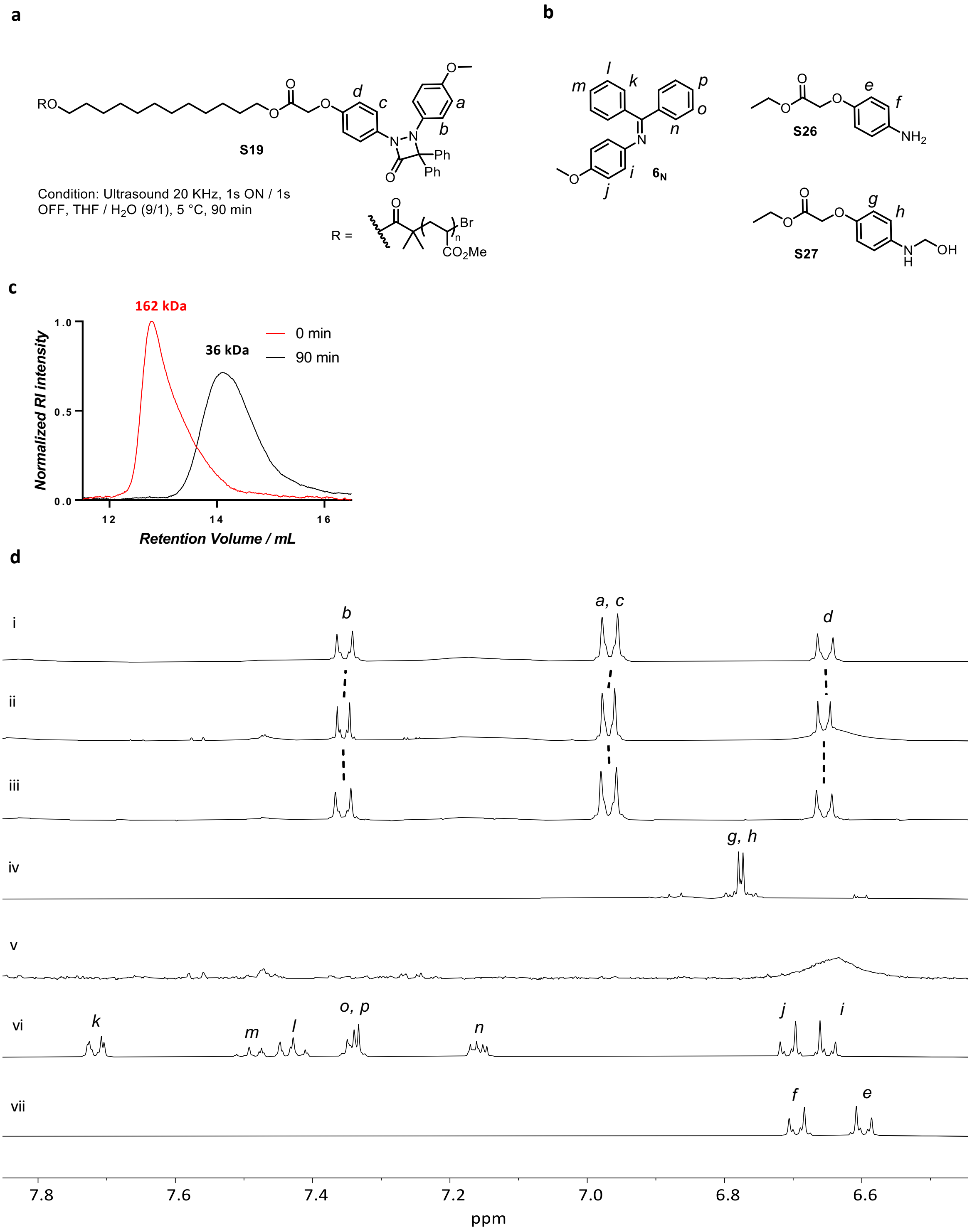

Figure S13. Sonication of polymer **S19** in THF/H_2O (9/1). Structure of polymer **S19** and condition of sonication (a). Reference species **6$_N$**, **S26** and **S27** (b). SEC traces of polymer **S19** (c) with M_n values before (red) and after (black) sonication. Partial ^{1}H NMR (500 MHz, Acetone-d_6, 298 K) spectra comparison (d) of the pre-sonication polymer **S19** (i), post-sonication polymer before being washed with methanol (ii), post-sonication polymer after being washed with methanol (iii), reference compound **S27** (iv), concentrated methanol washings (v), reference compound **6$_N$** (vi), and reference compound **S26** (vii).

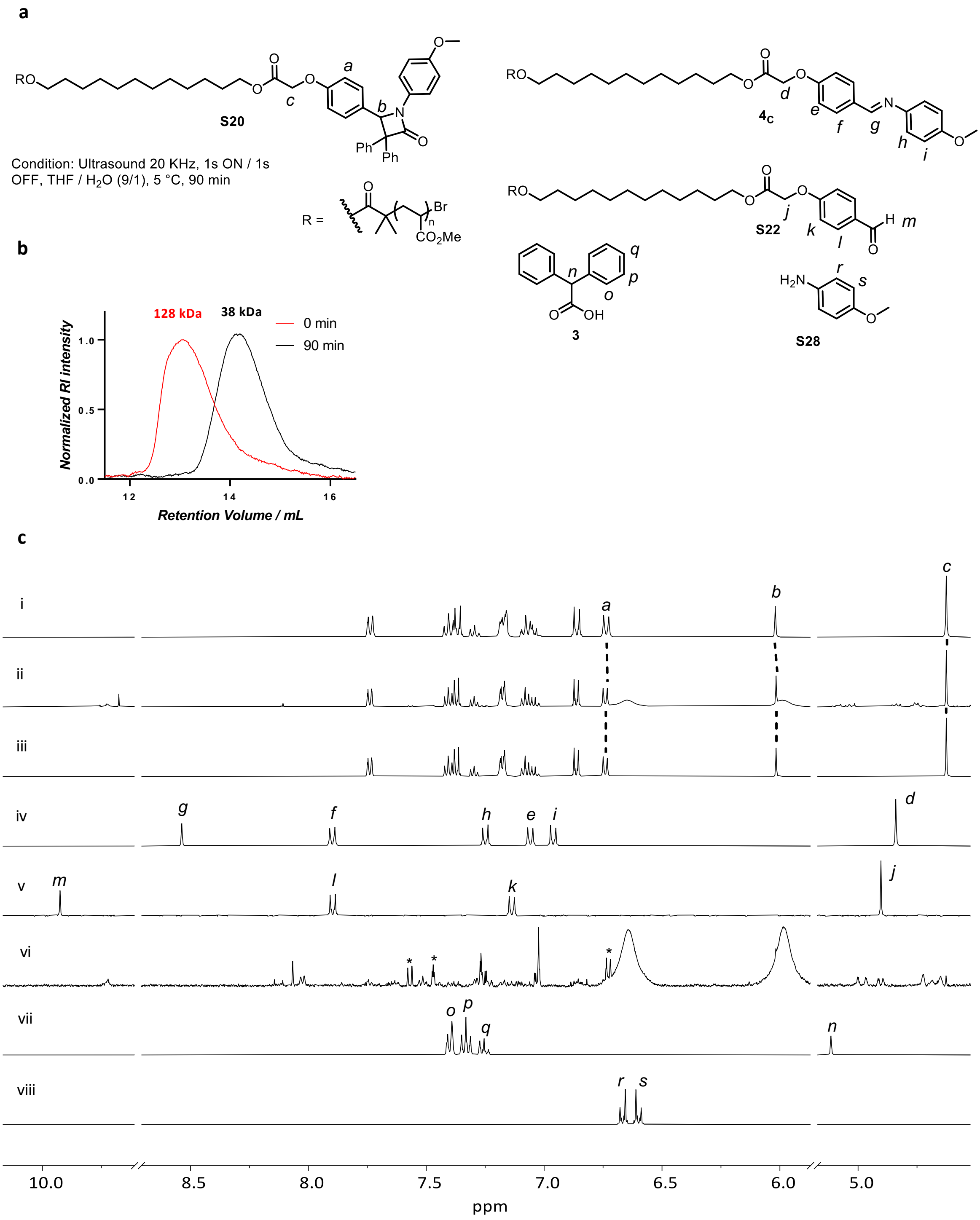

Figure S14. Sonication of polymer **S20** in THF/H_2O (9/1). Structure of polymer **S20** and condition of sonication (a). Reference species **4**$_\mathbf{C}$, **S22**, **3** and **S28** (b). SEC traces of polymer **S20** (c) with M_n values before (red) and after (black) sonication. Partial ^{1}H NMR (500 MHz, Acetone-d_6, 298 K) spectra comparison (d) of the pre-sonication polymer **S20** (i), post-sonication polymer before being washed with methanol (ii), post-sonication polymer after being washed with methanol (iii), reference compound **4**$_\mathbf{C}$ (iv), reference compound **S22** (v), concentrated methanol washings (vi), reference compound **3** (vii), and reference compound **S28** (viii). * marks peaks from contaminants, see section 5.8 for details.

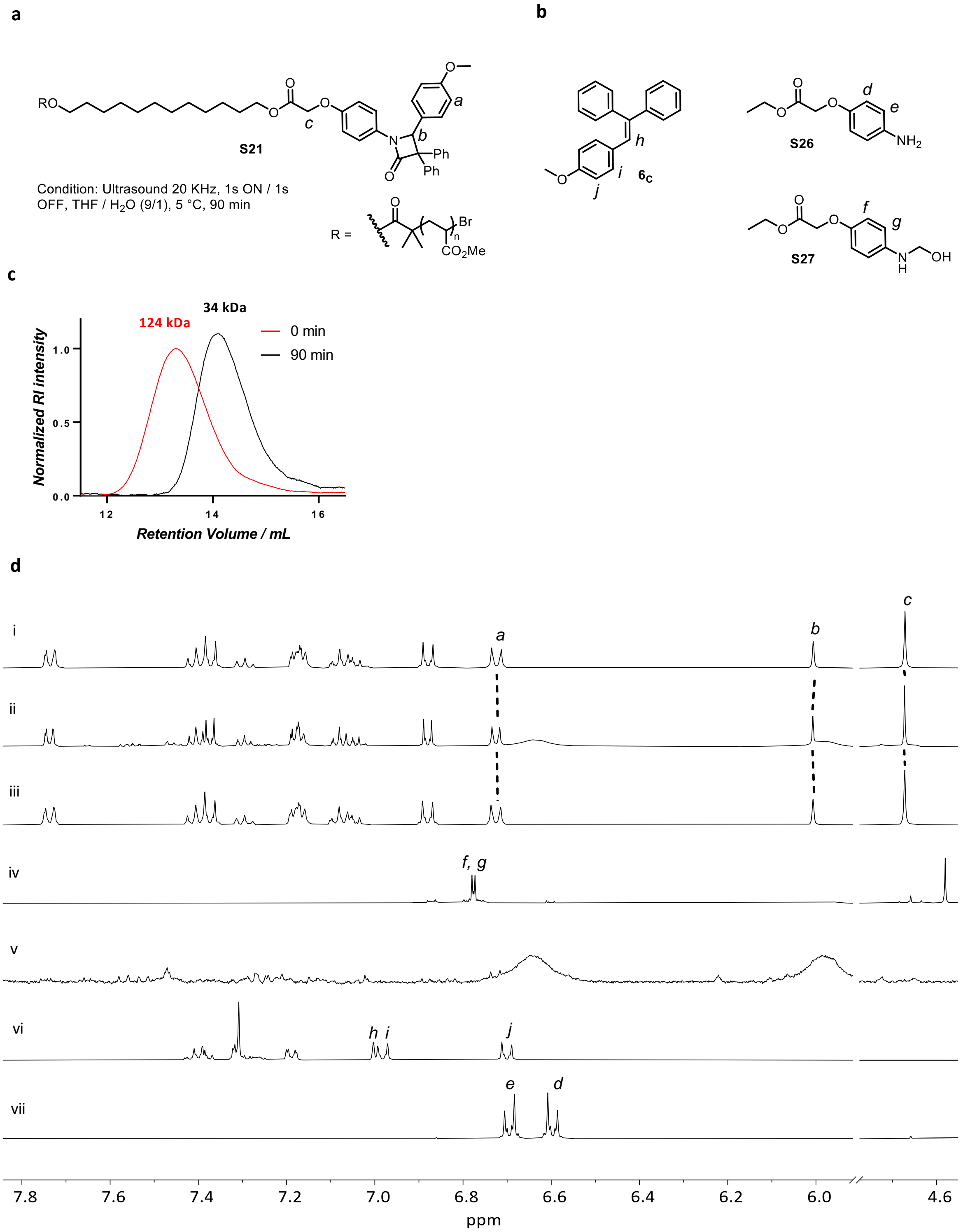

Figure S15. Sonication of polymer **S21** in THF/H_2O (9/1). Structure of polymer **S21** and condition of sonication (a). Reference species **6$_C$**, **S26** and **S27** (b). SEC traces of polymer **S21** (c) with M_n values before (red) and after (black) sonication. Partial ^{1}H NMR (500 MHz, Acetone-d_6, 298 K) spectra comparison (d) of the pre-sonication polymer **S21** (i), post-sonication polymer before being washed with methanol (ii), post-sonication polymer after being washed with methanol (iii), reference compound **S27** (iv), concentrated methanol washings (v), and reference compound **6$_C$** (vi), and reference compound **S26** (vii).

5.7 Determination of the Degradation Product of S26 in the Sonication Experiment

Sonication of compound **S26**, using the methodology described in the general procedure (*Section 5.1*) and with the solvent used being THF (1 mg in 20 mL THF), was carried out to determine degradation product from **S26** after sonication. Also, a control experiment, in which only solvent was used, was conducted.

Comparison of the ^{1}H NMR spectra (**Figure S16**b) of pre- and post-sonication reference compound **S26** showed that it was fully degradated to a new species after sonication, in which all peaks from **S26** were slightly shifted. Apart from peaks in control experiment, two new peaks (*g, h*) were found, which indicates this new species was generated from **S26** reacting with formaldehyde which was normally found in THF after sonication. ^{13}C NMR (**Figure S16**c) and MS spectra (**Figure S17**) also confirmed the structure of **S27**. This hemiaminal structure is relatively stable and can be generated from mixture of aromatic amine and paraformaldehyde at high temperature according to the publication.[9] MS spectrommetry showed two main signals belonging to **S27** after loss of formaldehyde or water molecule respectively.

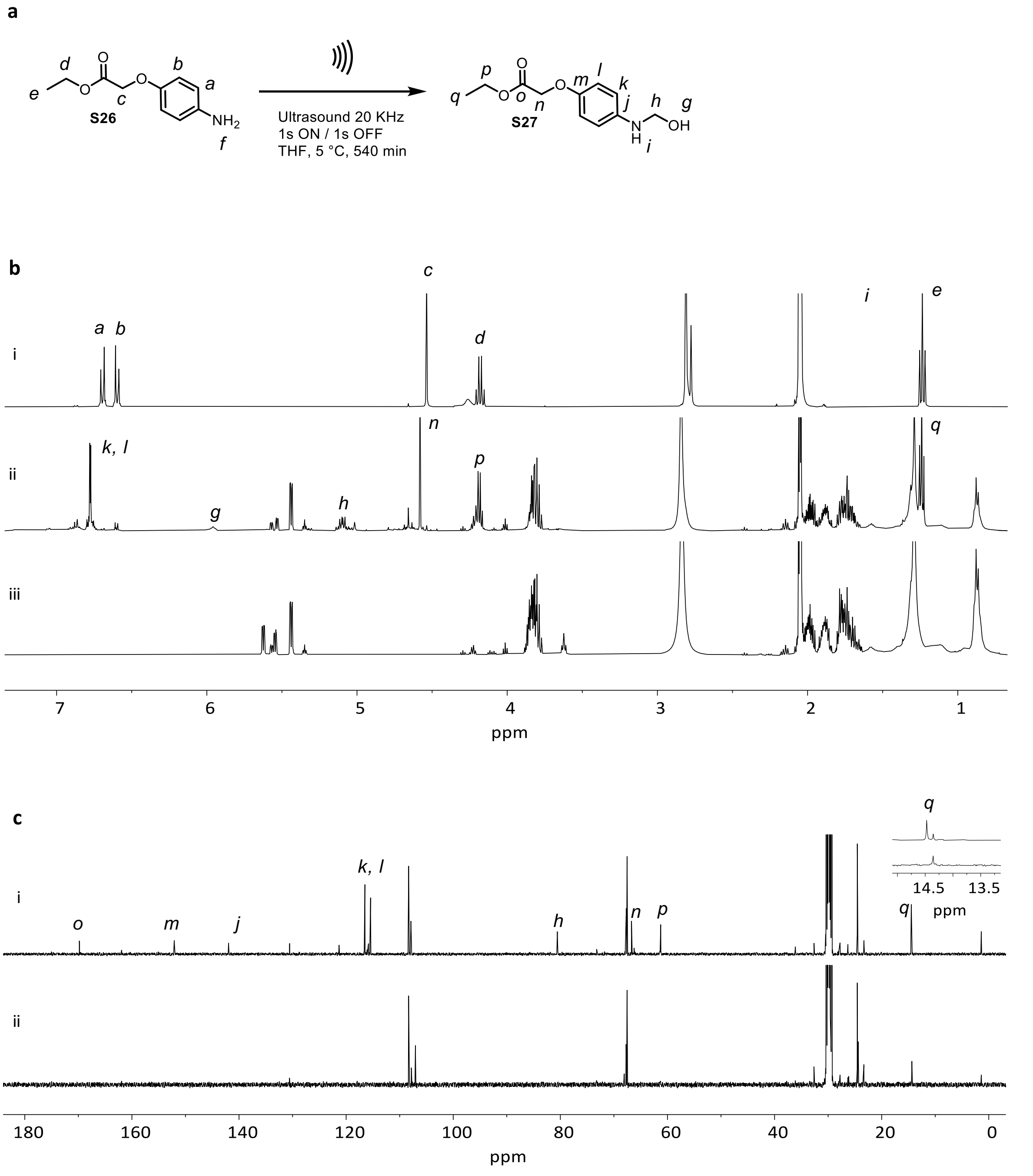

Figure S16. Sonication of compound **S26** in THF. Sonication of **S26** affords a new species **S27** (a). ^{1}H NMR (500 MHz, Acetone-d_6, 298 K) spectra comparison (b) of **S26** (i), **S27** (ii), and control experiment (iii). ^{13}C NMR (133 MHz, Acetone-d_6, 298 K) spectra comparison (c) of **S27** (i), and control experiment (ii).

a

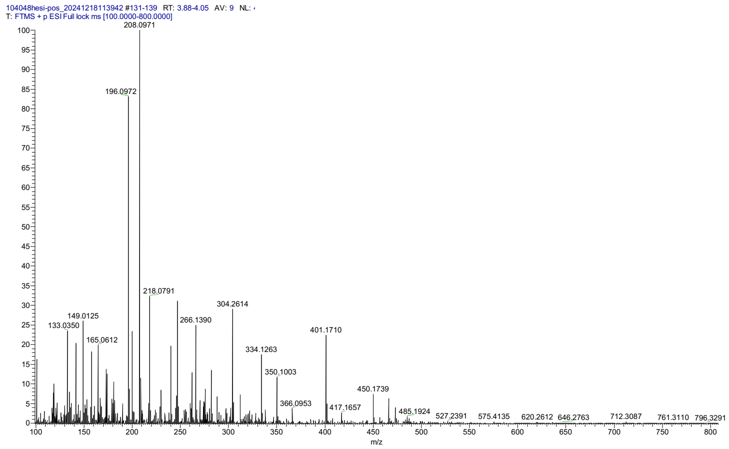

b

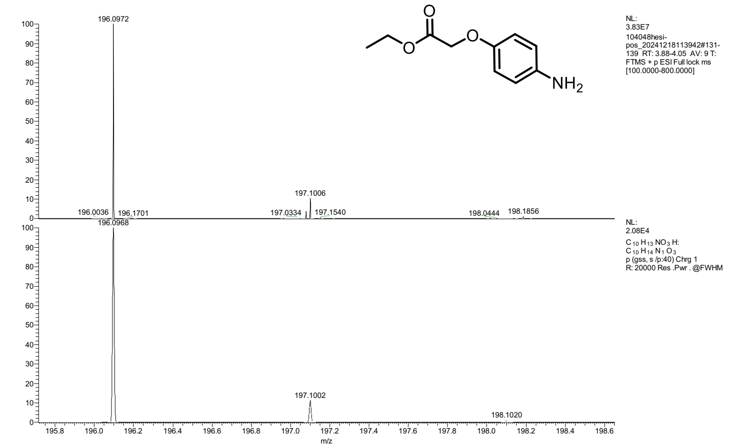

c

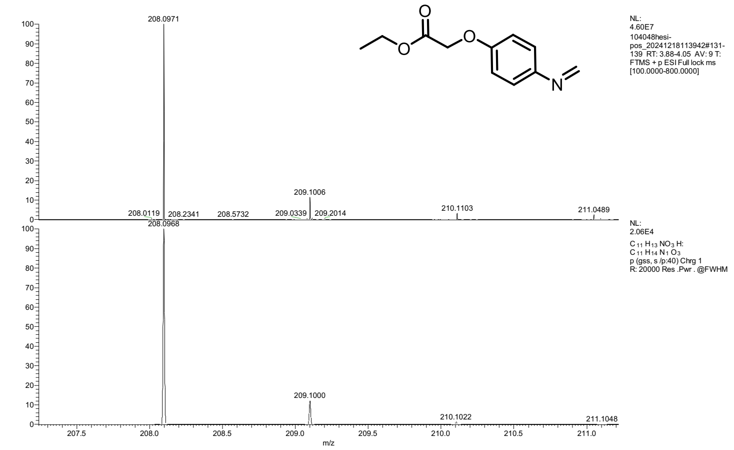

Figure S17. HRMS-ESI spectra of **S27**, including scan of m/z from 100 to 800 (a) and isotopic distribution of **S27** after loss of formaldehyde (b) or water (c). Top: Measured isotopic distribution. Bottom: Simulated isotopic distribution.

5.8 Analysis of Contaminants in Post-Sonication MeOH Extracts

Here we use MeOH extract from post-sonication polymer **1**$_{\mathbf{N}\text{-}\boldsymbol{cis}}$ (run 1) as an example to analyze the external contaminants. We identified the origin of the contaminant peaks found in the MeOH extract of various samples. They mainly come from the degradation of THF during the sonication, and from contaminants present in the single-use 20 mL plastic syringe (presumably from the coating on the syringe and/or the needle) and from the methanol itself.

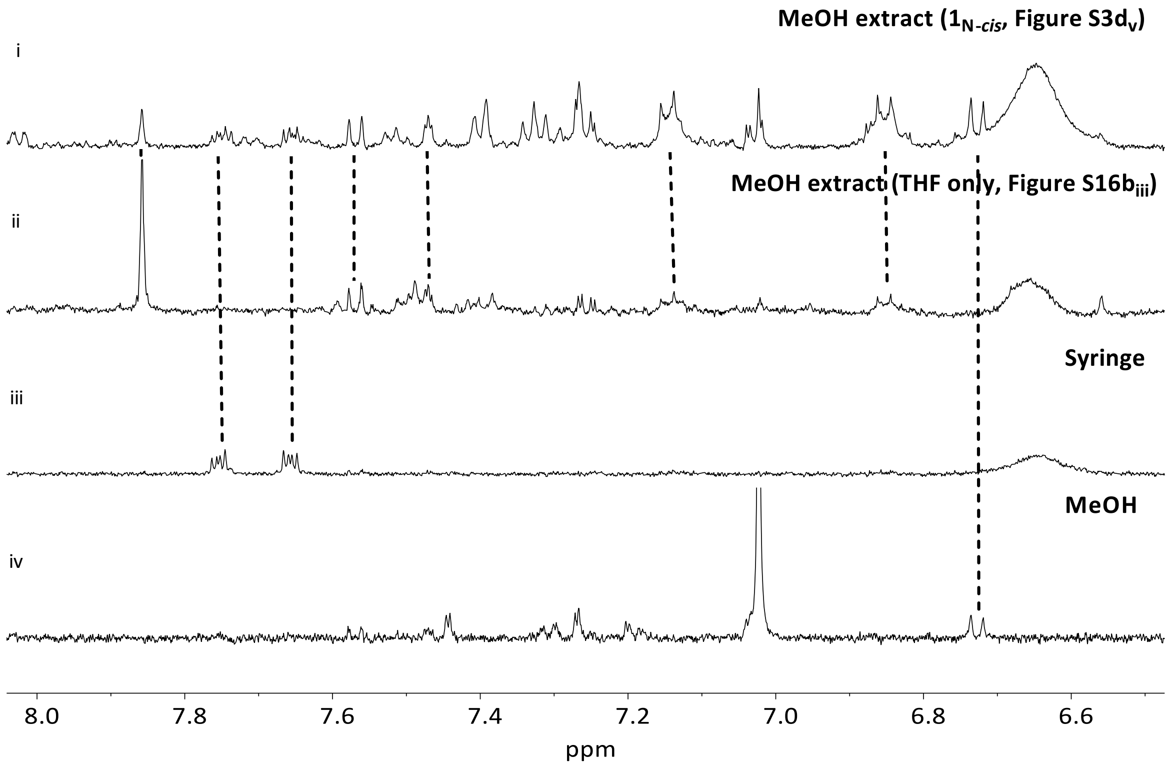

Figure S18. Partial ^{1}H NMR (500 MHz, Acetone-d_6, 298 K) spectra comparison of MeOH extract from post-sonication of polymer **1**$_{\mathbf{N}\text{-}\boldsymbol{cis}}$ (i) and control experiment in which 20 mL of THF were sonicated in the conditions described in section 5.1, ii), MeCN extract from syringe (20 mL of MeCN were drawn and left in the syringe for 5min before being injected in a flask. The operation was repeated another 2 times before evaporating the solvent, iii), and residue after evaporation of 500 mL of MeOH (iv).

6 Calculation of Extent of Mechanophore Activation

6.1 Calculations for Polymer $1_{N\text{-}cis}$

Here we use sonication (run 1) of polymer $\mathbf{1}_{\mathbf{N}\text{-}\boldsymbol{cis}}$ as an example of how we calculated the extent of activation for retro-[2+2] cycloaddition of diazetidinone.

a

b

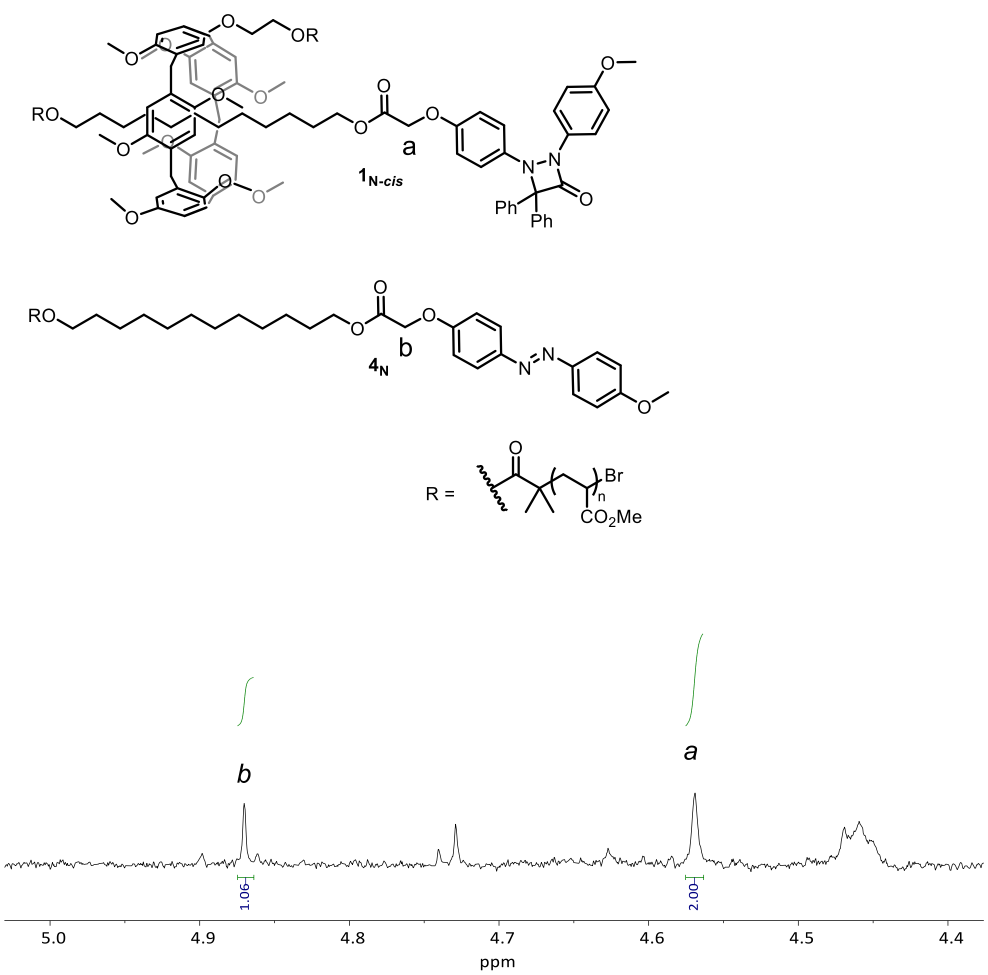

Figure S19. Polymer $\mathbf{1}_{\mathbf{N}\text{-}\boldsymbol{cis}}$ affords polymer fragment $\mathbf{4}_{\mathbf{N}}$ after activation (a). And, partial ^{1}H NMR (500 MHz, Acetone-d_6, 298 K) spectra of post-sonication polymer $\mathbf{1}_{\mathbf{N}\text{-}\boldsymbol{cis}}$ after being washed with methanol (b).

Here, we use values determined from integration of peaks a (I_a) and b (I_b) in the ^{1}H NMR spectrum of post-sonication polymer $\mathbf{1}_{\mathbf{N}\text{-}\boldsymbol{cis}}$ after being washed with methanol.

The extent of retro-[2+2] cycloaddition (C_r) having occurred during the sonication was determined by the formula below:

$$C_r = \frac{I_b}{I_a + I_b} \times 100\%$$

In this case, $\boldsymbol{C_r}$ **= 35%** [(1.06 / (2.00 + 1.06)) × 100%].

6.2 Calculations for Polymer $\mathbf{1}_{\mathbf{N}\text{-}\boldsymbol{trans}}$

Here we use sonication (run 1) of polymer $\mathbf{1}_{\mathbf{N}\text{-}\boldsymbol{trans}}$ as an example of how we calculated the extent of activation for retro-[2+2] cycloaddition of diazetidinone.

a

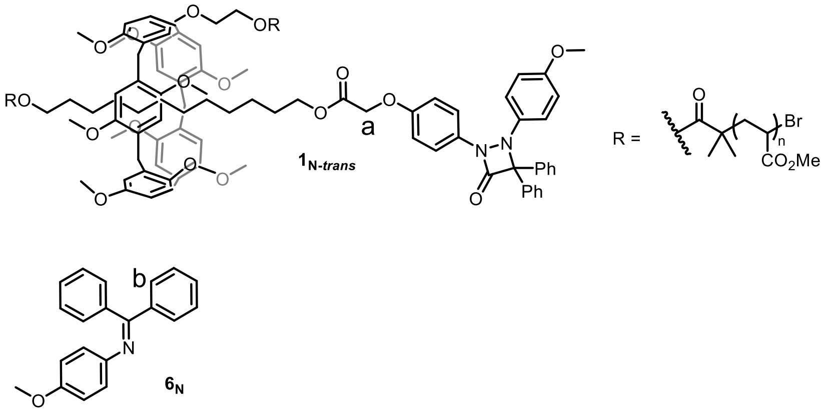

b

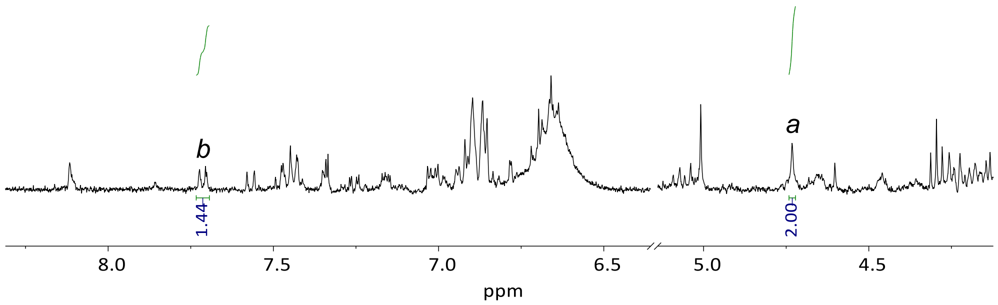

Figure S20. Polymer $\mathbf{1}_{\mathbf{N}\text{-}\boldsymbol{trans}}$ affords compound $\mathbf{6_N}$ after activation (a). And, partial ^{1}H NMR (500 MHz, Acetone-d_6, 298 K) spectra of post-sonication polymer $\mathbf{1}_{\mathbf{N}\text{-}\boldsymbol{trans}}$ before being washed with methanol (b).

Here, we use values determined from integration of peaks a (I_a) and b (I_b) in the ^{1}H NMR spectrum of post-sonication polymer $\mathbf{1}_{\mathbf{N}\text{-}\boldsymbol{trans}}$ before being washed with methanol.

The extent of retro-[2+2] cycloaddition (C_r) having occurred during the sonication was determined by the formula below:

$$C_r = \frac{I_b}{I_a + I_b} \times 100\%$$

In this case, $\boldsymbol{C_r}$ **= 42%** [(1.44 / (2.00 + 1.44)) × 100%].

6.3 Calculations for Polymer $\mathbf{1}_{C\text{-}cis}$

Here we use sonication (run 1) of polymer $\mathbf{1}_{C\text{-}cis}$ as an example of how we calculated the extent of activation for retro-[2+2] cycloaddition of β-lactam.

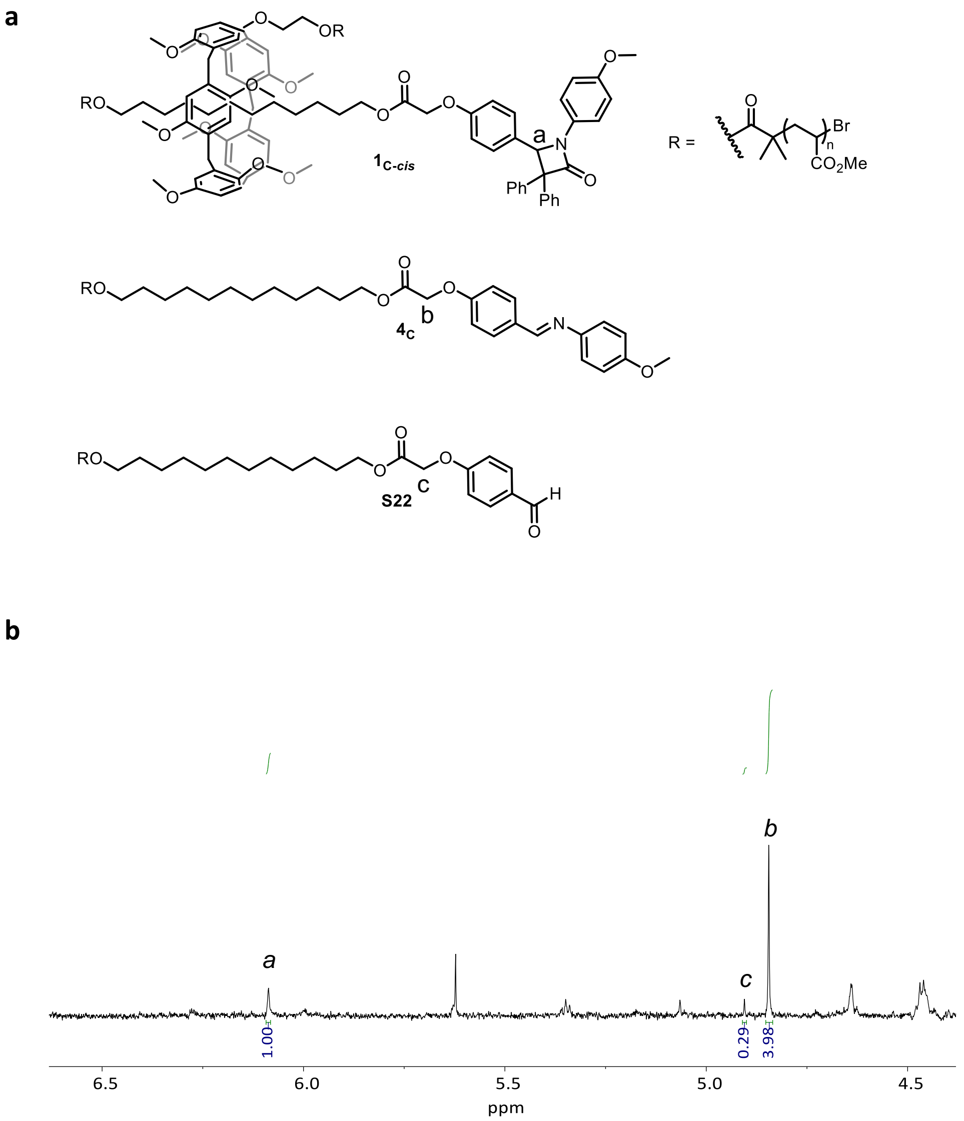

Figure S21. Polymer $\mathbf{1}_{C\text{-}cis}$ affords polymer fragment $\mathbf{4}_{C}$ and **S22** after activation (a). And, partial ^{1}H NMR (500 MHz, Acetone-d_6, 298 K) spectra of post-sonication polymer $\mathbf{1}_{C\text{-}cis}$ after being washed with methanol (b).

Here, we use values determined from integration of peaks a (I_a), b (I_b) and c (I_c) in the ^{1}H NMR spectra of post-sonication polymer $\mathbf{1}_{C\text{-}cis}$ after being washed with methanol.
The extent of retro-[2+2] cycloaddition (C_r) having occurred during the sonication was determined by the formula below:

$$C_r = \frac{I_b + I_c}{2I_a + I_b + I_c} \times 100\%$$

In this case, $\boldsymbol{C_r}$ **= 68%** [(3.98+0.29) / (2*1.00 + 3.98+0.29) × 100%].

6.4 Calculations for Polymer $\mathbf{1}_{\mathbf{C}\text{-}\boldsymbol{trans}}$

Here we use sonication (run 1) of polymer $\mathbf{1}_{\mathbf{C}\text{-}\boldsymbol{trans}}$ as an example of how we calculated the extent of activation for retro-[2+2] cycloaddition of β-lactam.

a

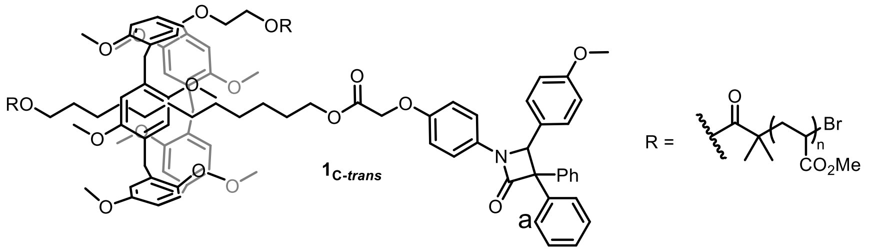

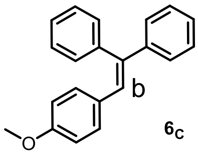

b

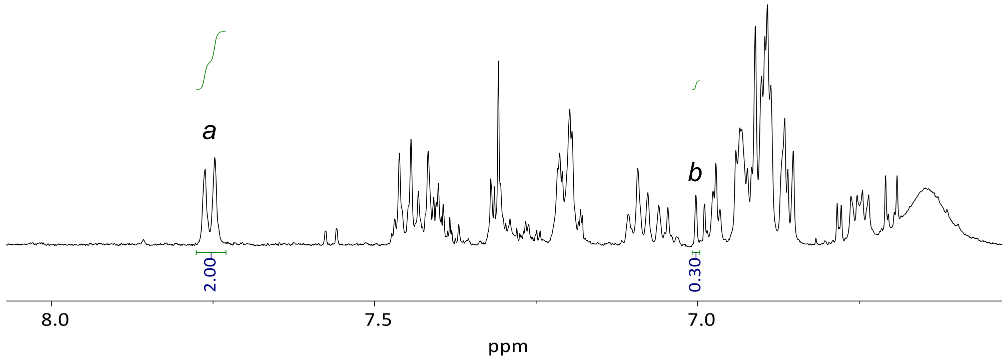

Figure S22. Polymer $\mathbf{1}_{\mathbf{C}\text{-}\boldsymbol{trans}}$ affords compound $\mathbf{6}_{\mathbf{C}}$ after activation (a). And, partial ^{1}H NMR (500 MHz, Acetone-d_6, 298 K) spectra of post-sonication polymer $\mathbf{1}_{\mathbf{C}\text{-}\boldsymbol{trans}}$ before being washed with methanol (b).

Here, we use values determined from integration of peaks a (I_a) and b (I_b) in the ^{1}H NMR spectra of post-sonication polymer $\mathbf{1}_{\mathbf{C}\text{-}\boldsymbol{trans}}$ after being washed with methanol.
The extent of retro-[2+2] cycloaddition (C_r) having occurred during the sonication was determined by the formula below:

$$C_r = \frac{2I_b}{I_a + 2I_b} \times 100\%$$

In this case, $\boldsymbol{C_r}$ **= 23%** [(2*0.30 / (2.00 + 2*0.30)) × 100%].

6.5 Summary of Mechanophores Activated by Sonication

Table S2. Analysis of mechanical activation *via* ultrasound of mechanophore and control polymers in THF/H_2O (9/1).

**No.**	**Polymer**			**Pre-sonication**		**Post-sonication**		**Conversion (%)**		
				***M_n* (kDa)**	***Đ***	***M_n* (kDa)**	***Đ***	**Intact mechanophore[a]**	**Cleavage conversion**	**Average conversion**
**1**	**Mechanophore polymer**	**1**$_{N\text{-}cis}$	**Run 1**	149	1.42	38	1.29	65	35	35
**2**			**Run 2**			36	1.33	65	35	
**3**		**1**$_{N\text{-}trans}$	**Run 1**	131	1.39	39	1.34	58	42	43
**4**			**Run 2**			40	1.30	57	43	
**5**		**1**$_{C\text{-}cis}$	**Run 1**	138	1.18	37	1.28	32	68	69
**6**			**Run 2**			44	1.22	31	69	
**7**		**1**$_{C\text{-}trans}$	**Run 1**	147	1.17	36	1.35	77	23	23
**8**			**Run 2**			41	1.27	77	23	
**9**	**Control polymer**	**S18**	**NA**	147	1.34	35	1.35	NA		0
**10**		**S19**		162	1.29	36	1.40			
**11**		**S20**		128	1.37	38	1.32			
**12**		**S21**		124	1.37	34	1.48			

Notes:

a) This value is given as the remaining percentage that is unaccounted for those that undergo the desired activation.

7 CoGEF Calculations

7.1 General method

CoGEF calculations were performed on GaussianView'6 following Beyer's method.[10] The structure of the mechanophore was built in GaussianView'6. The distance between the terminal methyl groups was constrained and increased by increments with 0.4 Å or 0.5 Å depending on the mechanophore. Each step was run with DFT (B3LYP/6-31G) in vaccum. The relative energy of each intermediate was determined by setting the energy of the initial state at 0 kJ/mol. F_{max} values were determined from the slope of the final 3 points.

7.2 CoGEF of models of $1_{N\text{-}cis}$'

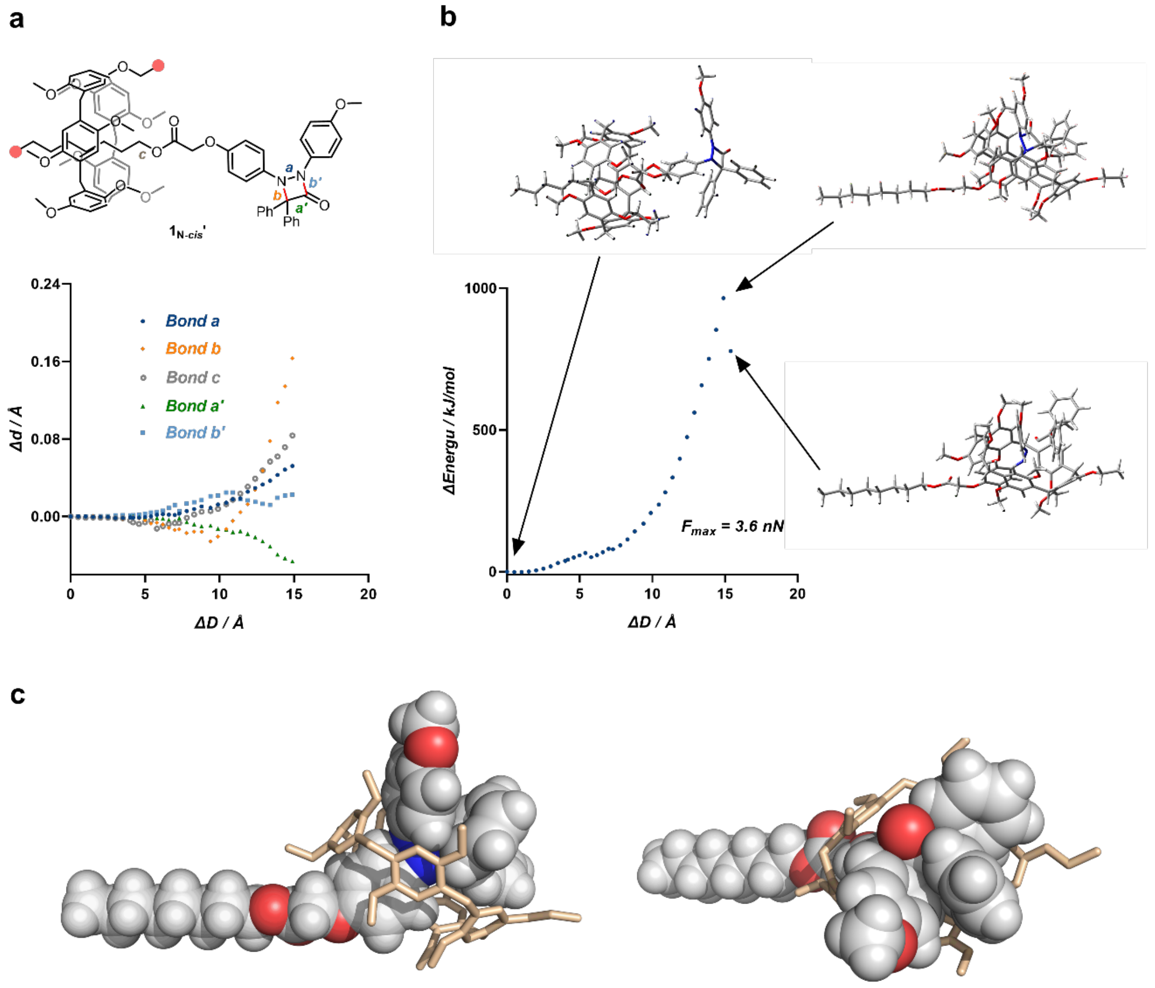

Figure S23. Evolution of bond *a*, *a'*, *b*, *b'*, and *c* (a) and energy (b) upon simulated elongation (CoGEF, DFT B3LYP/6-31G, vac) of a model of diazetidinone structure in **1**$_{N\text{-}cis}$**'** activated by pushing force with rotaxane actuator. Models in the computation indicating key structural parameters. Predicted s*cis*sile bond are shown in red. Anchor atoms are indicated by the pink disks. And side and top views (c) of the model (space-filling for axle part and stick for macrocycle) of **1**$_{N\text{-}cis}$**'** at maximal deformation (E_{max}) reveals the extent of the contact surface between the mechanophore and the macrocycle. Hydrogen atoms omitted for clarity in the tube representation.

7.3 CoGEF of models of $1_{N\text{-}trans}$'

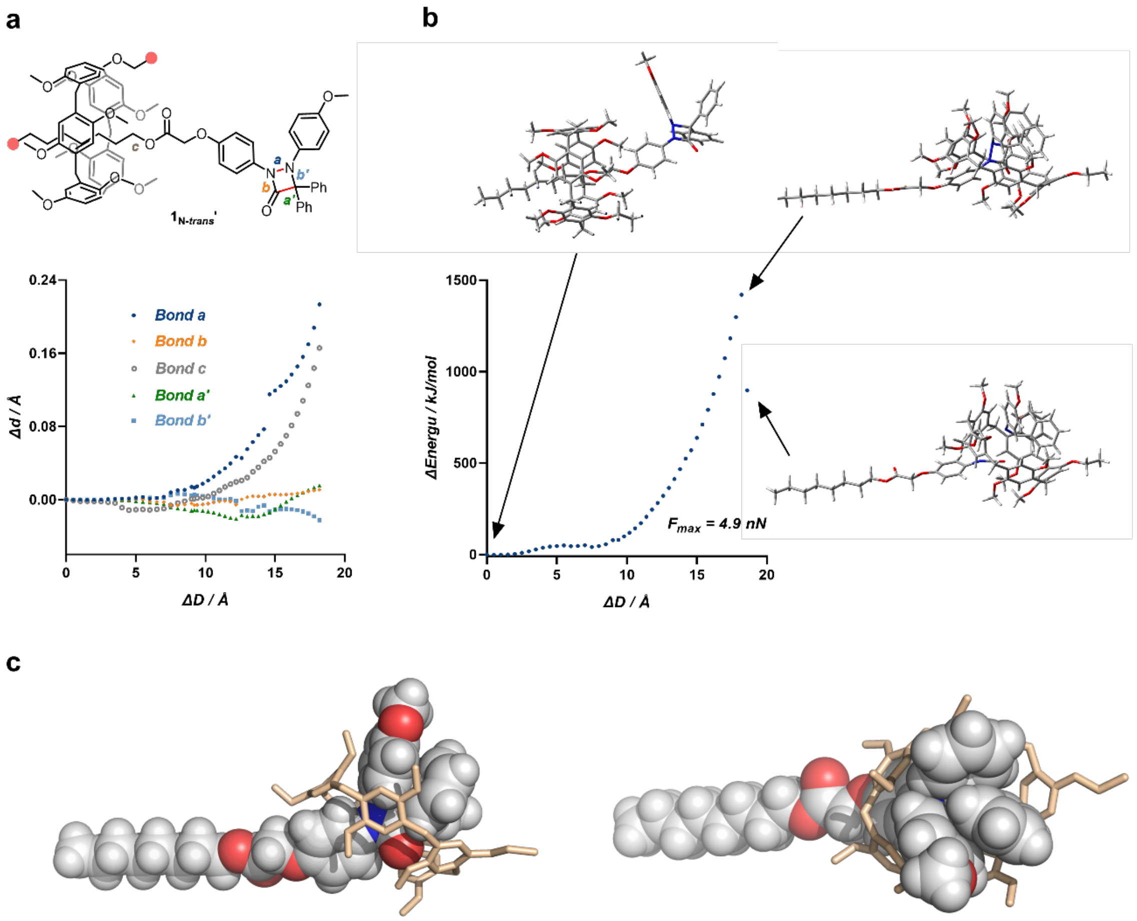

Figure S24. Evolution of bond *a*, *a'*, *b*, *b'*, and *c* (a) and energy (b) upon simulated elongation (CoGEF, DFT B3LYP/6-31G) of a model of diazetidinone structure in **$1_{N\text{-}trans}$'** activated by pushing force with rotaxane actuator. Models in the computation indicating key structural parameters. Predicted s*cis*sile bond are shown in red. Anchor atoms are indicated by the pink disks. And side and top views (c) of the model (space-filling for axle part and stick for macrocycle) of **$1_{N\text{-}trans}$'** at maximal deformation (E_{max}) reveals the extent of the contact surface between the mechanophore and the macrocycle. Hydrogen atoms omitted for clarity in the tube representation.

7.4 CoGEF of models of $1_{C\text{-}cis}$’

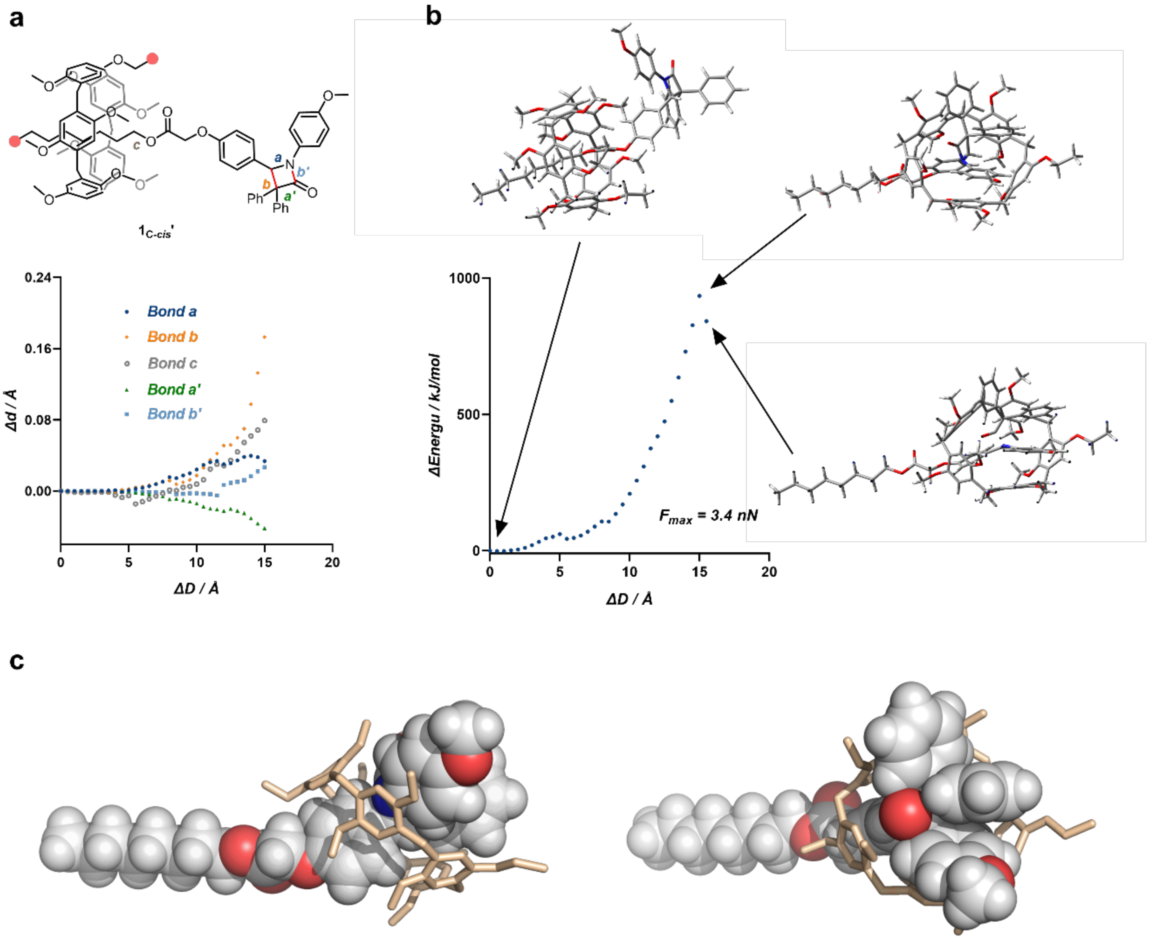

Figure S25. Evolution of bond *a*, *a’*, *b, b’*, and *c* (a) and energy (b) upon simulated elongation (CoGEF, DFT B3LYP/6-31G) of a model of β-lactam structure in $\mathbf{1}_{C\text{-}cis}$**’** activated by pushing force with rotaxane actuator. Models in the computation indicating key structural parameters. Predicted *scis*sile bond are shown in red. Anchor atoms are indicated by the pink disks. And side and top views (c) of the model (space-filling for axle part and stick for macrocycle) of $\mathbf{1}_{C\text{-}cis}$**’** at maximal deformation (E_{max}) reveals the extent of the contact surface between the mechanophore and the macrocycle. Hydrogen atoms omitted for clarity in the tube representation.

7.5 CoGEF of models of $1_{C\text{-}trans}$’

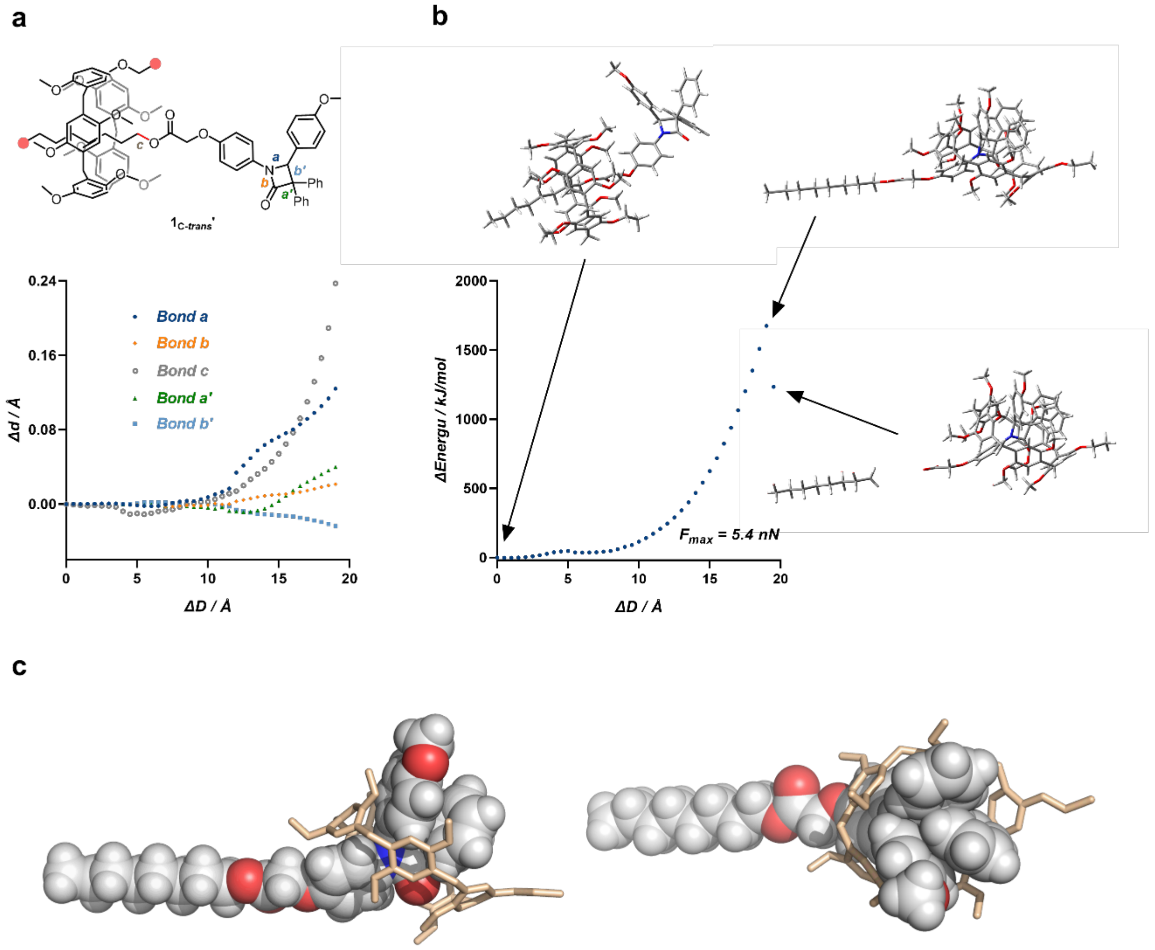

Figure S26. Evolution of bond *a*, *a’*, *b*, *b’*, and *c* (a) and energy (b) upon simulated elongation (CoGEF, DFT B3LYP/6-31G) of a model of β-lactam structure in $\mathbf{1}_{C\text{-}trans}$**’** activated by pushing force with rotaxane actuator. Models in the computation indicating key structural parameters. Predicted s*cis*sile bond are shown in red. Anchor atoms are indicated by the pink disks. And side and top views (c) of the model (space-filling for axle part and stick for macrocycle) of $\mathbf{1}_{C\text{-}trans}$**’** at maximal deformation (E_{max}) reveals the extent of the contact surface between the mechanophore and the macrocycle. Hydrogen atoms omitted for clarity in the tube representation.

8 NMR Spectra

8.1 Small Molecule NMR Spectra

8.1.1 Spectra of S1

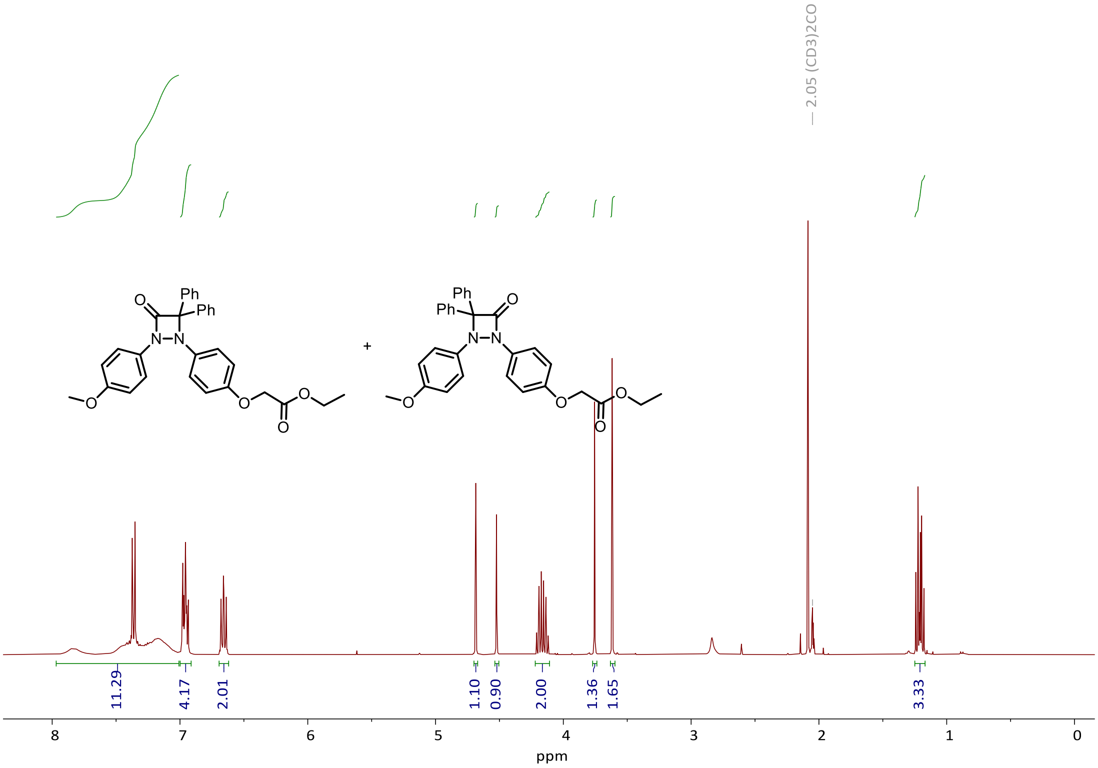

Spectrum S1. ^{1}H NMR (400 MHz, Acetone-d_6, 298 K) spectrum of compound **S1**.

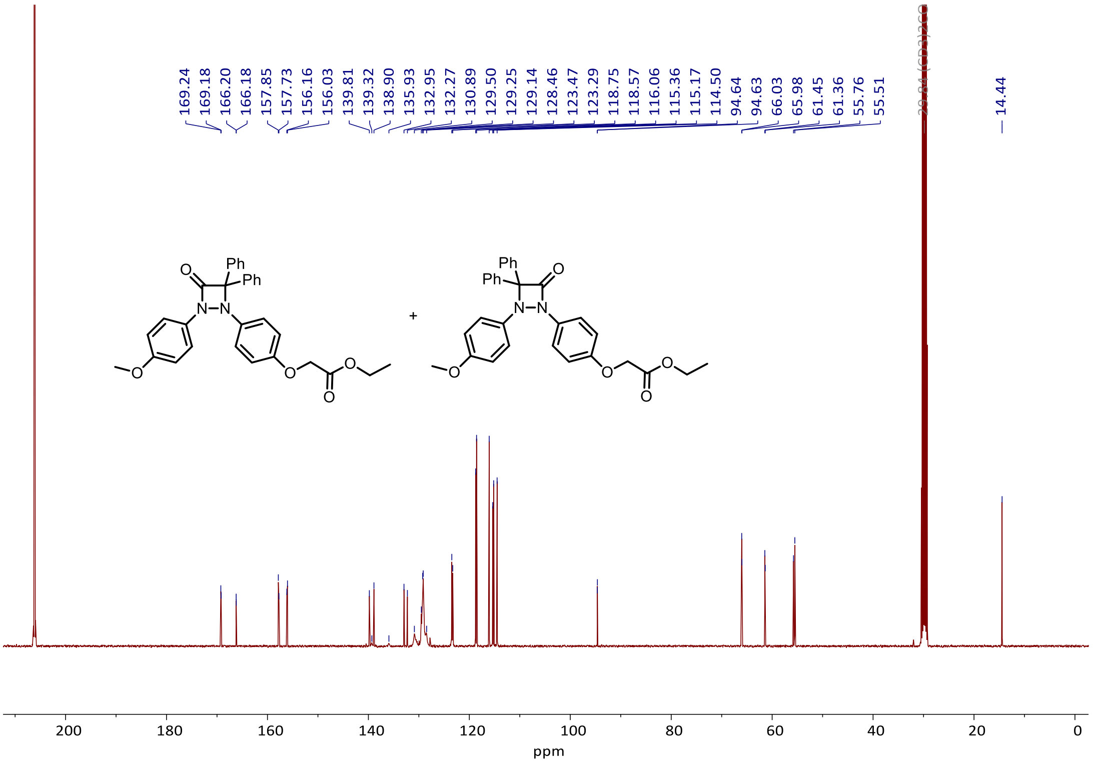

Spectrum S2. ^{13}C NMR (101 MHz, Acetone-d_6, 298 K) spectrum of compound **S1**.

8.1.2 Spectra of S3

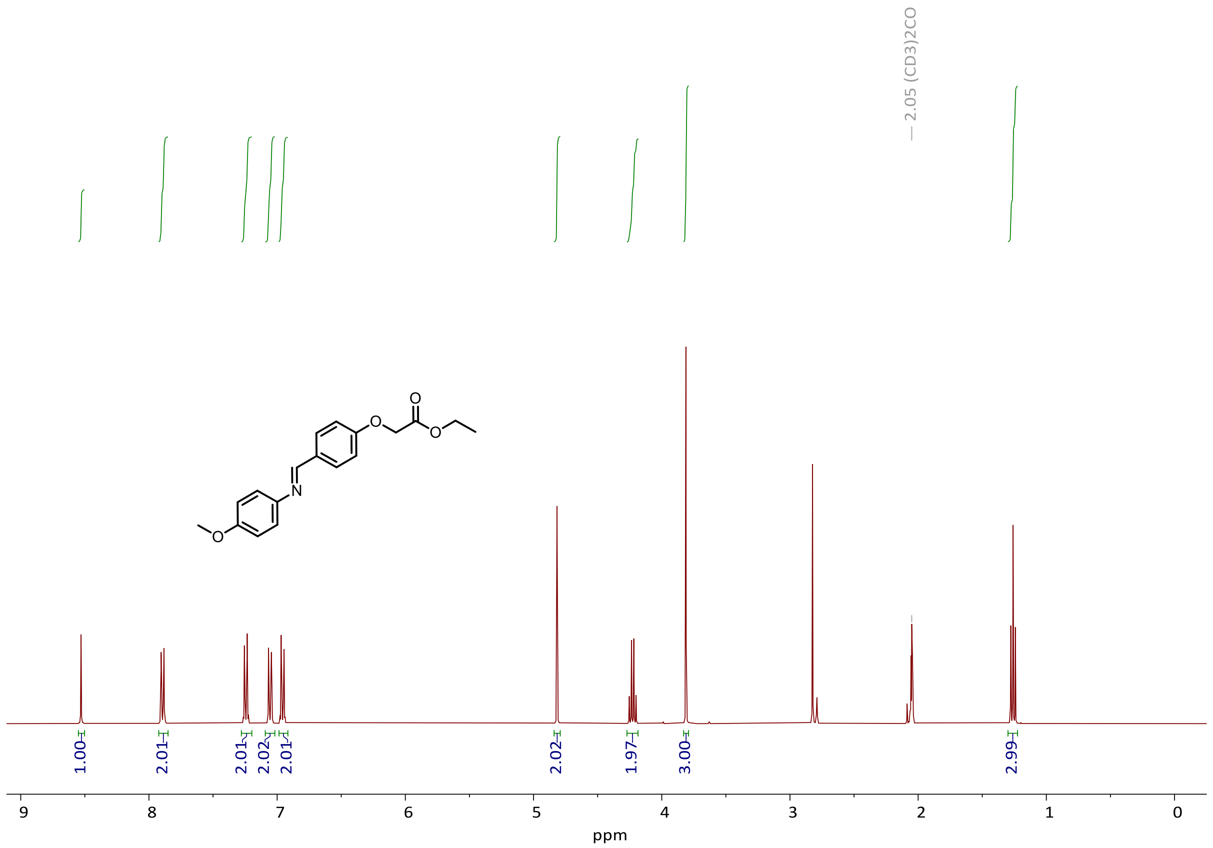

Spectrum S3. ^{1}H NMR (400 MHz, Acetone-d_6, 298 K) spectrum of compound **S3**.

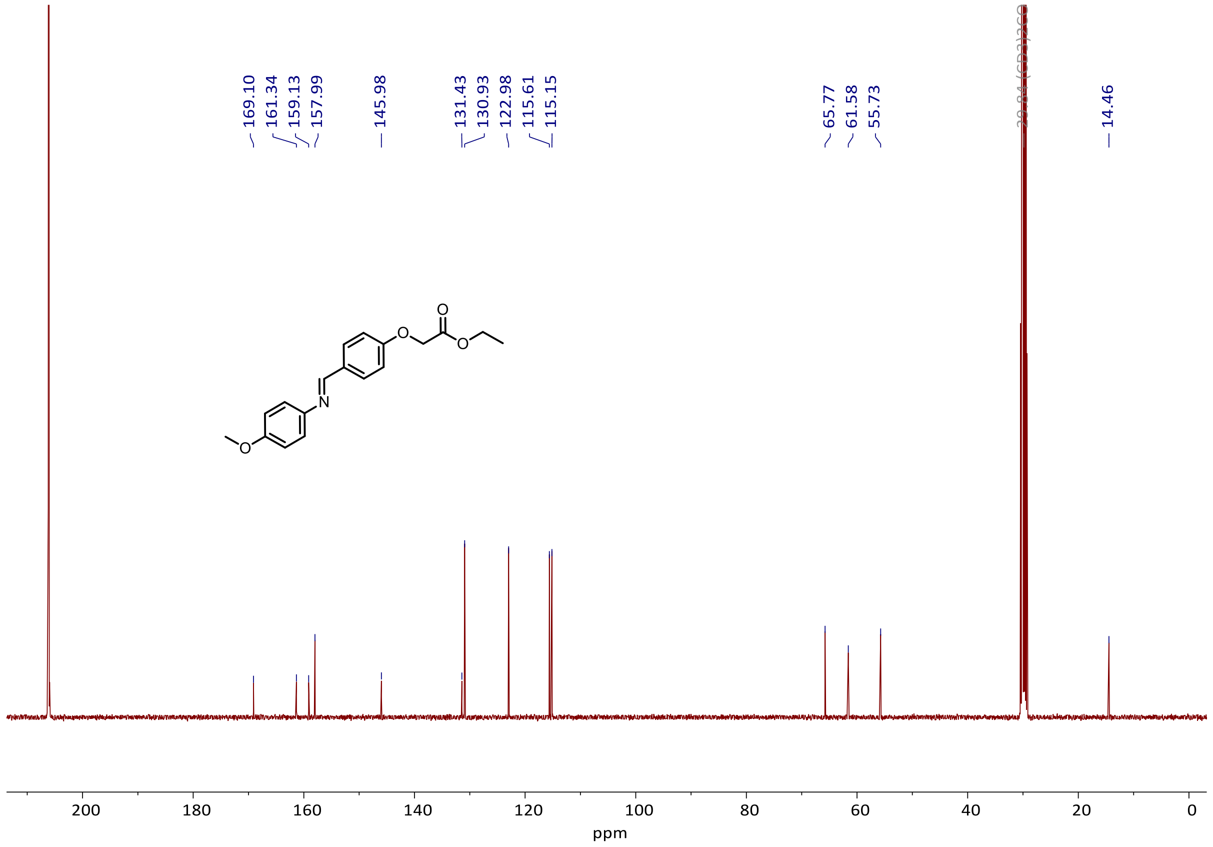

Spectrum S4. ^{13}C NMR (101 MHz, Acetone-d_6, 298 K) spectrum of compound **S3**.

8.1.3 Spectra of S4

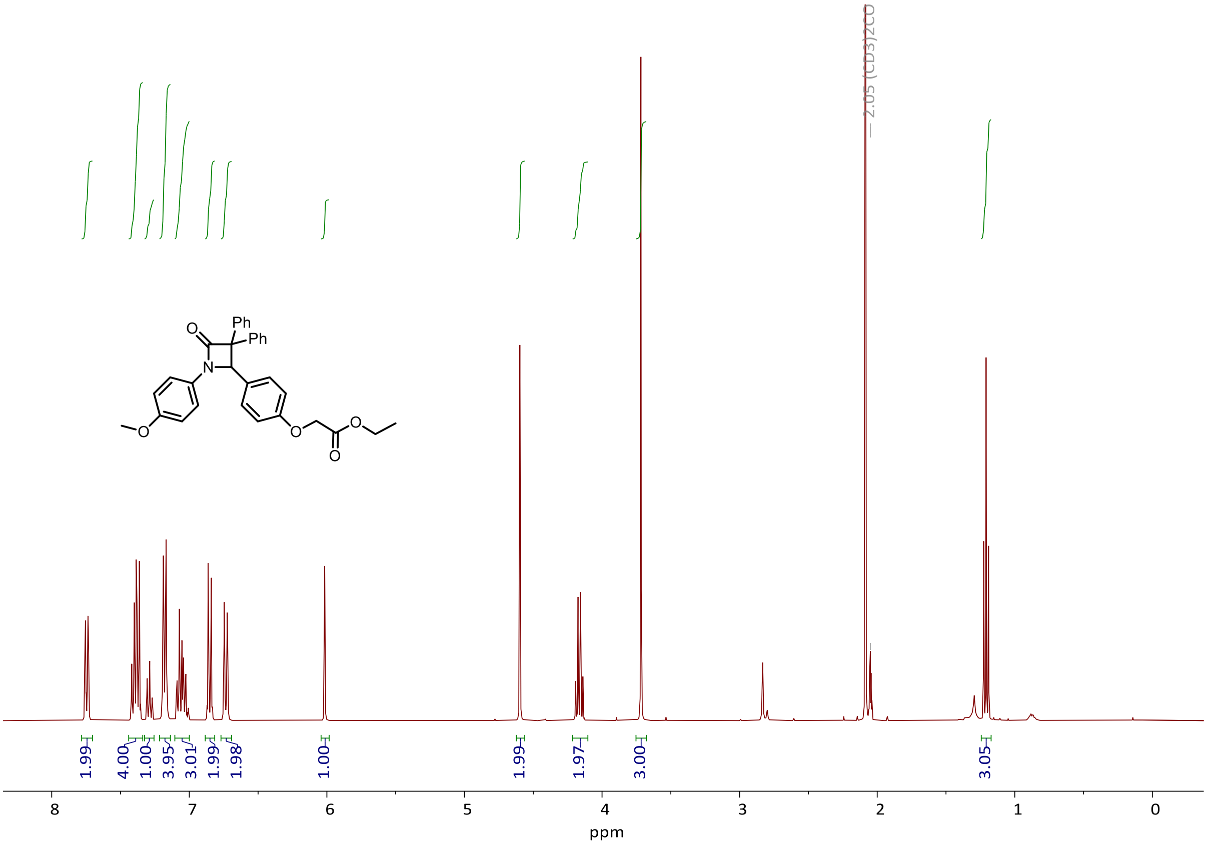

Spectrum S5. ^{1}H NMR (400 MHz, Acetone-d_6, 298 K) spectrum of compound **S4**.

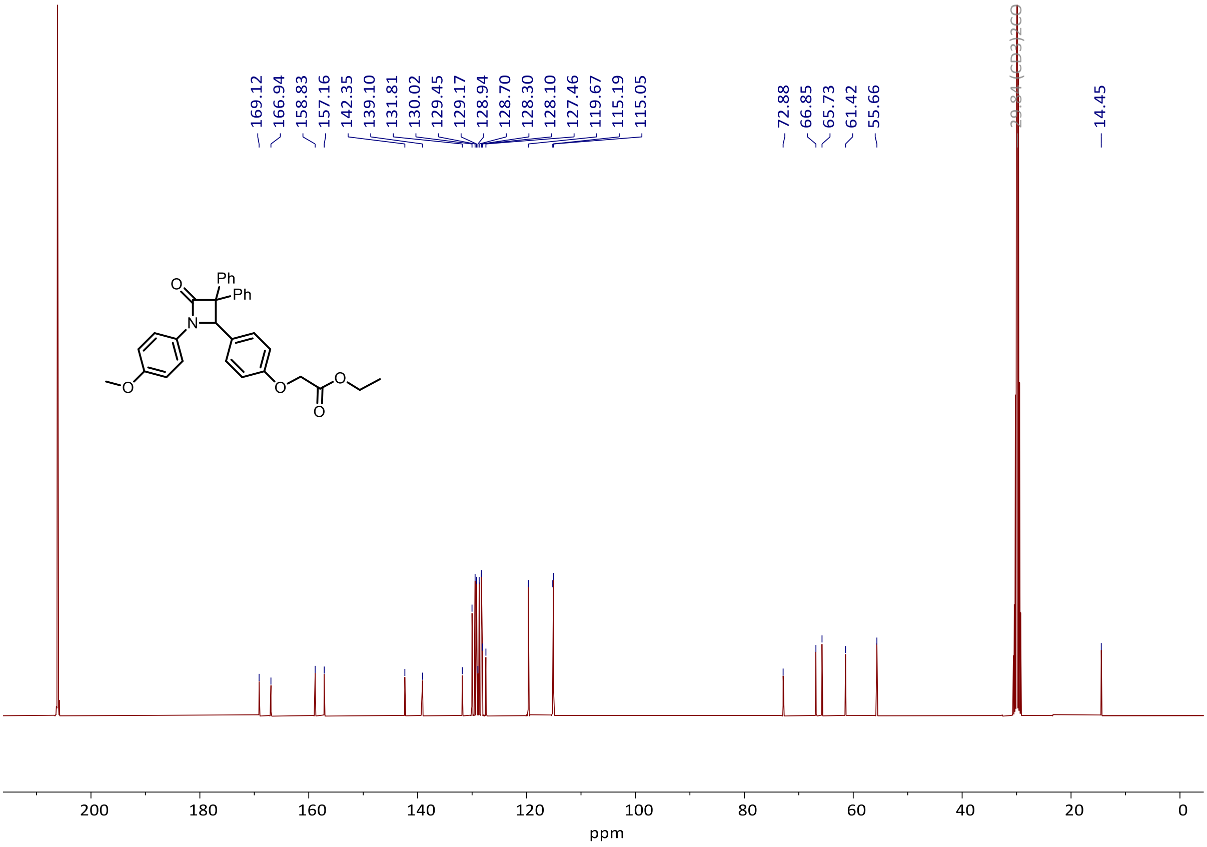

Spectrum S6. ^{13}C NMR (101 MHz, Acetone-d_6, 298 K) spectrum of compound **S4**.

8.1.4 Spectra of S5

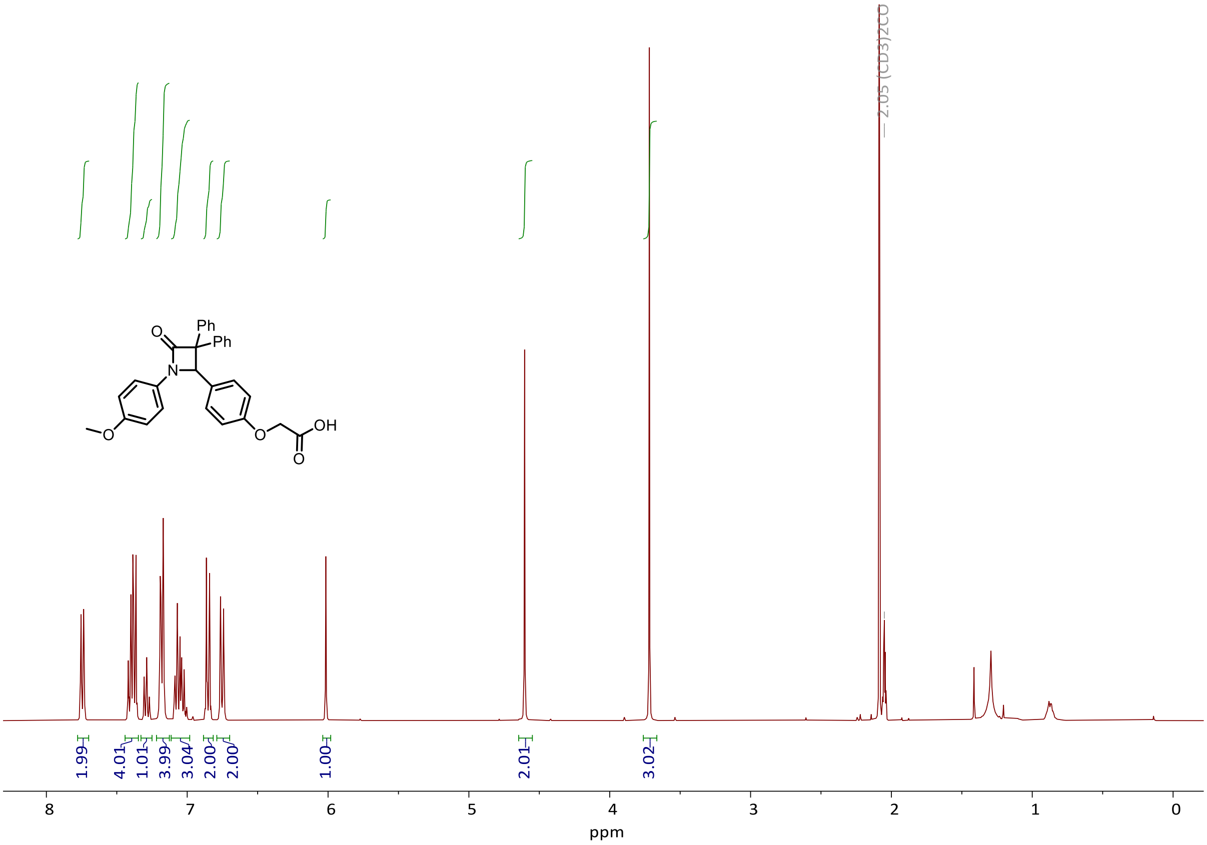

Spectrum S7. ^{1}H NMR (400 MHz, Acetone-d_6, 298 K) spectrum of compound **S5**.

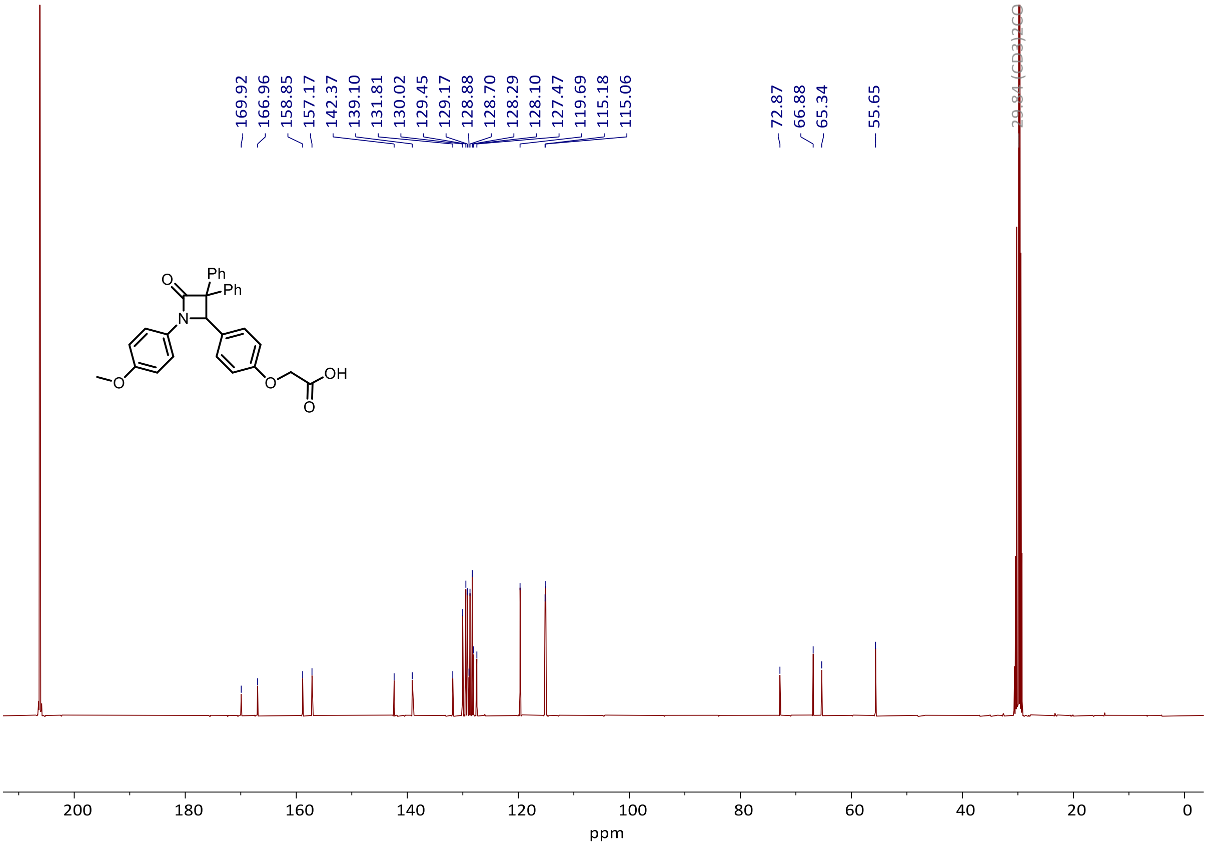

Spectrum S8. ^{13}C NMR (101 MHz, Acetone-d_6, 298 K) spectrum of compound **S5**.

8.1.5 Spectra of S7

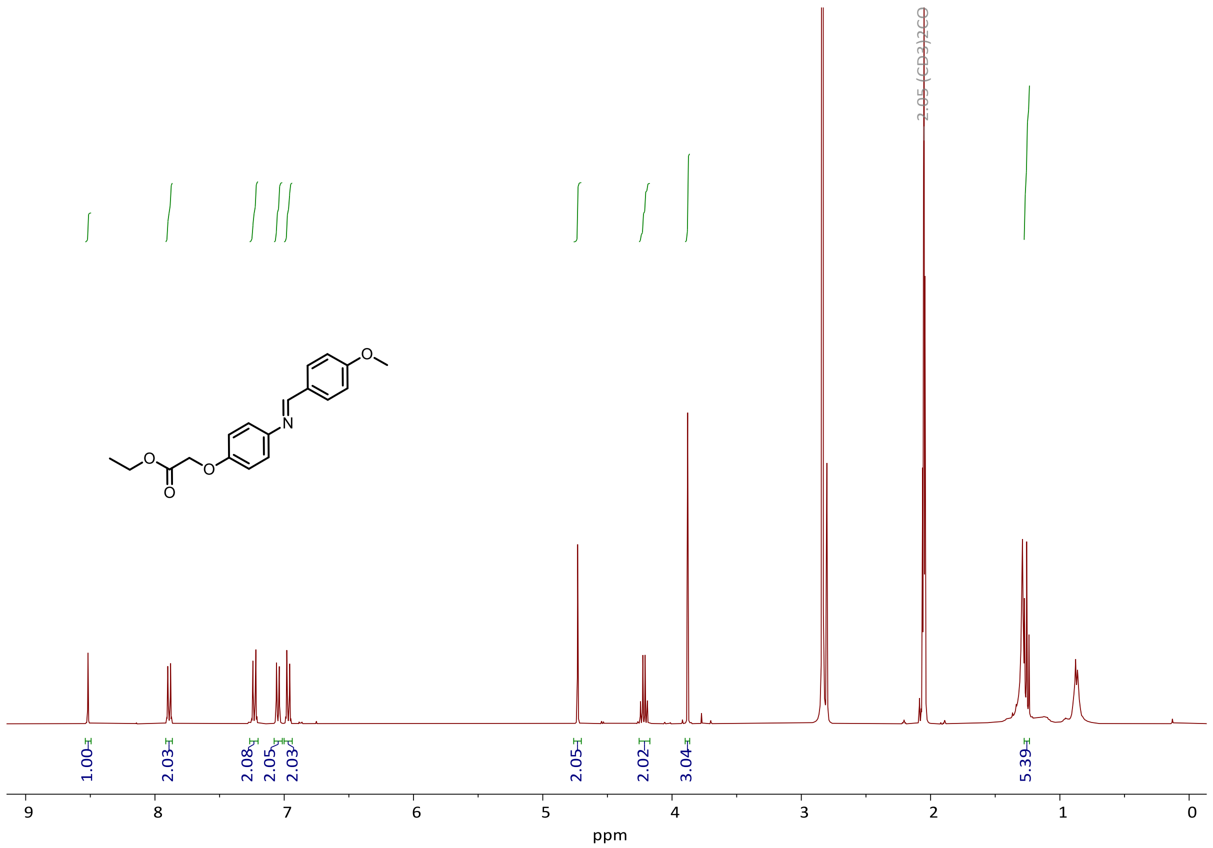

Spectrum S9. ^{1}H NMR (400 MHz, Acetone-d_6, 298 K) spectrum of compound **S7**.

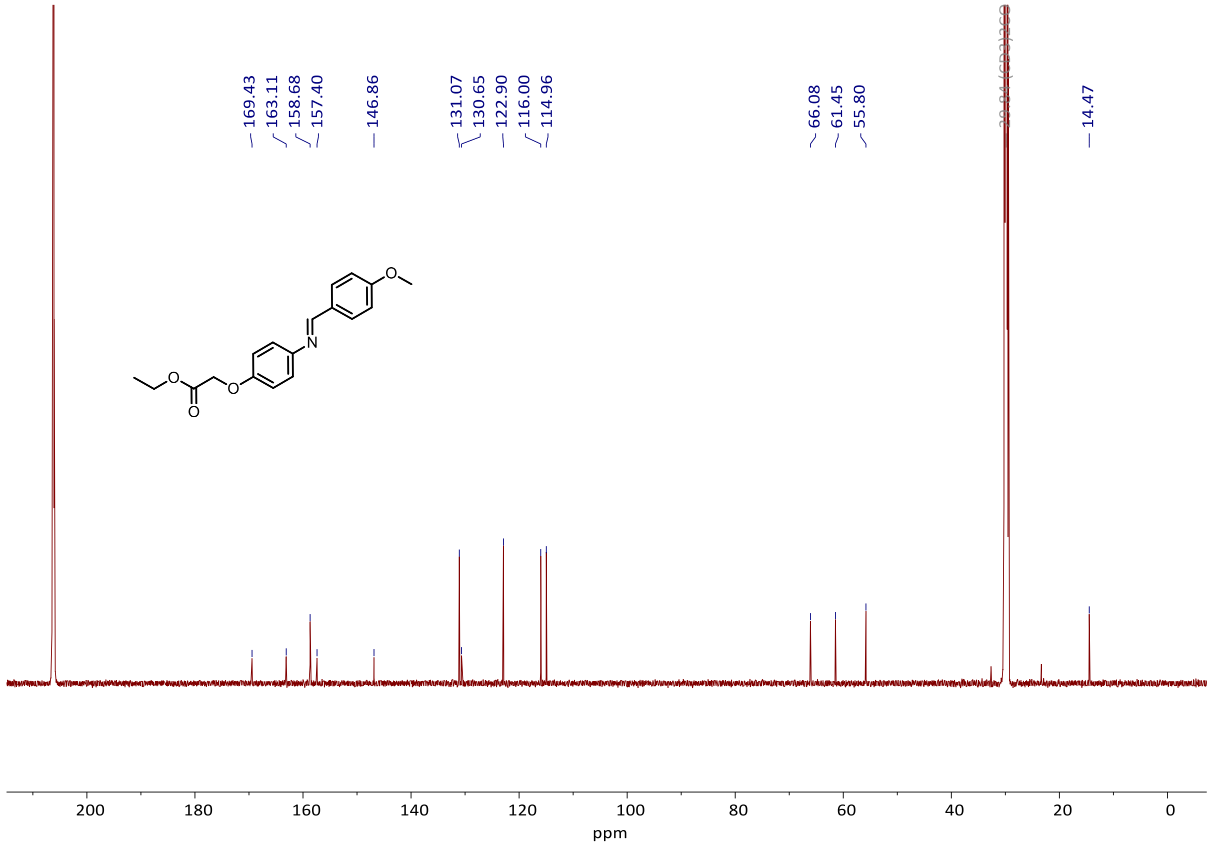

Spectrum S10. ^{13}C NMR (101 MHz, Acetone-d_6, 298 K) spectrum of compound **S7**.

8.1.6 Spectra of S8

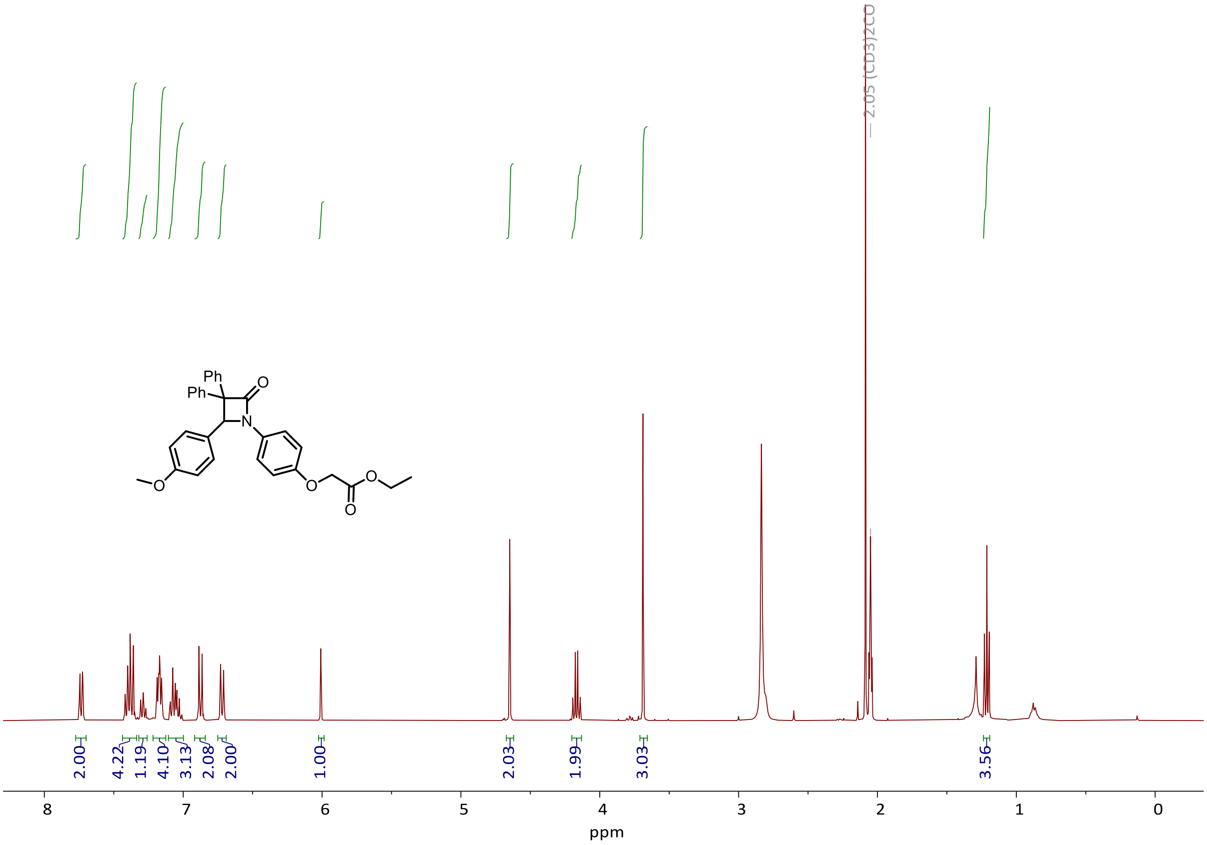

Spectrum S11. ^{1}H NMR (400 MHz, Acetone-d_6, 298 K) spectrum of compound **S8**.

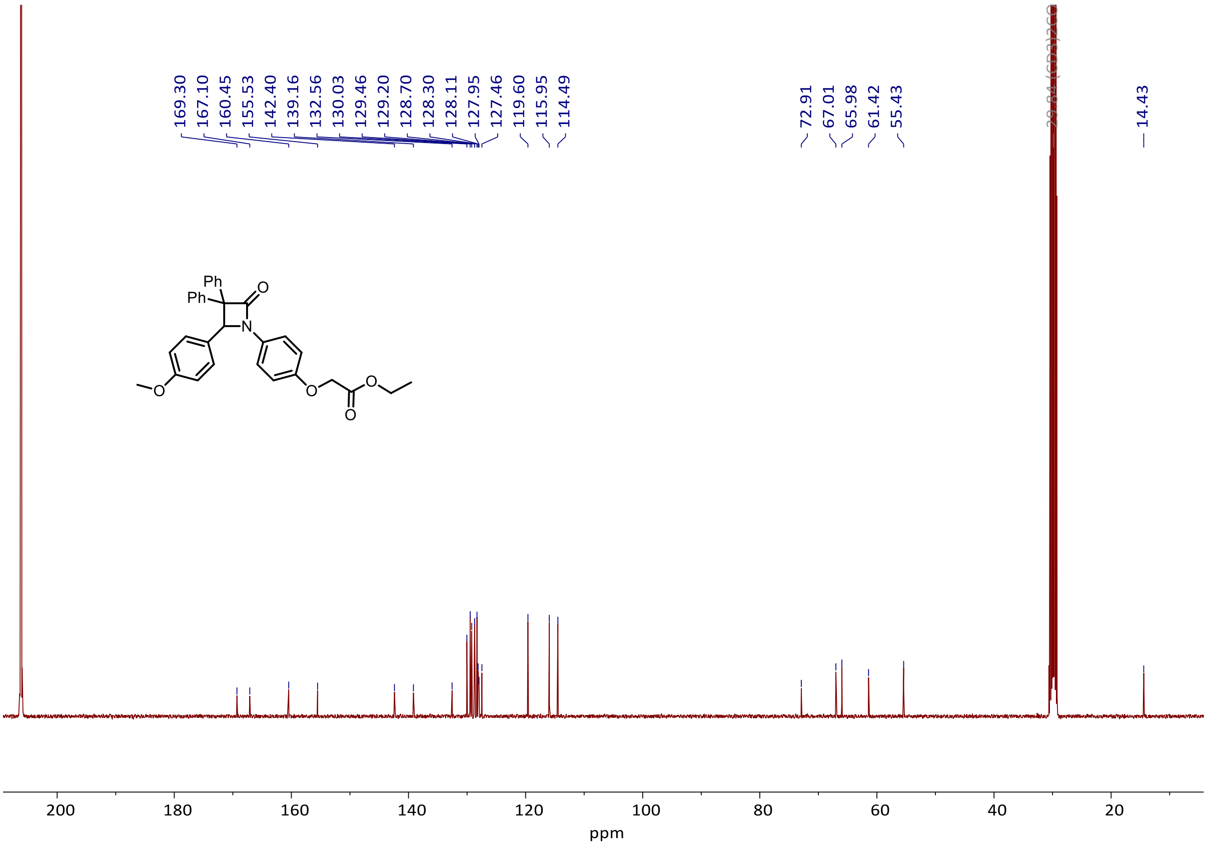

Spectrum S12. ^{13}C NMR (101 MHz, Acetone-d_6, 298 K) spectrum of compound **S8**.

8.1.7 Spectra of S9

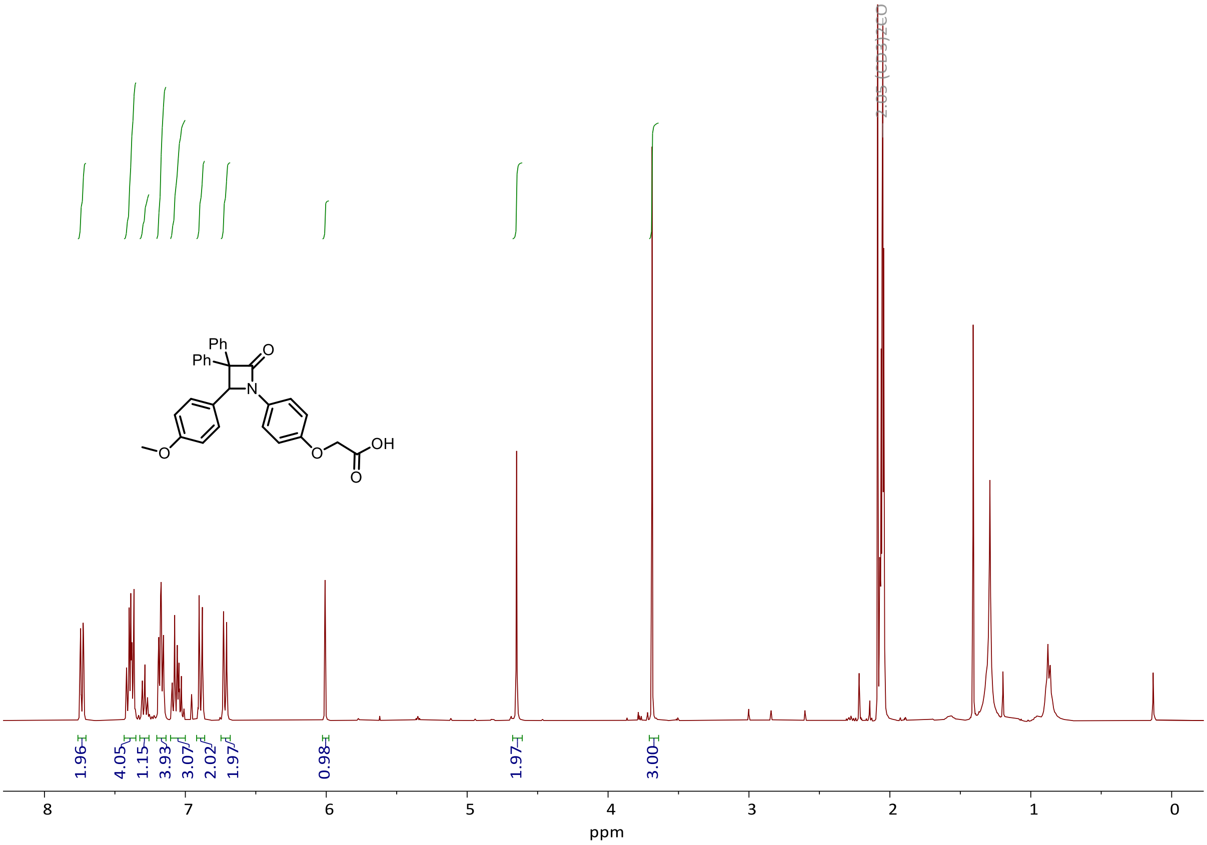

Spectrum S13. ^{1}H NMR (400 MHz, Acetone-d_6, 298 K) spectrum of compound **S9**.

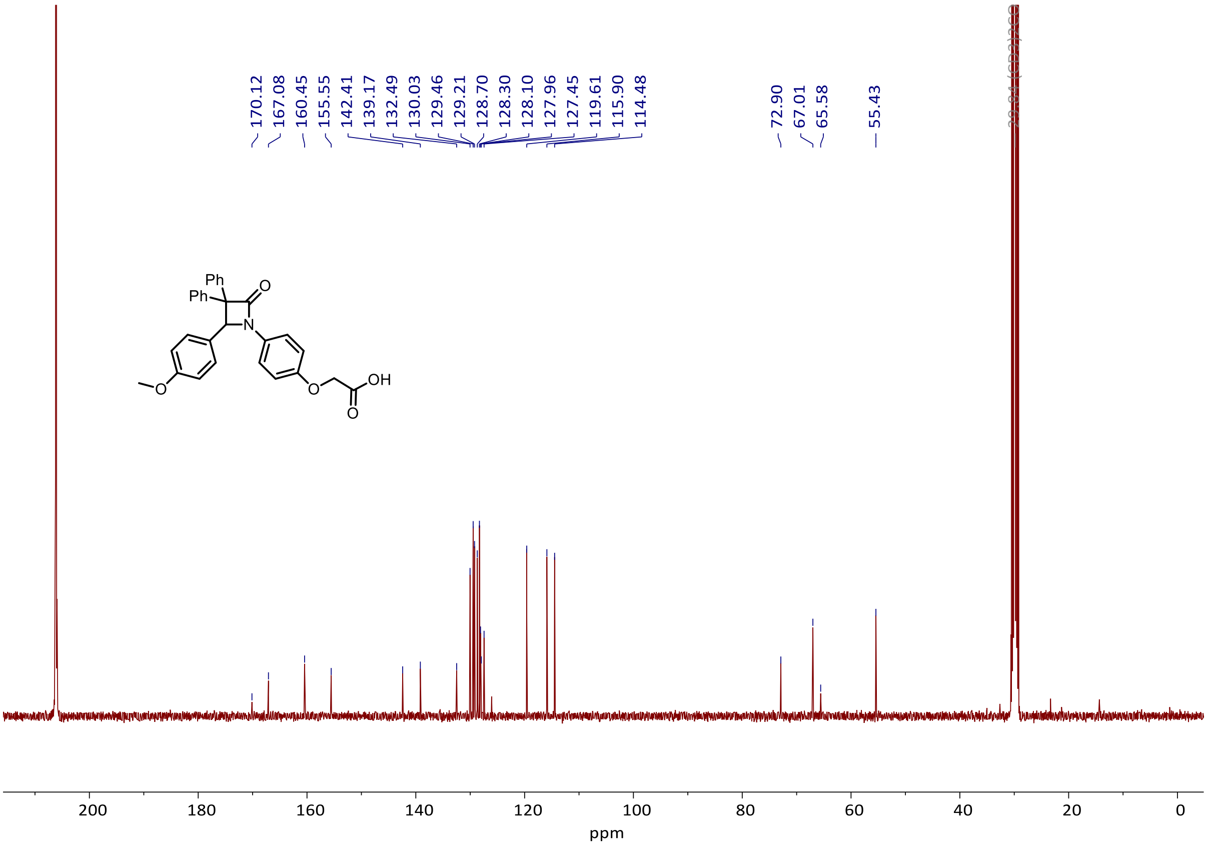

Spectrum S14. ^{13}C NMR (101 MHz, Acetone-d_6, 298 K) spectrum of compound **S9**.

8.1.8 Spectra of $11_{N\text{-}cis}$

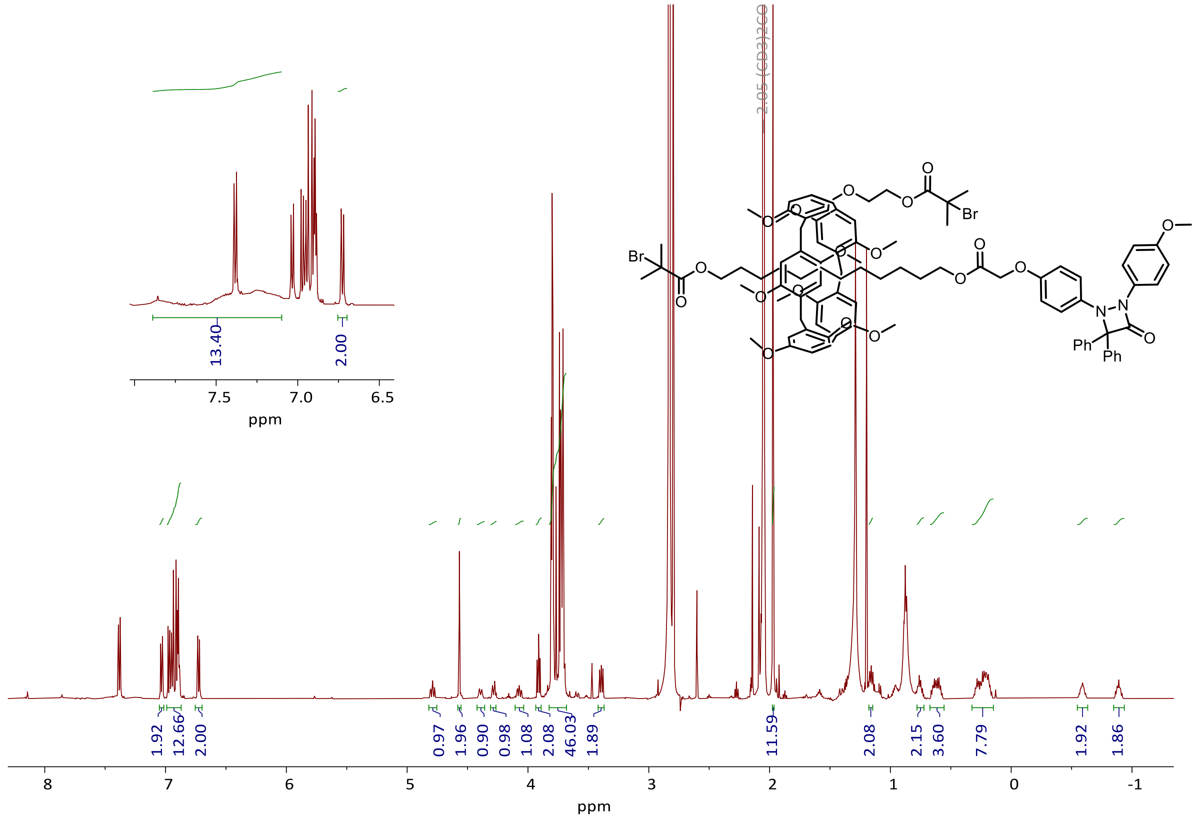

Spectrum S15. ^{1}H NMR (600 MHz, Acetone-d_6, 298 K) spectrum of compound $\mathbf{11}_{N\text{-}cis}$.

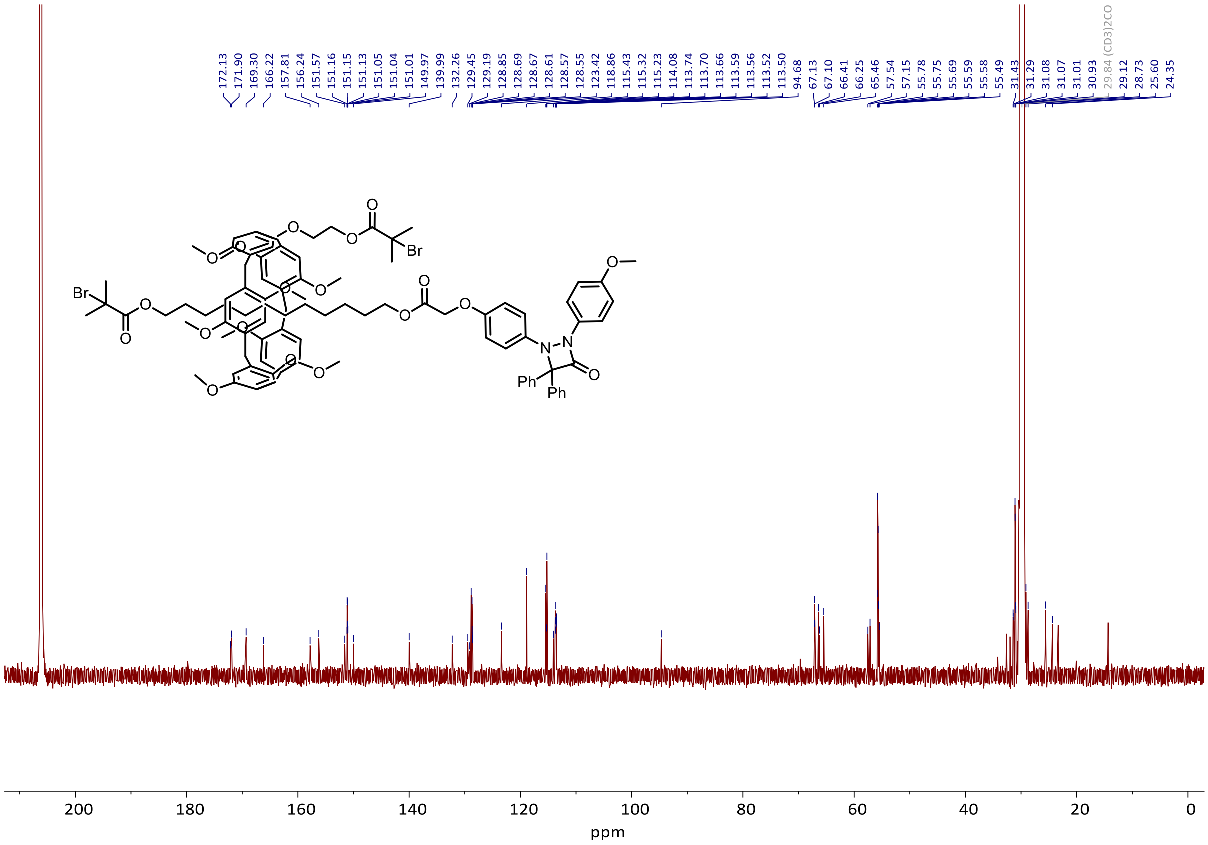

Spectrum S16. ^{13}C NMR (151 MHz, Acetone-d_6, 298 K) spectrum of compound $\mathbf{11}_{N\text{-}cis}$.

8.1.9 Spectra of $11_{N\text{-}trans}$

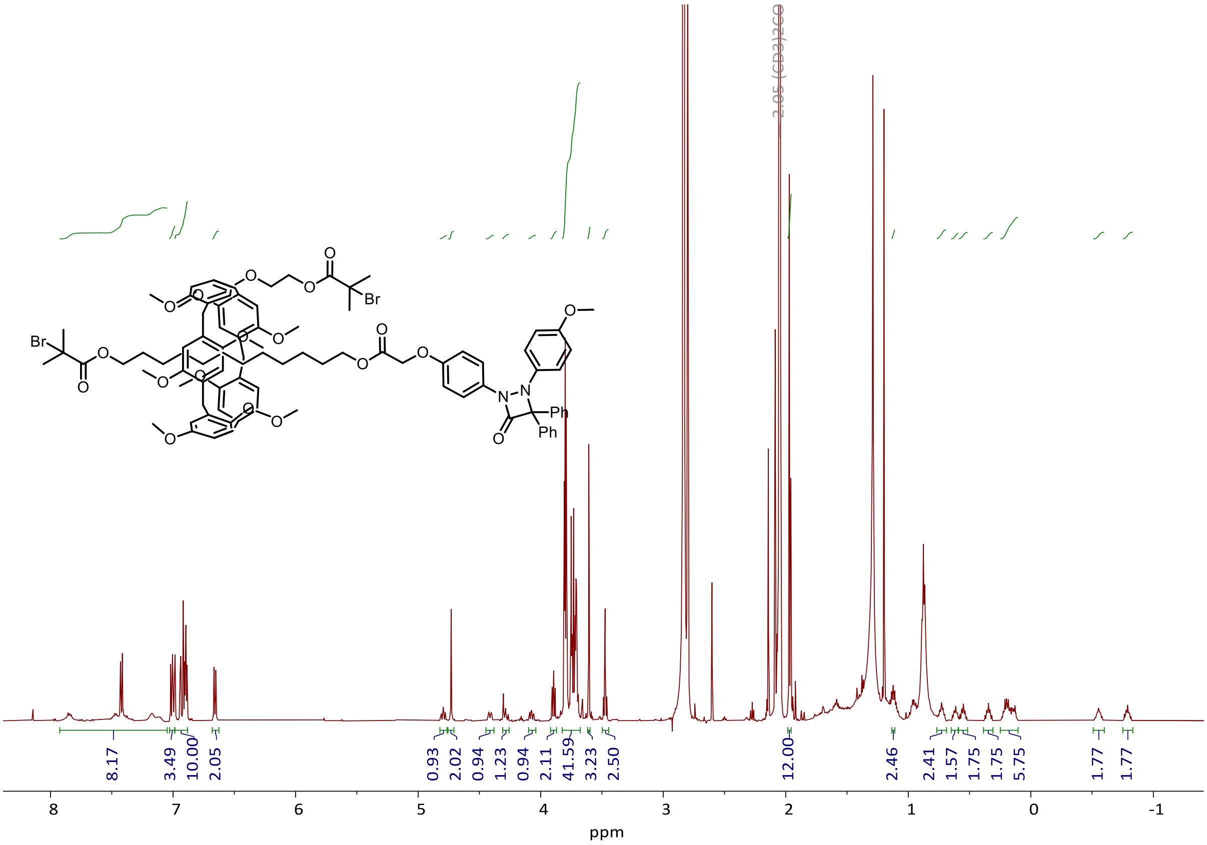

Spectrum S17. ^{1}H NMR (600 MHz, Acetone-d_6, 298 K) spectrum of compound $\mathbf{11}_{N\text{-}trans}$.

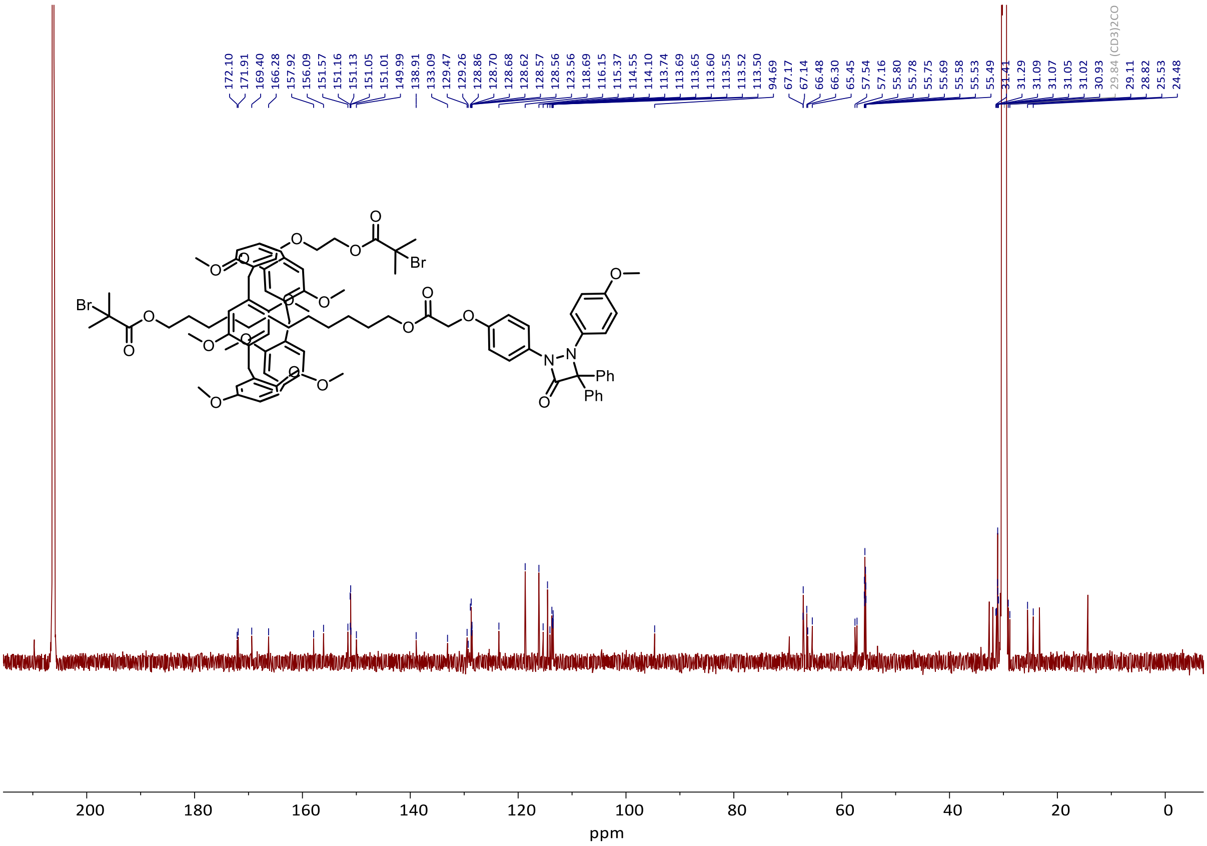

Spectrum S18. ^{13}C NMR (151 MHz, Acetone-d_6, 298 K) spectrum of compound $\mathbf{11}_{N\text{-}trans}$.

8.1.10 Spectra of $\mathbf{11}_{\mathbf{C}\text{-}\boldsymbol{cis}}$

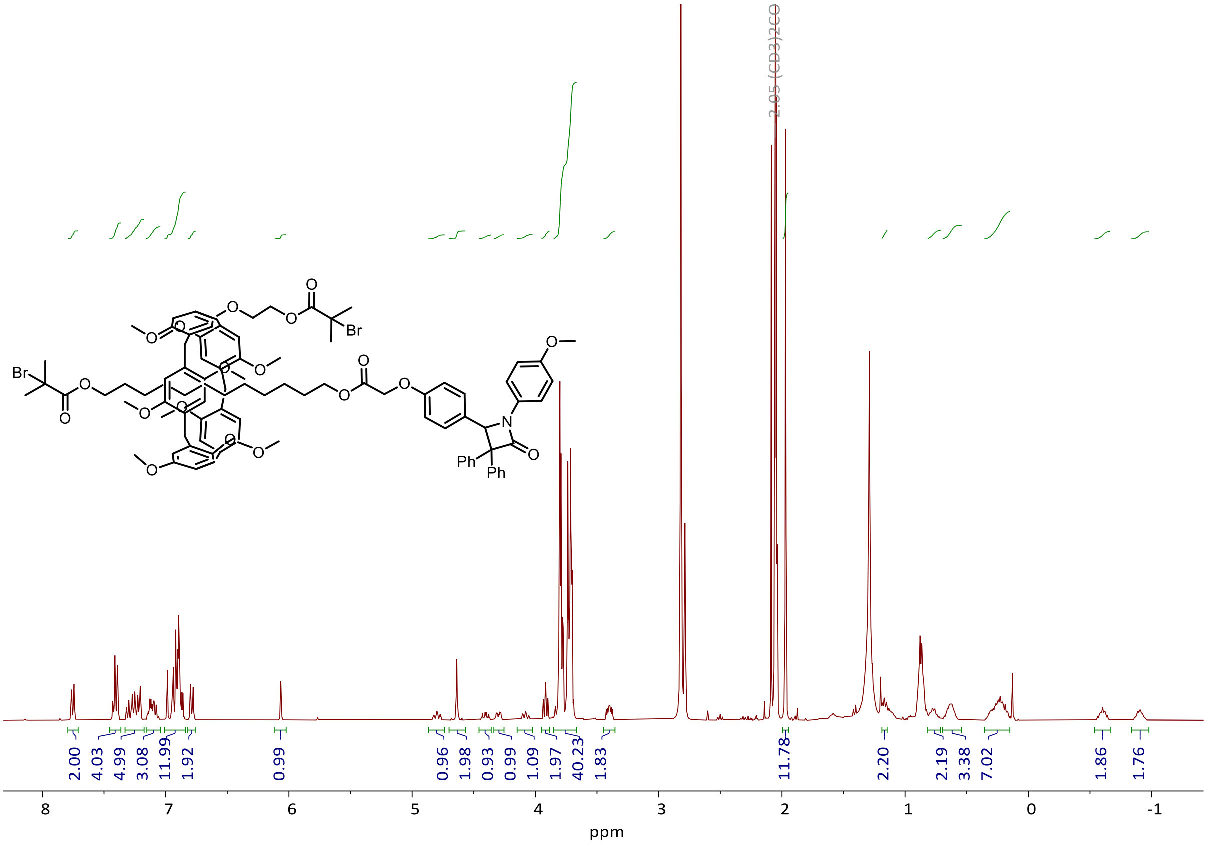

Spectrum S19. ^{1}H NMR (400 MHz, Acetone-d_6, 298 K) spectrum of compound $\mathbf{11}_{\mathbf{C}\text{-}\boldsymbol{cis}}$.

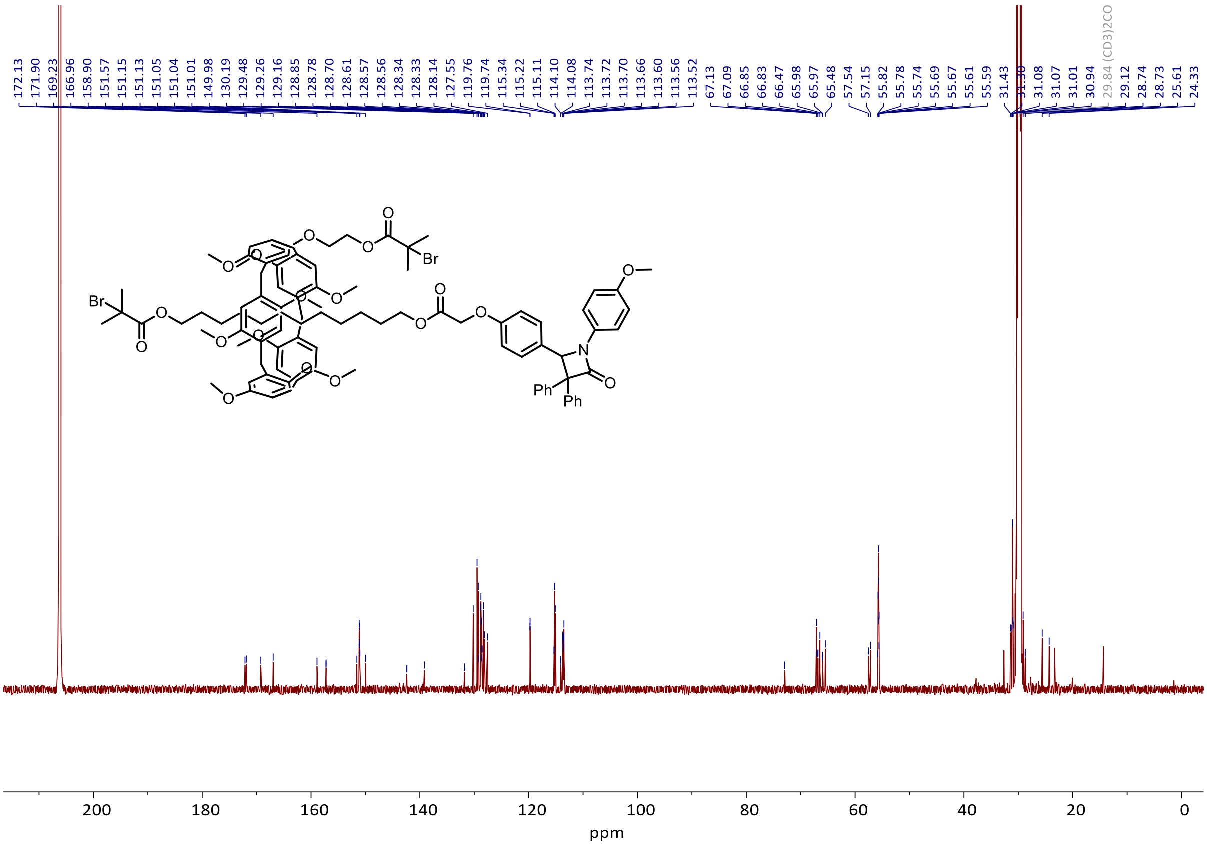

Spectrum S20. ^{13}C NMR (151 MHz, Acetone-d_6, 298 K) spectrum of compound $\mathbf{11}_{\mathbf{C}\text{-}\boldsymbol{cis}}$.

8.1.11 Spectra of 11$_{C\text{-}trans}$

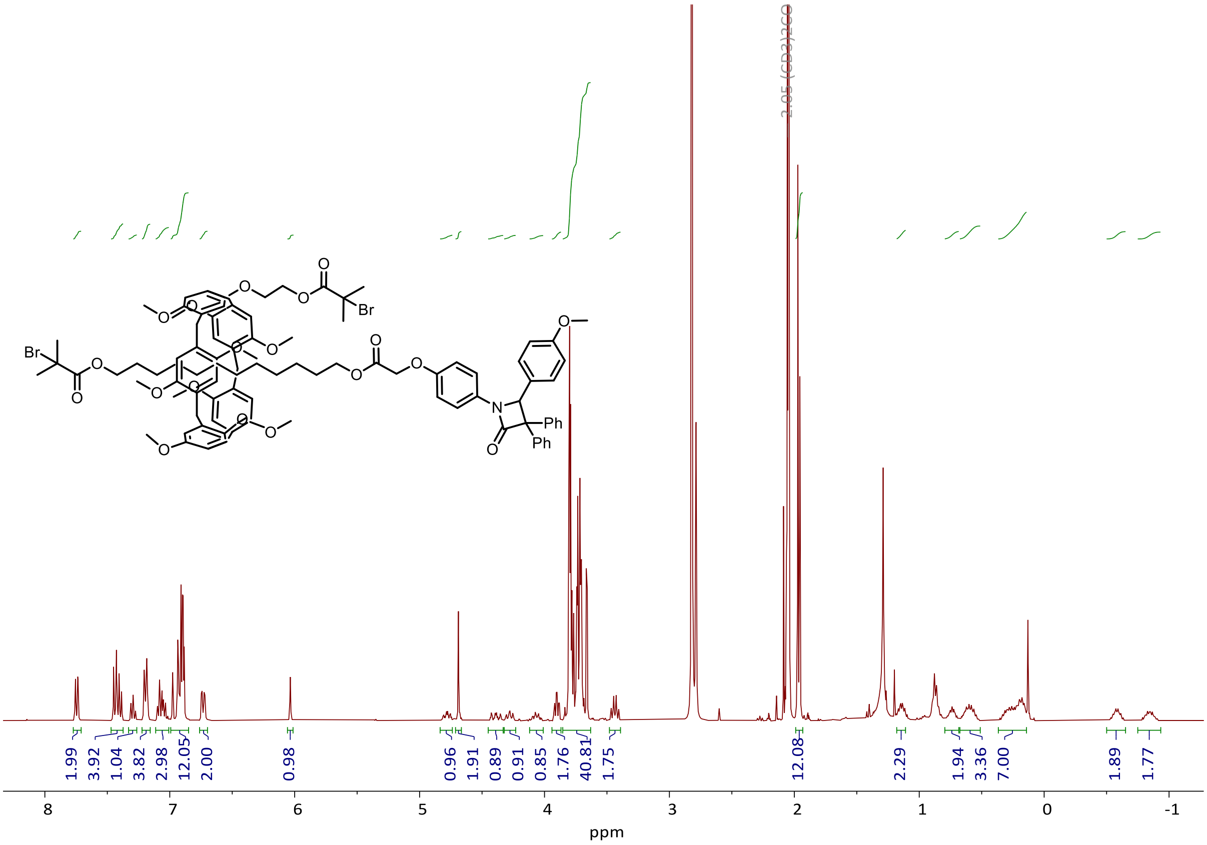

Spectrum S21. ^{1}H NMR (400 MHz, Acetone-d_6, 298 K) spectrum of compound **11**$_{\mathbf{C}\text{-}\boldsymbol{trans}}$.

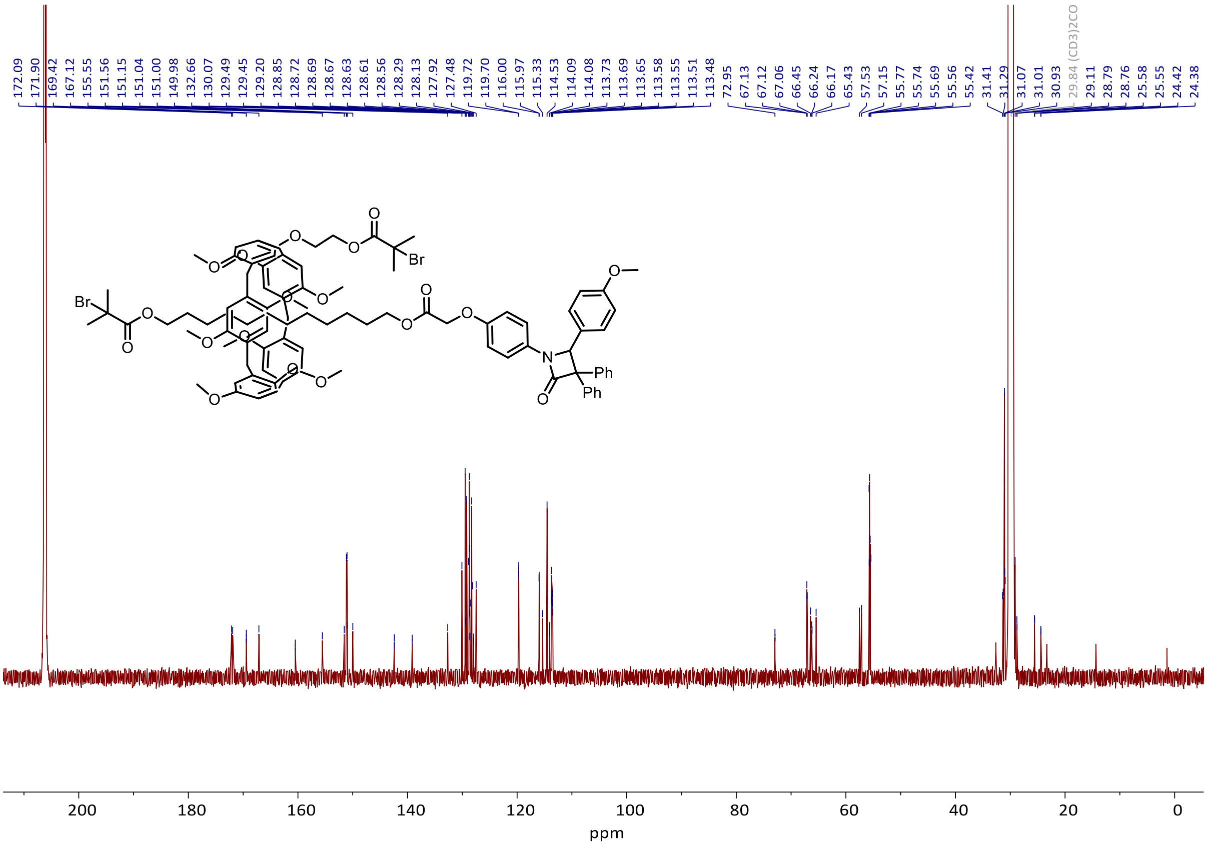

Spectrum S22. ^{13}C NMR (151 MHz, Acetone-d_6, 298 K) spectrum of compound **11**$_{\mathbf{C}\text{-}\boldsymbol{trans}}$.

8.1.12 Spectra of S11

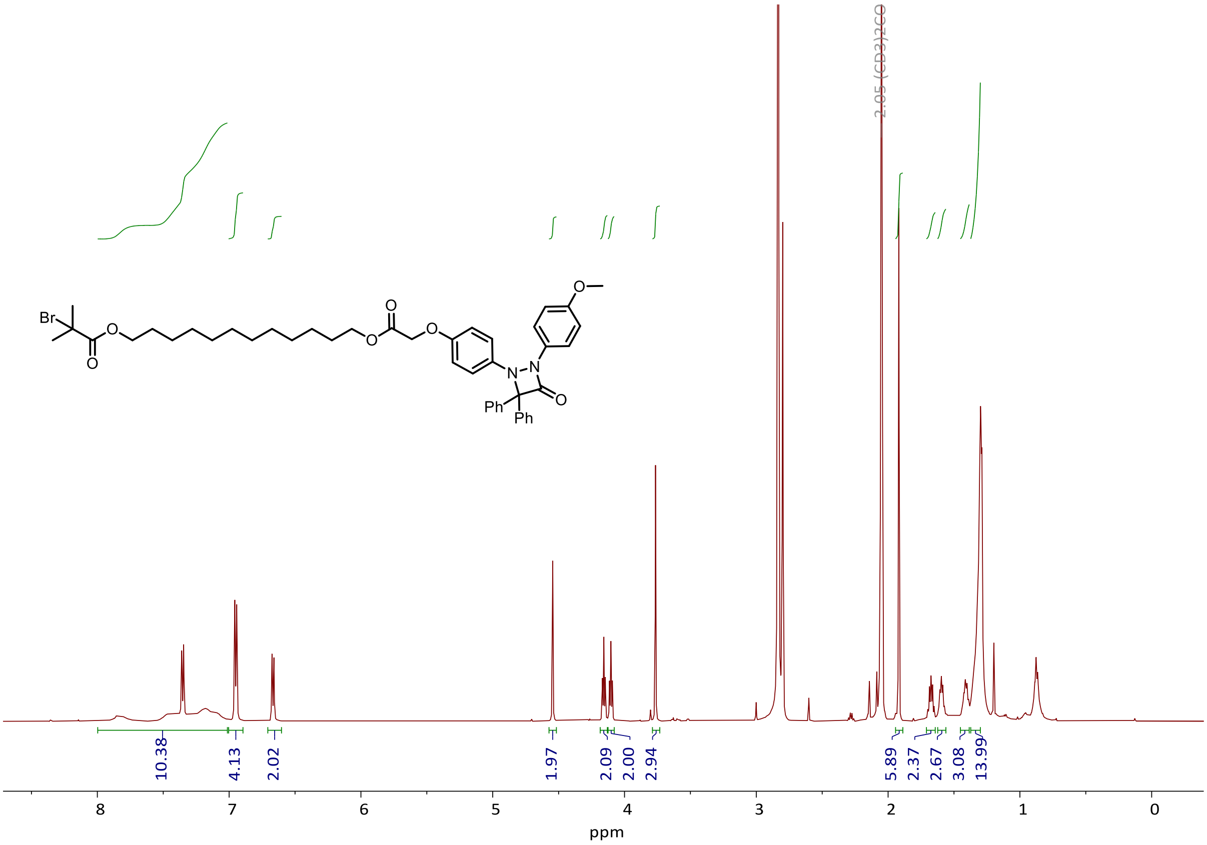

Spectrum S23. ^{1}H NMR (600 MHz, Acetone-d_6, 298 K) spectrum of compound **S11**.

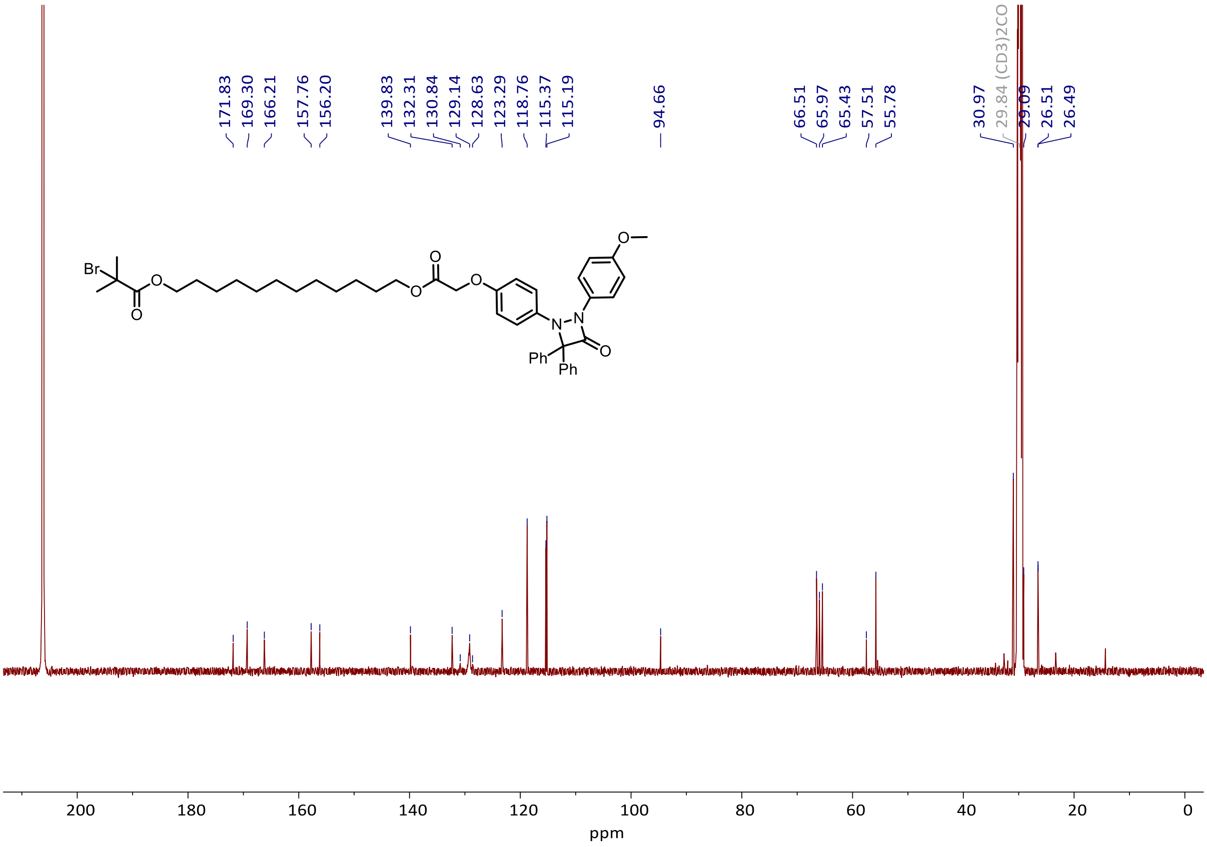

Spectrum S24. ^{13}C NMR (151 MHz, Acetone-d_6, 298 K) spectrum of compound **S11**.

8.1.13 Spectra of S12

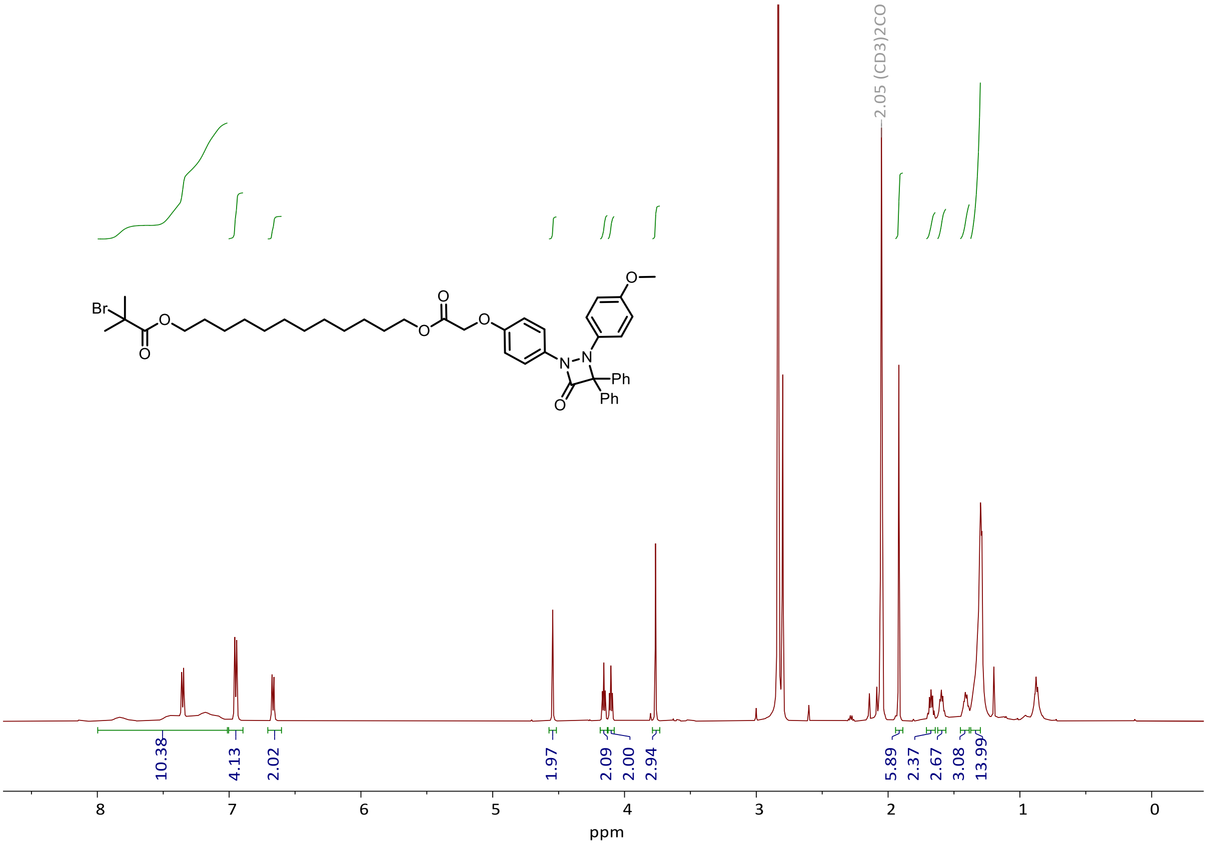

Spectrum S25. ^{1}H NMR (600 MHz, Acetone-d_6, 298 K) spectrum of compound **S12**.

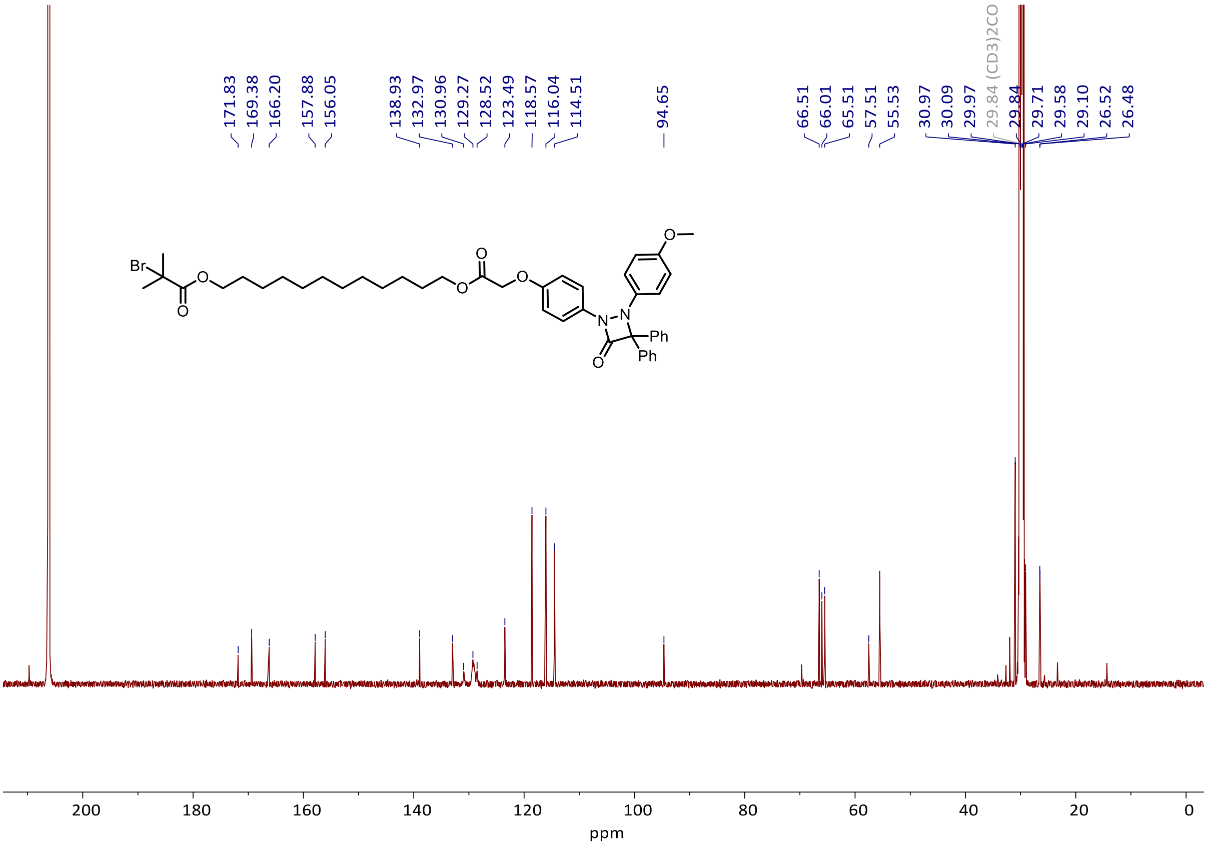

Spectrum S26. ^{13}C NMR (151 MHz, Acetone-d_6, 298 K) spectrum of compound **S12**.

8.1.14 Spectra of S13

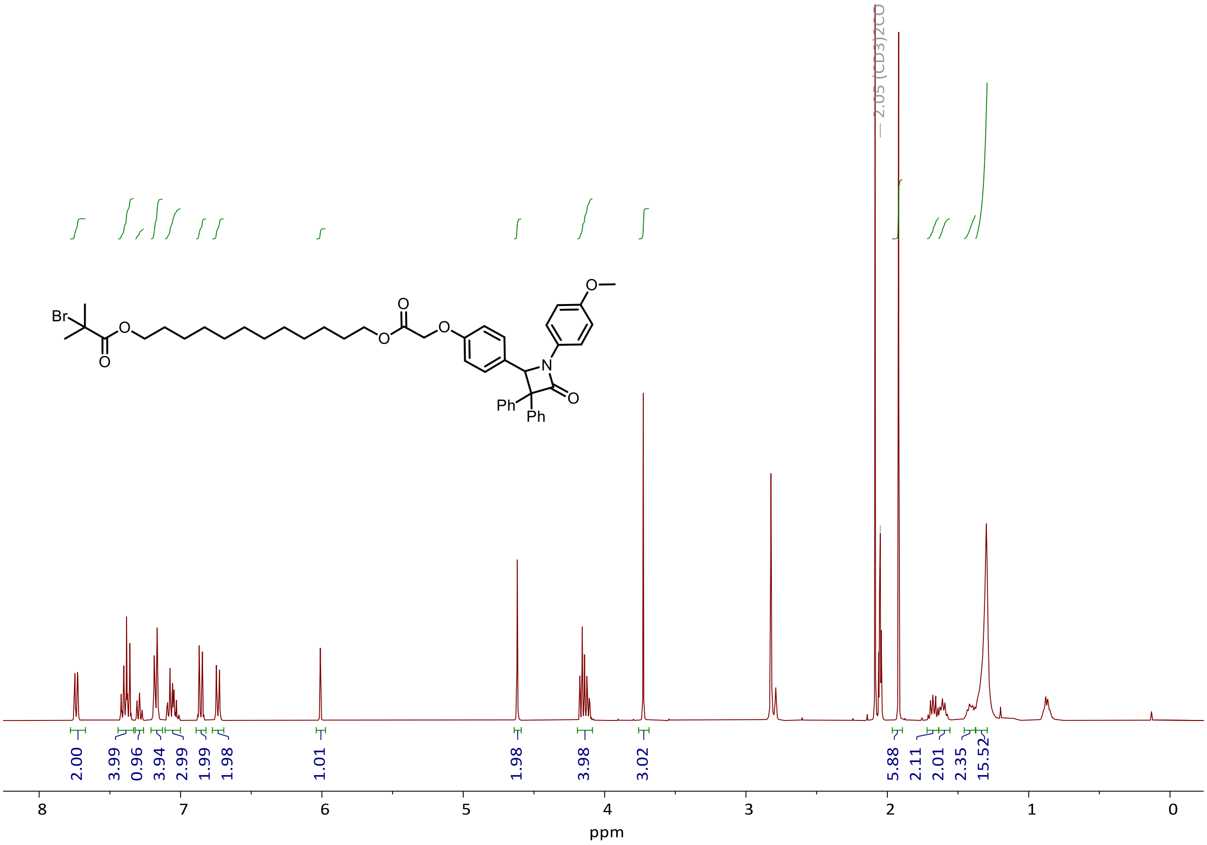

Spectrum S27. ^{1}H NMR (400 MHz, Acetone-d_6, 298 K) spectrum of compound **S13**.

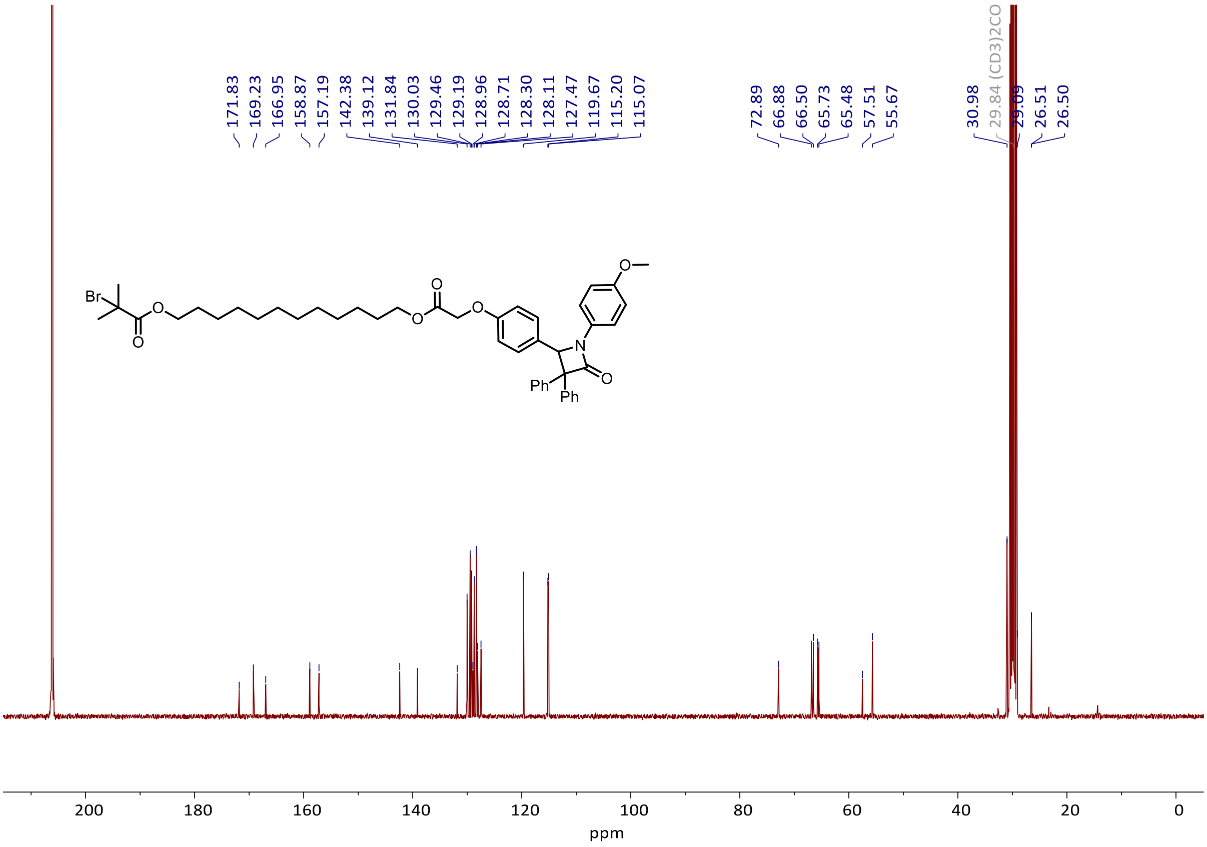

Spectrum S28. ^{13}C NMR (101 MHz, Acetone-d_6, 298 K) spectrum of compound **S13**.

8.1.15 Spectra of S14

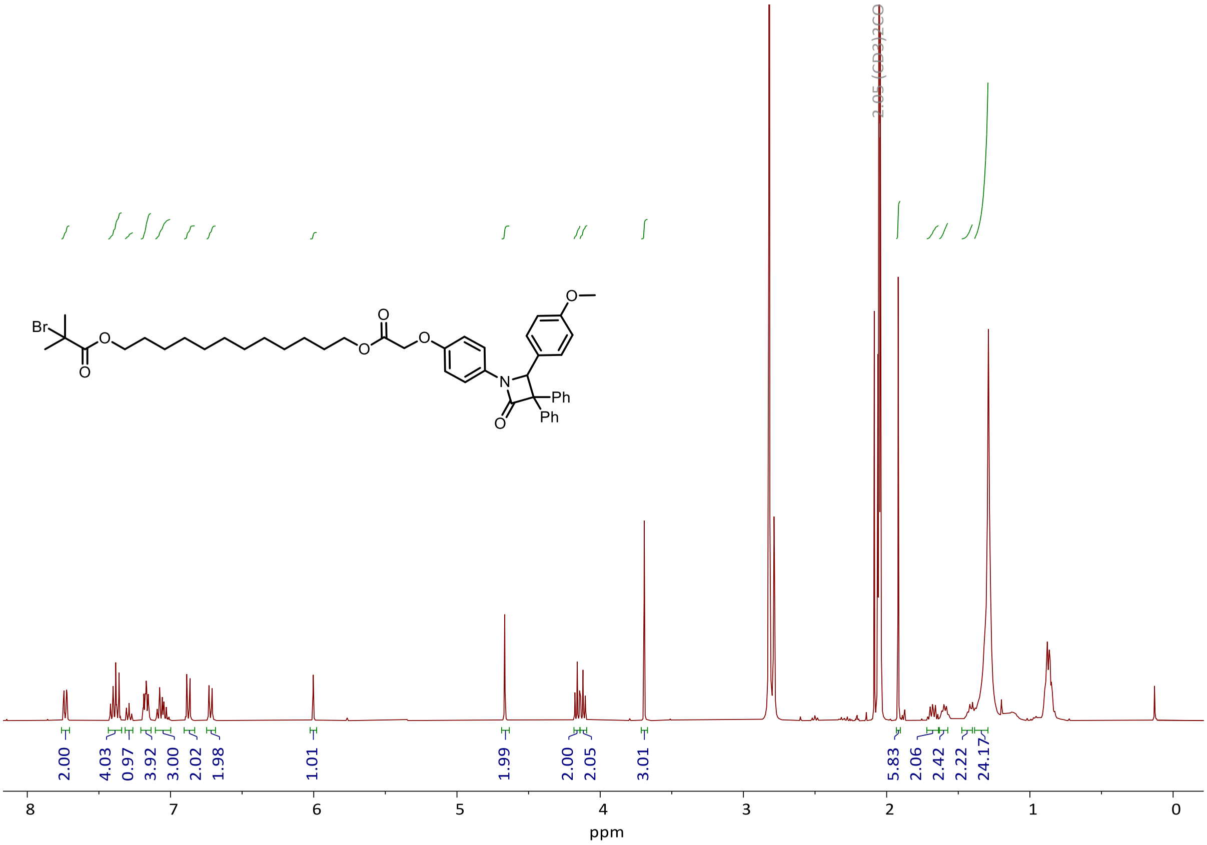

Spectrum S29. ^{1}H NMR (400 MHz, Acetone-d_6, 298 K) spectrum of compound **S14**.

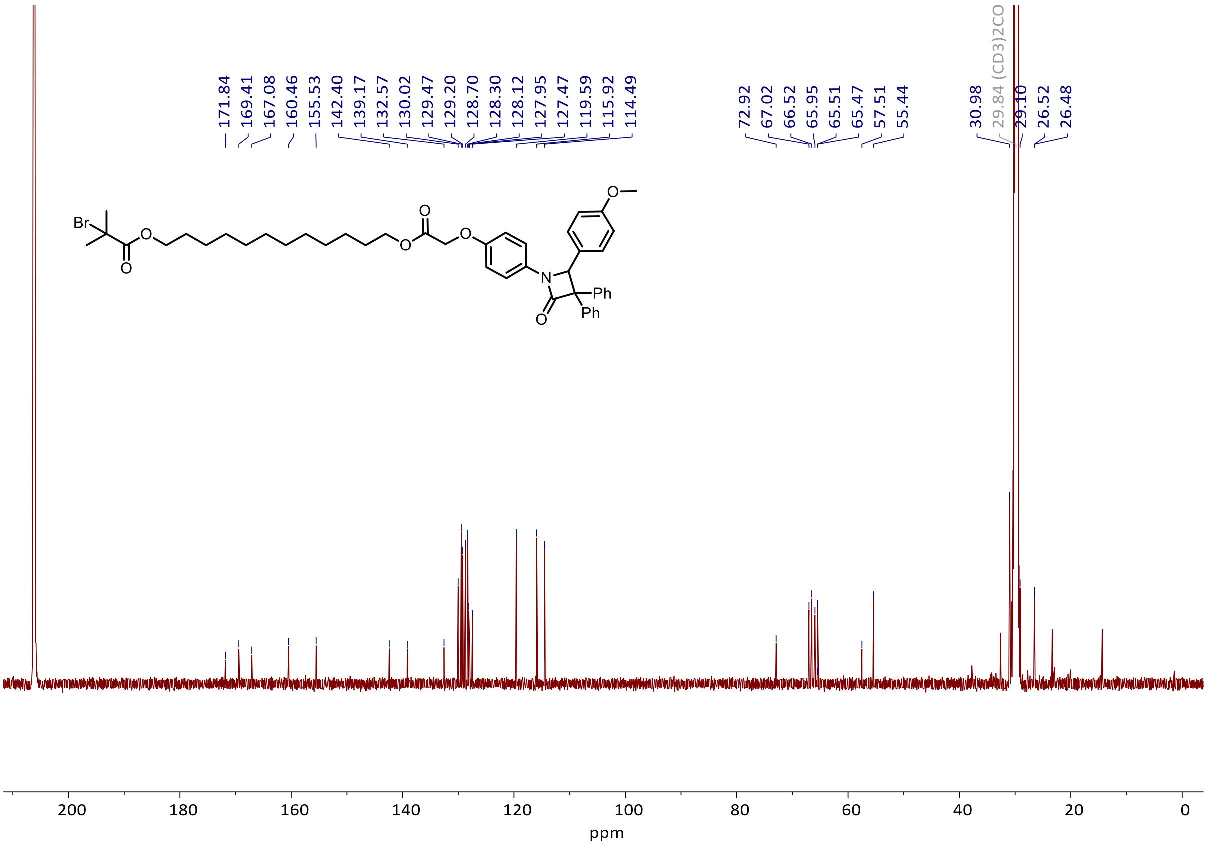

Spectrum S30. ^{13}C NMR (151 MHz, Acetone-d_6, 298 K) spectrum of compound **S14**.

8.1.16 Spectra of S16

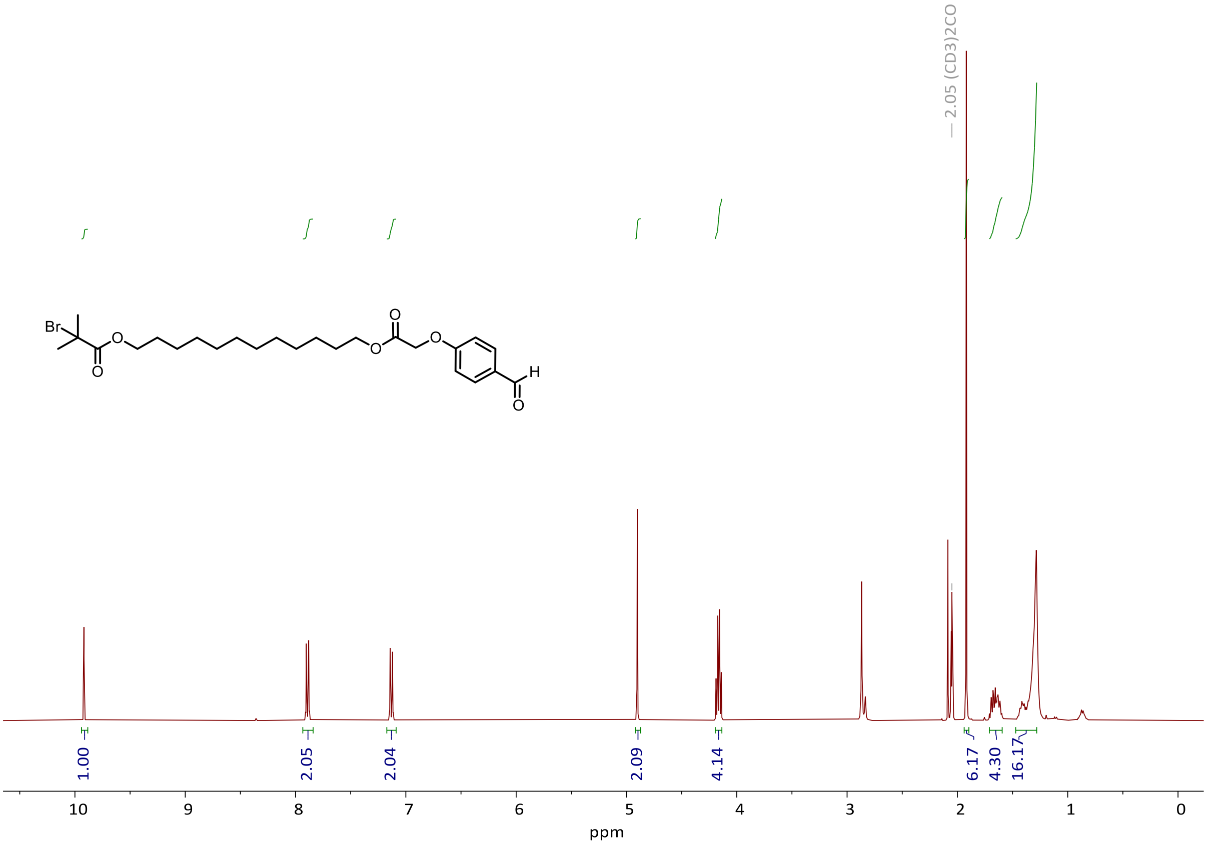

Spectrum S31. ^{1}H NMR (400 MHz, Acetone-d_6, 298 K) spectrum of compound **S16**.

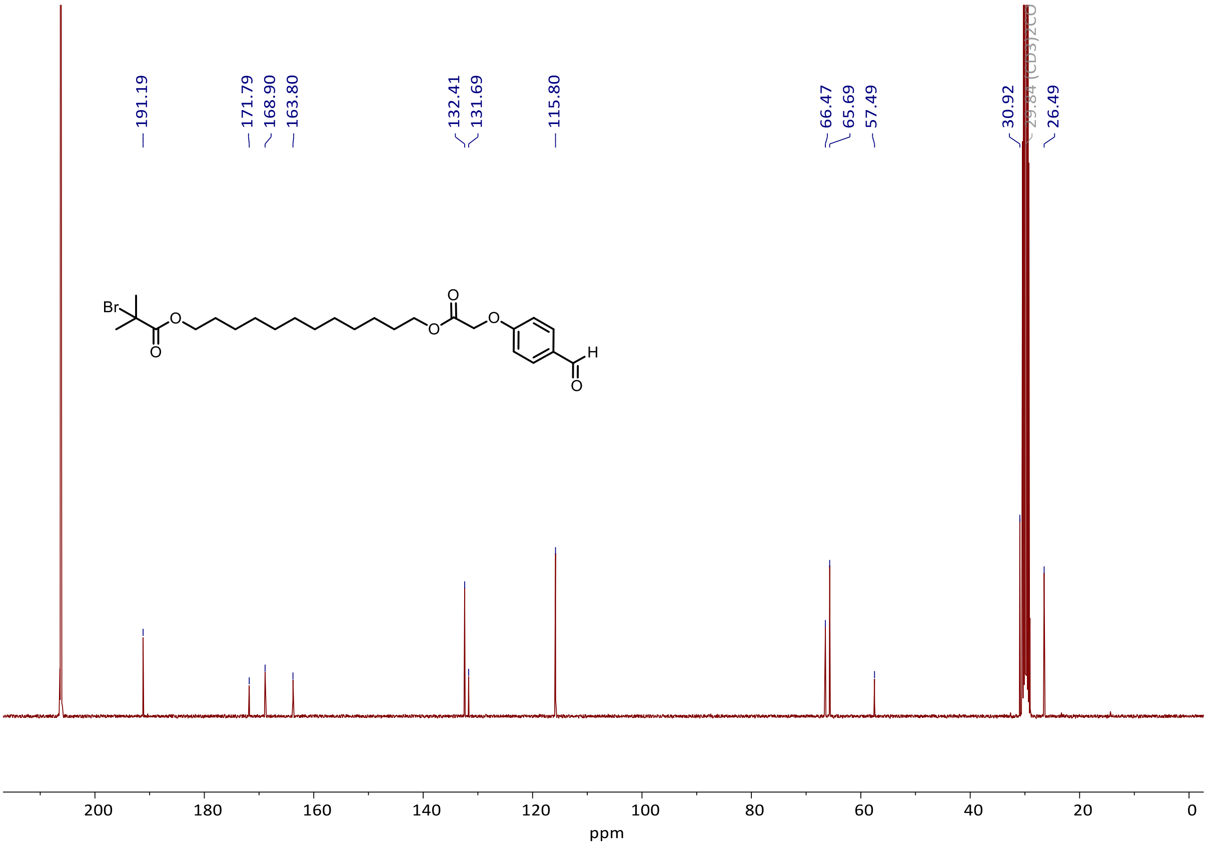

Spectrum S32. ^{13}C NMR (101 MHz, Acetone-d_6, 298 K) spectrum of compound **S16**.

8.1.17 Spectra of S17

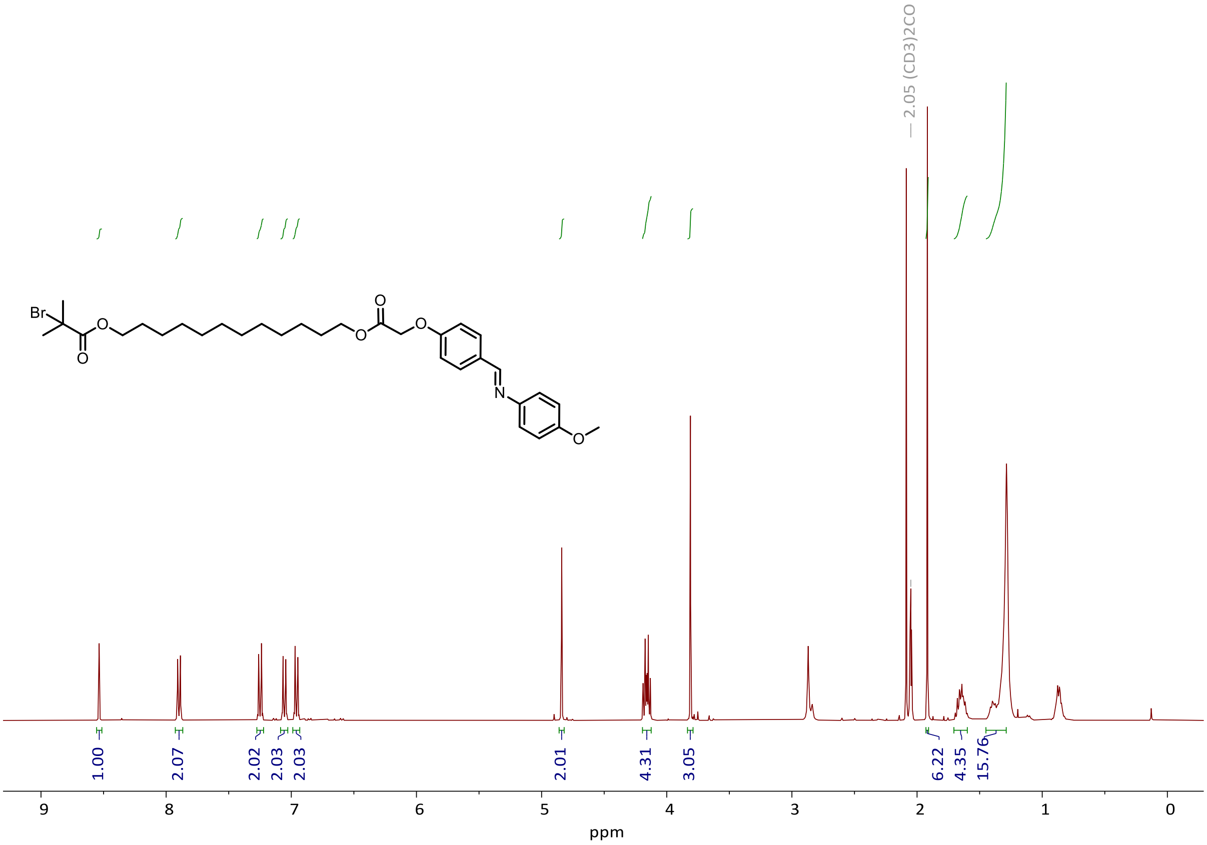

Spectrum S33. ^{1}H NMR (400 MHz, Acetone-d_6, 298 K) spectrum of compound **S17**.

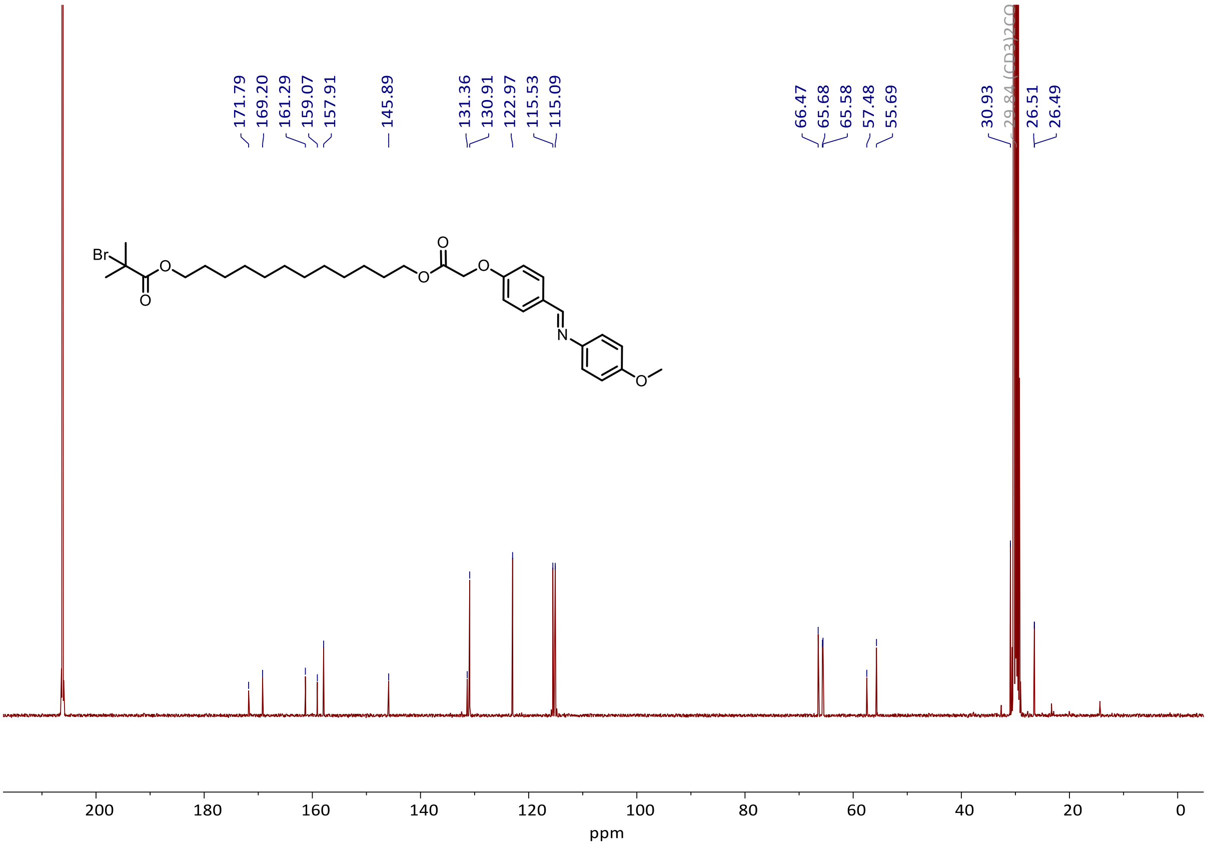

Spectrum S34. ^{13}C NMR (101 MHz, Acetone-d_6, 298 K) spectrum of compound **S17**.

8.2 Polymer NMR Spectra

8.2.1 Spectra of polymer $1_{N\text{-}cis}$

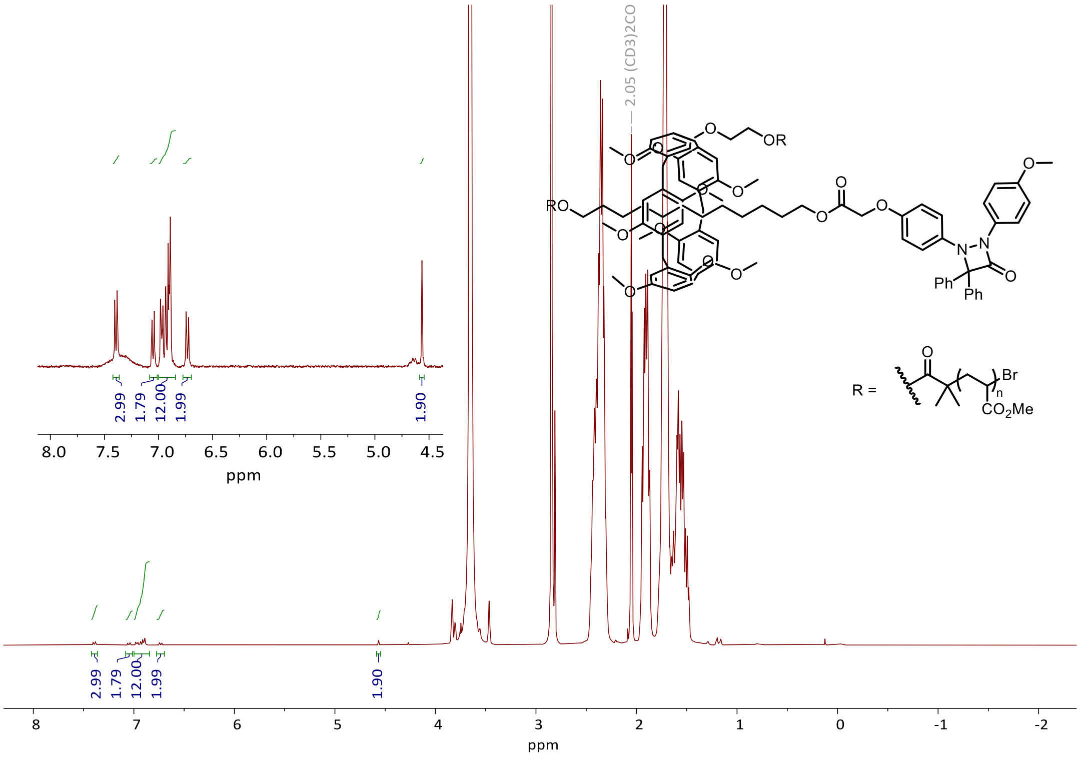

Spectrum S35. ^{1}H NMR (400 MHz, Acetone-d_6, 298 K) spectrum of polymer $\mathbf{1}_{N\text{-}cis}$.

8.2.2 Spectra of polymer $1_{N\text{-}trans}$

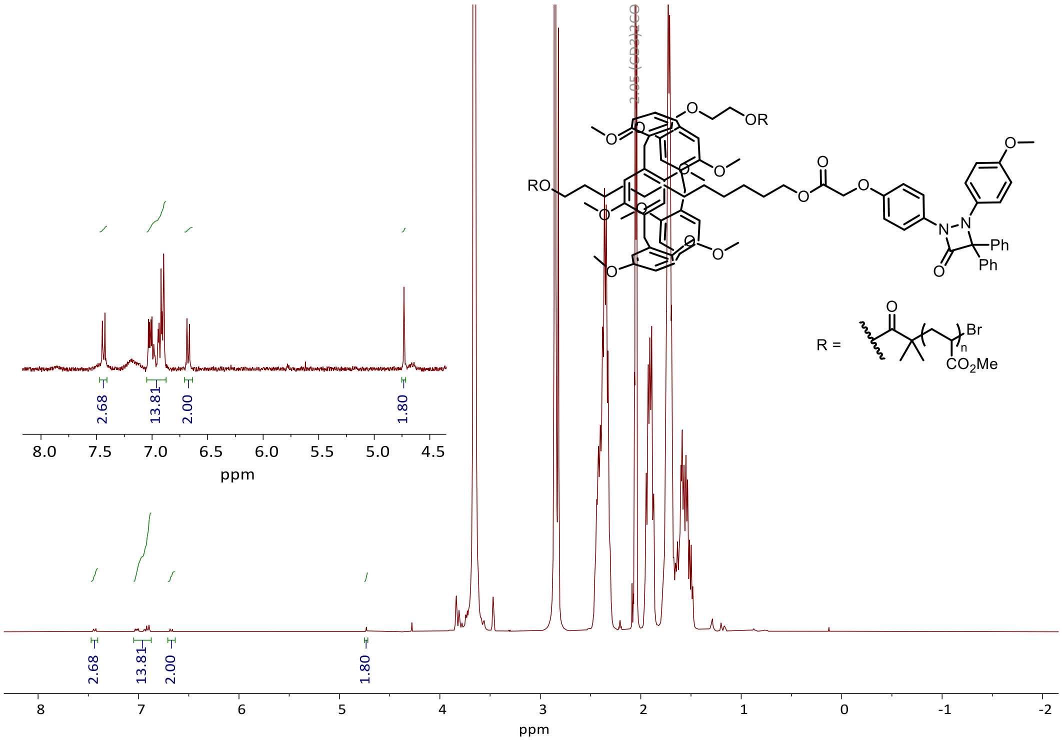

Spectrum S36. ^{1}H NMR (400 MHz, Acetone-d_6, 298 K) spectrum of polymer $\mathbf{1}_{N\text{-}trans}$.

8.2.3 Spectra of polymer $1_{C\text{-}cis}$

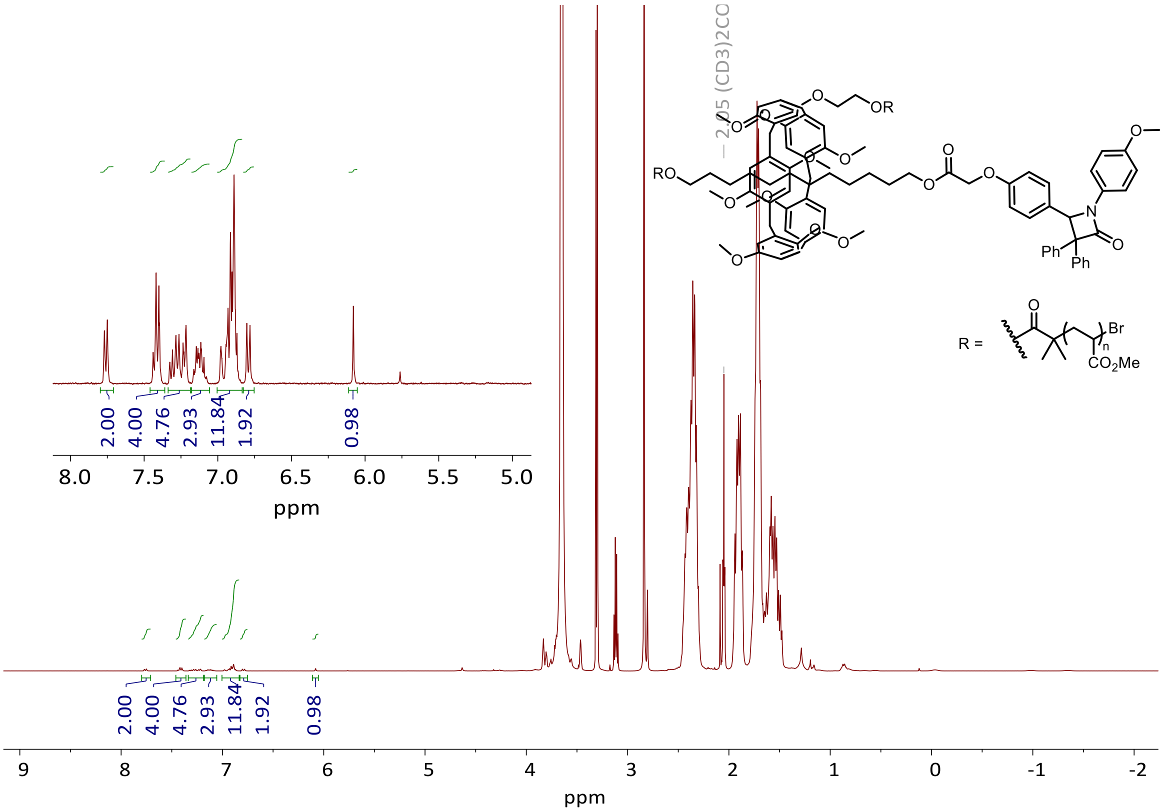

Spectrum S37. ^{1}H NMR (400 MHz, Acetone-d_6, 298 K) spectrum of polymer $\mathbf{1}_{\mathbf{C}\text{-}\boldsymbol{cis}}$.

8.2.4 Spectra of polymer $1_{C\text{-}trans}$

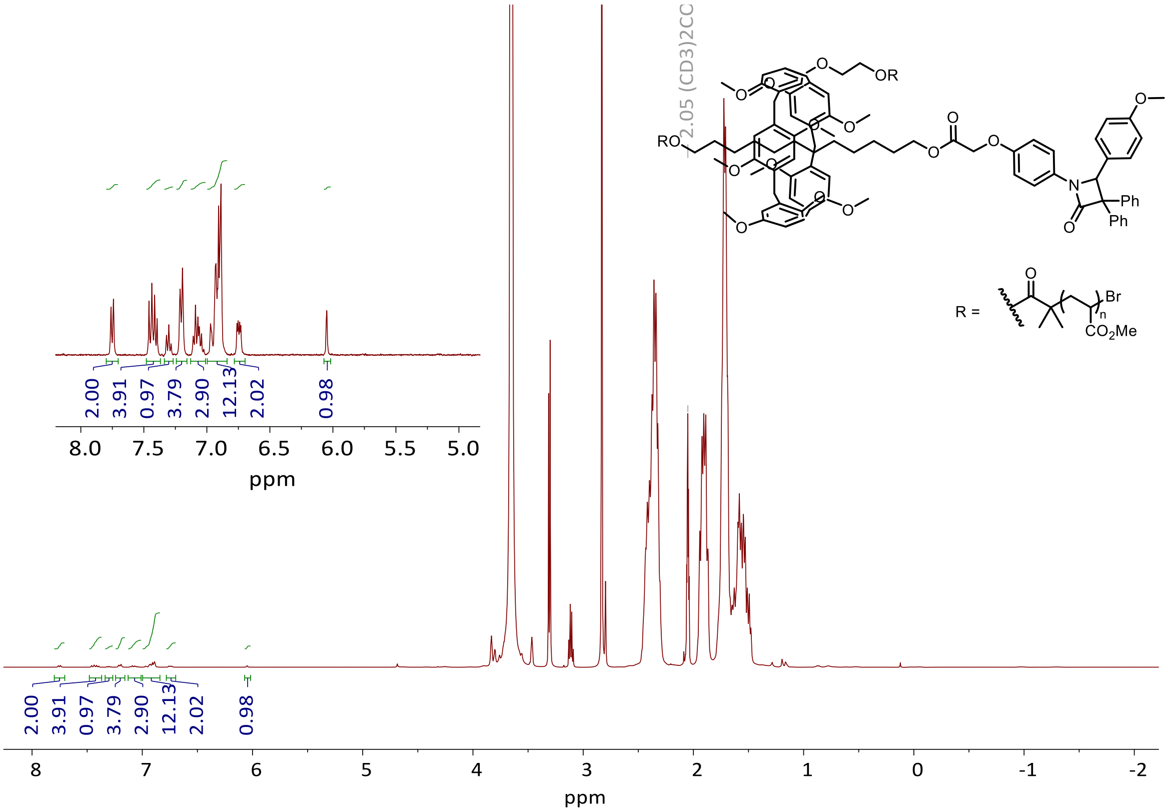

Spectrum S38. ^{1}H NMR (400 MHz, Acetone-d_6, 298 K) spectrum of polymer $\mathbf{1}_{\mathbf{C}\text{-}\boldsymbol{trans}}$.

8.2.5 Spectra of polymer S18

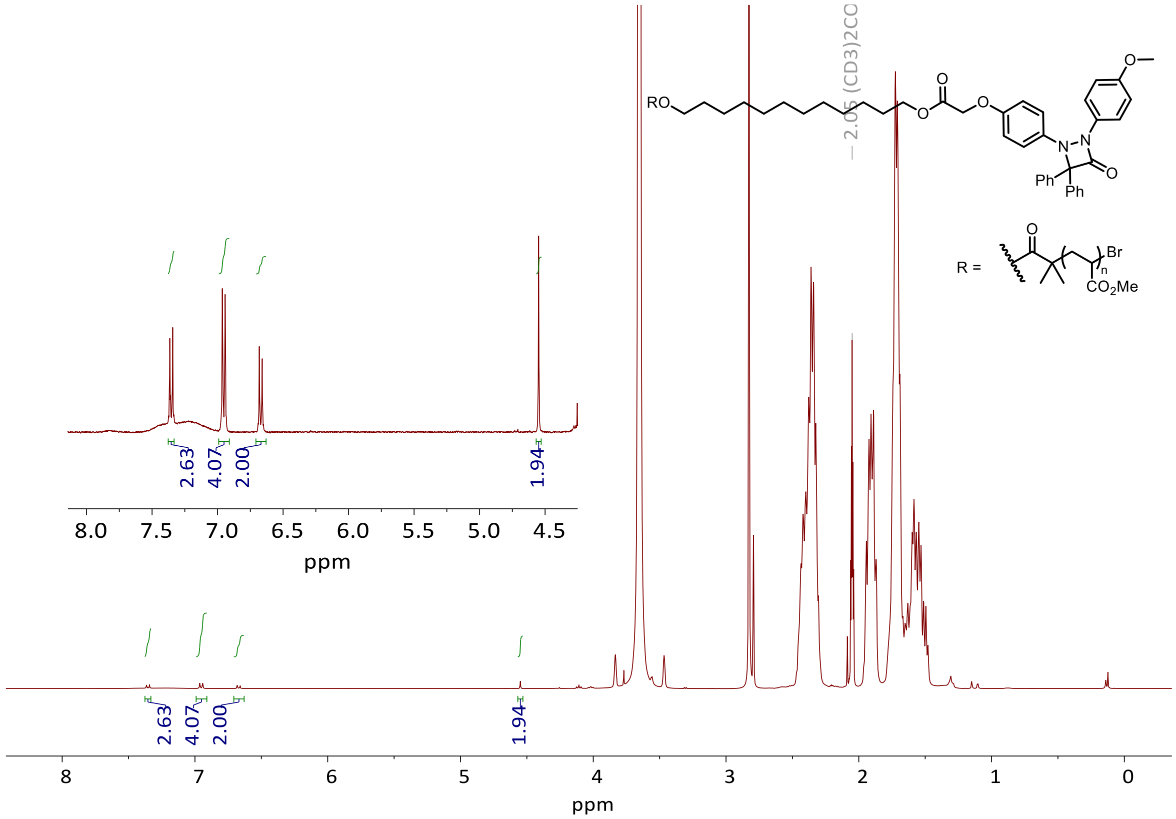

Spectrum S39. ^{1}H NMR (400 MHz, Acetone-d_6, 298 K) spectrum of polymer **S18**.

8.2.6 Spectra of polymer S19

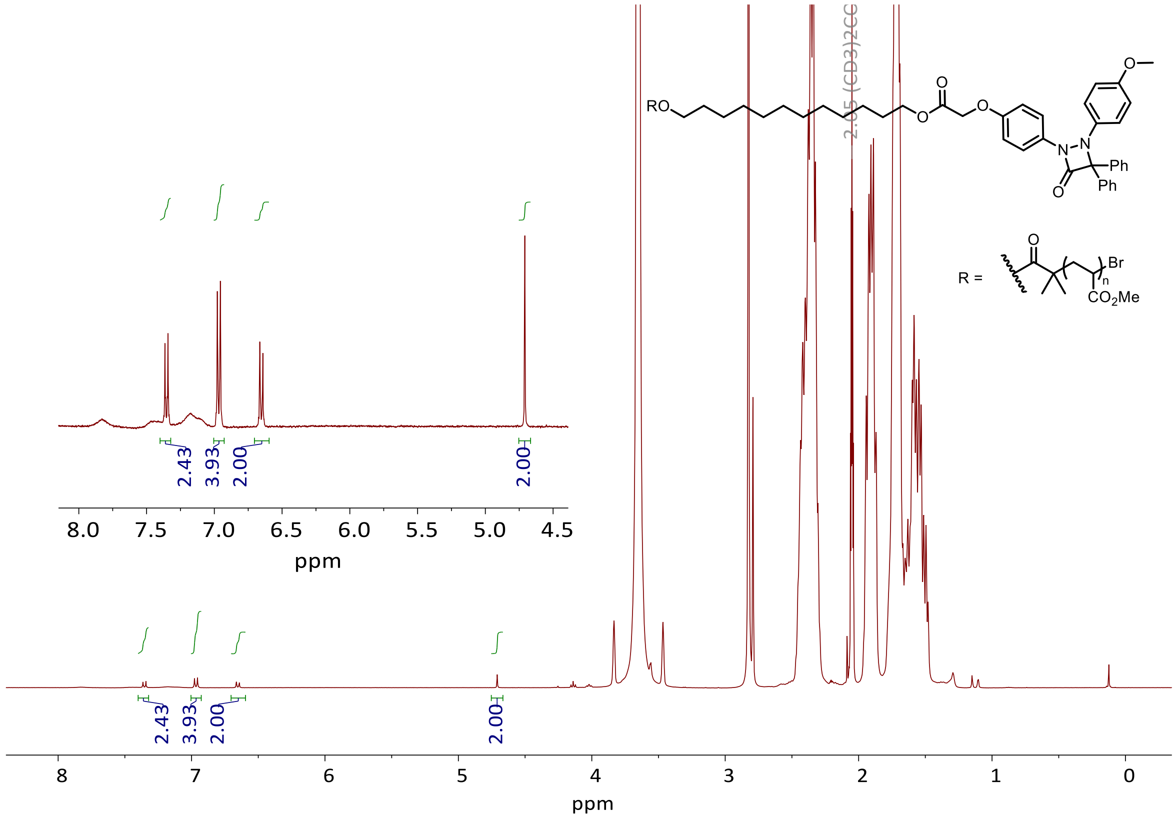

Spectrum S40. ^{1}H NMR (400 MHz, Acetone-d_6, 298 K) spectrum of polymer **S19**.

8.2.7 Spectra of polymer S20

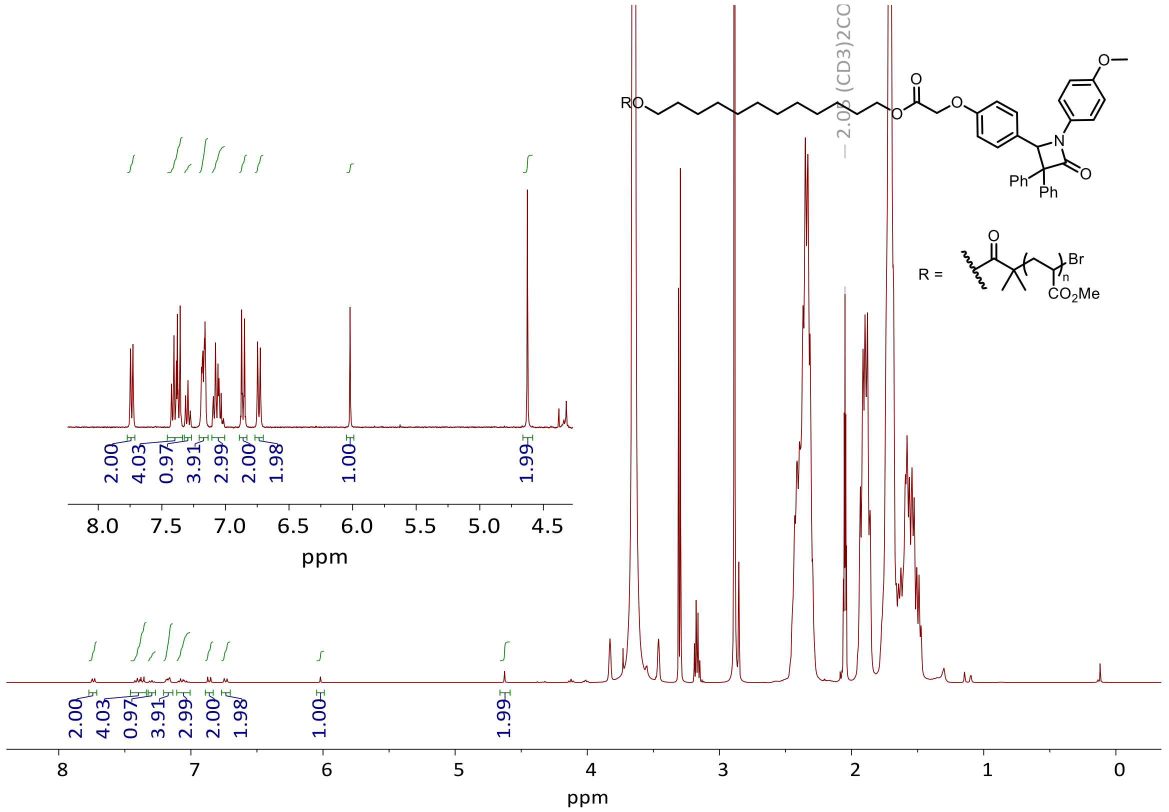

Spectrum S41. ^{1}H NMR (400 MHz, Acetone-d_6, 298 K) spectrum of polymer **S20**.

8.2.8 Spectra of polymer S21

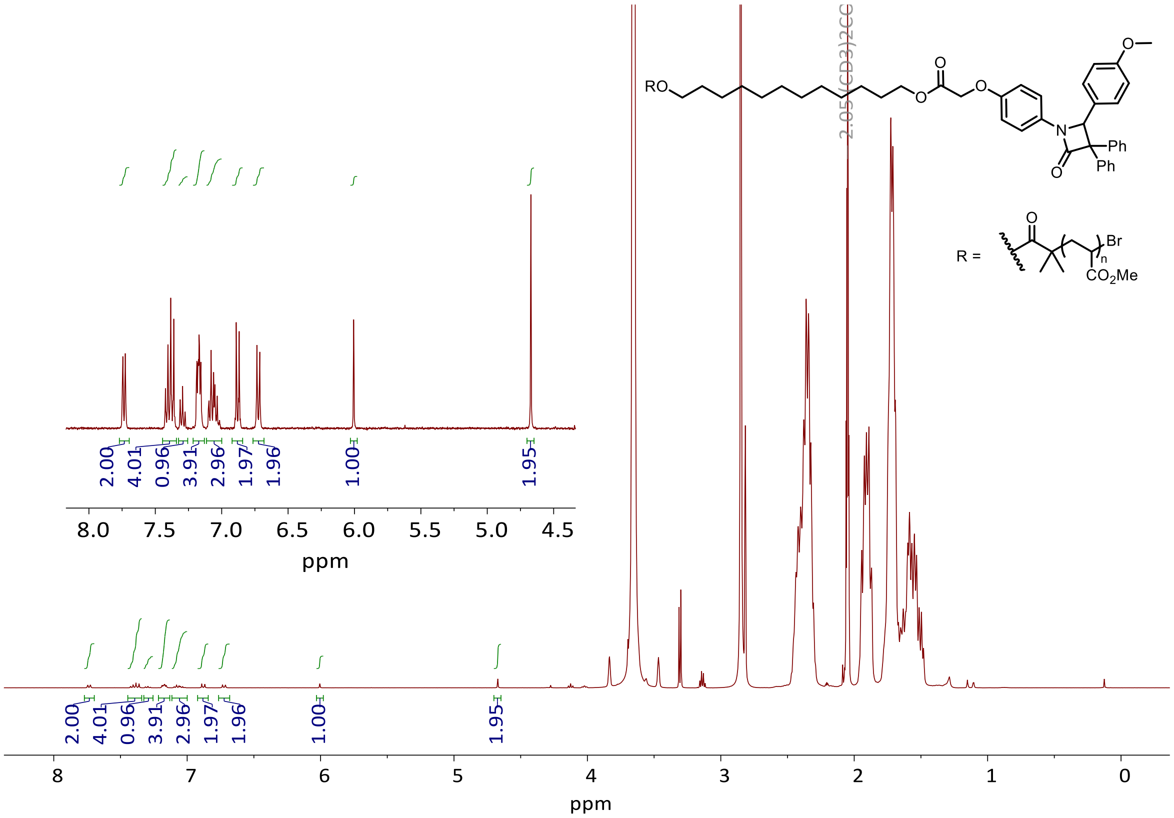

Spectrum S42. ^{1}H NMR (400 MHz, Acetone-d_6, 298 K) spectrum of polymer **S21**.

8.2.9 Spectra of polymer S22

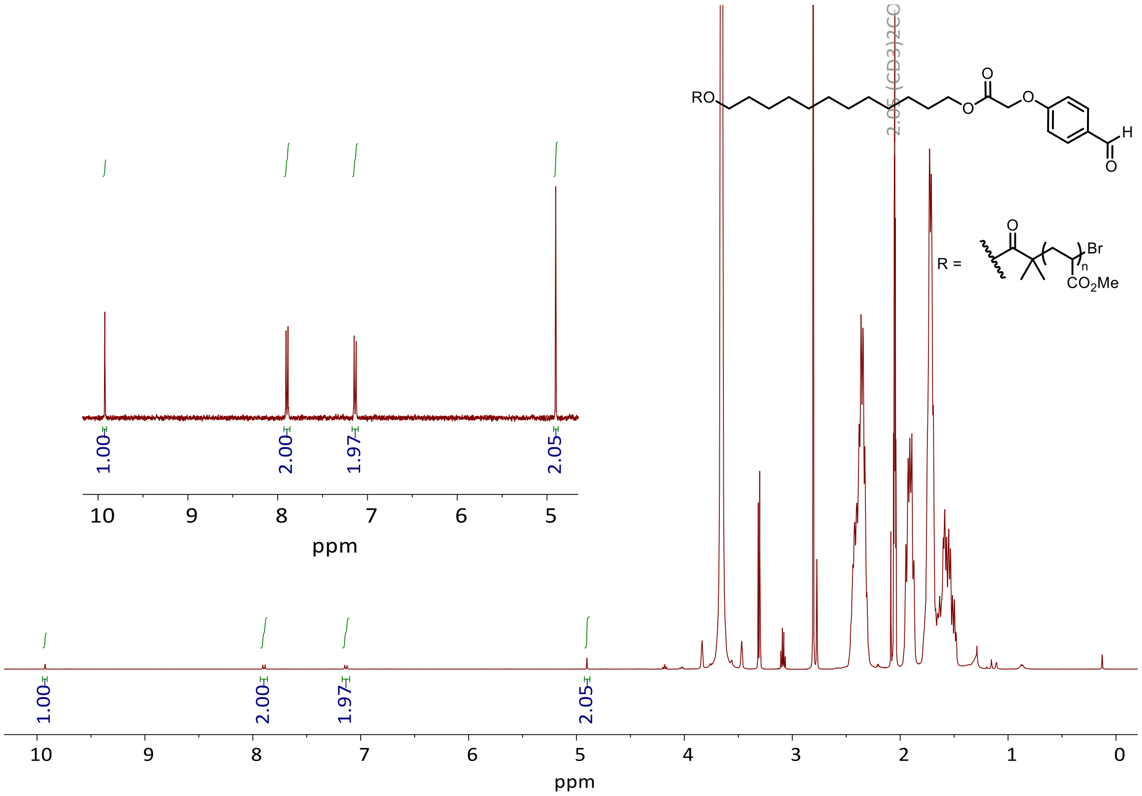

Spectrum S43. ^{1}H NMR (400 MHz, Acetone-d_6, 298 K) spectrum of polymer **S22**.

8.2.10 Spectra of polymer 4_C

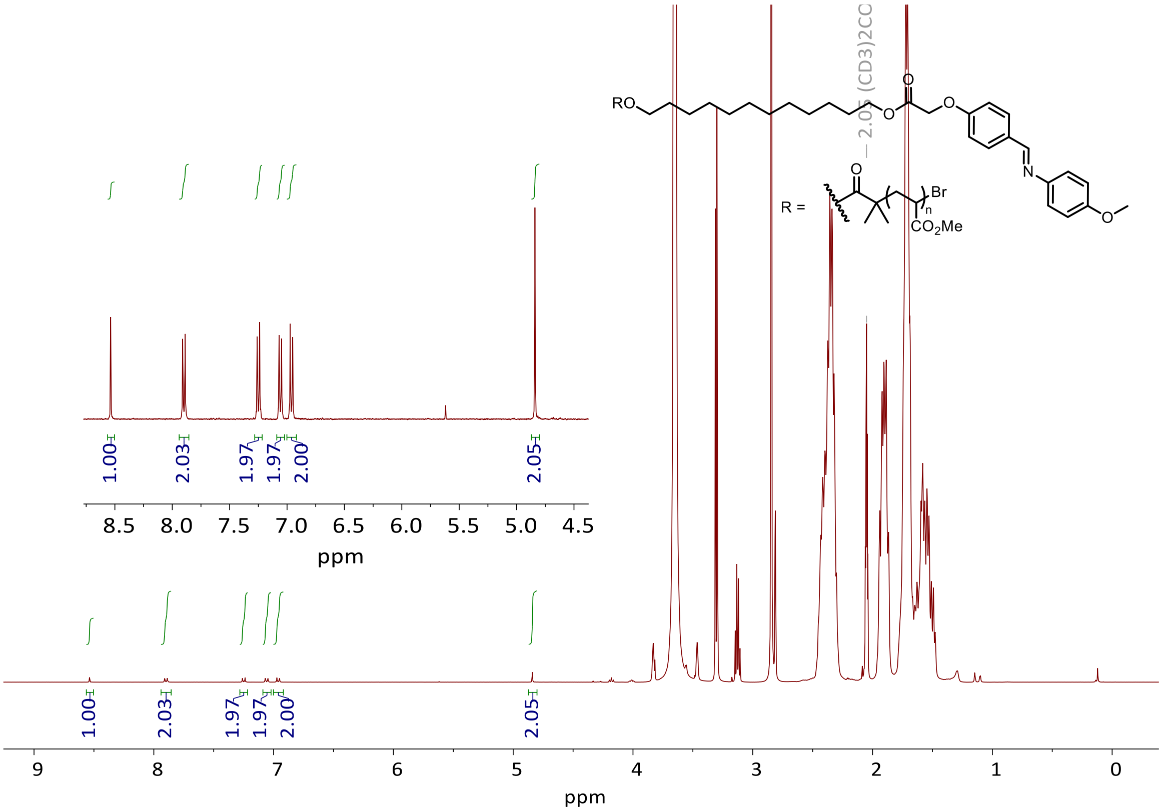

Spectrum S44. ^{1}H NMR (400 MHz, Acetone-d_6, 298 K) spectrum of polymer **4C**.

8.3 Post-Sonication NMR Spectra

8.3.1 Post-Sonication ^{1}H NMR Spectra of Polymer $\mathbf{1}_{\mathbf{N}\text{-}\boldsymbol{cis}}$ (Run 1)

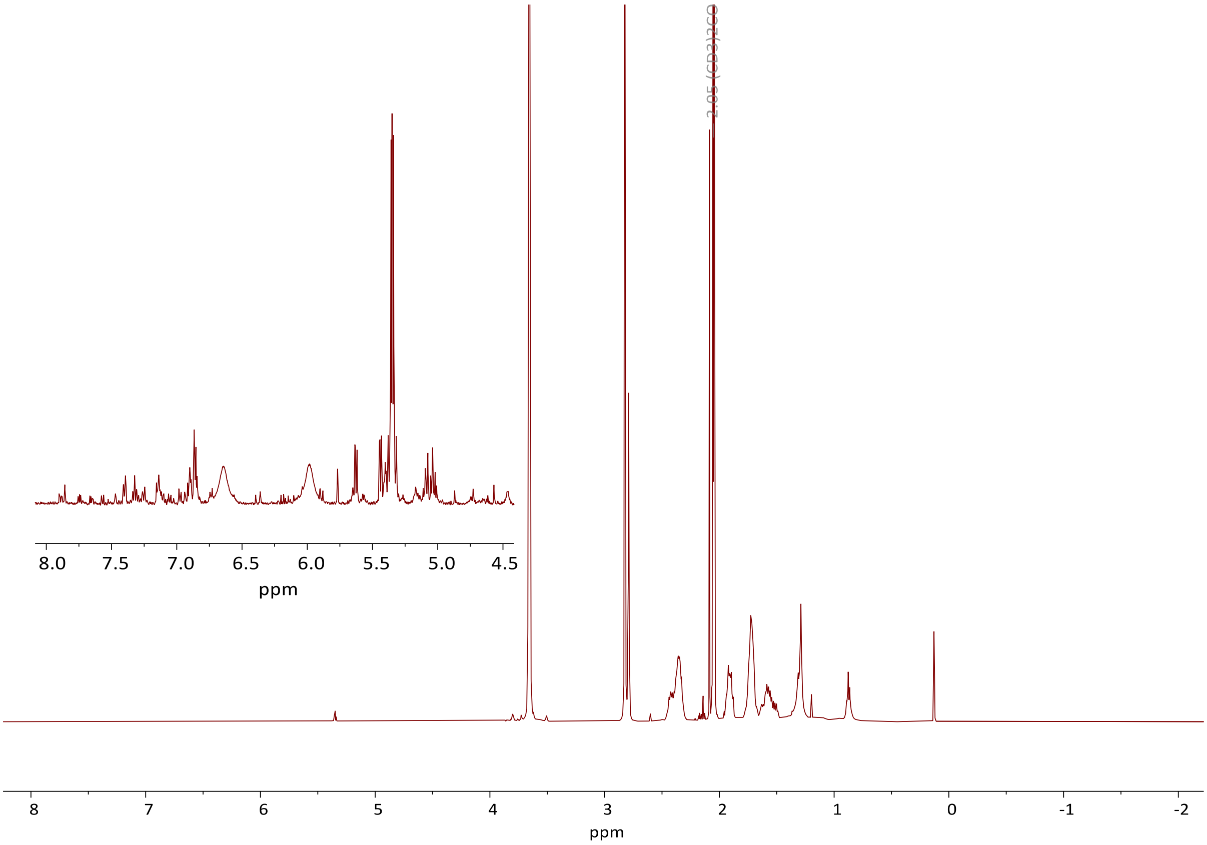

Spectrum S45. ^{1}H NMR (500 MHz, Acetone-d_6, 298 K) spectrum of post-sonication polymer $\mathbf{1}_{\mathbf{N}\text{-}\boldsymbol{cis}}$ before being washed with methanol.

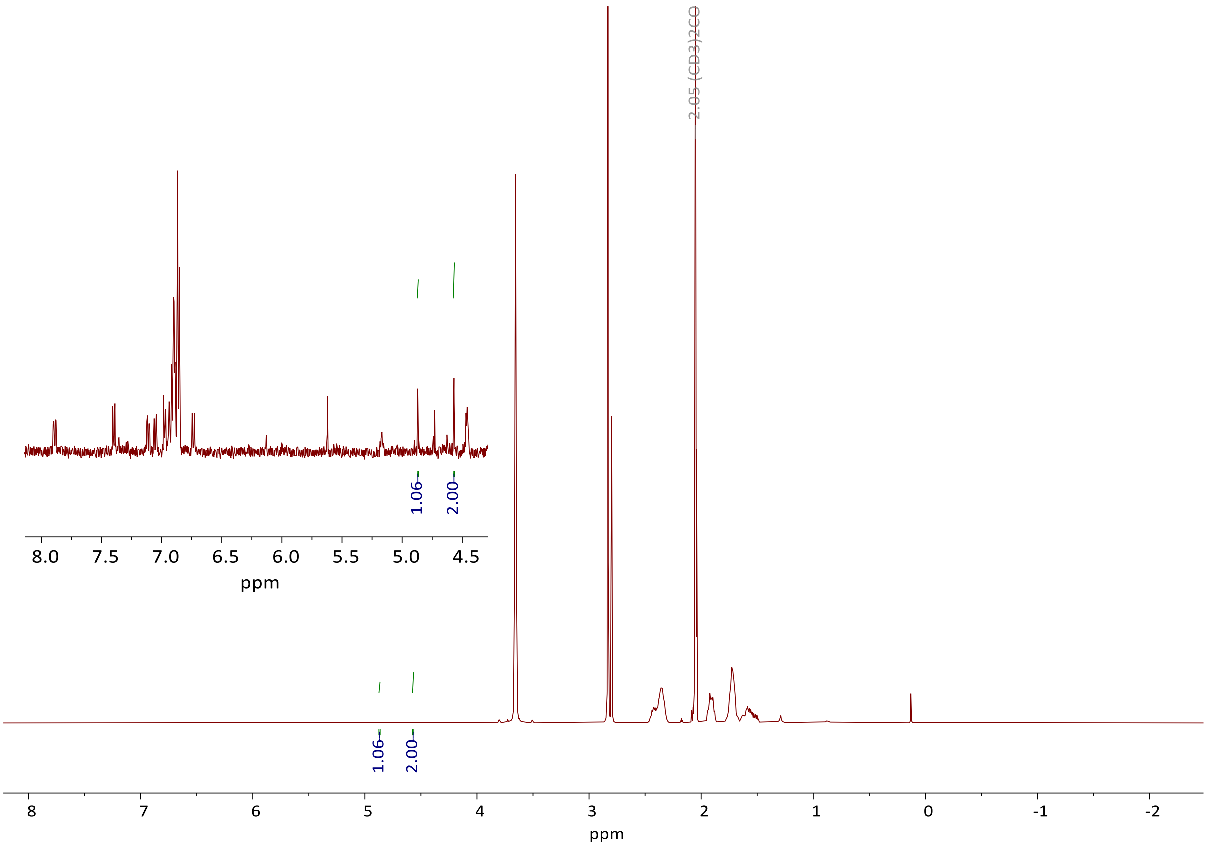

Spectrum S46. ^{1}H NMR (500 MHz, Acetone-d_6, 298 K) spectrum of post-sonication polymer **1**$_{\mathbf{N}\text{-}\boldsymbol{cis}}$ after being washed with methanol.

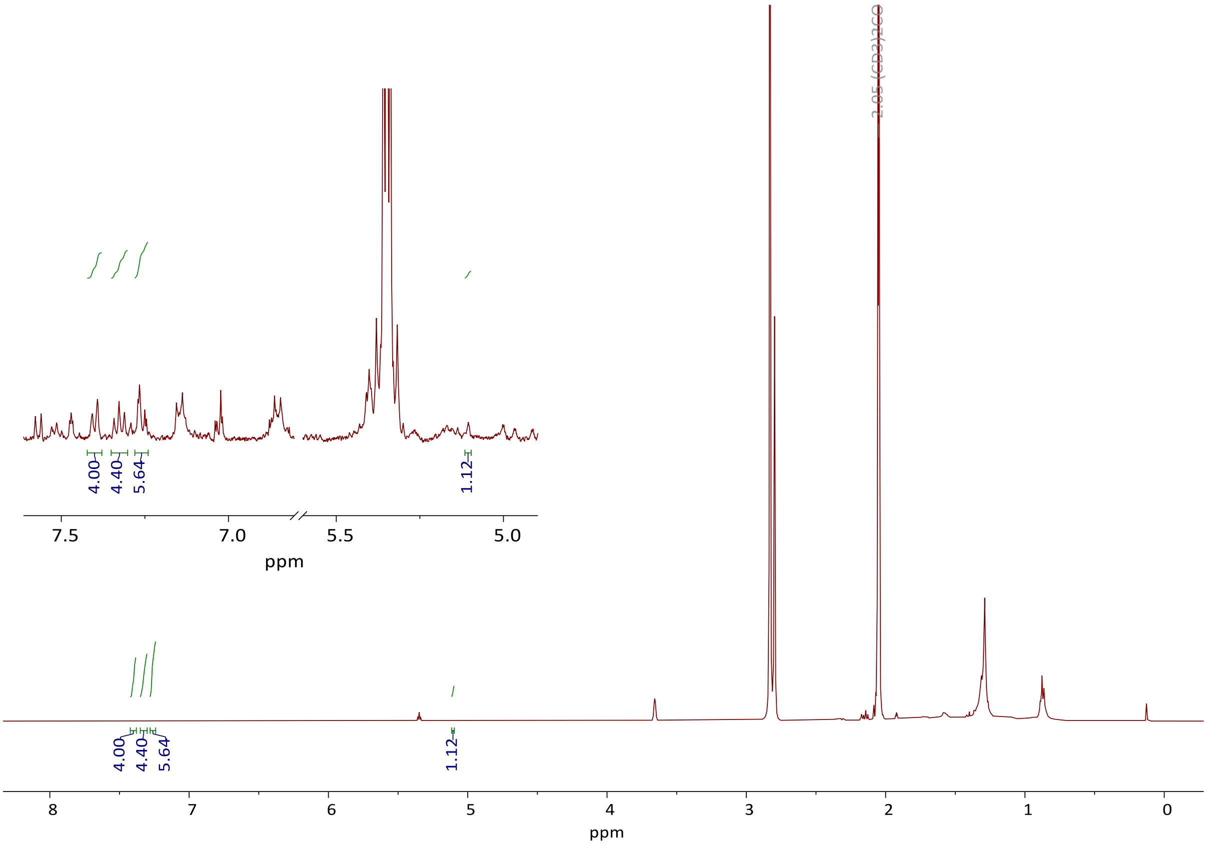

Spectrum S47. ^{1}H NMR (500 MHz, Acetone-d_6, 298 K) spectrum of the concentrated methanol washings from post-sonication polymer **1**$_{\mathbf{N}\text{-}\boldsymbol{cis}}$.

8.3.2 Post-Sonication ^{1}H NMR Spectra of Polymer $\mathbf{1}_{\mathbf{N}\text{-}\boldsymbol{cis}}$ (Run 2)

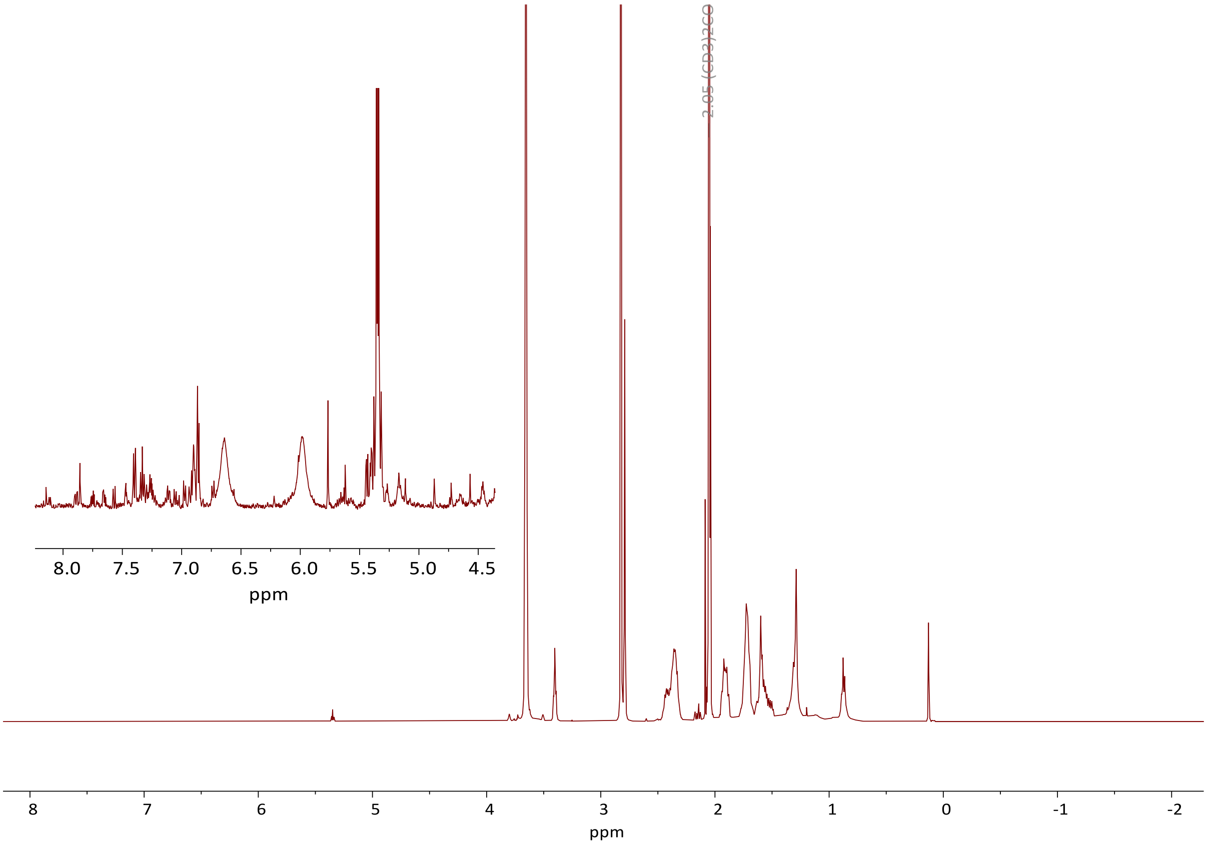

Spectrum S48. ^{1}H NMR (500 MHz, Acetone-d_6, 298 K) spectrum of post-sonication polymer $\mathbf{1}_{\mathbf{N}\text{-}\boldsymbol{cis}}$ before being washed with methanol.

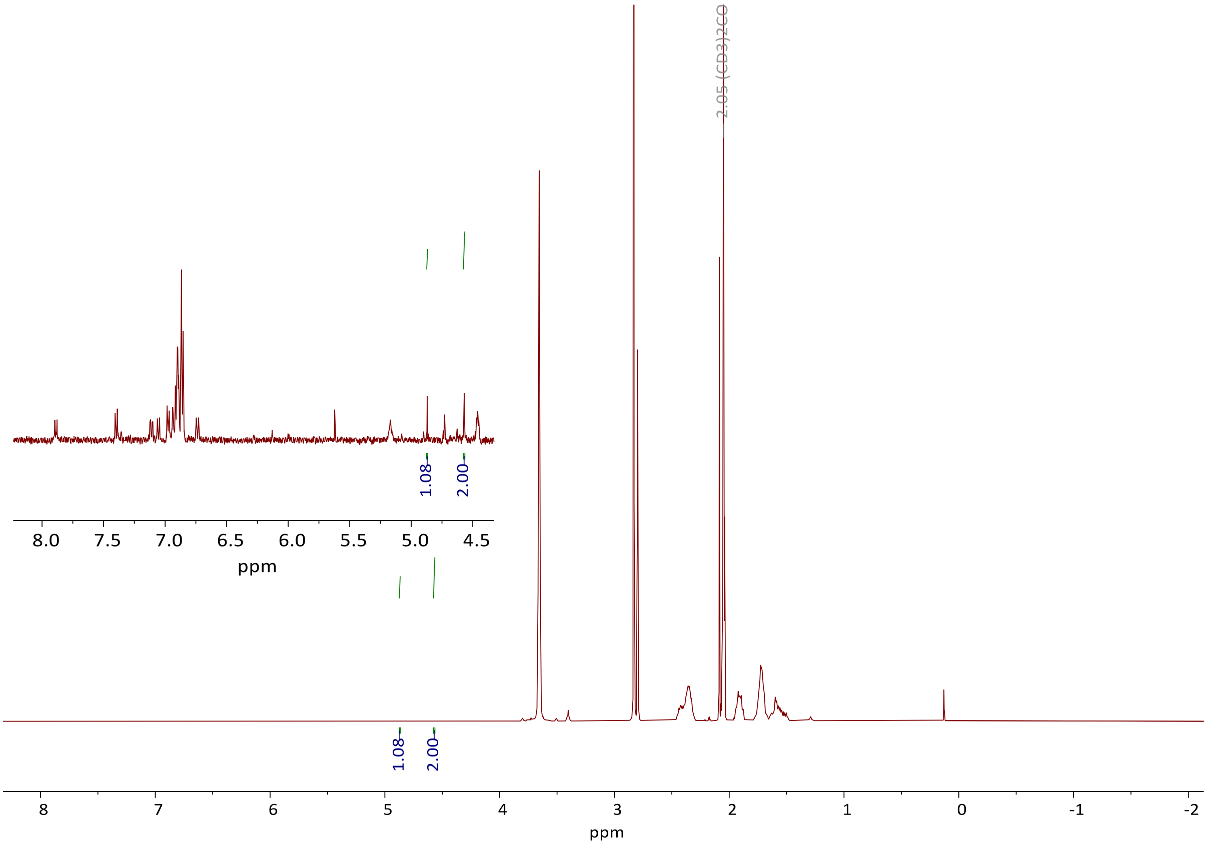

Spectrum S49. ^{1}H NMR (500 MHz, Acetone-d_6, 298 K) spectrum of post-sonication polymer $\mathbf{1}_{\mathbf{N}\text{-}\boldsymbol{cis}}$ after being washed with methanol.

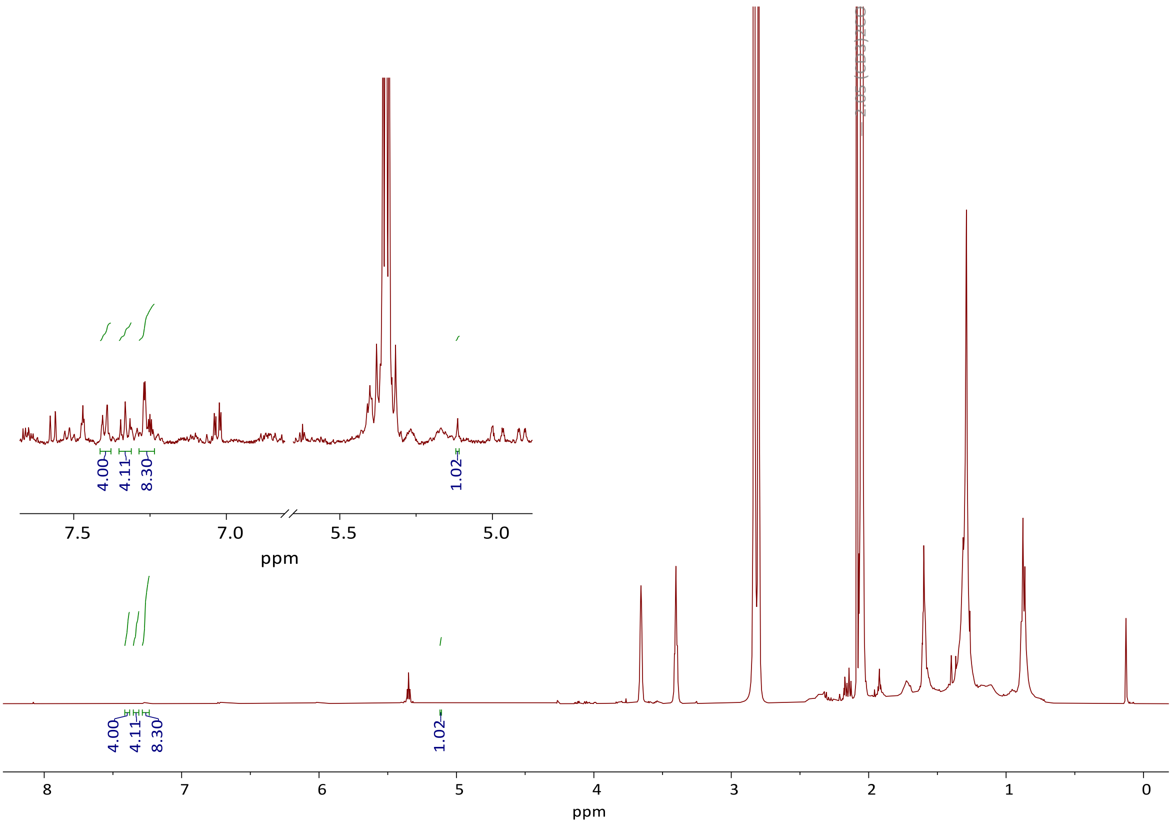

Spectrum S50. ^{1}H NMR (500 MHz, Acetone-d_6, 298 K) spectrum of the concentrated methanol washings from post-sonication polymer $\mathbf{1}_{\text{N-}cis}$.

8.3.3 Post-Sonication ^{1}H NMR Spectra of Polymer $\mathbf{1}_{\text{N-}trans}$ (Run 1)

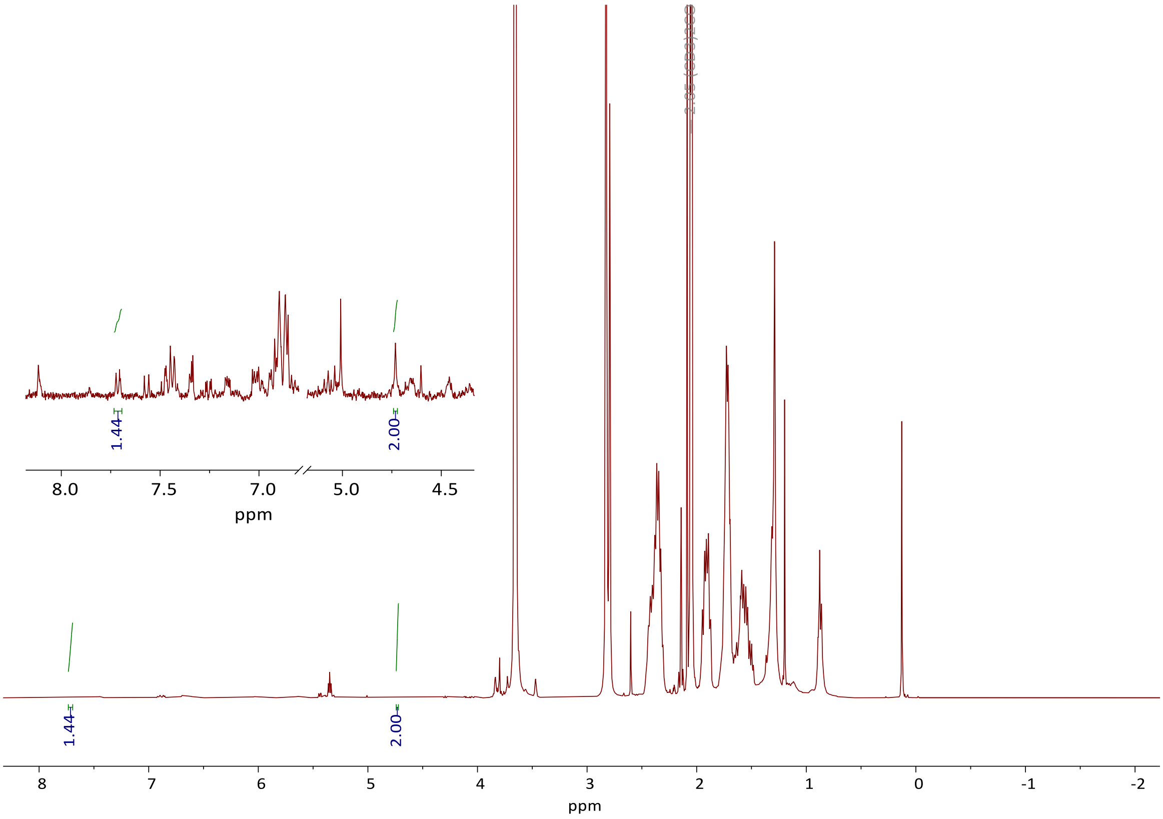

Spectrum S51. ^{1}H NMR (400 MHz, Acetone-d_6, 298 K) spectrum of post-sonication polymer $\mathbf{1}_{\text{N-}trans}$ before being washed with methanol.

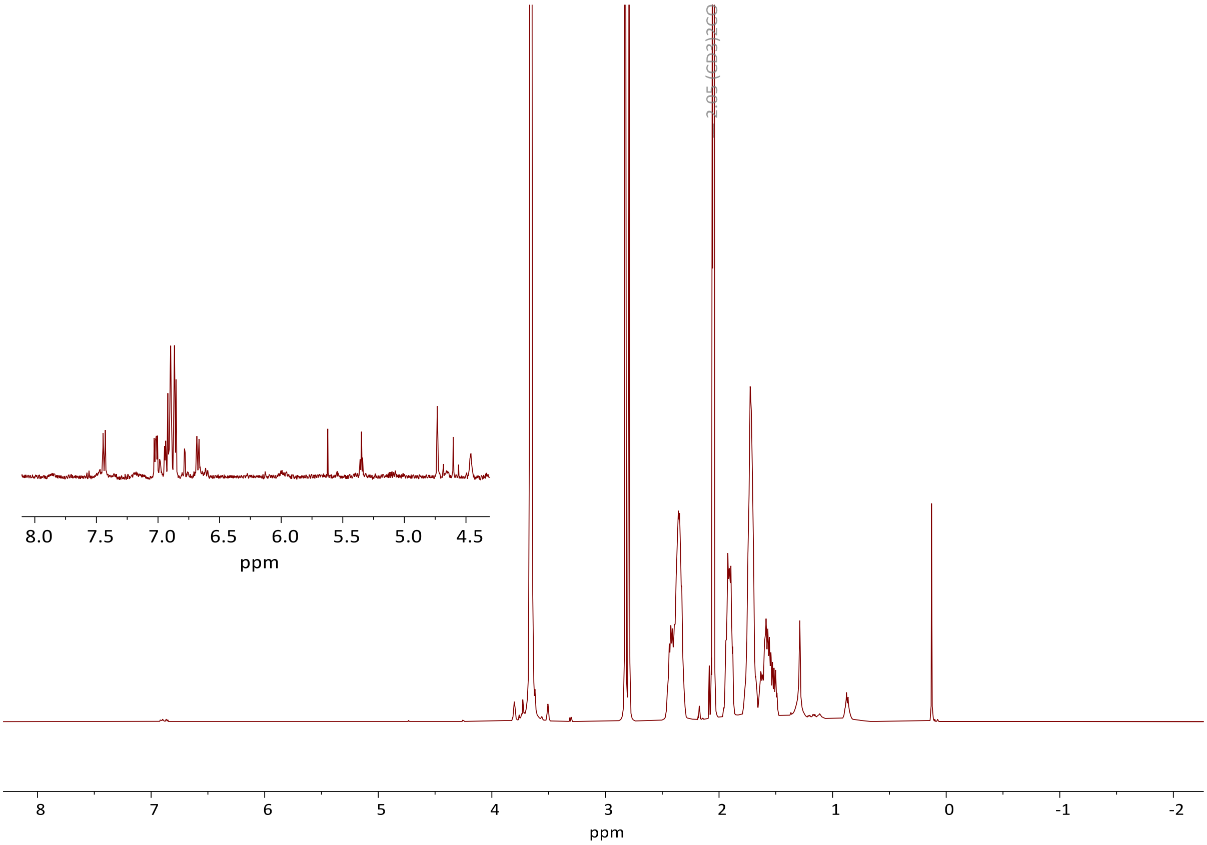

Spectrum S52. ^{1}H NMR (500 MHz, Acetone-d_6, 298 K) spectrum of post-sonication polymer $\mathbf{1}_{\mathbf{N}\text{-}\boldsymbol{trans}}$ after being washed with methanol.

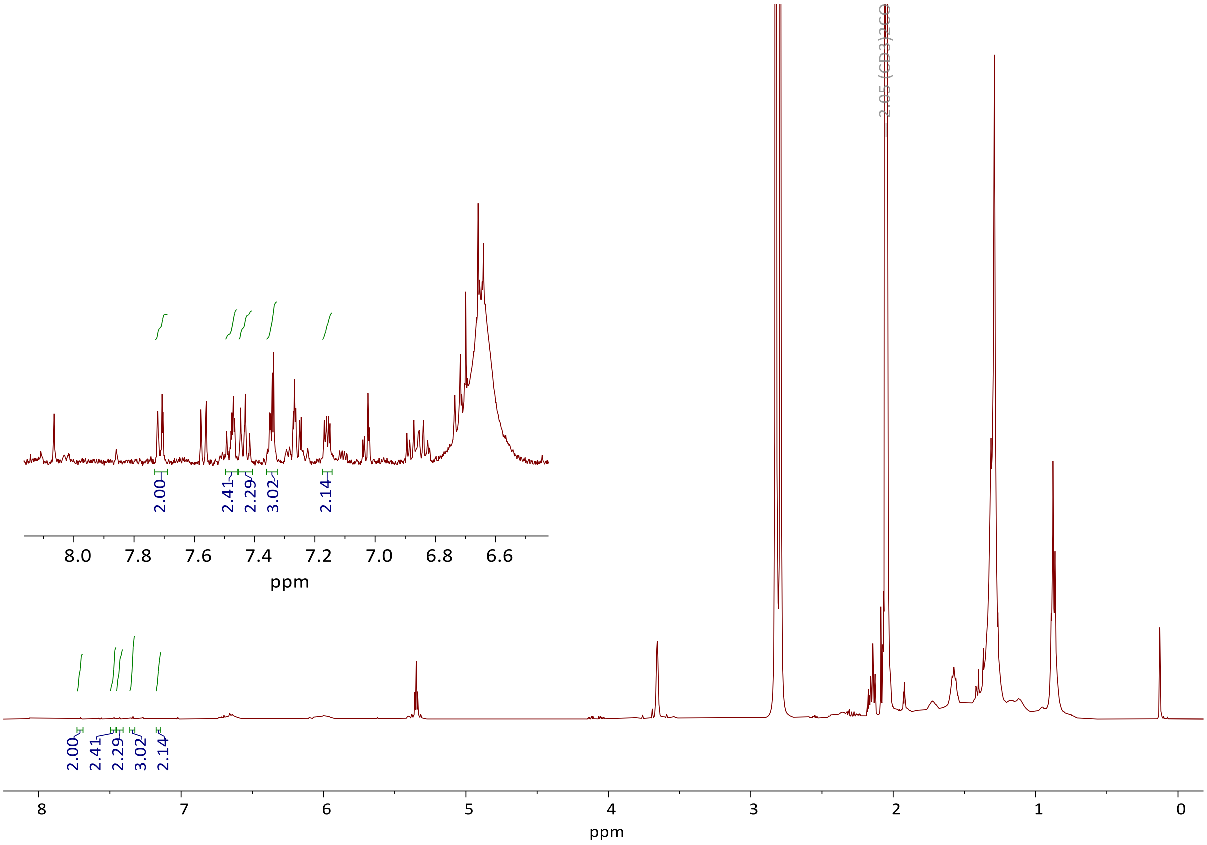

Spectrum S53. ^{1}H NMR (500 MHz, Acetone-d_6, 298 K) spectrum of the concentrated methanol washings from post-sonication polymer $\mathbf{1}_{\mathbf{N}\text{-}\boldsymbol{trans}}$.

8.3.4 Post-Sonication ^{1}H NMR Spectra of Polymer $1_{N\text{-}trans}$ (Run 2)

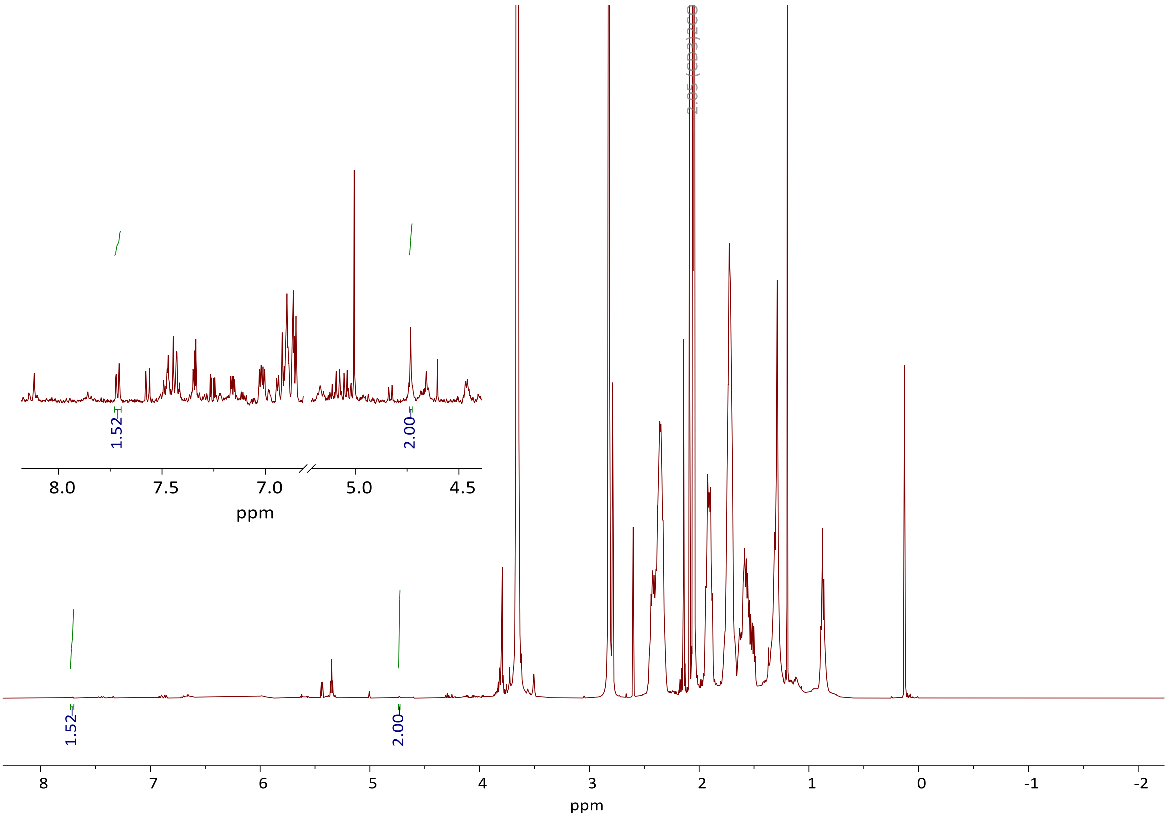

Spectrum S54. ^{1}H NMR (500 MHz, Acetone-d_6, 298 K) spectrum of post-sonication polymer $\mathbf{1}_{N\text{-}trans}$ before being washed with methanol.

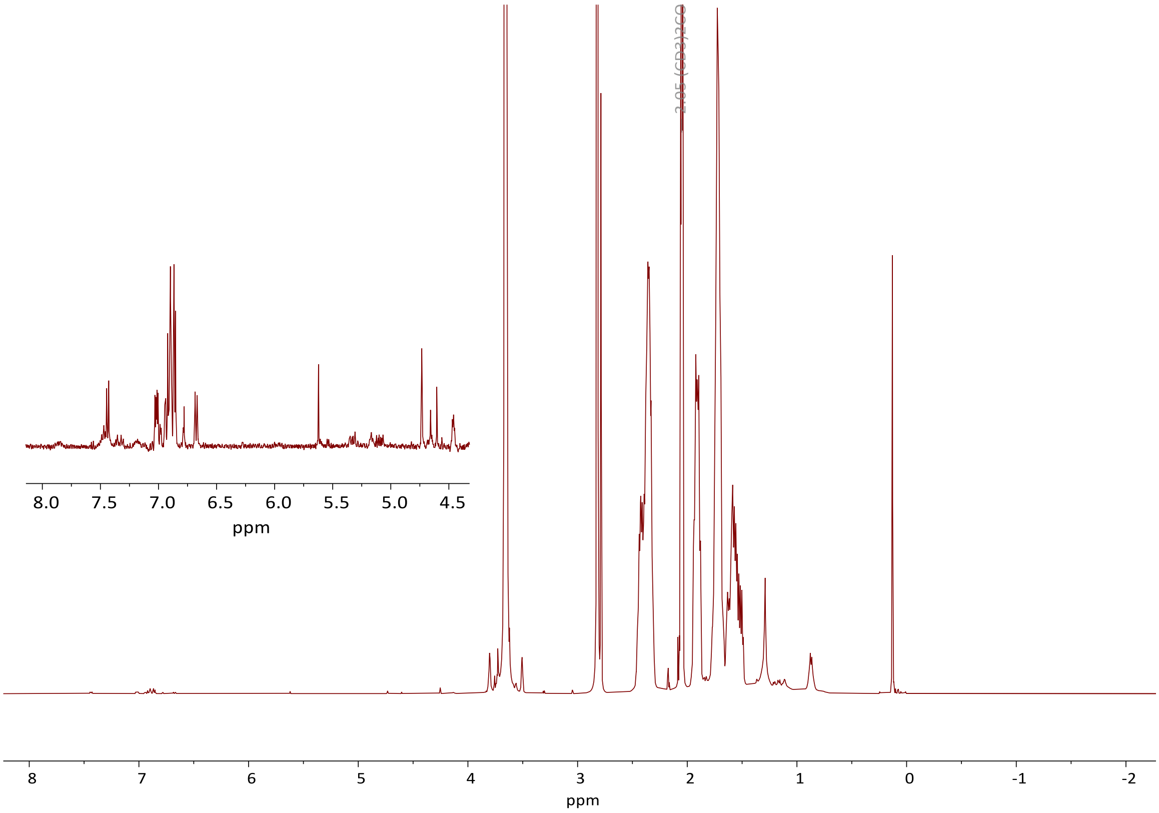

Spectrum S55. ^{1}H NMR (500 MHz, Acetone-d_6, 298 K) spectrum of post-sonication polymer $\mathbf{1}_{N\text{-}trans}$ after being washed with methanol.

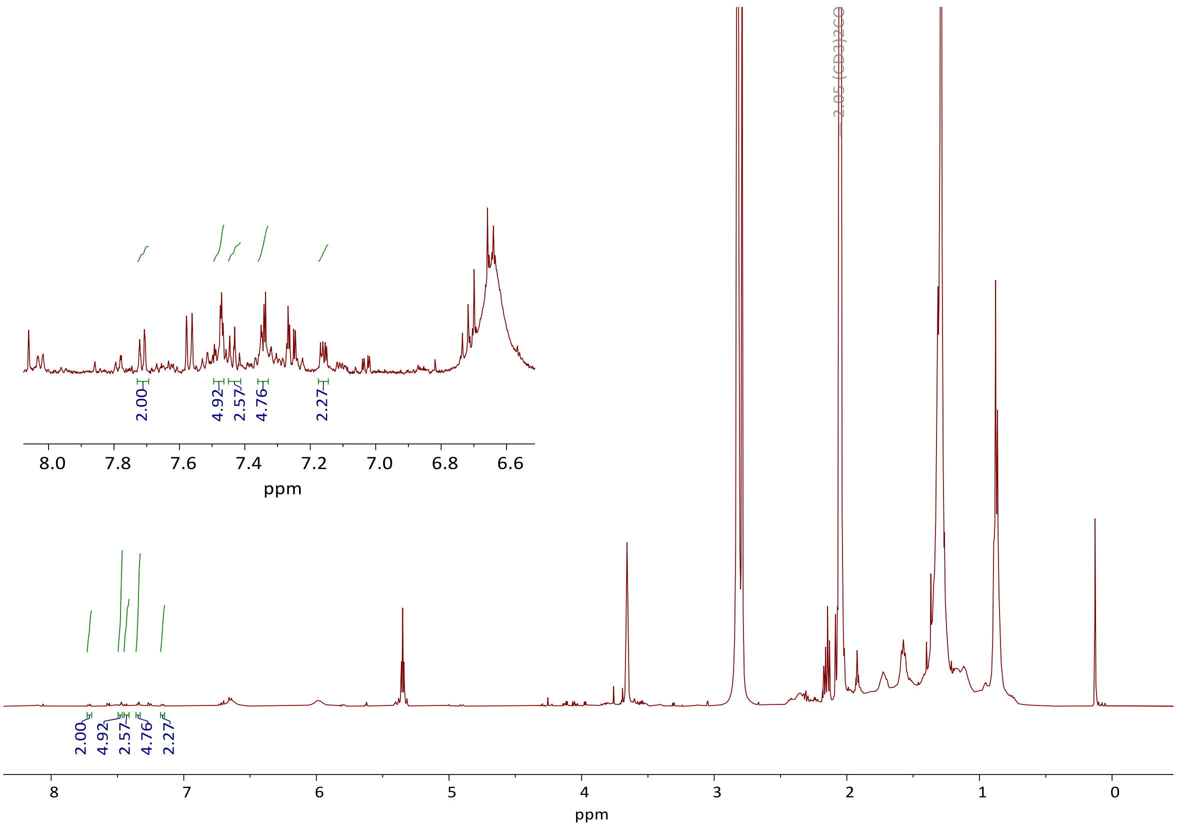

Spectrum S56. ^{1}H NMR (500 MHz, Acetone-d_6, 298 K) spectrum of the concentrated methanol washings from post-sonication polymer $\mathbf{1}_{\mathbf{N}\text{-}\boldsymbol{trans}}$.

8.3.5 Post-Sonication ^{1}H NMR Spectra of Polymer $\mathbf{1}_{\mathbf{C}\text{-}\boldsymbol{cis}}$ (Run 1)

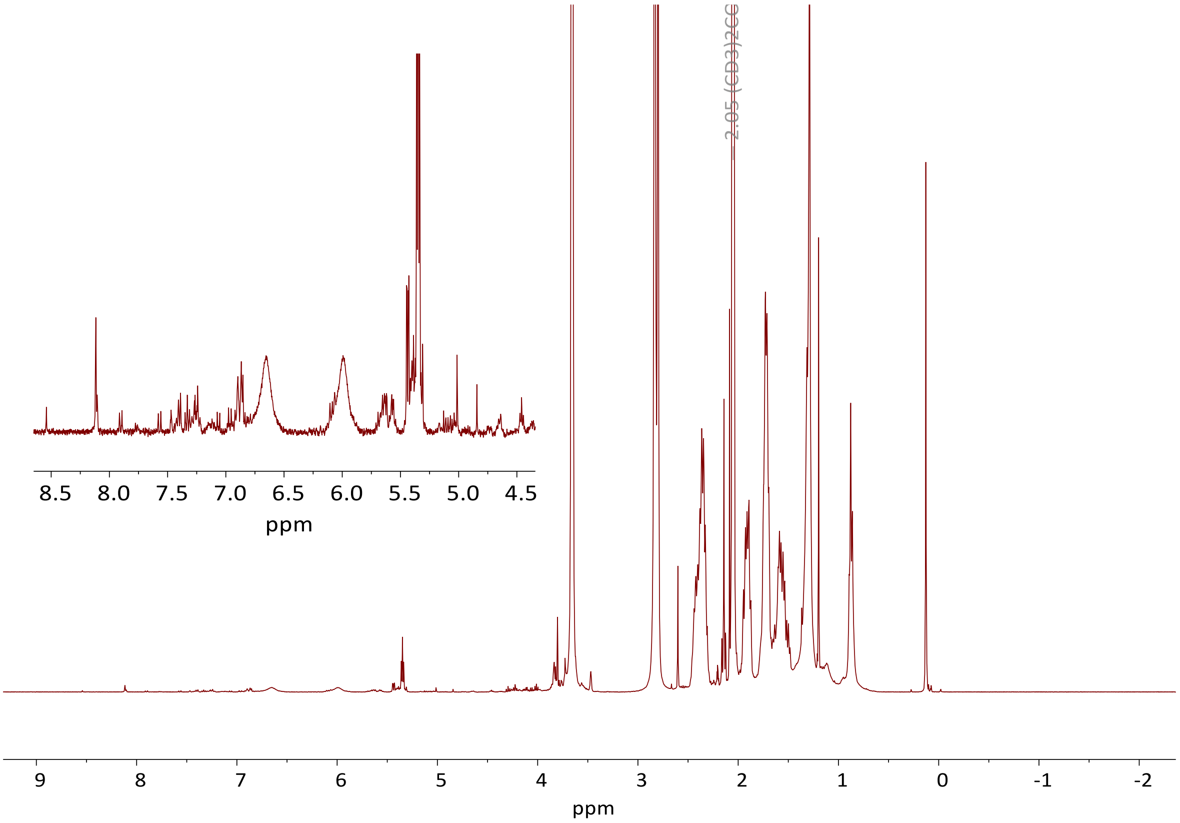

Spectrum S57. ^{1}H NMR (400 MHz, Acetone-d_6, 298 K) spectrum of post-sonication polymer $\mathbf{1}_{\mathbf{C}\text{-}\boldsymbol{cis}}$ before being washed with methanol.

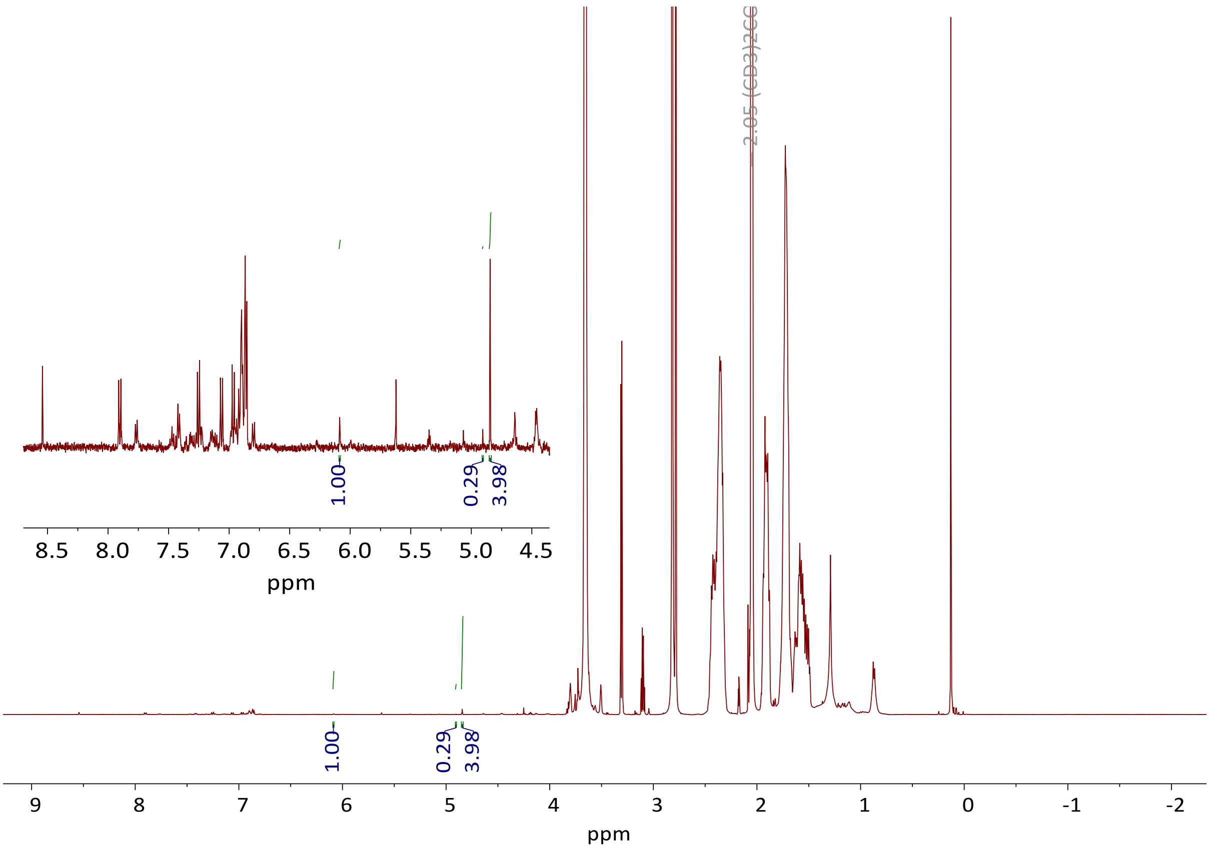

Spectrum S58. ^{1}H NMR (500 MHz, Acetone-d_6, 298 K) spectrum of post-sonication polymer **1**$_{\mathbf{C}\text{-}\boldsymbol{cis}}$ after being washed with methanol.

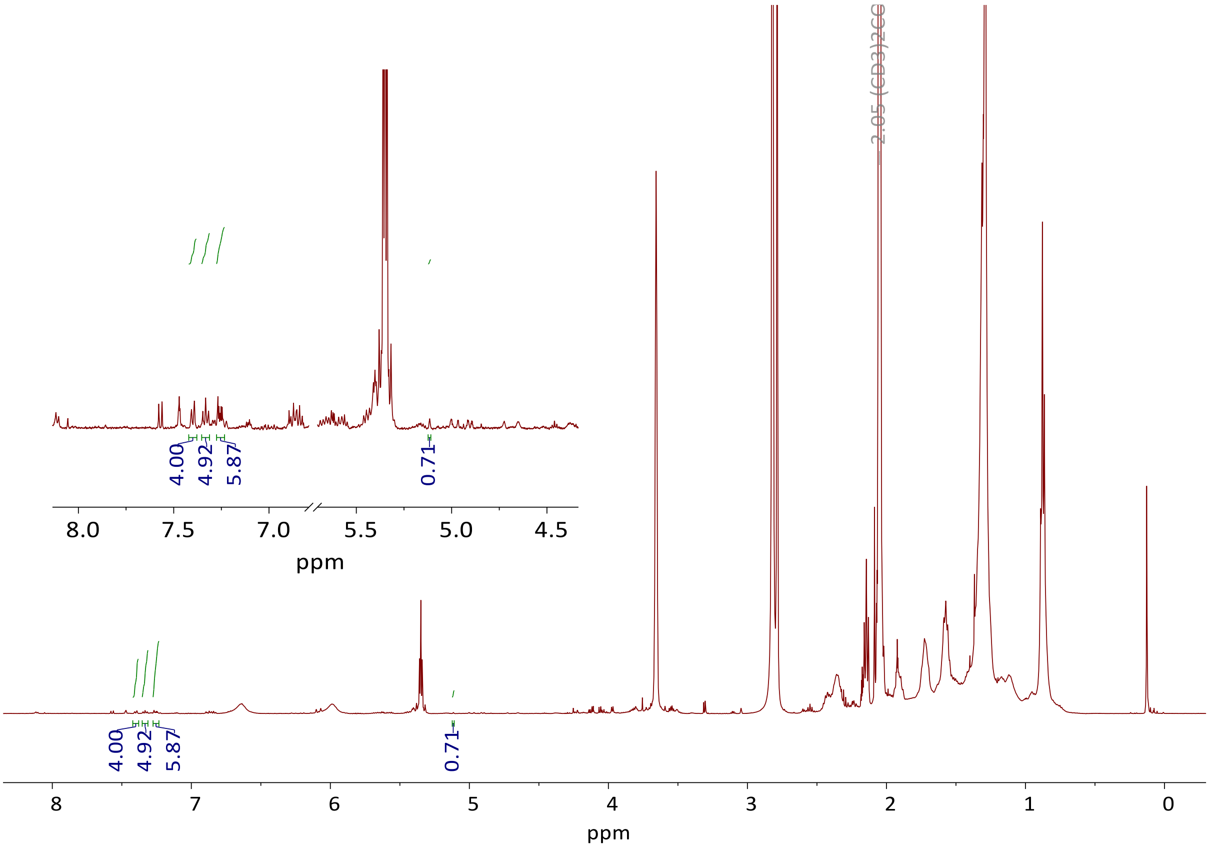

Spectrum S59. ^{1}H NMR (500 MHz, Acetone-d_6, 298 K) spectrum of the concentrated methanol washings from post-sonication polymer **1**$_{\mathbf{C}\text{-}\boldsymbol{cis}}$.

8.3.6 Post-Sonication ^{1}H NMR Spectra of Polymer $1_{C\text{-}cis}$ (Run 2)

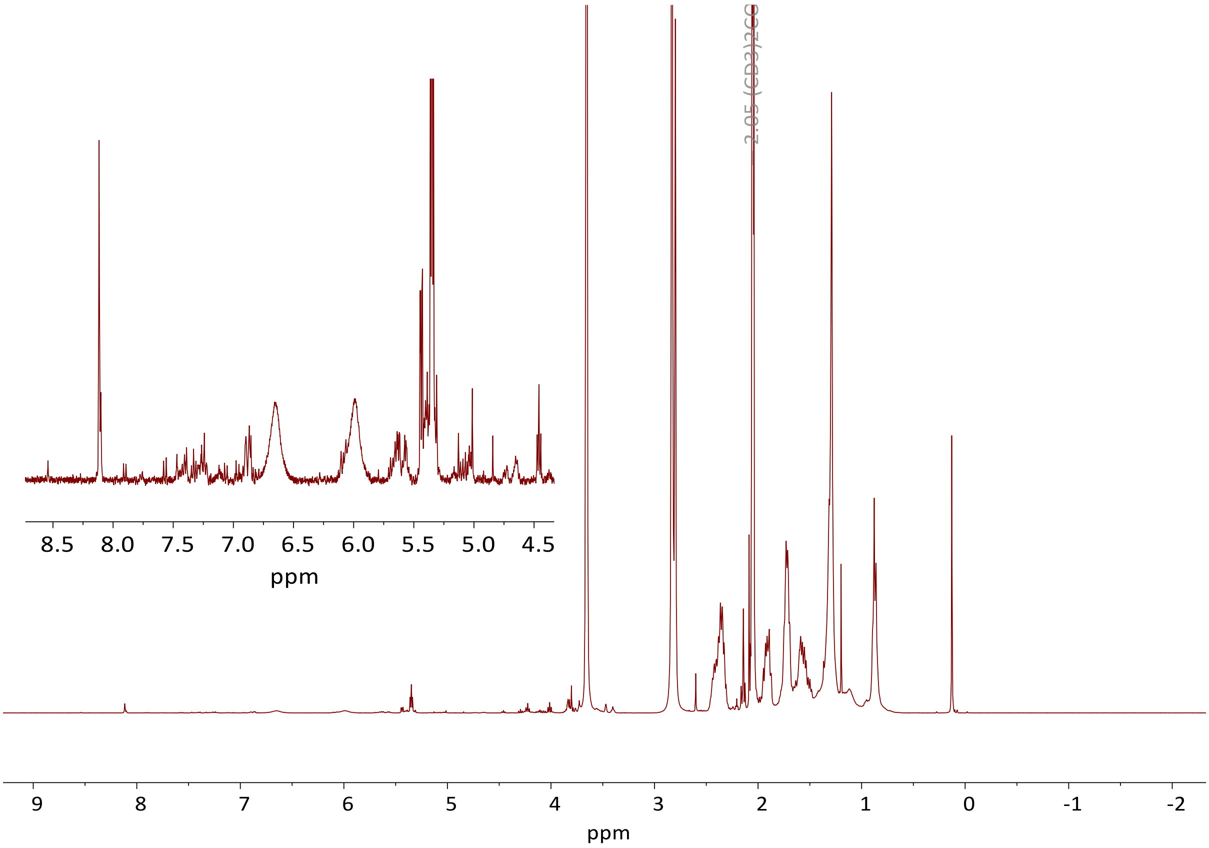

Spectrum S60. ^{1}H NMR (400 MHz, Acetone-d_6, 298 K) spectrum of post-sonication polymer $\mathbf{1}_{C\text{-}cis}$ before being washed with methanol.

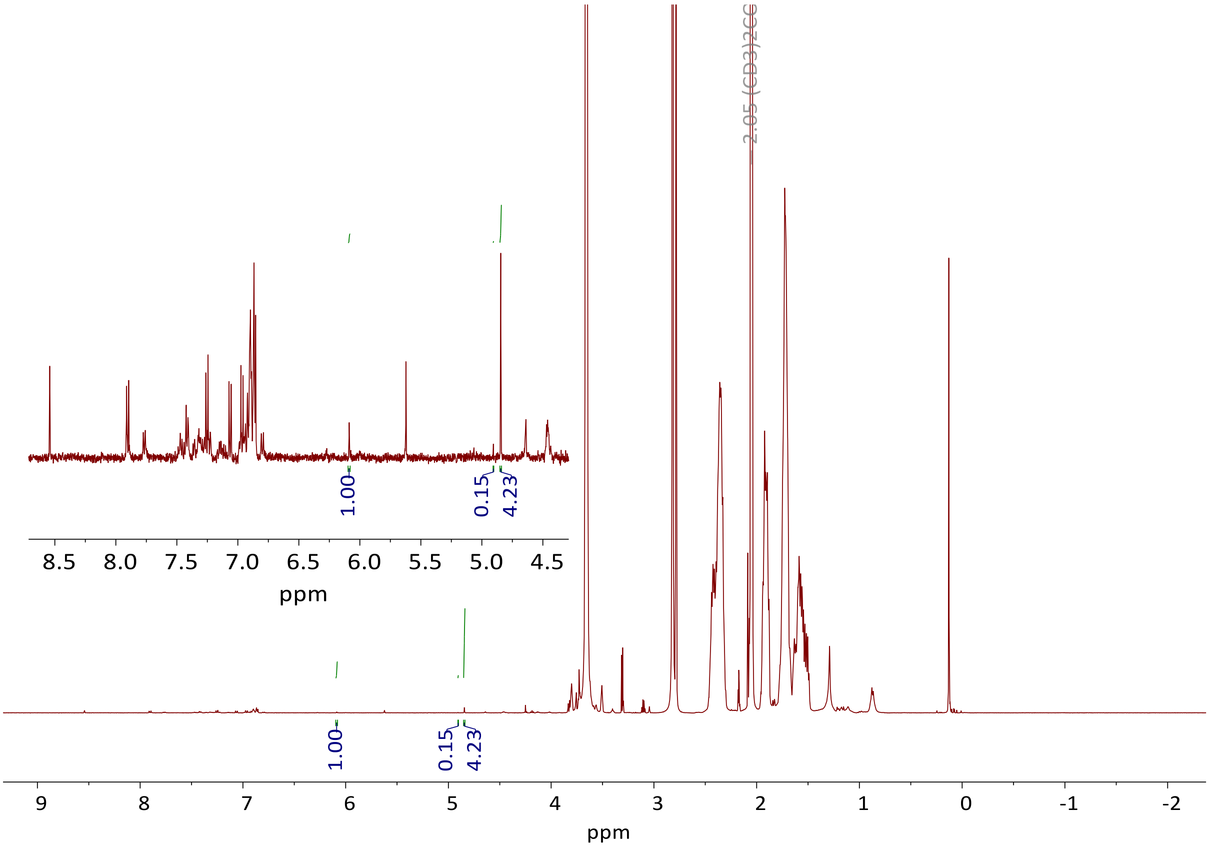

Spectrum S61. ^{1}H NMR (500 MHz, Acetone-d_6, 298 K) spectrum of post-sonication polymer $\mathbf{1}_{C\text{-}cis}$ after being washed with methanol.

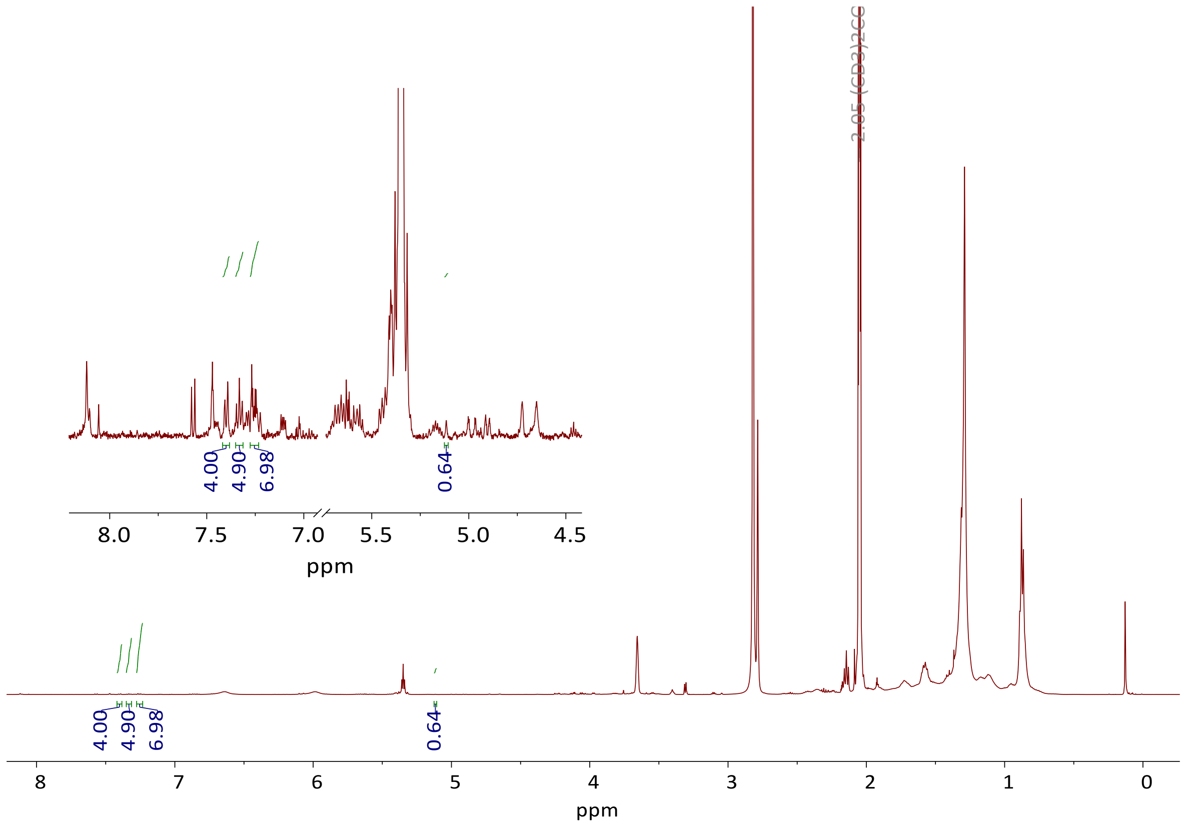

Spectrum S62. ^{1}H NMR (500 MHz, Acetone-d_6, 298 K) spectrum of the concentrated methanol washings from post-sonication polymer $\mathbf{1}_{\mathbf{C}\text{-}\boldsymbol{cis}}$.

8.3.7 Post-Sonication ^{1}H NMR Spectra of Polymer $\mathbf{1}_{\mathbf{C}\text{-}\boldsymbol{trans}}$ (Run 1)

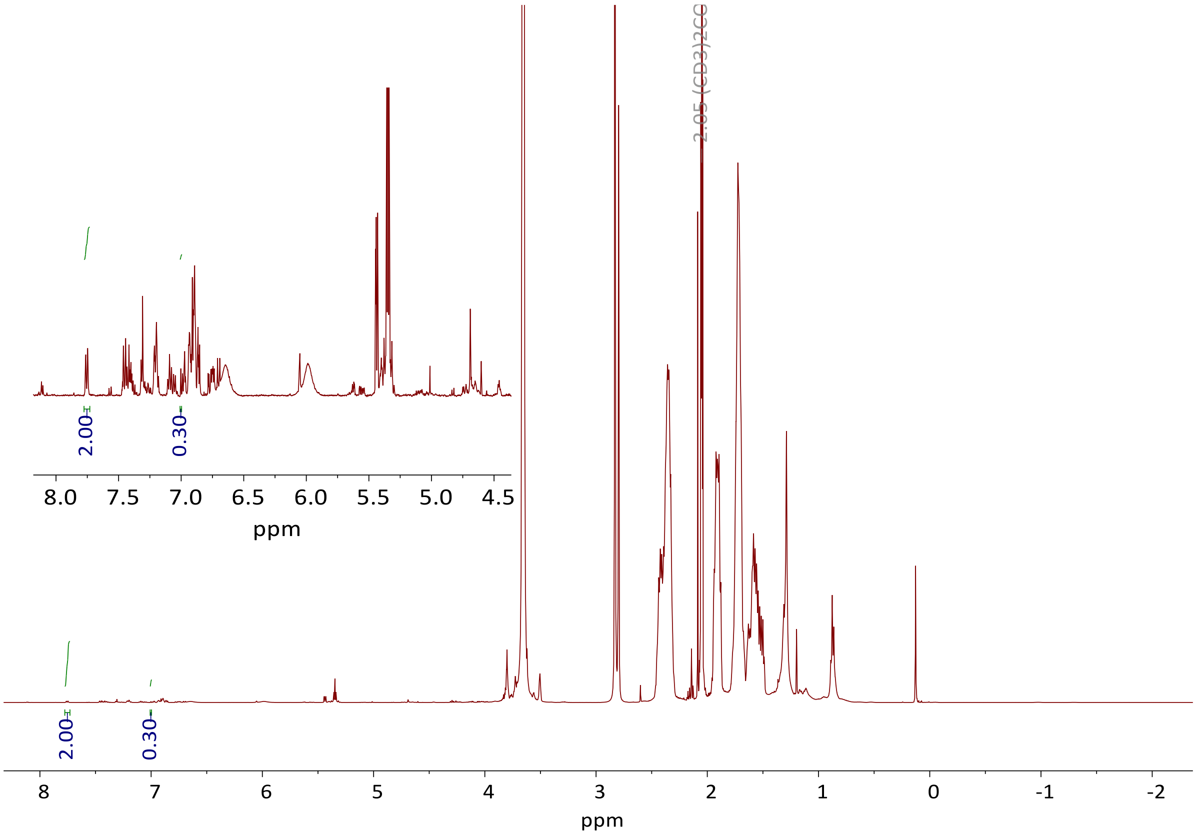

Spectrum S63. ^{1}H NMR (500 MHz, Acetone-d_6, 298 K) spectrum of post-sonication polymer $\mathbf{1}_{\mathbf{C}\text{-}\boldsymbol{trans}}$ before being washed with methanol.

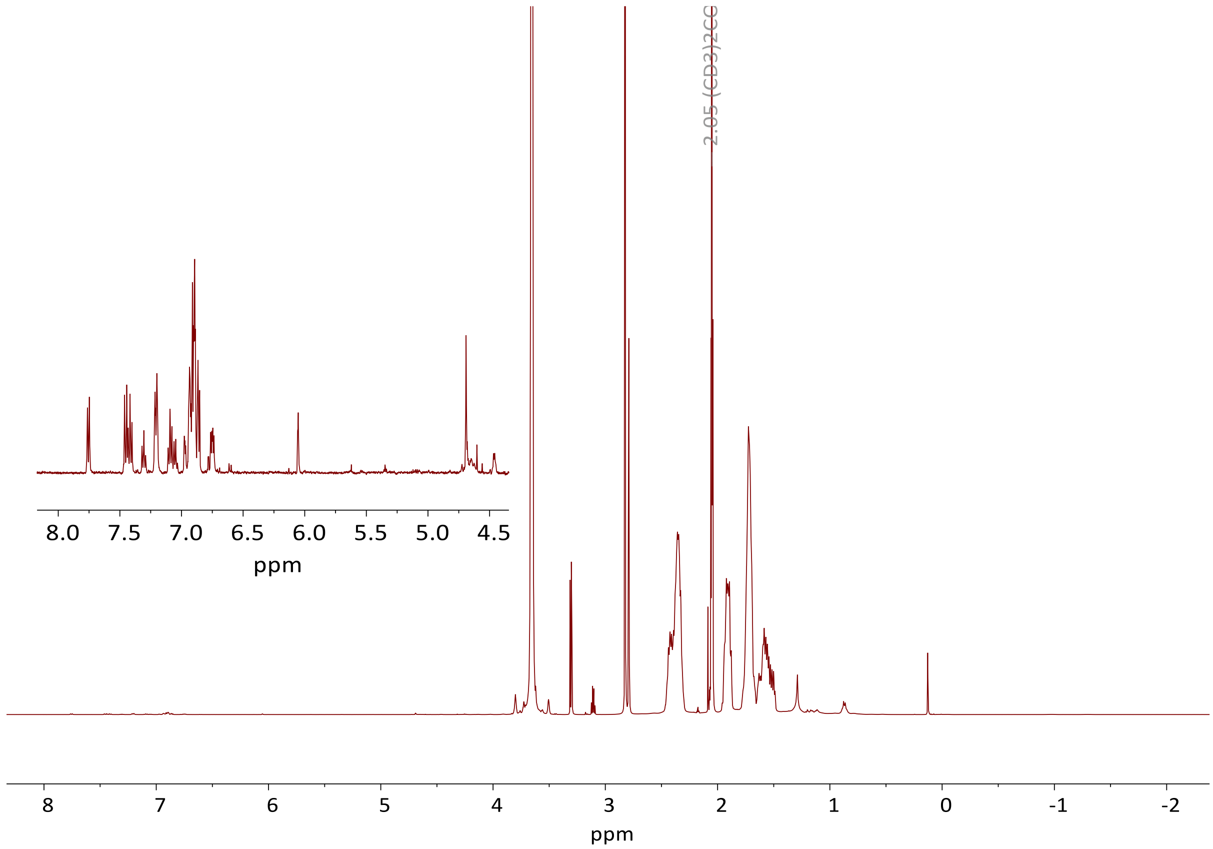

Spectrum S64. ^{1}H NMR (500 MHz, Acetone-d_6, 298 K) spectrum of post-sonication polymer $\mathbf{1}_{\mathbf{C}\text{-}\boldsymbol{trans}}$ after being washed with methanol.

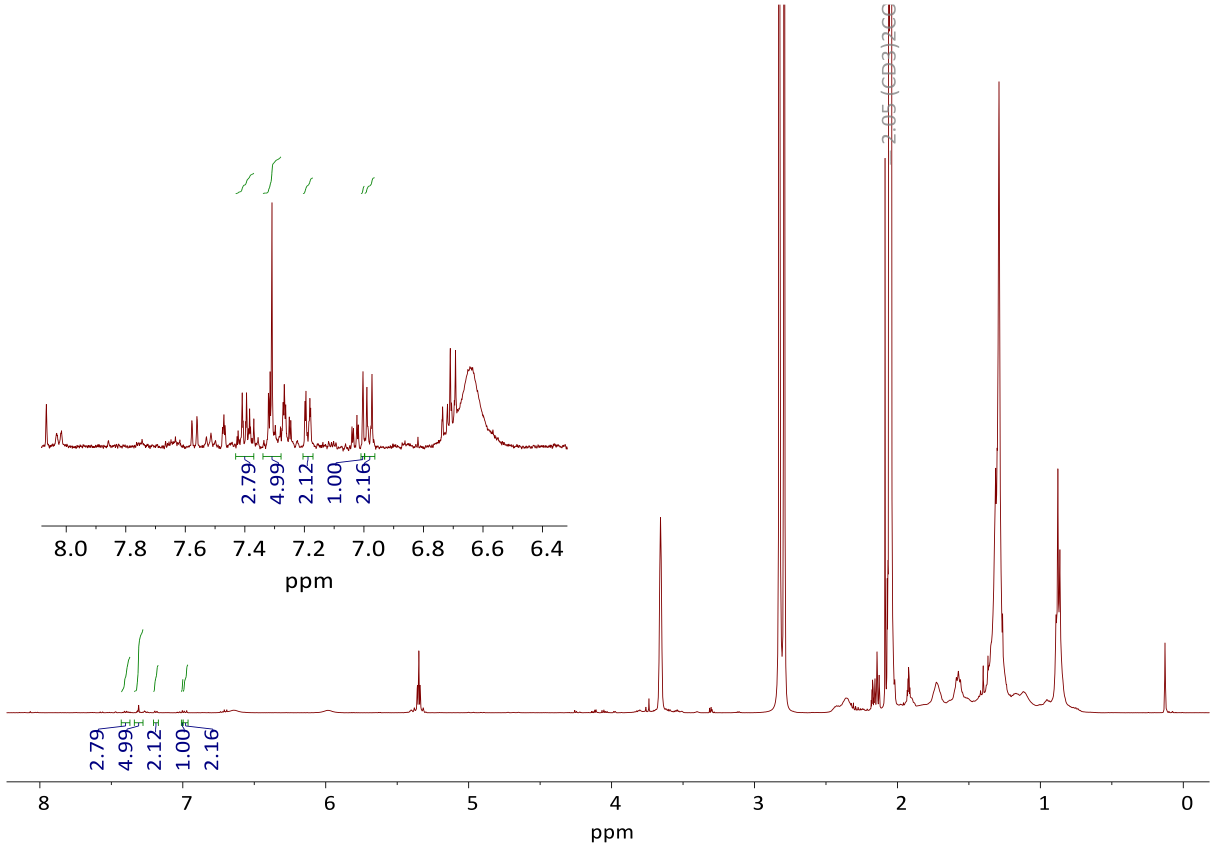

Spectrum S65. ^{1}H NMR (500 MHz, Acetone-d_6, 298 K) spectrum of the concentrated methanol washings from post-sonication polymer $\mathbf{1}_{\mathbf{C}\text{-}\boldsymbol{trans}}$.

8.3.8 Post-Sonication ^{1}H NMR Spectra of Polymer $\mathbf{1}_{C\text{-}trans}$ (Run 2)

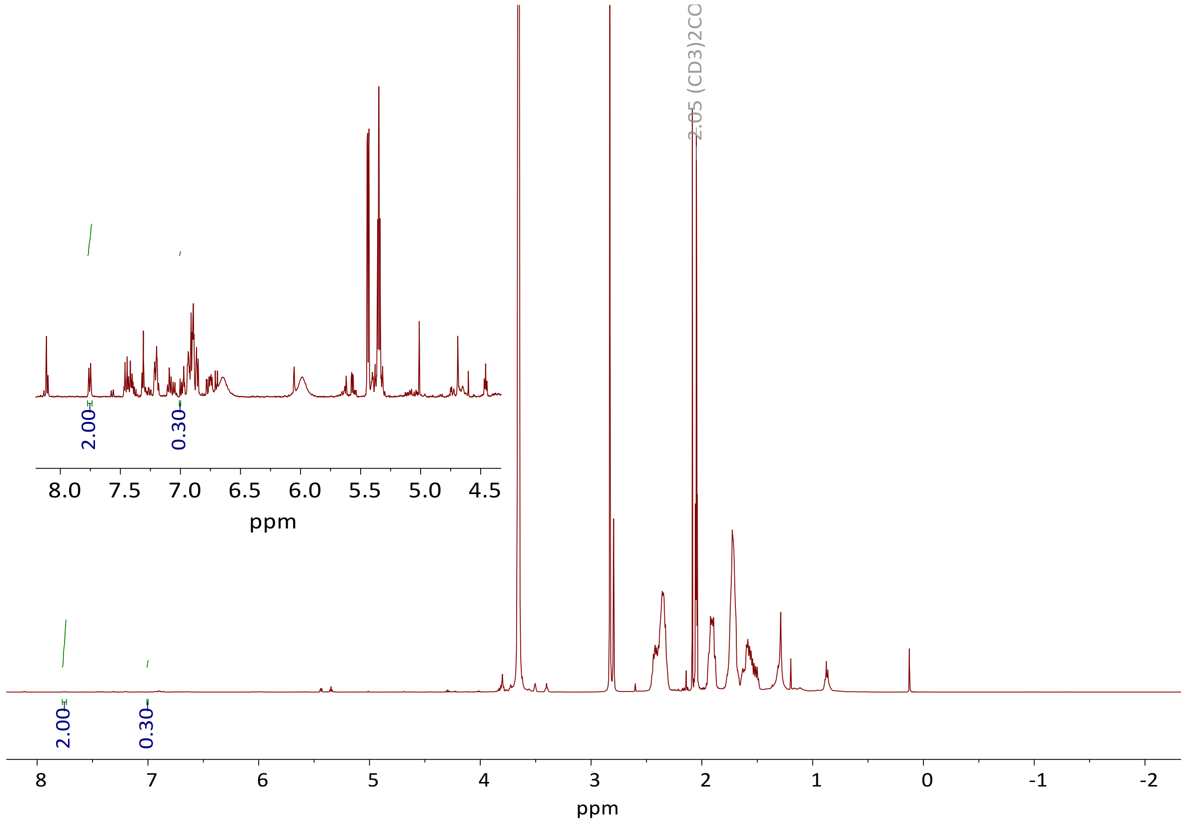

Spectrum S66. ^{1}H NMR (500 MHz, Acetone-d_6, 298 K) spectrum of post-sonication polymer $\mathbf{1}_{C\text{-}trans}$ before being washed with methanol.

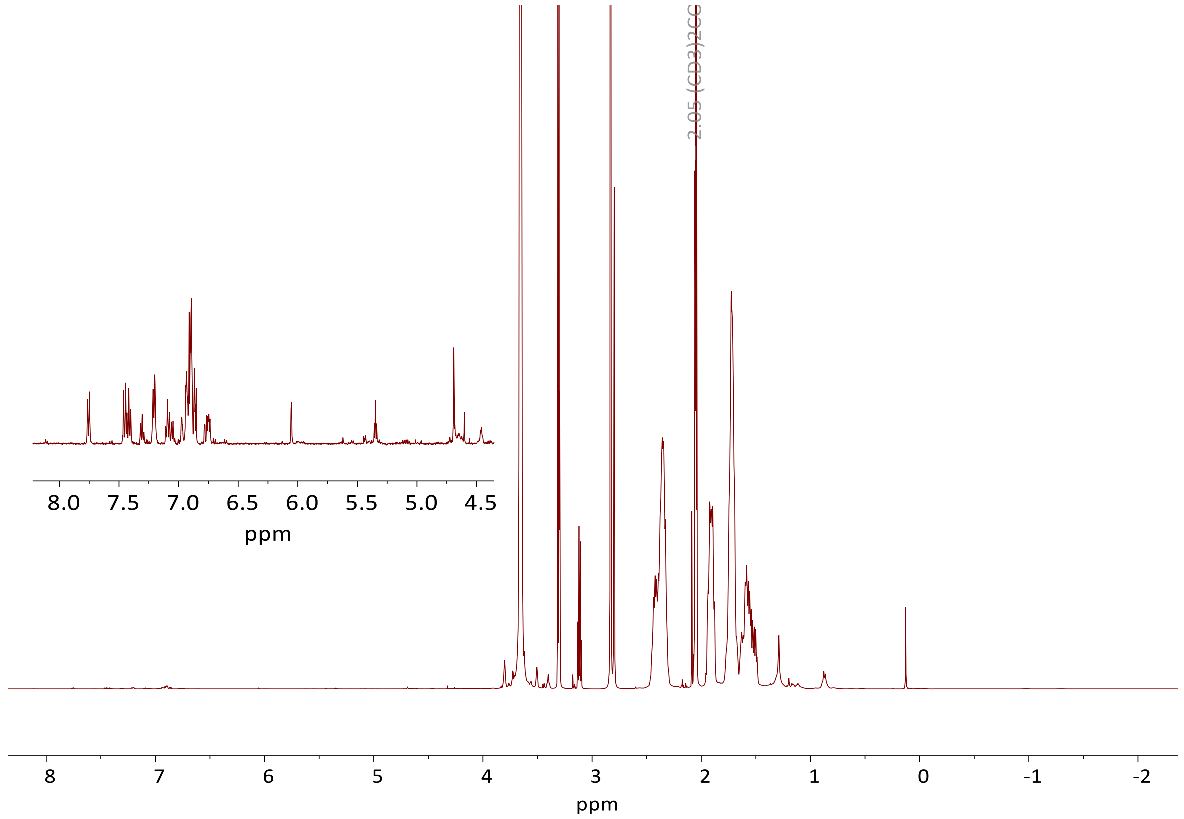

Spectrum S67. ^{1}H NMR (500 MHz, Acetone-d_6, 298 K) spectrum of post-sonication polymer $\mathbf{1}_{C\text{-}trans}$ after being washed with methanol.

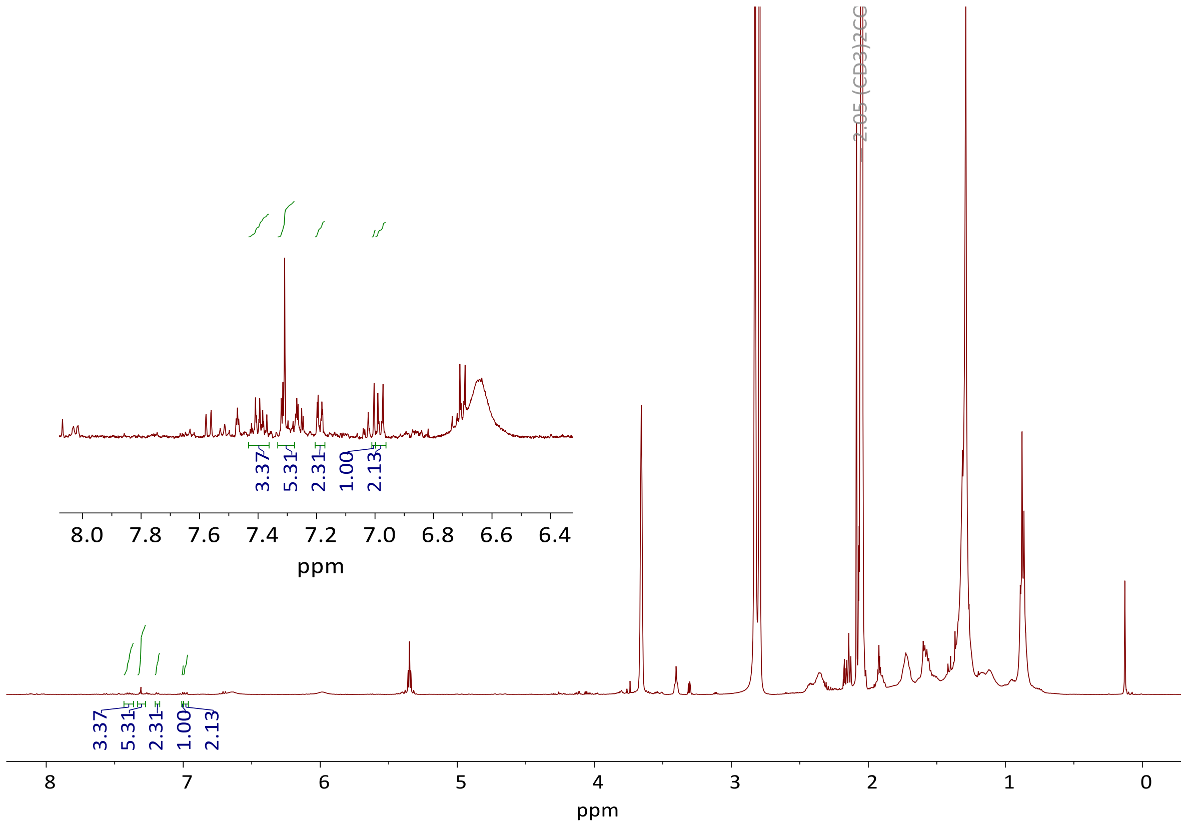

Spectrum S68. ^{1}H NMR (500 MHz, Acetone-d_6, 298 K) spectrum of the concentrated methanol washings from post-sonication polymer $\mathbf{1}_{C\text{-}trans}$.

8.3.9 Post-Sonication ^{1}H NMR Spectra of Polymer S18

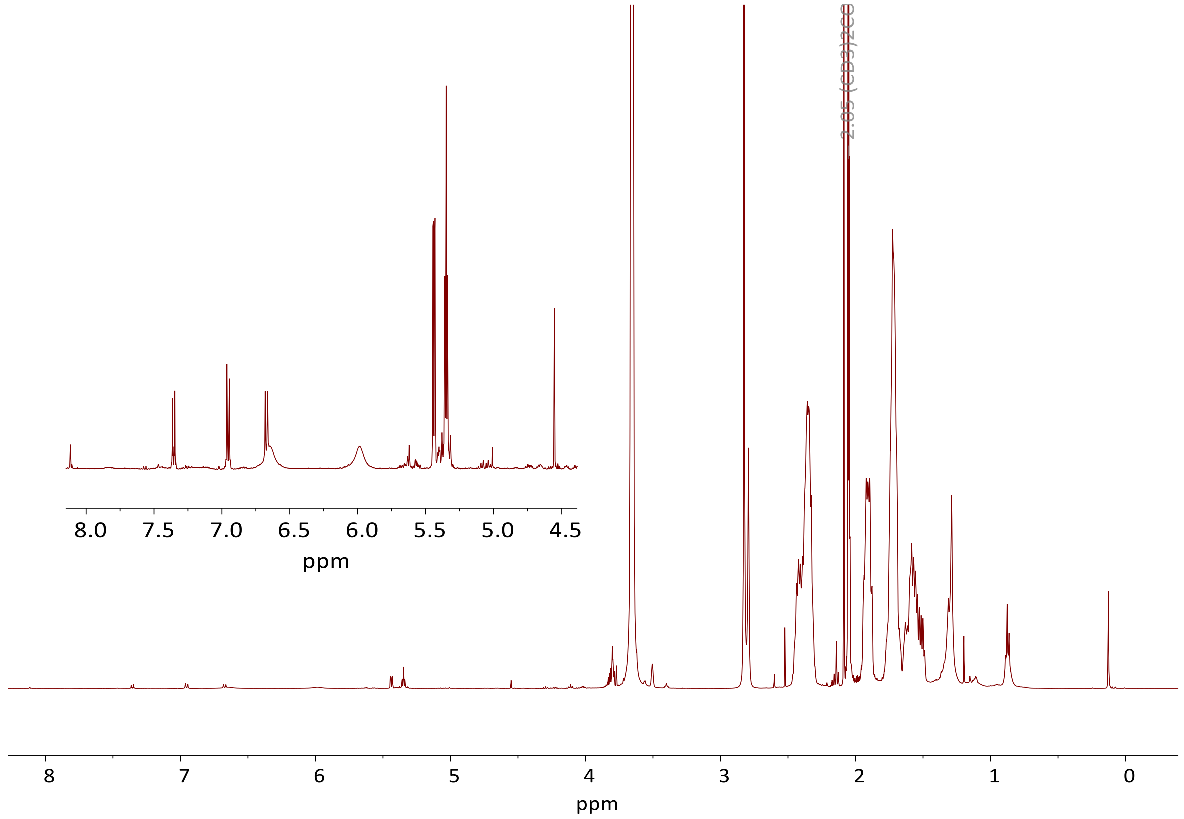

Spectrum S69. ^{1}H NMR (500 MHz, Acetone-d_6, 298 K) spectrum of post-sonication polymer **S18** before being washed with methanol.

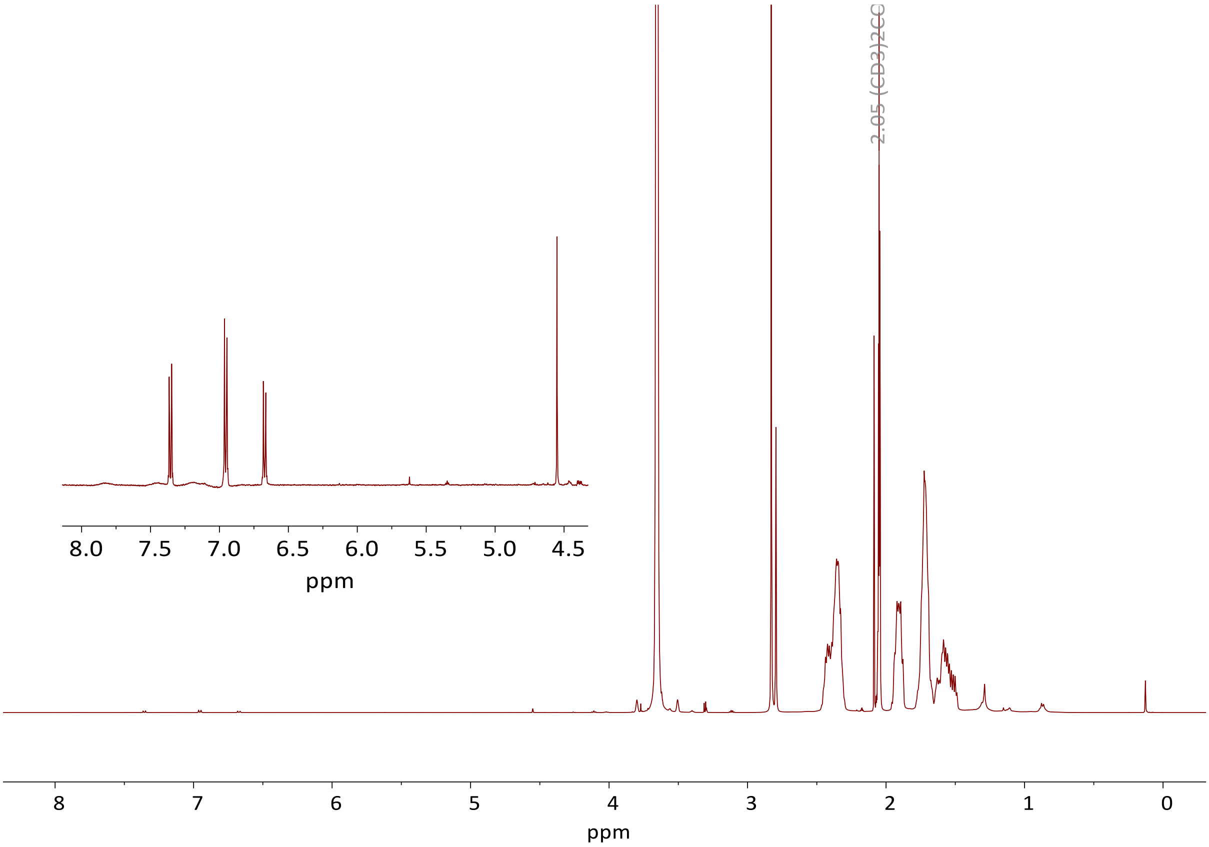

Spectrum S70. ^{1}H NMR (500 MHz, Acetone-d_6, 298 K) spectrum of post-sonication polymer **S18** after being washed with methanol.

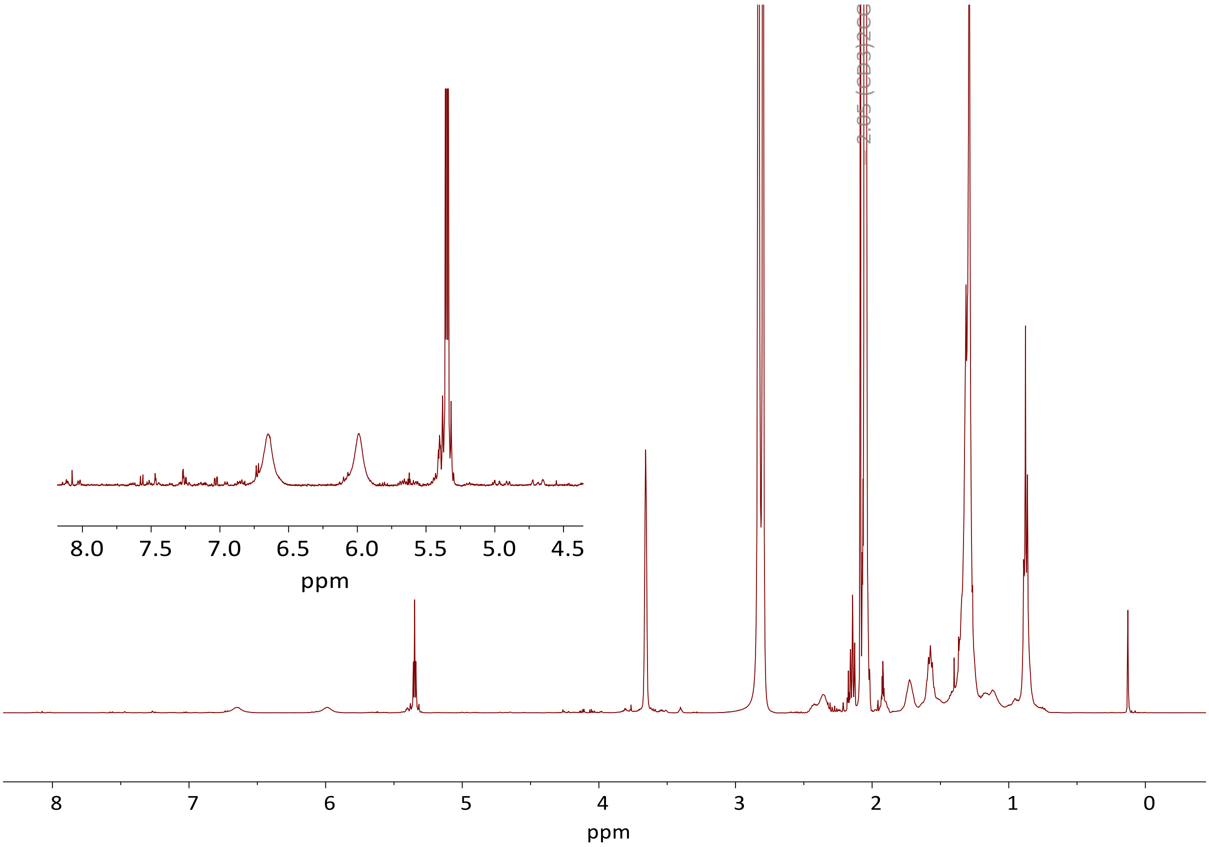

Spectrum S71. ^{1}H NMR (500 MHz, Acetone-d_6, 298 K) spectrum of the concentrated methanol washings from post-sonication polymer **S18**.

8.3.10 Post-Sonication ^1H NMR Spectra of Polymer S19

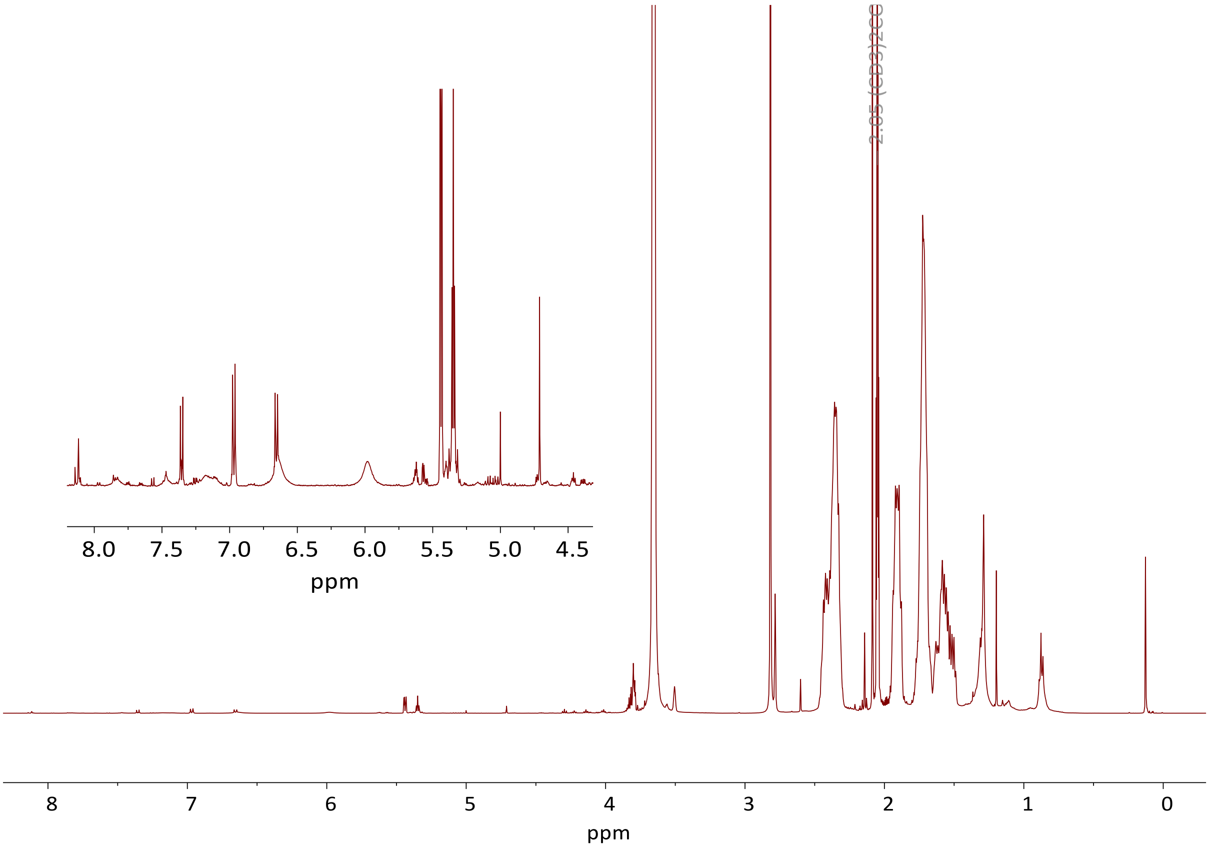

Spectrum S72. ^1H NMR (500 MHz, Acetone-d_6, 298 K) spectrum of post-sonication polymer **S19** before being washed with methanol.

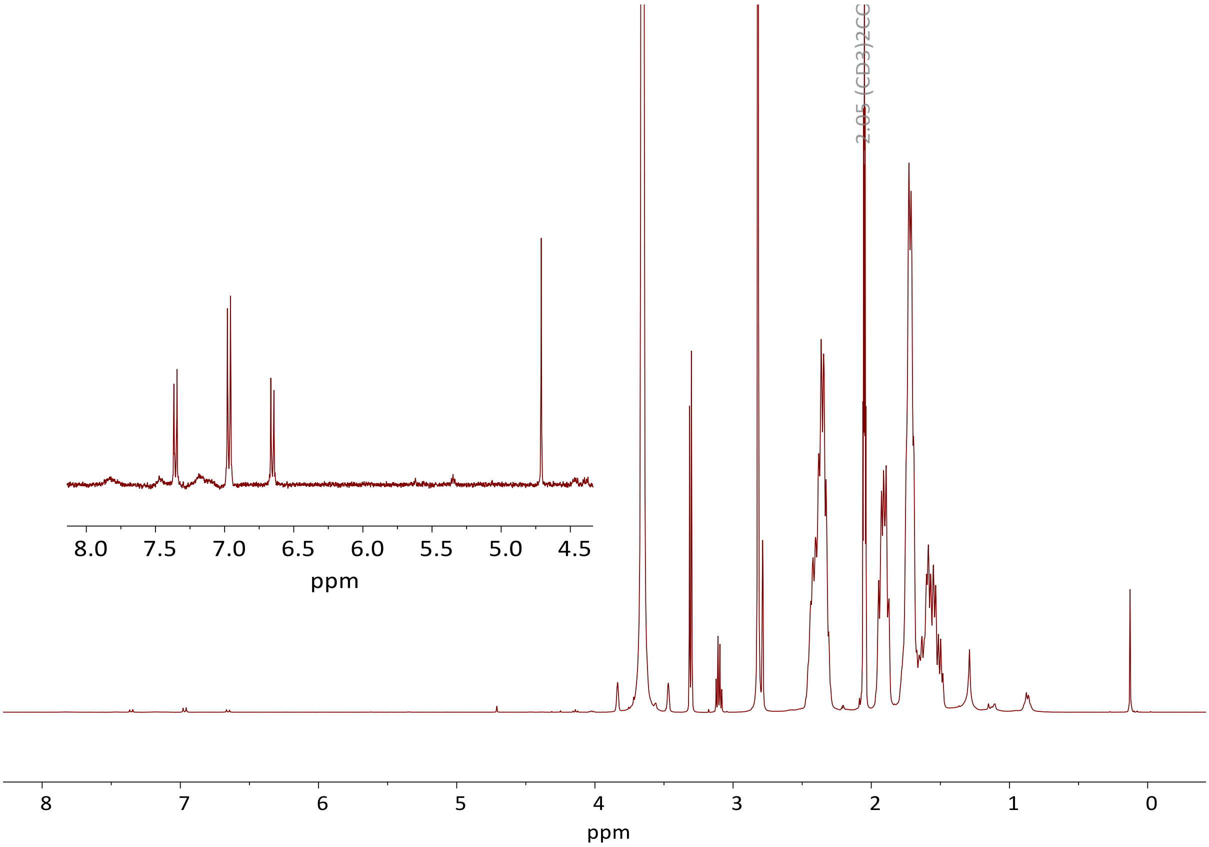

Spectrum S73. ^1H NMR (400 MHz, Acetone-d_6, 298 K) spectrum of post-sonication polymer **S19** after being washed with methanol.

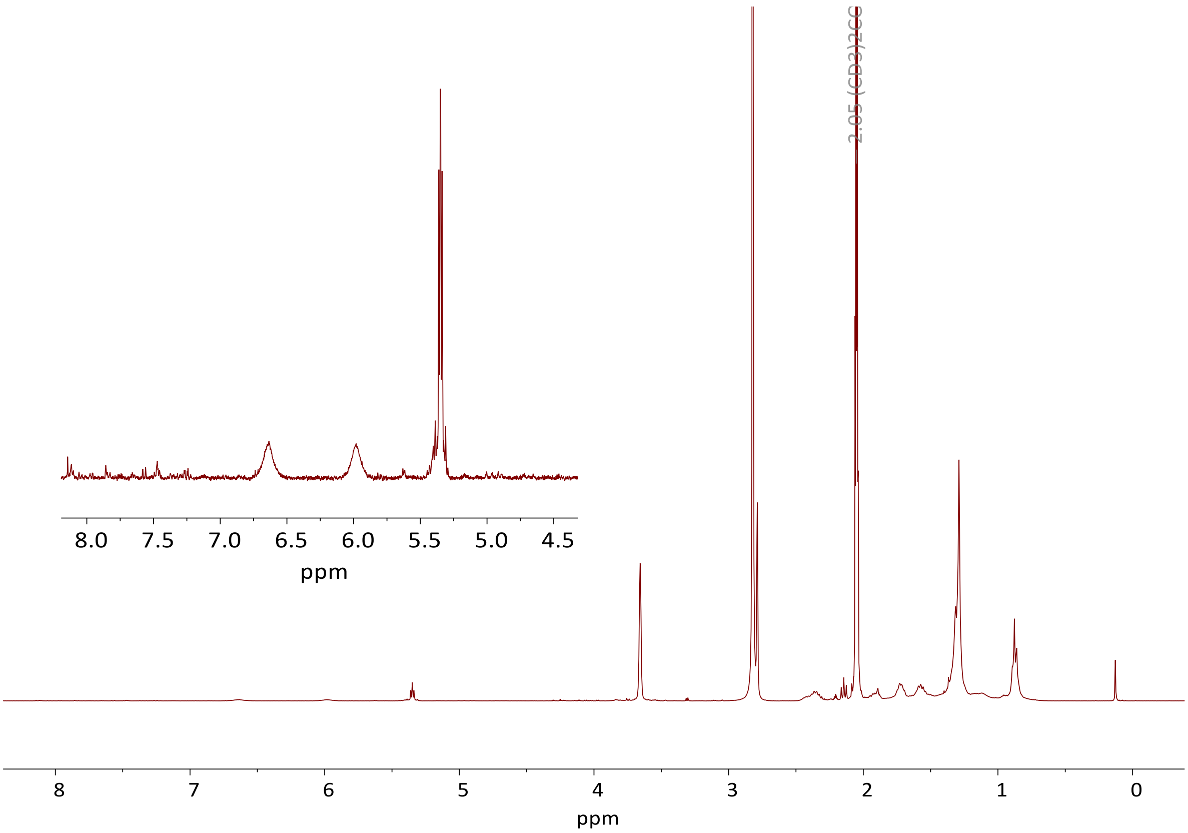

Spectrum S74. ^{1}H NMR (400 MHz, Acetone-d_6, 298 K) spectrum of the concentrated methanol washings from post-sonication polymer **S19**.

8.3.11 Post-Sonication ^{1}H NMR Spectra of Polymer S20

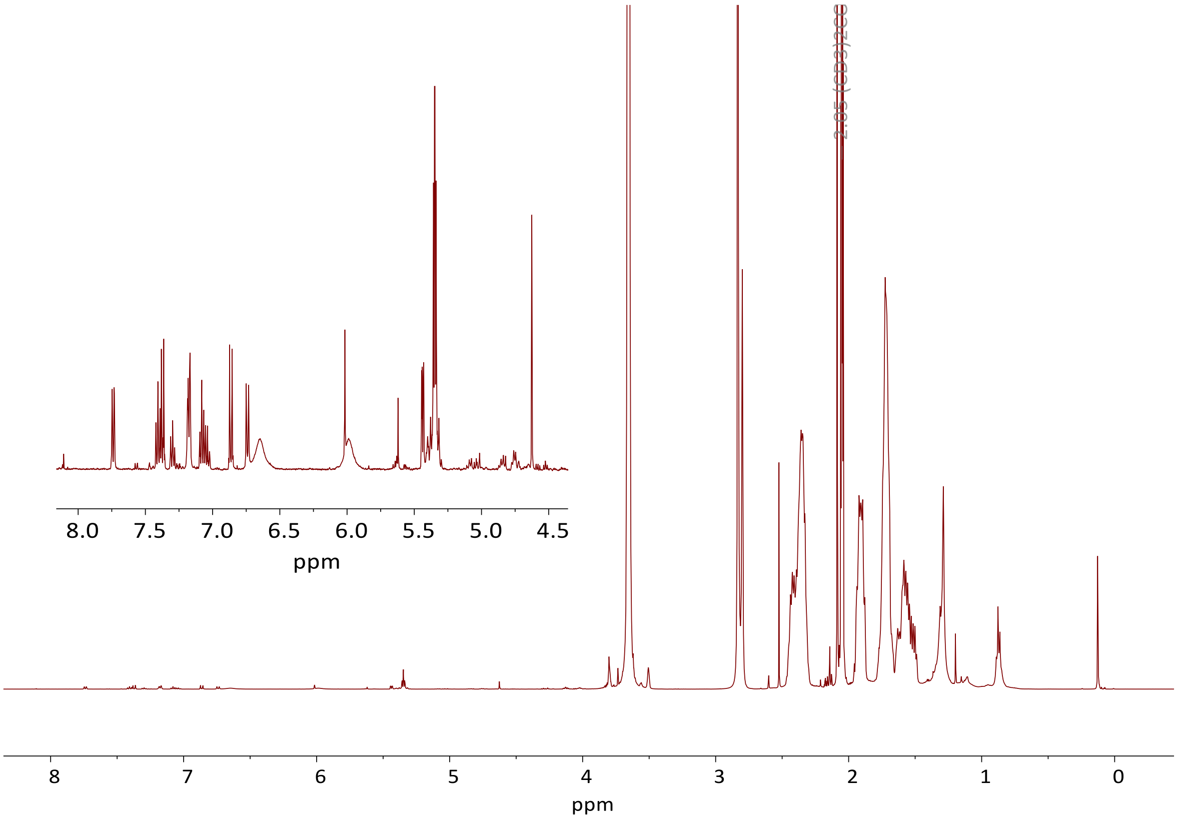

Spectrum S75. ^{1}H NMR (500 MHz, Acetone-d_6, 298 K) spectrum of post-sonication polymer **S20** before being washed with methanol.

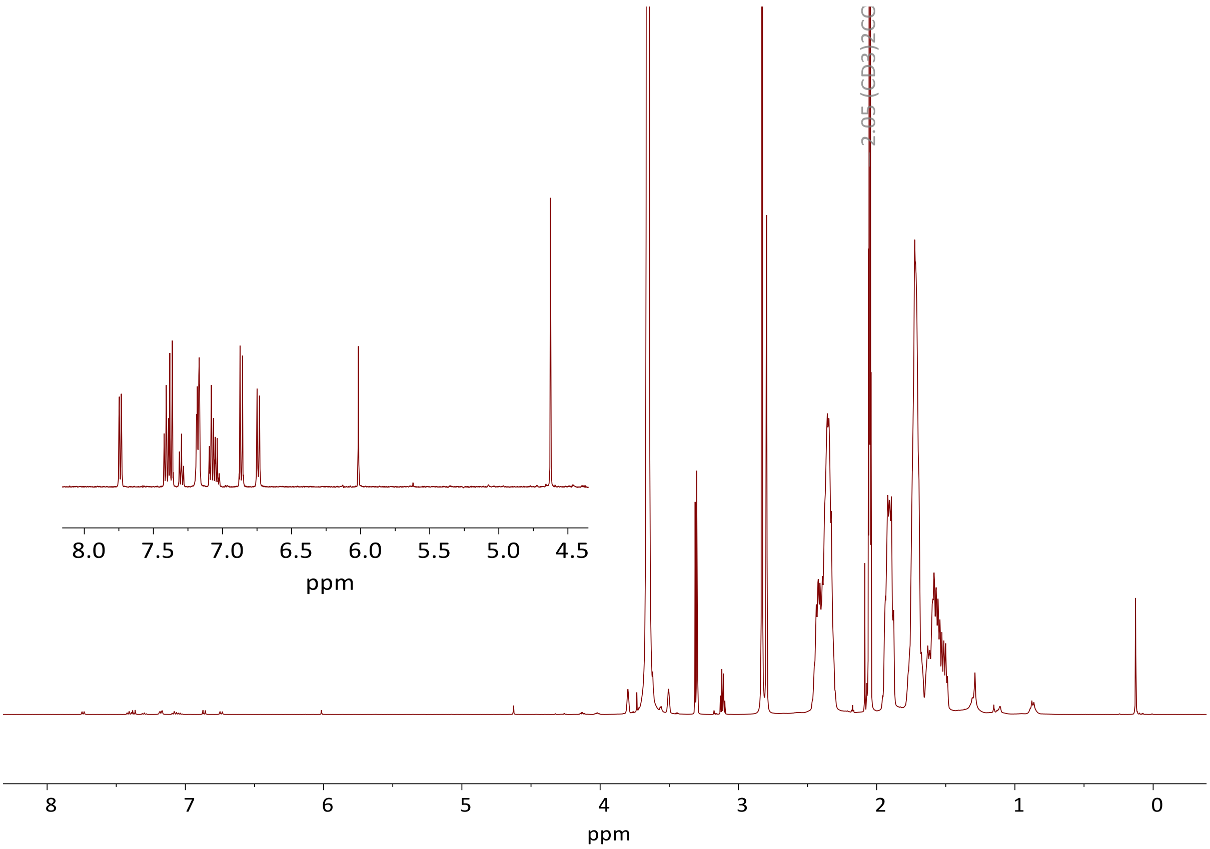

Spectrum S76. ^{1}H NMR (500 MHz, Acetone-d_6, 298 K) spectrum of post-sonication polymer **S20** after being washed with methanol.

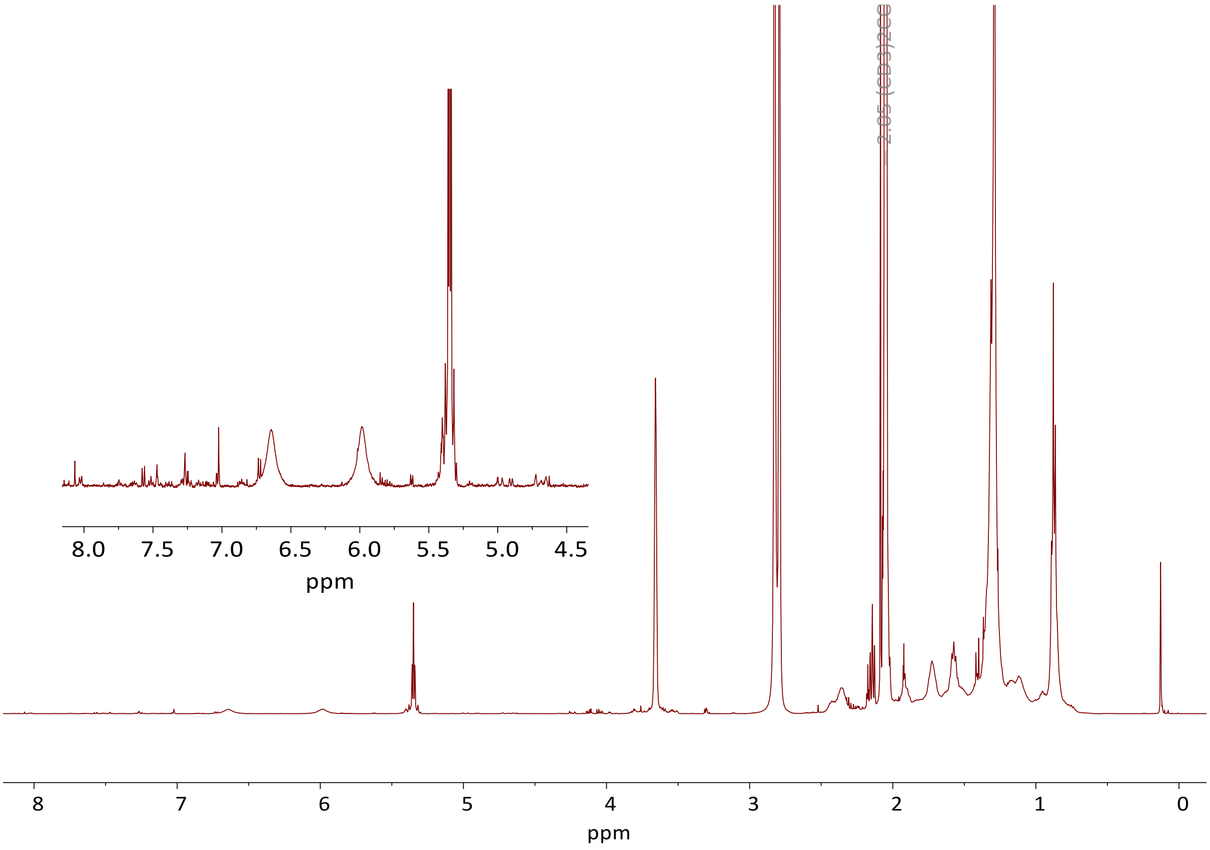

Spectrum S77. ^{1}H NMR (500 MHz, Acetone-d_6, 298 K) spectrum of the concentrated methanol washings from post-sonication polymer **S20**.

8.3.12 Post-Sonication ^{1}H NMR Spectra of Polymer S21

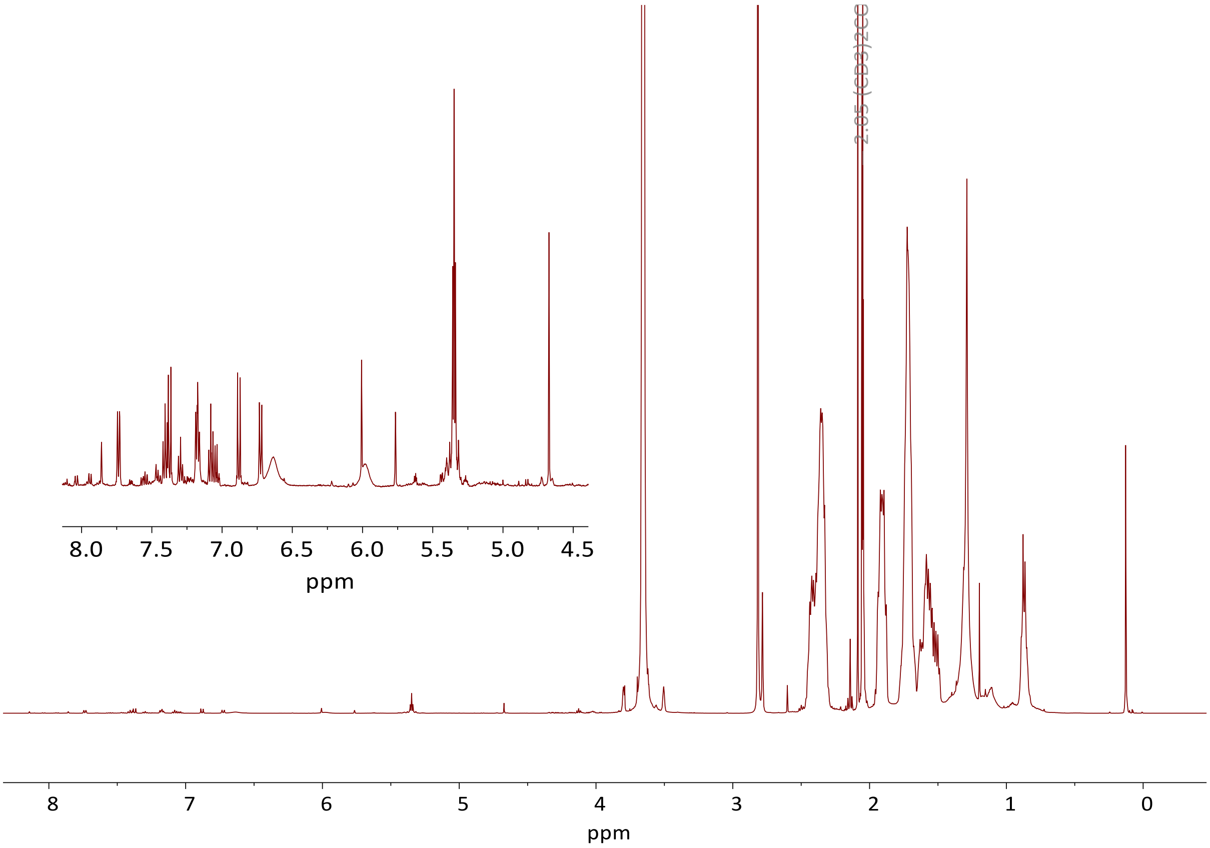

Spectrum S78. ^{1}H NMR (500 MHz, Acetone-d_6, 298 K) spectrum of post-sonication polymer **S21** before being washed with methanol.

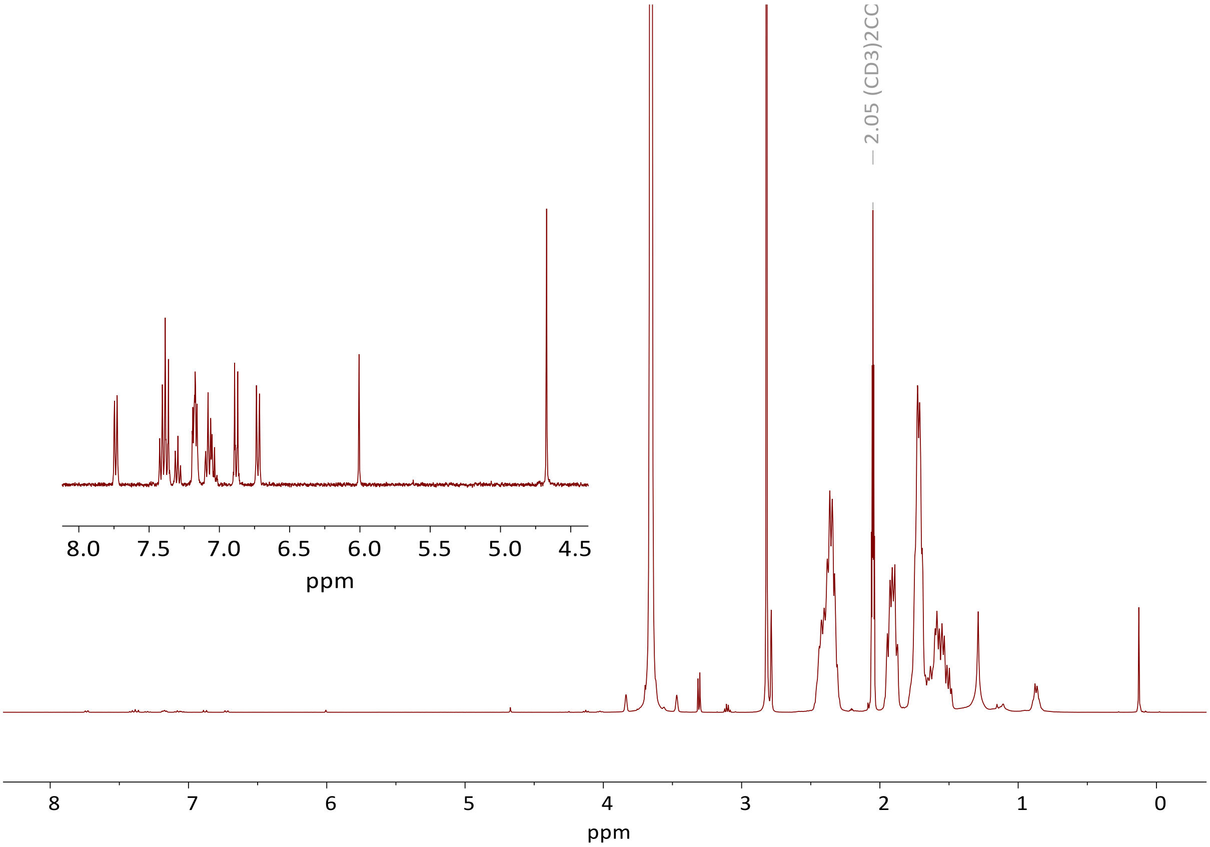

Spectrum S79. ^{1}H NMR (400 MHz, Acetone-d_6, 298 K) spectrum of post-sonication polymer **S21** after being washed with methanol.

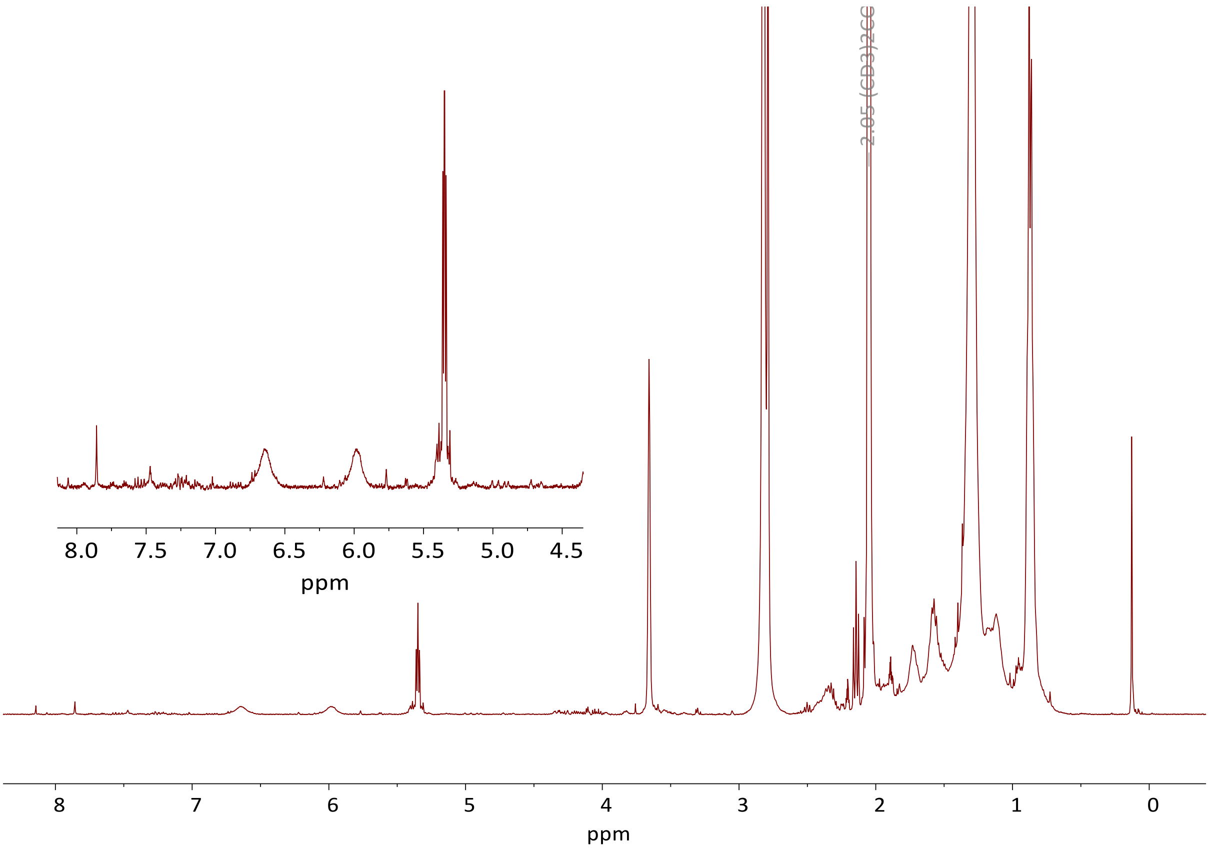

Spectrum S80. ^{1}H NMR (400 MHz, Acetone-d_6, 298 K) spectrum of the concentrated methanol washings from post-sonication polymer **S21**.

9 Mass Spectrometry Isotopic Patterns

9.1 Isotopic distribution of $\mathbf{11}_{N\text{-}cis}$

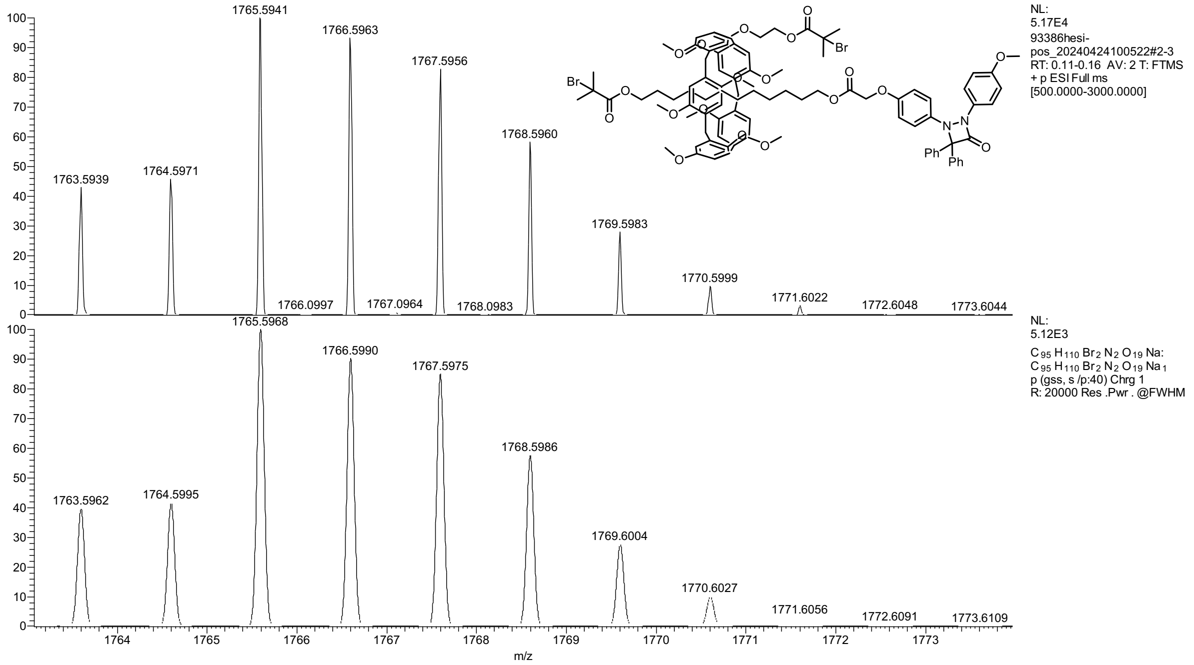

Spectrum S81. Isotopic distribution of $\mathbf{11}_{N\text{-}cis}$. Top: Measured isotopic distribution for $C_{95}H_{110}Br_2N_2O_{19}Na$ ($[M+Na]^+$, +ESI). Bottom: Simulated isotopic distribution for $C_{95}H_{110}Br_2N_2O_{19}Na^+$.

9.2 Isotopic distribution of $\mathbf{11}_{N\text{-}trans}$

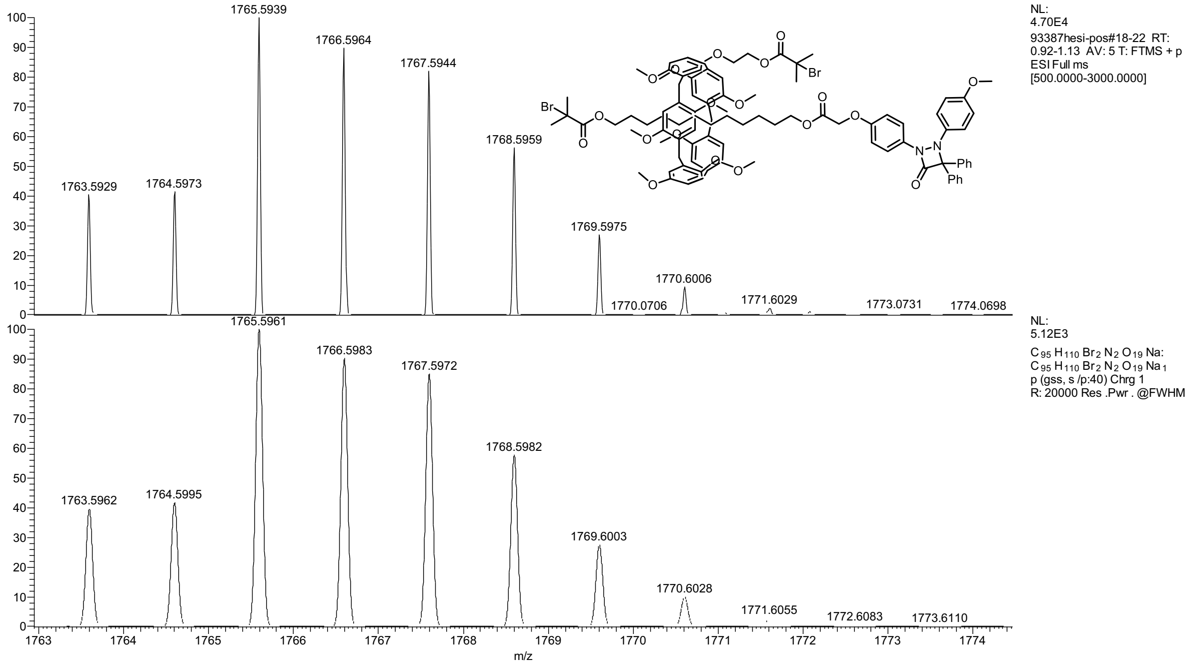

Spectrum S82. Isotopic distribution of $\mathbf{11}_{N\text{-}trans}$. Top: Measured isotopic distribution for $C_{95}H_{110}Br_2N_2O_{19}Na$ ($[M+Na]^+$, +ESI). Bottom: Simulated isotopic distribution for $C_{95}H_{110}Br_2N_2O_{19}Na^+$.

9.3 Isotopic distribution of $11_{C\text{-}cis}$

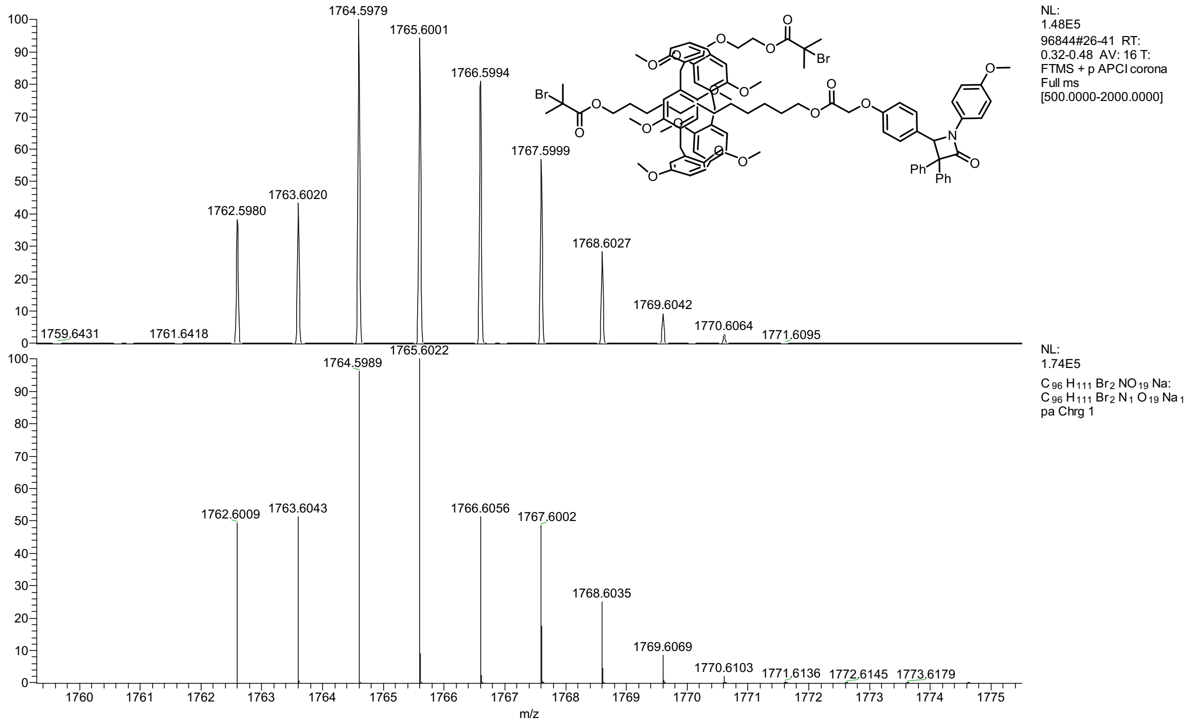

Spectrum S83. Isotopic distribution of $\mathbf{11}_{C\text{-}cis}$. Top: Measured isotopic distribution for $C_{96}H_{111}Br_2NO_{19}Na$ ($[M+Na]^+$, +APCI). Bottom: Simulated isotopic distribution for $C_{96}H_{111}Br_2NO_{19}Na^+$.

9.4 Isotopic distribution of $11_{C\text{-}trans}$

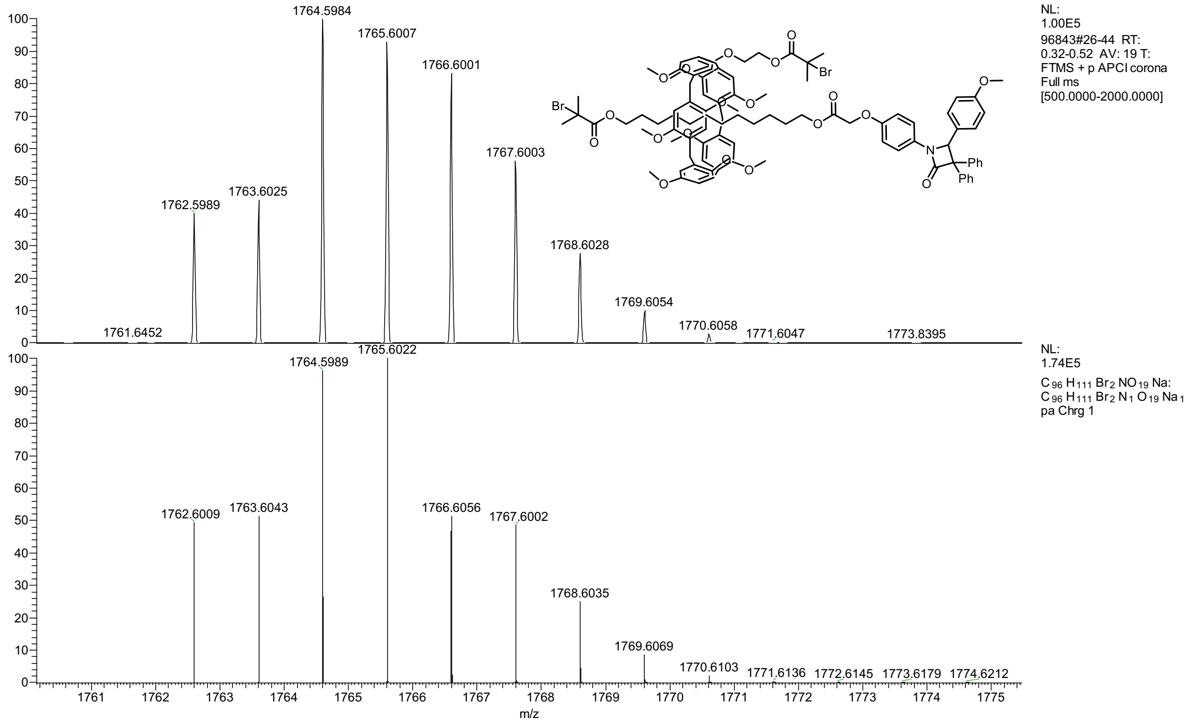

Spectrum S84. Isotopic distribution of $\mathbf{11}_{C\text{-}trans}$. Top: Measured isotopic distribution for $C_{96}H_{111}Br_2NO_{19}Na$ ($[M+Na]^+$, +APCI). Bottom: Simulated isotopic distribution for $C_{96}H_{111}Br_2NO_{19}Na^+$.

9.5 Isotopic distribution of S11

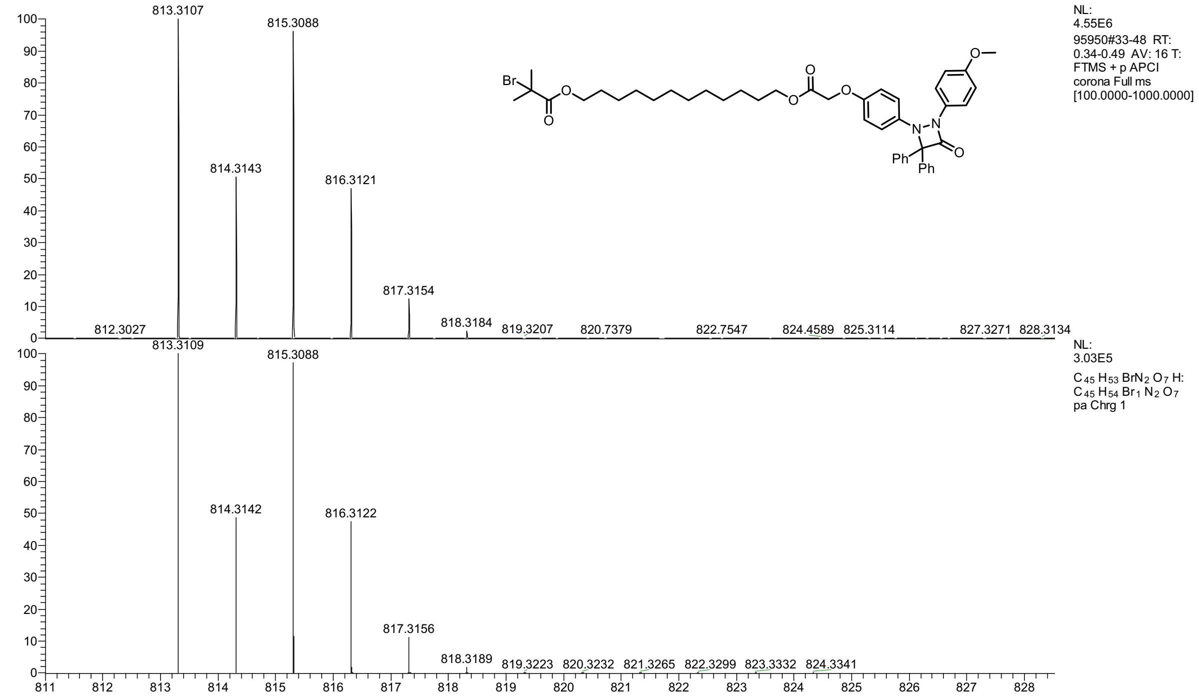

Spectrum S85. Isotopic distribution of **S11**. Top: Measured isotopic distribution for $C_{45}H_{53}BrN_2O_7H$ ($[M+H]^+$, + APCI). Bottom: Simulated isotopic distribution for $C_{45}H_{53}BrN_2O_7H^+$.

9.6 Isotopic distribution of S12

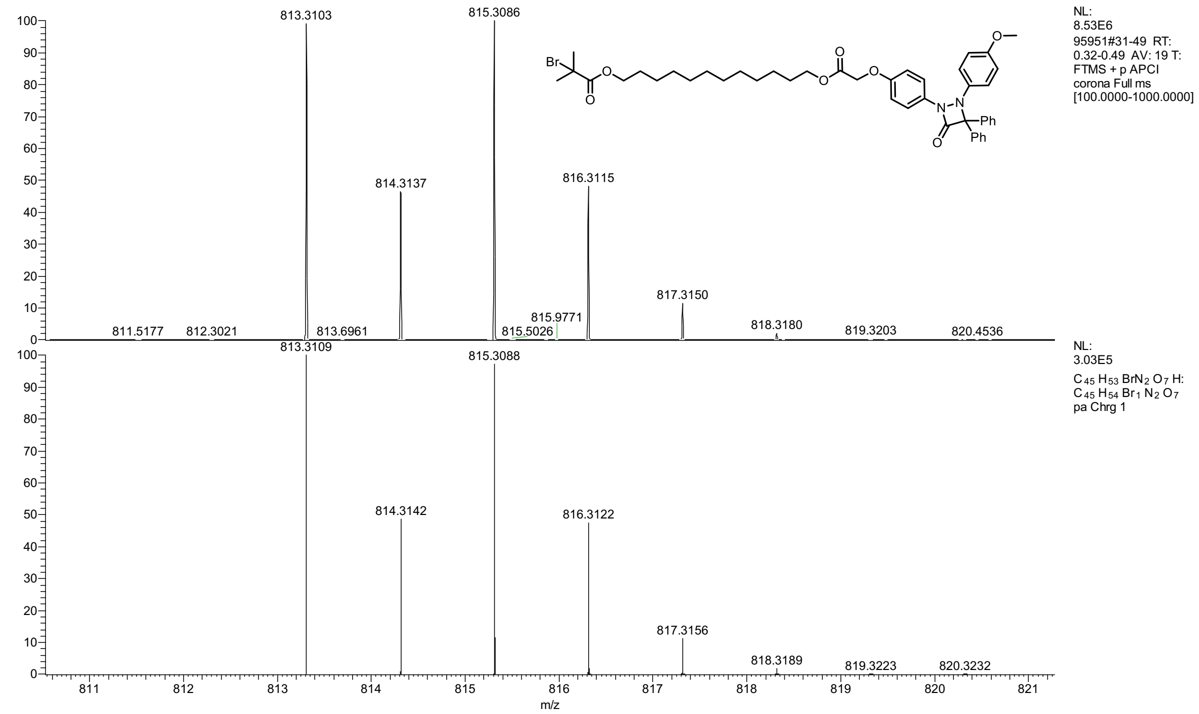

Spectrum S86. Isotopic distribution of **S12**. Top: Measured isotopic distribution for $C_{45}H_{53}BrN_2O_7H$ ($[M+H]^+$, + APCI). Bottom: Simulated isotopic distribution for $C_{45}H_{53}BrN_2O_7H^+$.

9.7 Isotopic distribution of S13

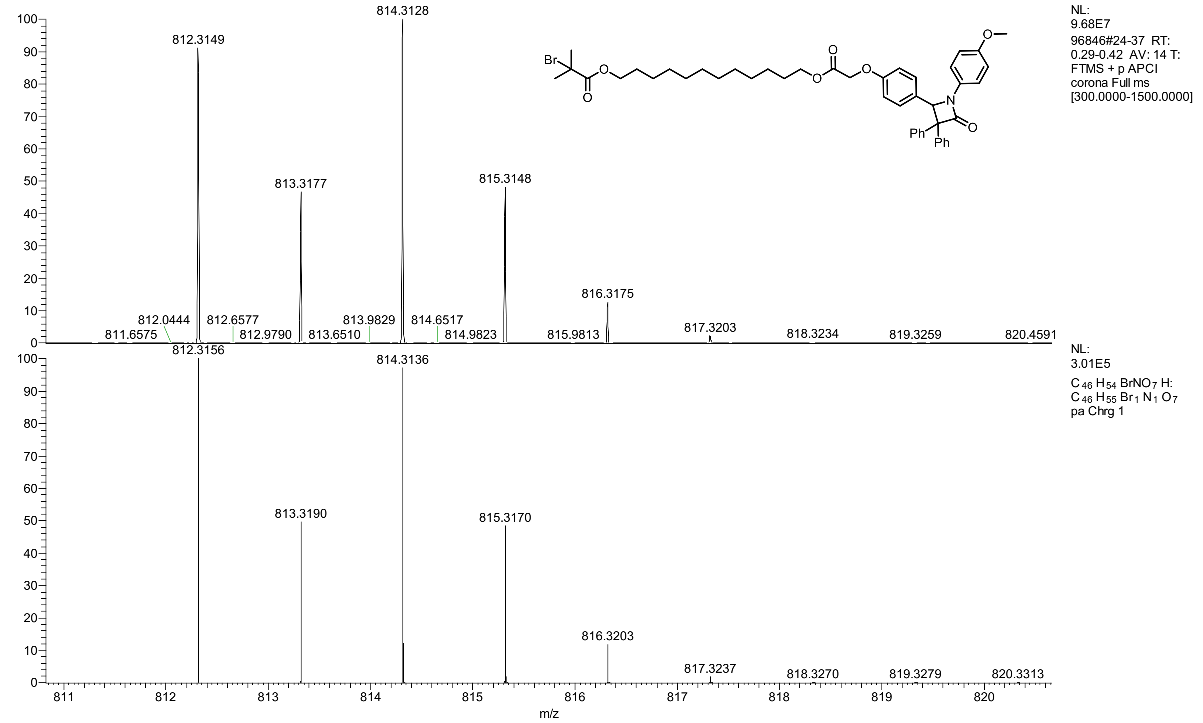

Spectrum S87. Isotopic distribution of **S13**. Top: Measured isotopic distribution for $C_{46}H_{54}BrNO_7H$ ([M+H]$^+$, + APCI). Bottom: Simulated isotopic distribution for $C_{46}H_{54}BrNO_7H^+$.

9.8 Isotopic distribution of S14

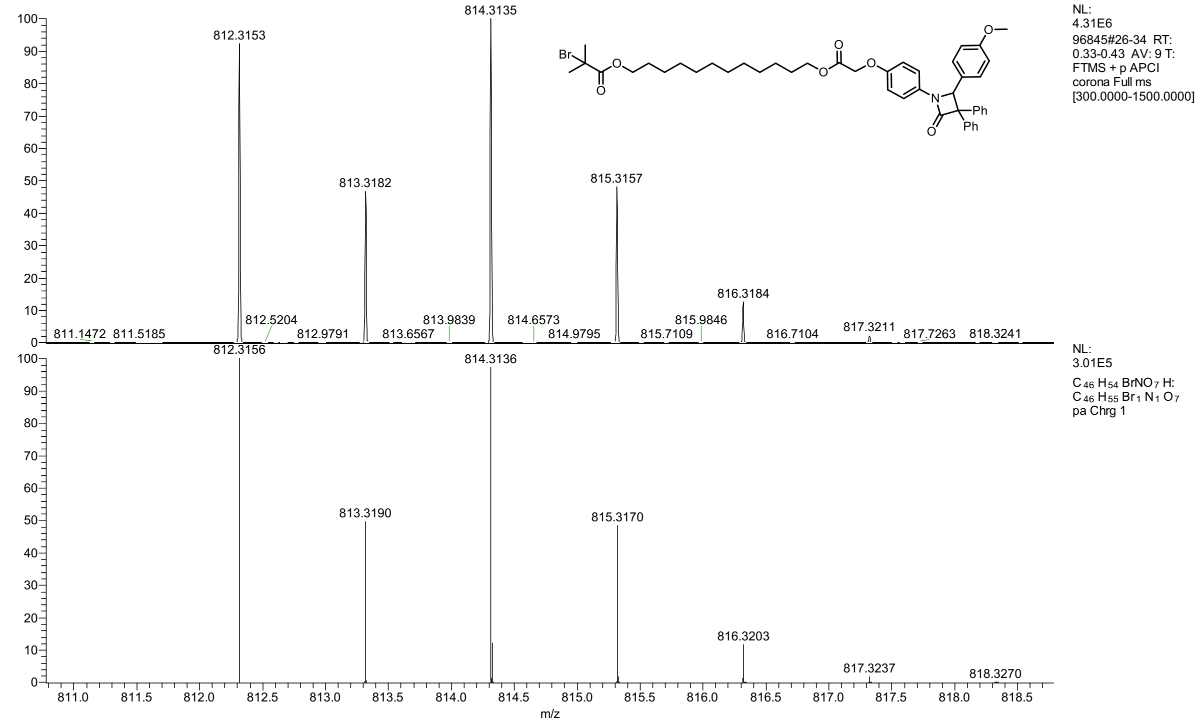

Spectrum S88. Isotopic distribution of **S14**. Top: Measured isotopic distribution for $C_{46}H_{54}BrNO_7H$ ([M+H]$^+$, + APCI). Bottom: Simulated isotopic distribution for $C_{46}H_{54}BrNO_7H^+$.

10 References

[1] R. M. Parker, J. C. Gates, H. L. Rogers, P. G. Smith, M. C. Grossel, *J. Mater. Chem.* **2010**, *20*, 9118-9125.

[2] U. Schatzschneider, J. K. Barton, *J. Am. Chem. Soc.* **2004**, *126*, 8630-8631.

[3] J. Bhaumik, Z. Yao, K. E. Borbas, M. Taniguchi, J. S. Lindsey, *J. Org. Chem.* **2006**, *71*, 8807-8817.

[4] L. Chen, R. Nixon, G. De Bo, *Nature* **2024**, *628*, 320-325.

[5] M. D. Bowman, R. C. Jeske, H. E. Blackwell, *Org. Lett.* **2004**, *6*, 2019-2022.

[6] Y. Lin, C.-C. Chang, S. L. Craig, *Org. Chem. Front.* **2019**, *6*, 1052-1057.

[7] P. G. Bangar, P. R. Jawalkar, S. R. Dumbre, D. J. Patil, S. Iyer, *Appl. Organomet. Chem.* **2018**, *32*, e4159.

[8] C. R. Hickenboth, J. S. Moore, S. R. White, N. R. Sottos, J. Baudry, S. R. Wilson, *Nature* **2007**, *446*, 423-427.

[9] G. O. Jones, J. M. García, H. W. Horn, J. L. Hedrick, *Org. Lett.* **2014**, *16*, 5502-5505.

[10] M. K. Beyer, *J. Chem. Phys.* **2000**, *112*, 7307-7312.
